# Metal-free molecular editing of indole via tandem reaction: Access to 2-aryl-3-aryldiazenylindole for theranostic applications

**DOI:** 10.1016/j.isci.2025.113325

**Published:** 2025-08-08

**Authors:** Lin Zhang, Yonghong Liu, Rui Chen, Bin Wang, Azhar Iqbal, Weiwei Jin, Yu Xia, Shaofeng Wu, Ziren Chen, Penji Yan, Chenjiang Liu, Yonghong Zhang

**Affiliations:** 1 Urumqi Key Laboratory of Green Catalysis and Synthesis Technology, Key Laboratory of Oil and Gas Fine Chemicals, Ministry of Education & Xinjiang Uygur Autonomous Region, State Key Laboratory of Chemistry and Utilization of Carbon Based Energy Resources, College of Chemistry, Xinjiang University, Urumqi 830017, P.R. China; 2 Key Laboratory of Hexi Corridor Resources Utilization of Gansu Universities, College of Chemistry and Chemical Engineering, Hexi University, Zhangye 734000, P.R. China; 3 Department of Chemistry, Bacha Khan University, Charsadda, Pakistan

**Keywords:** chemistry, chemical engineering, catalysis, organic synthesis, computational chemistry

## Abstract

A practical molecular editing strategy has been established for the direct and consecutive C-3 diazenylation and C-2 arylation of indoles with aryltriazenes under metal-free and ambient conditions. A sequence of tandem reactions has been developed via the sustained release of aryldiazonium species from aryltriazenes to achieve the controlled multiple bond sequential cleavage and formation. This strategy enables the chemo-controlled consecutive C−H functionalization of indole at two adjacent reactive sites (C-2 versus C-3) without pre-functionalization of the reactive sites and even without the assistance of the directing group, metal catalyst, or ligand. Detailed mechanistic experiments and DFT (density functional theory) calculations supported a sequence of tandem reaction mechanisms. The synthetic importance of this methodology is evident from the simple operation, good functional group tolerance, gram-scale synthesis, late-stage modification of pharmaceuticals, and *in vitro* cytotoxicity evaluation.

## Highlights

• Direct consecutive indole C-3 diazenylation/C-2 arylation without metals or heating
• Tandem reactions via aryl diazonium release achieve multiple bond cleavage/formation
• No pre-functionalization, directing groups, metals, or ligands required
• Simple, scalable, good tolerance; drug modification; and bioactivity shown

## Graphical abstract

**Figure:**
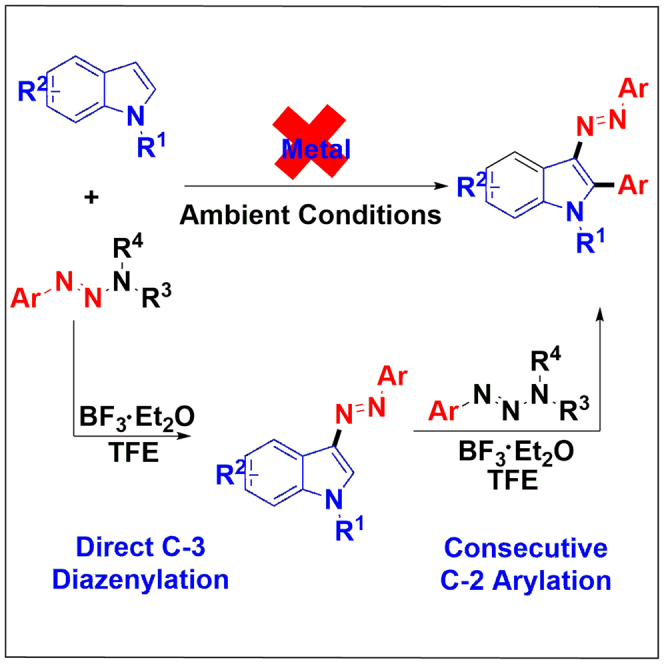

## Introduction

Selective introduction of multiple functional groups into the core skeleton of a molecule is the most direct but challenging strategy for the rapid generation of structural diversity.^1^ However, the simultaneous introduction of multiple functional groups into a molecule usually requires multi-step synthesis, which will inevitably decrease the synthesis efficiency, selectivity, and economy. Therefore, numerous one-pot synthesis methods for constructing molecular diversity were developed in recent years, such as multicomponent reactions,^2^^,^^3^ one-pot stepwise synthesis,^4^^,^^5^ cascade/tandem reaction,^6^^,^^7^^,^^8^^,^^9^ molecular editing strategy,^1^^,^^10^^,^^11^^,^^12^ etc. Especially, as a considered ideal synthesis approach, molecular editing strategy was used to direct modification of bicyclic aza-arenes at any given site by Yu group recently.^1^ In the past few years, molecular editing has been applied to the complex natural product synthesis, direct modification of valuable core structures, small-molecule functionalization, and drug modifications.^1^^,^^10^^,^^13^

**Scheme 1:**
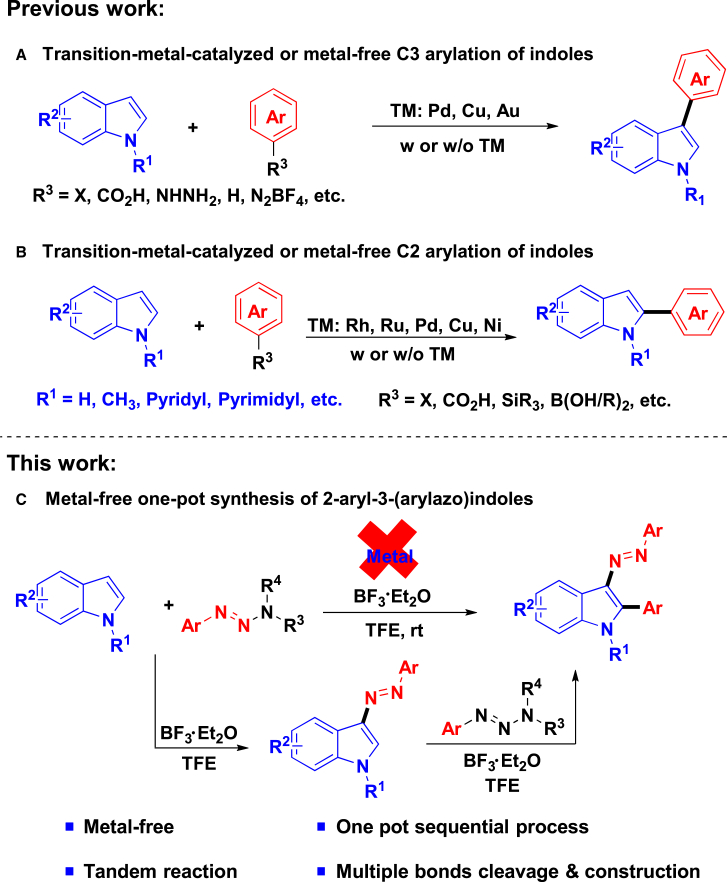
Various approaches for the direct and site-selective C-2 and C-3 C−H arylation of indoles (A) Transition-metal-catalyzed or metal-free C3 arylation of indoles. (B) Transition-metal-catalyzed or metal-free C2 arylation of indoles. (C) Metal-free one-pot synthesis of 2-aryl-3-(arylazo)indoles.

Indoles are privileged motifs among the most recurrent nitrogen-containing heterocycles in bioactive natural products and pharmaceutical drugs,^14^^,^^15^^,^^16^^,^^17^ which were ideal substrates for the development of skeletal editing reactions, as indole has six C−H bonds in its core including C-2 to C-7 positions that can be functionalized.^18^^,^^19^^,^^20^ Consequently, over the last few years, great efforts have been devoted to the site-selective and direct C−H functionalization of indole nucleus.^18^^,^^19^^,^^20^^,^^21^^,^^22^^,^^23^^,^^24^ In particular, a number of synthetic approaches were developed to access C-3 (Scheme 1A) or C-2 (Scheme 1B) functionalization of indoles.^18^^,^^21^^,^^25^^,^^26^^,^^27^^,^^28^ Among them, the site selectivity was mainly achieved by employing transition metal,^29^ organic catalyst,^30^ the combination of metal and ligand,^31^^,^^32^ or installation of directing groups (DGs)^33^; however, only limited strategies are available under the metal-free and mild conditions.^34^^,^^35^^,^^36^^,^^37^^,^^38^^,^^39^^,^^40^^,^^41^^,^^42^ On the other hand, these methods mostly rely on metal catalysts with ligands or DGs that could be a substantial limitation for their further application in large-scale syntheses and pharmaceutical development. Furthermore, to the best of our knowledge, metal- and DG-free methods of direct and selective introduction of different groups simultaneously to C-2 and C-3 positions of indole are rare. Due to the ubiquitous substructures and biological activity of C-2 or C-3 arylindoles and azo-containing heterocycles, the development of efficient method for the synthesis of diverse azo-containing arylated indoles is a high-priority aim. Early achievement made by Magedov group resulted in the successful synthesis of 2-aryl-3-(arylazo)indoles via one-pot stepwise C-3 diazenylation and C-2 arylation of the indole with aryl diazonium salts^43^; however, low yield and selectivity of the reaction, as well as the use of unstable and potentially explosive aryl diazonium salts, greatly restrict its application. Hence, based on our continuous study on the development of efficient and sustainable triazene and radical chemistry,^44^^,^^45^^,^^46^^,^^47^^,^^48^^,^^49^^,^^50^^,^^51^^,^^52^^,^^53^ herein, we envisioned that the site-selective C-3 diazenylation and C-2 arylation of indole could be achieved by using molecular editing strategy, which by replacing the aryl diazonium salts with aryltriazenes (masked diazonium surrogate) offers an alternative pathway to the sustained release of aryldiazonium species. Thus, a sequence of tandem reactions of consecutive C-3 diazenylation and C-2 arylation can be achieved (Scheme 1C).

## Results and discussion

### Optimization of the reaction conditions

As shown in Table 1, at the outset of the investigation, we employed *N*-methylindole 1a and *p*-methylphenyltriazene 2a as model substrates to optimize the reaction conditions. Initially, BF_3_·Et_2_O was used as a promoter to investigate the effect of solvent at room temperature. The results showed that halogenated organic solvents have good performance (entries 1–6). 2,2,2-Trifluoroethanol (TFE) afforded the desired product with the highest yield up to 86%; however, other halogenated solvents, such as 2,2,2-trichloroethanol (TCE), 1,1,1,3,3,3-hexafluoroisopropanol (HFIP), or dichloromethane (DCM) led to lower conversions. Furthermore, the yield of the desired product decreased dramatically when the reaction was carried out in ethanol or *N*,*N*-dimethylformamide (DMF). We speculate that acidic solvents also play a role in the activation of aryltriazenes. Subsequently, various Brønsted acids were screened to promote the reaction (entries 7–10); although most of the acids produce the desired product in good yields, the best choice is the Lewis acid BF_3_·Et_2_O. Finally, other factors were investigated to improve the yield of the desired product, which include elevating the reaction temperature (entry 11), increasing the amount of promoter (entry 12) and triazene (entry 13), and prolonging the reaction time (entry 14); however, there was no significant improvement in the yield. Furthermore, the concentration of the reaction system was also investigated; however, decreased yield was afforded when diluting the reaction solution (entry 15).

**Table 1:** Optimization of reaction conditions


Entry	Solvent	Promoter	Yield (%)^a^	
			**3a**	**4a**
1	TFE	BF_3_·Et_2_O	86 (82^b^)	7 (9^b^)
2	TCE	BF_3_·Et_2_O	43	15
3	HFIP	BF_3_·Et_2_O	56	38
4	DCM	BF_3_·Et_2_O	36	0
5	EtOH	BF_3_·Et_2_O	trace	20
6	DMF	BF_3_·Et_2_O	n.r.	n.r.
7	TFE	HBF_4_	76	17
8	TFE	HFP_6_	81	14
9	TFE	TFA	65	30
10	TFE	AcOH	37	55
11^c^	TFE	BF_3_·Et_2_O	85	6
12^d^	TFE	BF_3_·Et_2_O	83	10
13^e^	TFE	BF_3_·Et_2_O	87	9
14^f^	TFE	BF_3_·Et_2_O	86	6
15^g^	TFE	BF_3_·Et_2_O	49	15

a Reaction conditions: 1a (0.2 mmol) and 2a (0.7 mmol) were dissolved in 1.0 mL of solvent, and then a solution of promoter (0.12 mmol) in 1.0 mL solvent was added dropwise in 30 min and stirred at room temperature for 7 h. Yields were determined by HPLC.

b Isolated yield.

c At 60°C.

d Promoter (0.16 mmol).

e 2a (1.0 mmol).

f 12 h.

g 4.0 mL solvent.

Once the reaction conditions were optimized, we investigated the effects of different *N*-substituted groups of aryltriazenes on the yield of the reaction (Table 2). For cyclic substitution, the ring size and insertion of an additional heteroatom into the ring exhibited almost no effect on the yields (entries 1–3). In addition to the cyclic substitutions, the acyclic ones were then screened (entries 4–15), and the results showed that the acyclic substituents with low steric hindrance have similar results to the cyclic ones (entries 4–10). However, the more sterically hindered substituents dramatically decreased the yield (entry 8). In addition, the diversified reactive groups, such as allyl (entry 9), hydroxyl (entry 10), and benzyl (entry 11), obtained moderate to good yields. Furthermore, a substrate with a cyclohexyl group on the nitrogen atom reacted smoothly as well (entry 12), but the yield was not improved. However, when the substituent group was switched to sp^2^ substitution, the yield of the desired product decreased dramatically, which may be caused by the conjugation effects between the π-electrons on the phenyl and the lone-pair electron on the nitrogen atom of the triazene (entries 13–14). To our delight, a -CH_2_CH_2_- linked triazene dimer produced the corresponding product in 85% yield with lower substrate loading, and the yield was further improved to 92% when 0.5 mmol of triazene dimer was used (entry 15).

**Table 2:** Optimization of the N-substituted 1-aryltriazenes reaction conditions


Entry	R	Yield (%)^a^		Entry	R	Yield (%)^a^	
		3a	4a			3a	4a
1		86	7	9		67	10
2		85	9	10		78	11
3		85	8	11		84	8
4		85	7	12		77	16
5		83	10	13		29	17
6		80	11	14		n.r.	trace
7		83	9	15		85^b^	7^b^
8		65	11			92^c^	2^c^

a Reaction conditions: 1a (0.2 mmol) and 2 (0.7 mmol) were dissolved in 1.0 mL of TFE, and then a solution of BF_3_·Et_2_O (0.12 mmol) in 1.0 mL TFE was added dropwise in 30 min and stirred at room temperature for 7 h. Yields were determined by HPLC.

b 2 (0.35 mmol).

c 2 (0.5 mmol).

### Substrate scope of *N*-substituted 1-aryltriazenes

**Scheme 2:**
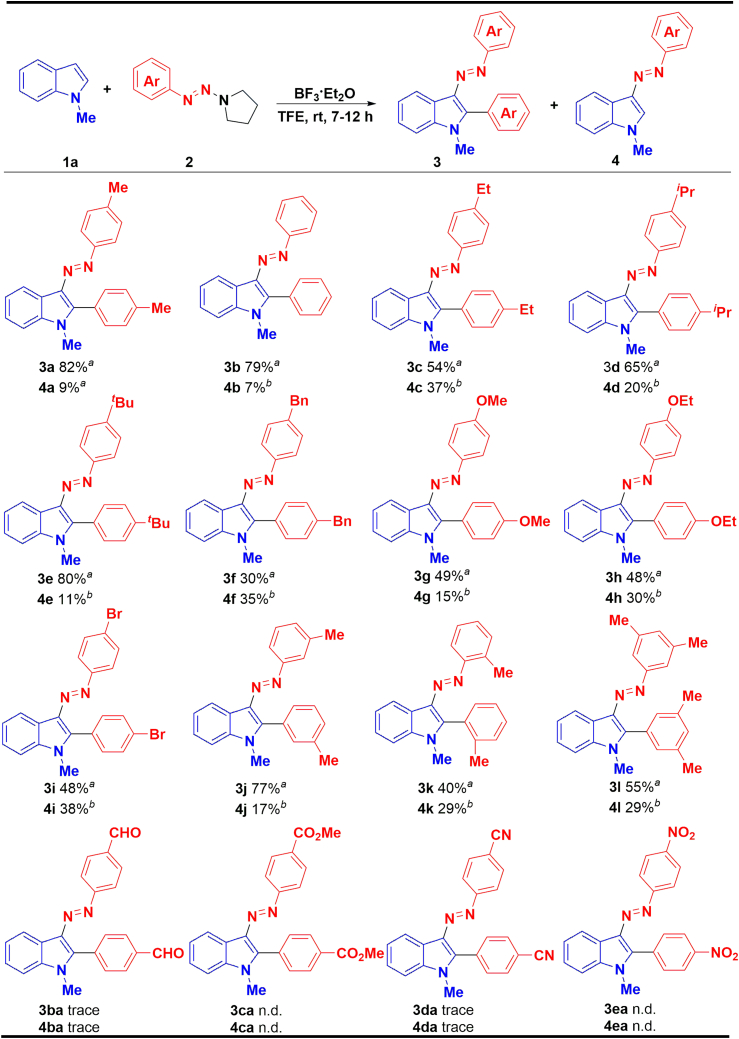
The scope of substituted 1-aryltriazenes ^a^Reaction conditions: 1a (0.2 mmol) and 2 (0.7 mmol) were dissolved in 1.0 mL of TFE, and then a solution of BF_3_·Et_2_O (0.12 mmol) in 1.0 mL TFE was added dropwise in 30 min and stirred at room temperature for 7–12 h, isolated yield. ^*b*^Yields were determined by HPLC.

With the identified conditions in hand, firstly, we expanded the substrate of *N*-tetrahydropyrrole substituted 1-aryltriazenes (Scheme 2). The reaction was generally effective for both electron-donating and electron-withdrawing substituents, affording moderate to good yields. Phenyltriazene (2b), 4-Me (2a), and 4-^*t*^Bu (2e) substituted aryltriazenes have excellent yields. For 4-Et (2c), 4-^*i*^Pr (2d), 4-OMe (2g), 4-OEt (2h), and 4-Br (2i) substituted aryltriazenes in moderate yields. However, when the substituent group of triazene is 4-Bn, only 30% yield is obtained (3f). The reaction also gave the desired product for *meta*-position substituted substrate, with no significant difference in yield compared with the *para-* position products (3j vs*.* 3a); however, when the substituent is in the *o*-position (3k), or 3,5- positions (3l), yields of 40% and 55% were obtained due to steric hindrance. Arytriazenes bearing strong electron-withdrawing substituents, such as CHO, CO_2_Me, CN, and NO_2_, were also tested; unfortunately, none of them afford the desired product.

**Scheme 3:**
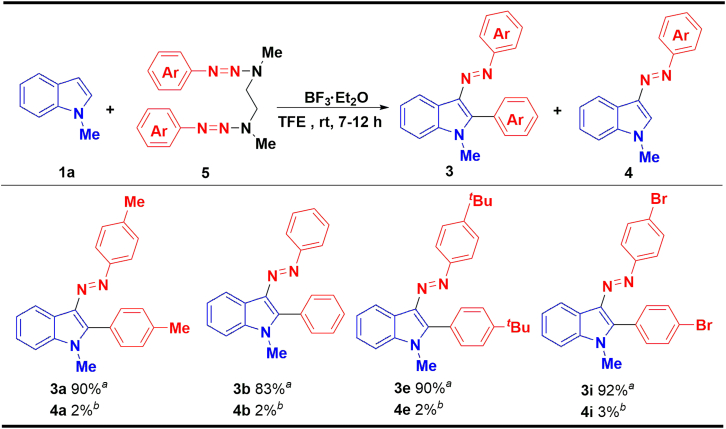
The scope of aryltriazene dimers ^a^Reaction conditions: (0.2 mmol) and **5** (0.5 mmol) were dissolved in 1.0 mL of TFE, and then a 1a solution of BF_3_·Et_2_O (0.12 mmol) in 1.0 mL TFE was added dropwise in 30 min and stirred at room temperature for 7–12 h, isolated yield. ^b^Yields were determined by HPLC.

Furthermore, in order to further improve the yield of 2-aryl-3-(arylazo)indoles, several -CH_2_CH_2_- linked aryltriazenes were selected for substrate universality studies (Scheme 3). Surprisingly, the yields of desired product with 4-methylphenyl (5a), phenyl (5b), 4-*tert*-butylphenyl (5e), and 4-bromophenyl (5i) triazenes were increased to 83%–92%.

**Scheme 4:**
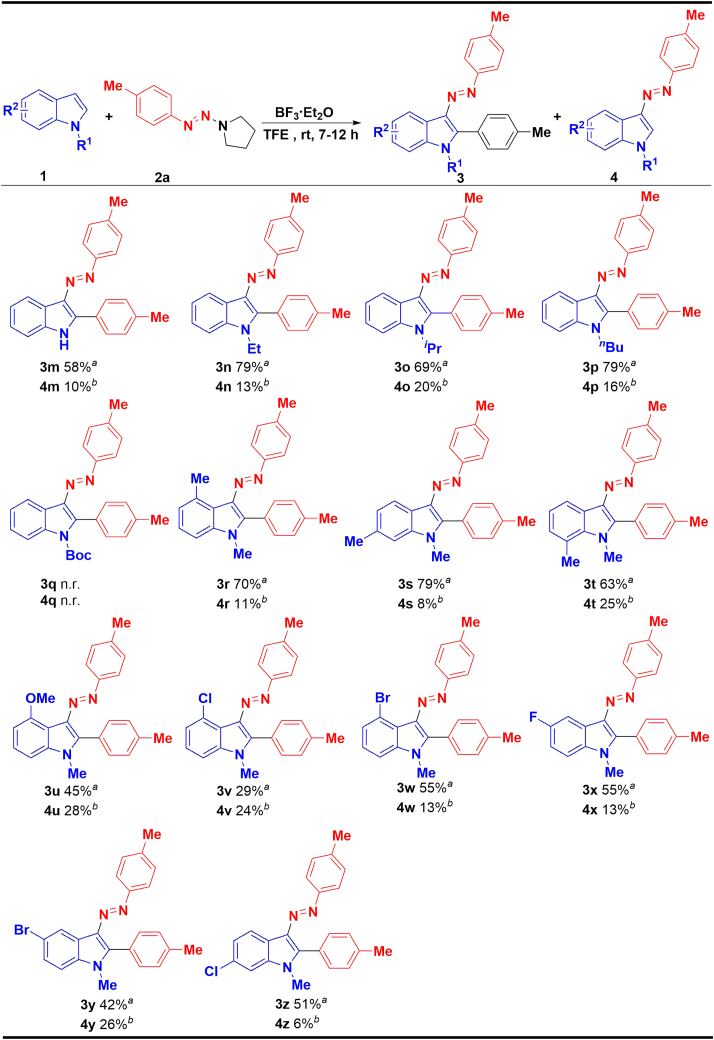
The scope of indoles and 1-(*p*-tolyldiazenyl)pyrrolidine ^a^Reaction conditions: 1 (0.2 mmol) and 2a (0.7 mmol) were dissolved in 1.0 mL of TFE, and then a solution of BF_3_·Et_2_O (0.12 mmol) in 1.0 mL TFE was added dropwise in 30 min and stirred at room temperature for 7–12 h, isolated yield. ^b^Yields were determined by HPLC.

**Scheme 5:**
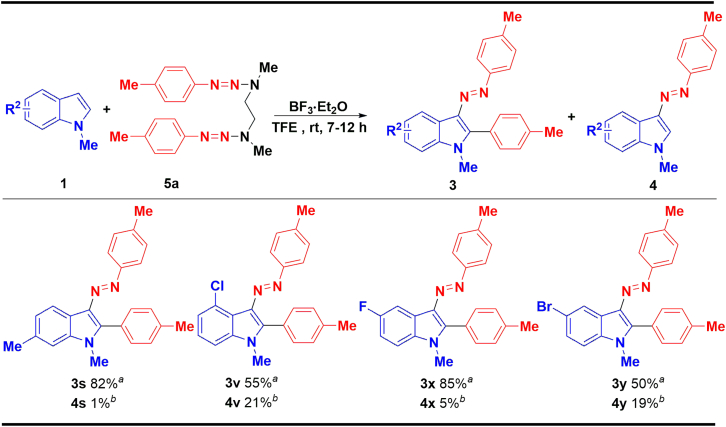
The scope of indoles and 1,2-bis(1-methyl-3-(*p*-tolyl)triaz-2-en-1-yl)ethane ^a^Reaction conditions: 1 (0.2 mmol) and 5a (0.7 mmol) were dissolved in 1.0 mL of TFE, and then a solution of BF_3_·Et_2_O (0.12 mmol) in 1.0 mL TFE was added dropwise in 30 min and stirred at room temperature for 7–12 h, isolated yield. ^b^Yields were determined by HPLC.

The substrate scope of various indoles with the established optimal method has also been evaluated (Scheme 4). For *N-*substituents, unprotected (1m) and electron-donating groups including Et- (1n), ^*i*^Pr- (1o), and ^*n*^Bu- (1p) substituted indoles delivered good yields, while electron-withdrawing substituent Boc-group (1q) failed to form the corresponding product. In general, substituents on C4-C7 of the indole’s benzene ring can obtain medium to good yields (3r-3z), for electron-donating substituents 4-CH_3_ (1r), 6-CH_3_ (1s), 7-CH_3_ (1t), and 4-OMe (1u) indole have good to excellent yields. Except for 4-Cl (3v)-substituted indoles that gave only 29% yield, other electron-withdrawing substituent such as 4-Br (1w), 5-F (1x), 5-Br (1y), and 6-Cl (1z) showed better tolerance (42%–55%). Considering the low yields of some examples, we still used 1,2-bis(1-methyl-3-(*p*-tolyl)triaz-2-en-1-yl)-ethane 5a instead of *p*-methylphenyltriazene 2a, and the results are summarized in Scheme 5. To our delight, the 6-Me and 5-F substituted indole obtained the desired products 3s and 3x in good yield (82% and 85%, respectively). However, 4-Cl and 5-Br substituted indole did not gave positive results; the corresponding products 3v and 3y were produced in lower yield (55% and 50%, respectively).

### Late-stage modification of sulfonamides

**Scheme 6:**
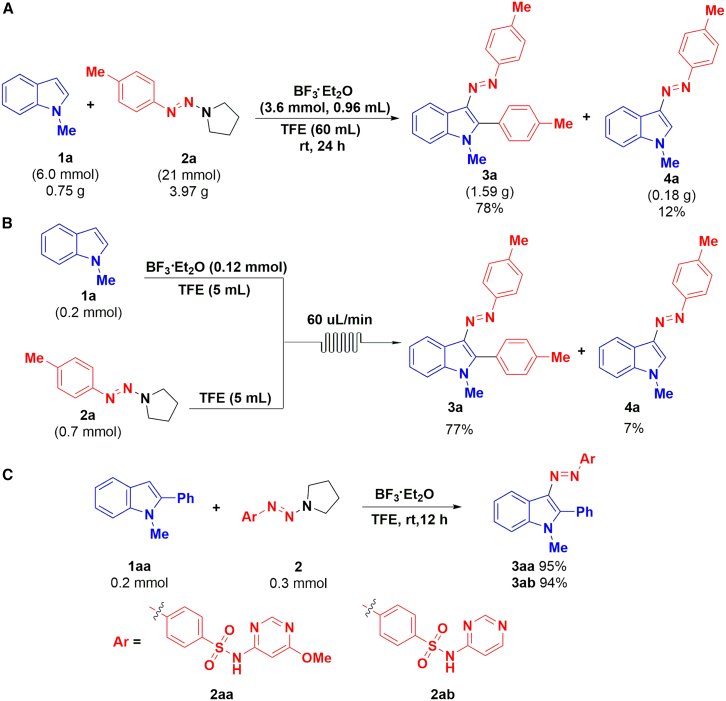
Gram-Scale reaction, flow chemistry experiments and late-stage modification of sulfonamides Gram scale in batch (A), and continuous flow synthesis (B) of (*E*)-1-methyl-2-(*p*-tolyl)-3-(*p*-tolyldiazenyl)-1*H*-indole, and late-stage modification of sulfonamides (C).

In order to further explore the efficiency and practicability of the protocol, gram-scale experiments (Scheme 6A), continuous flow synthesis (Scheme 6B), and late-stage modification of drugs (Scheme 6C) were carried out under modified reaction conditions. The results showed that this protocol could be successfully performed on gram scale; the desired product 3a was obtained smoothly in 78% yield at room temperature for 12 h. Recently, in view of its advantages such as safety, efficiency, and convenience, flow chemistry has attracted extensive attention.^54^^,^^55^ It can fully mix the reactants and reduce the occurrence of side reactions. Therefore, in order to compare continuous flow chemistry process with traditional batch processes, the direct and consecutive C-3 diazenylation and C-2 arylation of indoles with aryltriazenes were carried out under continuous flow conditions. Briefly, a solution of indole 1a (0.04 M) and promoter BF_3_·Et_2_O in TFE was pumped through the precooling loop (V = 10 mL) channel at a flow rate of 60 μL/min, where it was mixed with a solution of *p*-methylphenyltriazene 2a (0.14 M) in TFE at the same flow rate, and finally pumped into the micro-flow cell. Finally, the corresponding product 3a was obtained in 77% isolated yield (Scheme 6B).

**Figure 1:**
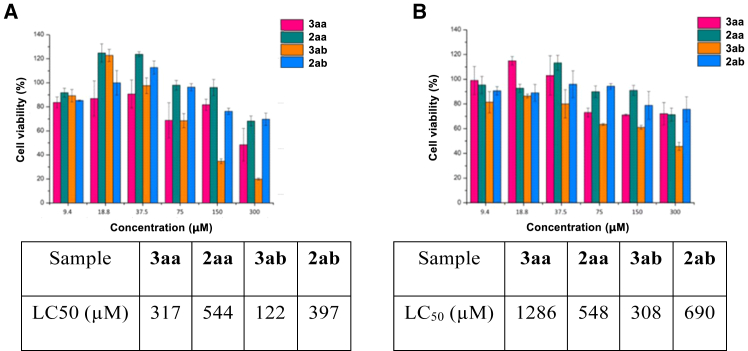
Inhibition efficacy-MTT assays (A) The cytotoxicity of HeLa cells; (B) The cytotoxicity of MRC-5; (The values in bars are presented as average ± standard deviation of three independent measurements).

**Figure 2:**
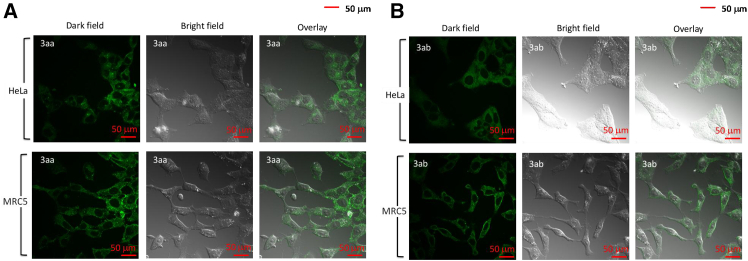
*In vitro* imaging of 3aa and 3ab (A and B) 10 μM, l_ex_ = 408 nm, scale bar length = 50 μm in HeLa cells (A) and MRC-5 cells (B) for 24 h incubation.

The 2-arylindole skeleton has been reported to have potential bactericidal activity. As sulfadiazine drugs are commercially available as antibacterial and anti-inflammatory drugs, we attempted to introduce sulfadiazine skeleton into 2-arylindole and test its potential biological activity. Here, late-stage modification sulfadiazine drug candidates with the developed method by using 1-methyl-2-phenyl-1*H*-indole 1aa as starting material was investigated to test the potential bioactivity of modified drugs.^24^ The desired products (*E*)-*N*-(5-methoxypyrimidin-4-yl)-4-((1-methyl-2-phenyl-1*H*-indol-3-yl)diazen-yl)-benze-nesulfonamide 3aa and (*E*)-4-((1-meth-yl-2-phenyl-1*H*-indol-3- yl)diazenyl)-*N*-(pyrimidin-4-yl)benzenesulfonamide 3ab were afforded under modified reaction conditions (Scheme 6C). The cytotoxicity of these sulfonamides before and after modification was then tested by using the MTT (methylthiazolyldiphenyl-tetrazolium bromide) viability assays with HeLa and MRC-5 cells (Figure 1). In the cytotoxicity experiment, modified sulfonamides demonstrate stronger cellular toxicity (3aa, LC_50_ [Lethal Concentration 50]) = 317 μM; 3 ab, LC_50_ = 122 μM) than unmodified sulfonamides (2aa, LC_50_ = 544 μM; 2 ab, LC_50_ = 397 μM) in HeLa cells (Figure 1A). These results show that the toxicity of the modified sulfonamides is potent against HeLa cells while they are relatively less cytotoxic in normal MRC-5 cells (3aa, LC_50_ = 1286 μM; 3 ab, LC_50_ = 308 μM) (Figure 1B). To explore the application of these modified sulfonamides as biomarkers, *in vitro* confocal imaging experiments were performed at 408 nm excitation (Figure 2). To our delight, all the modified sulfonamides could penetrate the cell membrane and label HeLa and MRC-5 cells with green luminescence.

### Mechanistic studies

**Scheme 7:**
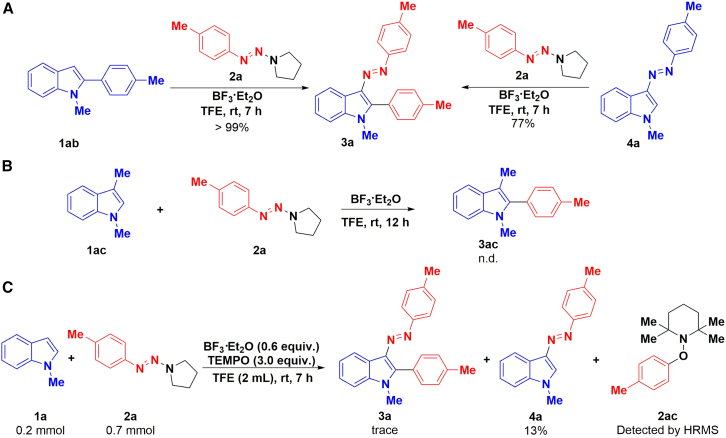
Control experiments (A–C) Reaction conditions: (A) 1ab or 4a (0.2 mmol), and 2a (0.3 mmol), were dissolved in 1.0 mL of TFE, then a solution of BF_3_·Et_2_O (0.12 mmol) in 1.0 mL TFE was added dropwise in 30 min, and stirred at room temperature for 7 h, isolated yield; (B) 1ac (0.2 mmol), and 2a (0.3 mmol), were dissolved in 1.0 mL of TFE, then a solution of BF_3_·Et_2_O (0.12 mmol) in 1.0 mL TFE was added dropwise in 30 min, and stirred at room temperature for 12 h, isolated yield; (C) 1a (0.2 mmol), 2a (0.7 mmol), and TEMPO (3.0 equiv., 0.6 mmol) were dissolved in 1.0 mL of TFE, then a solution of BF_3_·Et_2_O (0.12 mmol) in 1.0 mL TFE was added dropwise in 30 min, and stirred at room temperature for 7 h, isolated yield.

**Figure 3:**
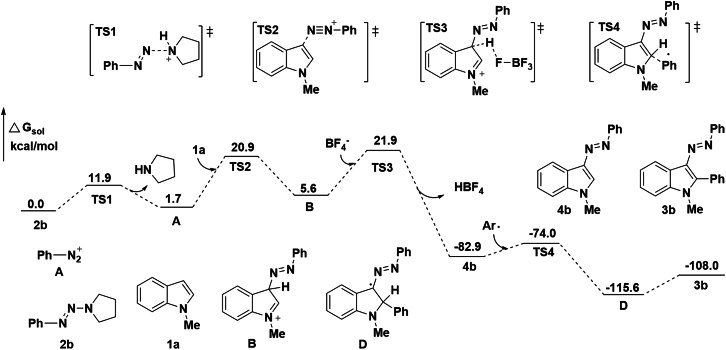
Schematic diagram showing the computed relative energy (in kcal/mol)

To gain insight into the reaction process, we carried out control experiments. Under standard conditions, two possible intermediates 4a and 1ab were used to react with aryltriazene. It was found that the diazenylation of 2-arylindole was very easy, and it could be almost completely transformed into the product 3a, while the arylation of 3-arylazoindole was slow and only 77% yield was obtained (Scheme 7A). Density functional theory (DFT) calculations predicted that the energy barrier of the diazenylation was reduced by 84.6 kcal/mol, while the energy barrier of the arylation was reduced by 25.1 kcal/mol. Therefore, the diazenylation process is more favorable, and the rate-limiting step in the transformation is the arylation reaction (Figure 3). In addition, 1,3-dimethylindole 1ac was used to react with aryltriazene; however, no arylation product was formed (Scheme 7B). Furthermore, HPLC was used to detect the conversion of the reaction mixture at intervals and found that most of the *N*-methyl indole was converted to the diazenylation product 4a at first. When prolonging reaction time, the increase of 3a was accompanied by the decrease of 4a. After 2 h, the increase of 3a was slowed down until the reaction hardly increased at 7 h. In addition, 2-arylindole 1ab was not detected during the reaction (see Figure S2). The aforementioned results showed that a consecutive C-3 diazenylation and C-2 arylation tandem reaction with indoles and aryltriazenes was involved in this method, and the C2 aryl of indole is the crucial step of the reaction. Moreover, the arylazo group attached to the C-3 position of indole plays a decisive role in C-2 arylation.

**Figure 4:**
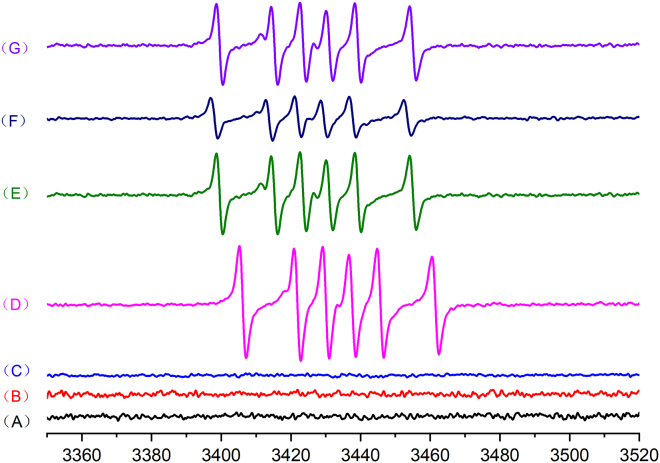
Electron paramagnetic resonance spectrum A solution of DMPO (5,5-dimethyl-1-pyrroline *N*-oxide) and TFA (trifluoroacetic acid) as promoter, in TFE (A) TFE and TFA; (B) 1a and TFE; (C) 1a, TFE, and TFA; (D) 2a and TFE; (E) 2a, TFE, and TFA; (F) 1aa and 2a at standard conditions; and (G) 4a and 2a at standard conditions.

To gain insight into the mechanism of this process, several radical-trapping experiments were carried out. First, when the reactions were conducted in the presence of 3.0 equiv. of 2,2,6,6-tetramethyl-1-piperidinyloxy (TEMPO) as the radical scavenger, only trace amounts of the desired products were detected (Scheme 7C). Moreover, electron paramagnetic resonance experiment was carried out next, as shown in Figure 4; in the absence of *p*-methylphenyltriazene 2a in the reaction system, no free radical signal was observed (Figures 4B and 4C). Surprisingly, free radical signal was detected when aryltriazene was added to the reaction mixture with or without the presence of promoter (Figures 4D and 4E). The results indicated that aryl radicals can be generated in the presence or absence of promoters. Therefore, various solvents were used to confirm that aryltriazene can be activated by some solvent under promoter-free conditions. The product 3a can be obtained in 37% yield in the presence of protic solvent TFE. However, aprotic solvents failed to promote reaction. The results indicated that the weakly acidic protic solvent TFE can activate aryltriazene (see Table S1). In addition, the combination of intermediate *N*-methyl-3- (4-tolyldiazenyl)- indole 4a and aryltriazene can also generate aryl radical signals under standard conditions (Figure 4G). Furthermore, TEMPO-adducted product 2ac was detected by high-resolution mass spectrometry (HRMS) (see Figure S3). The aforementioned results showed that aryl radicals were generated and involved in this transformation.

**Scheme 8:**
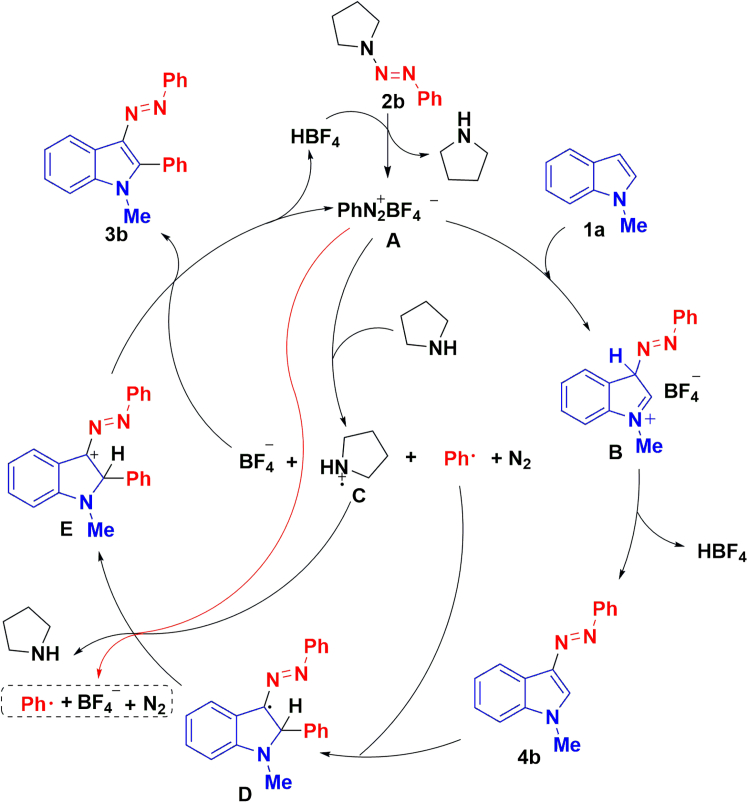
Possible reaction mechanism

Based on the aforementioned results and literature reports,^43^^,^^56^^,^^57^^,^^58^ a plausible reaction mechanism is presented in Scheme 8, which is also rationalized by the DFT calculations of the possible reaction mechanism (Figure 3). Phenyltriazene 2b was activated by acid to release phenyldiazonium A, which acts as the starting point of the reaction cycle. The electrophilic addition reaction on C-3 of indole ring with phenyldiazonium A was proceeded with an energy barrier of 20.9 kcal/mol. The intermediate B was deprotonation and rearomatization to 3-phenylazoindole 4b under BF_4_^−^. Although the process requires overcoming an energy barrier of 16.3 kcal/mol through TS2, the exotherm from B to 4b is 88.5 kcal/mol. On the other hand, the phenyl radical and intermediate aminyl cation radical C were produced *in situ* via single electron transfer between phenyl diazonium salt A and tetrahydropyrrole. Next, the Meerwein-type arylation was proceeded at C-2 position of 3-phenylazoindole 4b with phenyl radical to afford radical intermediate D, with an energy barrier of 8.9 kcal/mol. Radical intermediate D was oxidized to cationic intermediate E by aminyl cation radical C or phenyl diazo salt A. Finally, the desired product 3b was generated by the deprotonation of intermediate E with the assistance of BF_4_^−^ anion.

### Conclusions

In summary, we have developed a practical molecular editing strategy for the direct and consecutive C-3 diazenylation and C-2 arylation of indoles with aryltriazenes under metal-free and ambient conditions. A sequence of tandem reaction was carried out with aryltriazene as a safe equivalent of diazonium salt and electron shuttle to release the aryl-azo cation and aryl radical *in situ*, respectively. This strategy enables the region- and chemo-controlled consecutive C−H functionalization of indoles at two adjacent reactive sites (C-2 versus C-3) without pre-functionalization of the reactive sites and even without the assistance of the DG, metal catalyst, or ligand. Detailed mechanistic experiments supported a sequence of tandem reaction mechanism with the controlled multiple bond sequential cleavage and formation. The synthetic importance of this methodology is evident from the simple operation, good functional group tolerance, gram-scale synthesis, late-stage modification of pharmaceuticals, and *in vitro* cytotoxicity evaluation.

### Limitations of the study

This study has not yet been applied to drug research or *in vivo* cell labeling experiments, and its application value in the biological field remains to be further explored.

## Resource availability

### Lead contact

Further information and requests for resources and reagents should be directed to and will be fulfilled by the lead contact Prof. Yonghong Zhang (zhzhzyh@126.com).

### Materials availability

This study did not generate new unique reagents. All the cell lines used in this manuscript will be made available upon request.

### Data and code availability

• All data reported in this paper will be shared by the lead contact upon request.
• This study codes are available in the key resources table.
• Any additional information required to reanalyze the data reported in this paper is available from the lead contact upon request.

## Acknowledgments

This work is supported by the Tianshan Talents Program for Young Talents in Science and Technology Innovation (no. 2022TSYCCX0024), the Natural Science Foundation for Distinguished Young Scholars of Xinjiang Uyghur Autonomous Region (no. 2021D01E10), the Shanghai Cooperation Organization Science and Technology Partnership Program and International Science and Technology Cooperation Program (no. 2022E01042), the National Natural Science Foundation of Gansu (no. 23JRRG0037), the Tianshan Talents Program for Leading Talents in Science and Technology Innovation (no. 2022TSYCLJ0016), and the 10.13039/501100001809National Natural Science Foundation of China (nos. 21861036, 21961037, 22201241, and 22361044).

## Author contributions

L.Z.: conceptualization, data curation, formal analysis, investigation, methodology, writing – original draft, and writing – review and editing. Y.L.: data curation, formal analysis, and investigation. R.C.: data curation, investigation, and methodology. B.W.: data curation and methodology. A.I.: writing – review and editing. W.J.: conceptualization and methodology. Y.X.: methodology. S.W.: conceptualization and methodology. Z.C.: conceptualization and methodology. P.Y.: data curation and funding acquisition. C.L.: conceptualization, funding acquisition, and methodology. Y.Z.: conceptualization, data curation, formal analysis, funding acquisition, investigation, methodology, and writing – review and editing.

## Declaration of interests

The authors declare no competing interests.

## STAR★Methods

### Key resources table

**Table:** 

REAGENT or RESOURCE	SOURCE	IDENTIFIER
**Biological samples**		

Human cervical carcinoma HeLa cells	Cell resource center of Shanghai Institute of Biological Sciences, Chinese Academy of Sciences	Cat#SCRC-1001; RRID:CVCL_0030; The HeLa cell line unique identifier: CSTR:19375.09.3101HUMSCSP504
Human normal lung fibroblast (MRC-5) cells	Cell resource center of Shanghai Institute of Biological Sciences, Chinese Academy of Sciences	Cat#SCRC-1041; RRID:CVCL_0440; The MRC-5 cell line unique identifier: CSTR:19375.09.3101HUMGNHu41

**Software and algorithms**		

ChemBioDraw 14	CambridgeSoft	https://www.perkinelmer.com/category/chemdraw
OriginPro 2021	OriginLab Corporation	https://www.originlab.com
MestReNova 14.0.0	Mestrelab Research S.L.	https://www.mestrelab.com
Gaussview 6.0	Gaussian, Inc	http://gaussian.com/gaussview6/
GraphPad Prism 5.0	GraphPad Software Inc	https://www.graphpad.com/

### Experimental model and study participant details

#### Cell lines

Human normal lung fibroblast (MRC-5) cells and Human cervical carcinoma HeLa cells were provided by Cell resource center of Shanghai Institute of Biological Sciences, Chinese Academy of Sciences. Human normal lung fibroblast (MRC-5) cells grown in Minimum Essential Medium (MEM) (GIBCO 41500034). Human cervical carcinoma HeLa cells were grown in Dulbecco’s Modified Eagle Medium (DMEM). The cell lines were kindly provided by Prof. Ka-Leung Wong (Department of Applied Biological and Chemical Technology, Hong Kong Polytechnic University). Both medium were supplemented with 10% fetal bovine serum, 1% penicillin and streptomycin. The cell lines were incubated at 37 °C in a humidified atmosphere containing 5% CO_2_. This study was non-human subjects research. The donor gender of the HeLa cells was female, and the gender information of MRC-5 was not clear. Cell lines, tested for mycoplasma contamination, were not authenticated.

### Method details

#### General information

Unless otherwise noted, all reagents and solvents were purchased from commercial sources (Adamas-beta, Energy Chemical) and used without further purification.

##### NMR spectrum

^1^H and ^13^C NMR spectra were collected on 400 MHz NMR spectrometers (Varian Inova-400) and 600 MHz NMR spectrometers (Bruker AVANCE NEO 600). Chemical shifts for protons were reported in parts per million (ppm) downfield from tetramethylsilane and were referenced to residual protium in the NMR solvents (CDCl_3_ = δ 7.26, DMSO-*d*_*6*_ = δ 2.50). Chemical shifts for carbon resonances were reported in parts per million (ppm) downfield from tetramethylsilane and were referenced to the carbon resonances of the solvents (CDCl_3_ = δ 77.00, DMSO-*d*_*6*_ = δ 39.70). The following abbreviations were used to describe peak splitting patterns when appropriate: s = singlet, d = doublet, t = triplet, q = quartet, m= multiplet. Coupling constants J were reported in hertz unit (Hz).

##### Melting point

Melting point (M.P.) was recorded on BÜCHI (M-560) and uncorrected.

##### HRMS

High-resolution mass spectra (HRMS) were recorded on Thermo Fisher Scientific Q Exactive.

##### Column chromatography

Column chromatography was undertaken on silica gel (300-400 mesh) using a proper eluent.

##### TLC

Analytical thin layer chromatography (TLC) was performed on 0.25 mm silica gel 60 F254 plates and viewed by UV light (254 nm). HPLC (high performance liquid chromatography).

##### HPLC

HPLC analysis using a Shimadzu HPLC2010, OD-H column, hexanes/*i*-PrOH = 90:10, 0.5 mL/min, 30 °C, λ = 254 nm).

##### EPR spectra

EPR spectra were recorded on a Bruker EMX plus spectrometer.

##### Synthesis of *N*-protected indoles

A mixture of indole derivative (2 mmol, 1.0 equiv.), NaOH (4 mmol, 2.0 equiv.) and 5 mL dry DMSO was added to a dried round bottom flask. Then, iodinated alkane (4 mmol, 2.0 equiv.) was added dropwise to the reaction mixture and the resulting mixture was stirred at room temperature for 16 h (See Scheme S1). When the reaction was complete, 5 mL of water was added to the mixture and the aqueous mixture was extracted with ethyl acetate (3×5 mL). The combined organic phase was dried over anhydrous MgSO_4_, filtered and concentrated under reduced vacuum. The residue was purified by silica gel column chromatography (petroleum ether / ethyl acetate 30:1-10:1) to afford the desired product.

##### Synthesis of Aryltriazenes

A solution of arylamine (20 mmol) in concentrated HCl (4 mL) was cooled in an ice bath while a solution of NaNO_2_ (20 mmol) in water (10 mL) was added dropwise. The resulting solution of the diazonium salt was stirred in an ice bath for 10 min and then added all at once to a chilled solution of amine (22 mmol) or dimethylethylenediamine (11 mmol) in 20 mL aqueous K_2_CO_3_ (1.2 M) . The reaction mixture was stirred for 30 min with cooling and the resulting precipitate was isolated by filtration (See Scheme S2). The damp solid was recrystallized from EtOH and dried under reduced pressure or by column chromatography.^59^

#### Experimental procedures

##### General procedure for the synthesis of products (taking **3a** and **4a** as an example)

A mixture of *N*-methylindole 1a (0.2 mmol, 25 μL), *p*-methylphenyltriazene 2a (0.7 mmol, 132.4 mg) and 2,2,2-trifluoroethanol (1.0 mL) was added to a dried tube. Then, a solution of BF_3_·Et_2_O (0.12 mmol, 32 μL) in 2,2,2-trifluoroethanol (1.0 mL) was added dropwise to the above stirred solution in 30 minutes. The reaction mixture was stirred at ambient temperature for 7 h (Table 1, Entry 1). When the reaction was completed, 5 mL of water was added to the mixture and the aqueous mixture was extracted with ethyl acetate (3×5 mL). The combined organic phase was dried over anhydrous MgSO_4_, filtered and concentrated under reduced pressure, the residue was purified by silica gel column chromatography (eluent: petroleum ether/EtOAc = 40:1 to 10:1, v/v) to afford the desired product 3a as a yellow semisolid (55.8 mg, 82% yield) and 4a as a yellow semisolid (4.4 mg, 9% yield).

##### Gram-scale reaction

A mixture of *N*-methylindole 1a (6.0 mmol, 0.75 mL), *p*-methylphenyltriazene 2a (21.0 mmol, 3.97 g) and 2,2,2-trifluoroethanol (30.0 mL) was added to a dried round bottom flask. Then, a solution of BF_3_·Et_2_O (3.6 mmol, 0.96 mL) in 2,2,2-trifluoroethanol (30.0 mL) was added dropwise to the above stirred solution over 30 minutes. The reaction mixture was stirred at ambient temperature for 12 h (Scheme 6A). When the reaction was finished, 20 mL of water was added to the mixture and the aqueous mixture was extracted with ethyl acetate (3×20 mL). The combined organic phase was dried over anhydrous MgSO_4_, filtered and concentrated under reduced pressure, the residue was purified by silica gel column chromatography (eluent: petroleum ether/EtOAc = 40:1 to 10:1, v/v) to afford the desired product 3a as a yellow semisolid (1.59 g, 78% yield) and 4a as a yellow semisolid (0.18 g, 12% yield).

##### Flow chemistry

A mixture of *N*-methylindole 1a (0.2 mmol, 25 μL, 0.04 M), BF_3_·Et_2_O (0.12 mmol, 32 μL), and 2,2,2-trifluoroethanol (5.0 mL) were added to a dried 10 mL beaker, the mixture was stirred and premixed. The mixture was injected into a sampling syringe. A mixture of *p*-methylphenyltriazene 2a (0.7 mmol) and 2,2,2-trifluoroethanol (5.0 mL) was added to another dried 10 mL beaker and stirred (See Figure S1). The mixture was injected into another sampling syringe. The samples were mixed and flowed through the micro-reactor at a flow rate of 60 μL/min (Scheme 6B). When the reaction was finished, 5 mL of water was added to the mixture and the aqueous mixture was extracted with ethyl acetate (3×5 mL). The combined organic phase was dried over anhydrous MgSO_4_, filtered and concentrated under reduced pressure, the residue was purified by silica gel column chromatography (eluent: petroleum ether/EtOAc = 40:1 to 10:1, v/v) to afford the desired product 3a as a yellow semisolid 3a (52.2 mg, 77% yield) and 4a as a yellow semisolid (3.5 mg, 7% yield).

##### Late stage modification of sulfonamides

A mixture of *N*-methyl-2-phenyl indole 1aa (0.2 mmol, 41.4 mg), *p*-pyrimidine sulfatriazene 2aa or 2ab (0.3 mmol) and 2,2,2-trifluoroethanol (1.0 mL) were added to a dried tube and stirred at room temperature. Then, a solution of BF_3_·Et_2_O (0.12 mmol, 32 μL) in 2,2,2-trifluoroethanol (1.0 mL) was added dropwise to the above stirred solution in 30 minutes. The reaction mixture was stirred at ambient temperature for 12 h (Scheme 6C). When the reaction was finished, 5 mL of water was added to the system, and the solid was obtained by vacuum filtration. The crude product was purified by recrystallized with ethanol to afford the desired products 3aa (94.6 mg, 95% yield), and 3ab (88.0 mg, 94% yield).

##### *In vitro* imaging

HeLa and MRC-5 cells were seeded on 35 mm glass-bottomed culture dishes for 24 h. The cells were then incubated with 10 μM 3aa or 3ab in the dark for 24 h. The cells were washed twice with PBS and fresh medium was changed before performing confocal imaging. The 408 nm laser of the Nikon Eclipse Ti2 Confocal Microscope was used to take the *in vitro* images.

##### *In vitro* cytotoxic assays

The MTT viability assay was performed according to standard methods. In general, HeLa and MRC-5 cells were seeded on 96-well plates and incubated overnight. The cells were then treated with 3aa, 2aa, 3ab and 2ab at 37 °C 5% CO_2_ for 24 h. Cytotoxicity was determined by the MTT reduction assay. The cell monolayers were rinsed with PBS and then incubated with MTT, 3-(4,5-dimethylthiazol-2-yl)-2,5-diphenyltetrazolium bromide solution (0.5 mg·mL^-1^) at 37 °C for 3 h. Then, the solution was removed, and 100 μL of dimethylsulfoxide (DMSO) solubilizing reagent was added before shaking for 30 min to dissolve the formazan crystals produced in the living cells. The absorbance of the formazan crystals was measured at 540 nm and 690 nm, utilizing a dual-wavelength Labsystem Multiskan microplate reader (Merck Eurolab).

#### Control experiment

##### Synthesis of 3a with 1ab and 2a

A mixture of *N*-methyl-2-(4-methylphenyl)indole 1ab (44.2 mg, 0.2 mmol), *p*-methylphenyltriazene 2a (56.7 mg, 0.3 mmol) and 2,2,2-trifluoroethanol (1.0 mL) was added and stirred at ambient temperature. Then, a solution of BF_3_·Et_2_O (0.12 mmol, 32 μL) in 2,2,2-trifluoroethanol (1.0 mL) was added dropwise to the above stirred solution in 30 minutes. The reaction mixture was stirred at ambient temperature for 7 h (Scheme 7A, left side). When the reaction was finished, 5 mL of water was added to the mixture and the aqueous mixture was extracted with ethyl acetate (3×5 mL). The combined organic phase was dried over anhydrous MgSO_4_, filtered and concentrated under reduced pressure, the residue was purified by silica gel zuocecolumn chromatography (eluent: petroleum ether/EtOAc = 40:1 to 10:1, v/v) to afford the desired product 3a as a yellow semisolid (67.5 mg, > 99% yield).

##### Synthesis of **3a** with **4a** and **2a**

A mixture of *N*-methyl-3-(4-methylphenyldiazen- yl)indole 4a (49.8 mg, 0.2 mmol), *p*-methylphenyltriazene 2a (56.7 mg, 0.3 mmol) and 2,2,2-trifluoroethanol (1.0 mL) was added and stirred at ambient temperature. Then, a solution of BF_3_·Et_2_O (0.12 mmol, 32 μL) in 2,2,2-trifluoroethanol (1.0 mL) was added dropwise to the above solution in 30 minutes. The reaction mixture was stirred at ambient temperature for 7 h (Scheme 7A, right side). When the reaction was finished, 5 mL of water was added to the mixture, the aqueous mixture was extracted with ethyl acetate (3×5 mL). The combined organic phase was dried over anhydrous MgSO_4_, filtered and concentrated under reduced pressure, the residue was purified by silica gel column chromatography (eluent: petroleum ether/EtOAc = 40:1 to 10:1, v/v) to afford the desired product 3a as a yellow semisolid (52.4 mg, 77% yield).

##### The conversion rate of **1a**, **3a**, and **4a** versus time

A mixture of *N*-methylindole 1a (0.2 mmol, 25 μL), *p*-methylphenyltriazene 2a (0.7 mmol, 132.4 mg) and 2,2,2-trifluoroethanol (1.0 mL) was added and stirred at ambient temperature. Then, a solution of BF_3_·Et_2_O (0.12 mmol, 32 μL) in 2,2,2-trifluoroethanol (1.0 mL) was added dropwise to the above stirred solution in 30 minutes (See Scheme S3). Samples were taken at 0 h, 0.25 h, 0.5 h, 1 h, 2 h, 3 h, 4 h, 5 h, 6 h, 7 h, 8 h, and 9 h to measure the conversion rates of 1a, 3a, and 4a by HPLC, respectively. The results showed that 1a was initially converted to 4a. As the reaction progressed, the amount of 4a converted to 3a gradually decreased, and the amount of 3a increased. No C2 arylated product 1ab was detected during the reaction (See Figure S2).

##### The C2 arylation of 3-methylindole with *p*-methylphenyltriazene **2a**

A mixture of 1,3-dimethylindole 1ac (29.0 mg, 0.2 mmol), *p*-methylphenyltriazene 2a (56.7 mg, 0.3 mmol) and 2,2,2-trifluoroethanol (1.0 mL) was added and stirred at ambient temperature. Then, a solution of BF_3_·Et_2_O (0.12 mmol, 32 μL) in 2,2,2-trifluoroethanol (1.0 mL) was added dropwise to the above stirred solution in 30 minutes. The reaction mixture was stirred at ambient temperature for 12 h (Scheme 7B). TLC showed no reaction.

##### Radical trapping experiments

A mixture of *N*-methylindole 1a (0.2 mmol, 25 μL), *p*-methylphenyltriazene 2a (0.7 mmol, 132.4 mg), TEMPO (0.6 mmol, 93.8 mg) and 2,2,2-trifluoroethanol (1.0 mL) was added and stirred at ambient temperature. Then, a solution of BF_3_·Et_2_O (0.12 mmol, 32 μL) in 2,2,2-trifluoroethanol (1.0 mL) was added dropwise to the above stirred solution in 30 minutes. The reaction mixture was stirred at ambient temperature for 7 h (Scheme 7C). When the reaction was finished, 5 mL of water was added to the mixture and the aqueous mixture was extracted with ethyl acetate (3×5 mL). The combined organic phase was dried over anhydrous MgSO_4_, filtered and concentrated under reduced pressure, the residue was purified by silica gel column chromatography (eluent: petroleum ether/EtOAc = 40:1 to 10:1, v/v) to afford the product 4a (6.4 mg, 13% yield). TLC showed that only trace 3a was formed and the aryl radical-TEMPO adduct was detected by HRMS. HRMS (ESI^+^): *m/z* calcd. for C_23_H_24_N_3_ [M+H]^+^: 248.2009, found: 248.2002.

A mixture of *N*-methyl-3-(4-methylphenyldiazenyl)indole 4a (49.8 mg, 0.2 mmol), *p*-methylphenyltriazene 2a (56.7 mg, 0.3 mmol) and solvent (2.0 mL) was added and stirred at ambient temperature for 7 h. When the reaction was finished, 5 mL of water was added to the mixture and the aqueous mixture was extracted with ethyl acetate (3×5 mL). The combined organic phase was dried over anhydrous MgSO_4_, filtered and concentrated under reduced pressure, the residue was purified by silica gel column chromatography (eluent: petroleum ether/EtOAc = 40:1 to 10:1, v/v) to afford the desired product 3a as a yellow semisolid (24.9 mg, 37% yield). TLC showed that only trace amounts of product 3a were obtained when DCM was used as solvent. No corresponding product was obtained using DCE, toluene, acetonitrile and 1,4-dioxane as solvents (See Table S1).

##### Density functional theory (DFT) calculations

All calculations have been performed using the DFT method implemented in the commercial Gaussian 16 program package. Molecular geomtetries of the model complexes were optimized applying the M062X(D3) functional. For all the atoms the 6-31G∗ Pople basis set level with the SMD solvation model and 2,2,2-trifluoroethanol as the solvent was used. As soon as the convergences of optimizations were obtained, the frequency calculations ate the same level have been performed to identify all the stationary points as minima or transition states, which has the unique imaginary frequencies. And the intrinsic reaction coordinate (IRC) calculations have confirmed that all stationary points were smoothly connected to each other. All of the optimized geometries mentioned were built by Gaussview 6.0.^60^^,^^61^^,^^62^^,^^63^

### Quantification and statistical analysis

#### Statistical analysis

For Figure 1 Cytotoxicity was determined by the MTT reduction assay, and the absorbance of solutions was measured in Bio-Rad iMark microplate reader (540 nm and 690 nm). Triplicates were performed. Data analysis and plotting were operated by the GraphPad Prism 5.0 software , to calculate LC5.

## Supplemental information

Document S1. Figures S1–S35, Schemes S1–S3, and Table S1

Table S2. Standard orientation of computational structures

Data S1. Analytical data

Data S2. Copies of NMR spectra
