# Supplementary material for: Metal-free molecular editing of indole via tandem reaction: Access to 2-aryl-3-aryldiazenylindole for theranostic applications
Source: iScience. 2025 Aug 8;28(9):113325. doi: 10.1016/j.isci.2025.113325 (PMC12496193; doi:10.1016/j.isci.2025.113325)

iScience, Volume *28*

Supplemental information

Metal-free molecular editing of indole via tandem reaction: Access to 2-aryl-3-aryldiazenylindole for theranostic applications

Lin Zhang, Yonghong Liu, Rui Chen, Bin Wang, Azhar Iqbal, Weiwei Jin, Yu Xia, Shaofeng Wu, Ziren Chen, Penji Yan, Chenjiang Liu, and Yonghong Zhang

Supplemental Figures and Legends

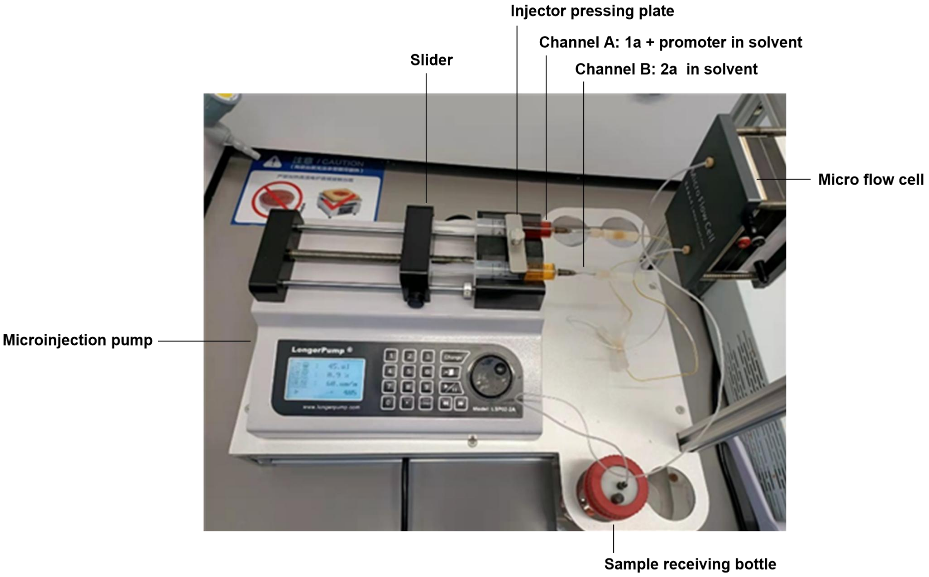

Fig. S1 Continuous flow system set-up

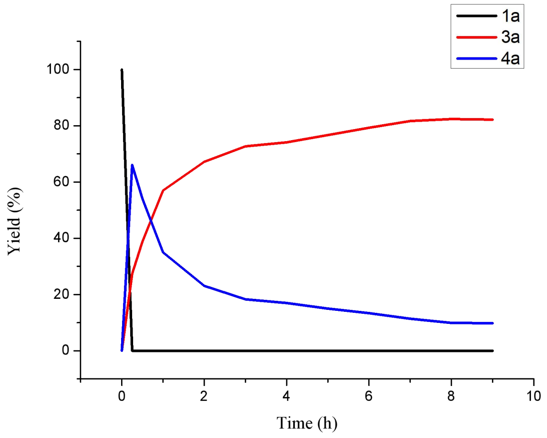

Fig. S2 The conversion rate of **1a**, **3a** and **4a** versus time

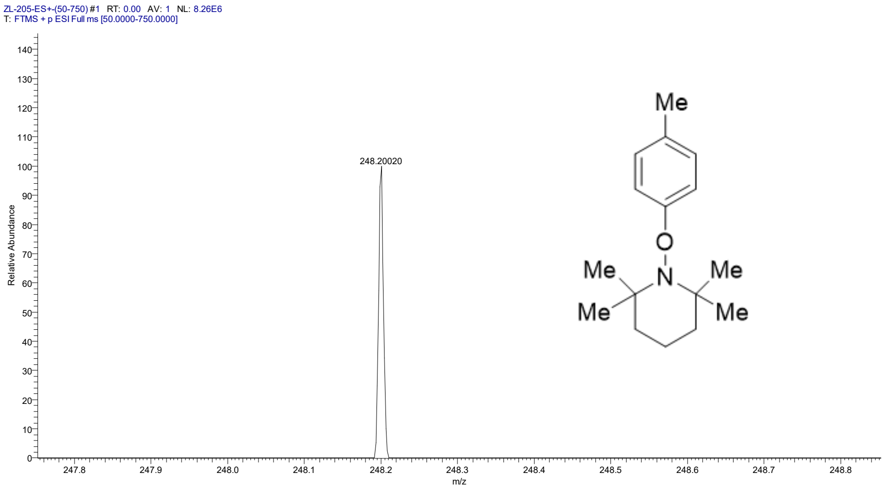

Fig. S3 HRMS spectra of the aryl radical-TEMPO adduct

Supplemental Schemes and Legends

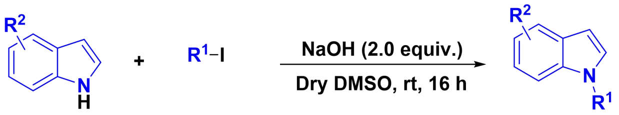

Scheme S1 Synthesis of *N*-Protected Indoles

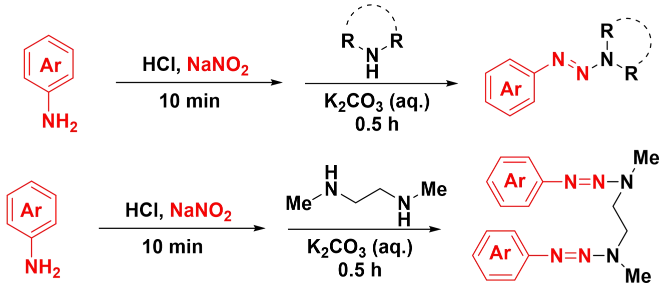

Scheme S2 Synthesis of Aryltriazenes

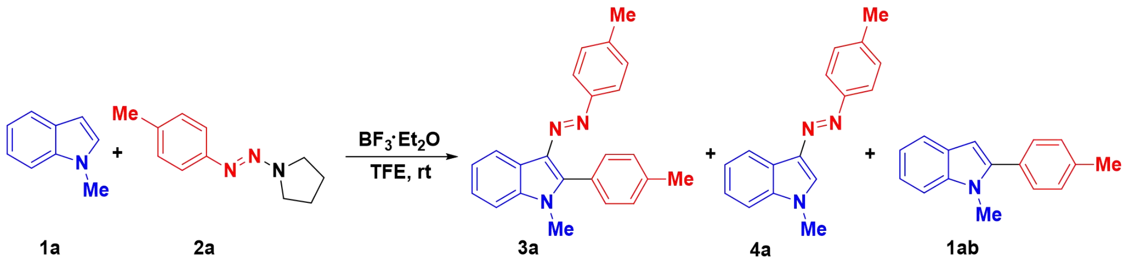

Scheme S3 The conversion rate of **1a**, **3a** and **4a** versus time

Supplemental Table

Table S1 C2 arylation of **4a** with **2a** under promoter-free condtion

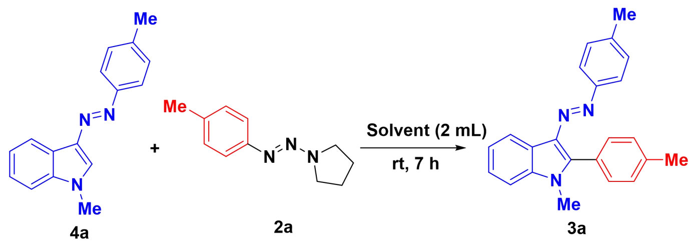

Entry	Solvent	**3a**
1	TFE	37
2	DCM	trace
3	DCE	N.R.
4	Toluene	N.R.
5	1,4-dioxane	N.R.
6	CH_3CN	N.R.

Fig. S4A: ^{1}H NMR of product **3a** in $CDCl_3$ (600 MHz)

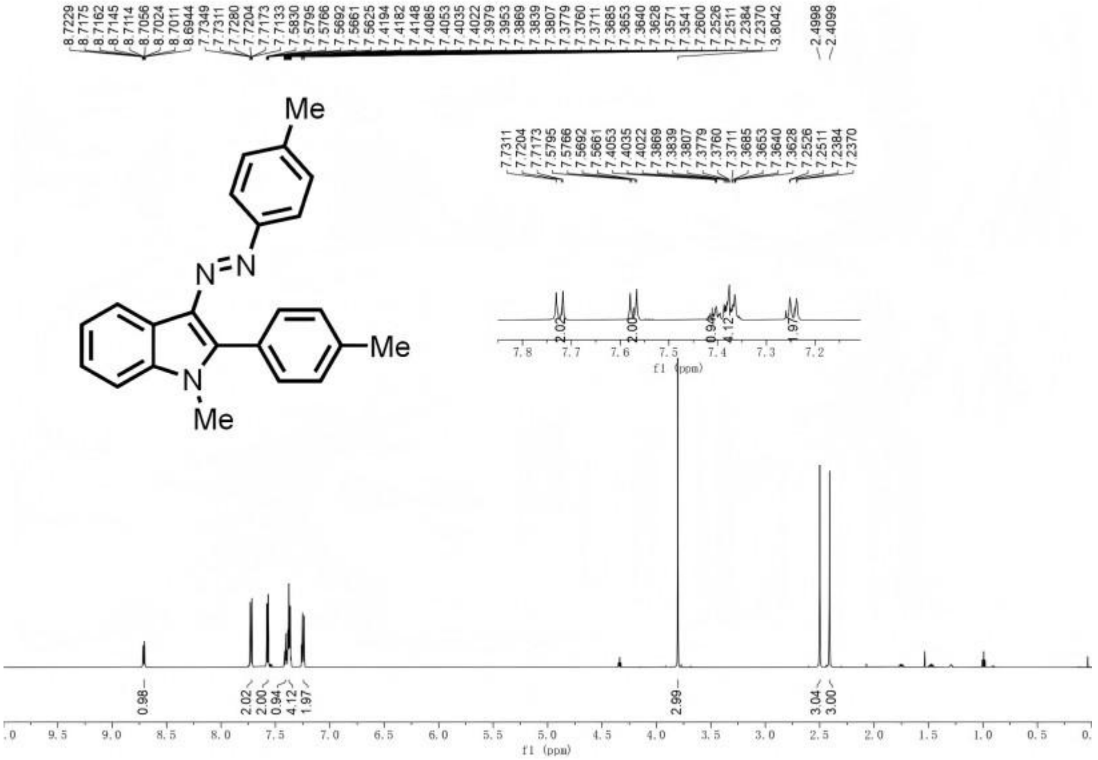

Fig. S4B: ^{13}C NMR of product **3a** in $CDCl_3$ (151 MHz)

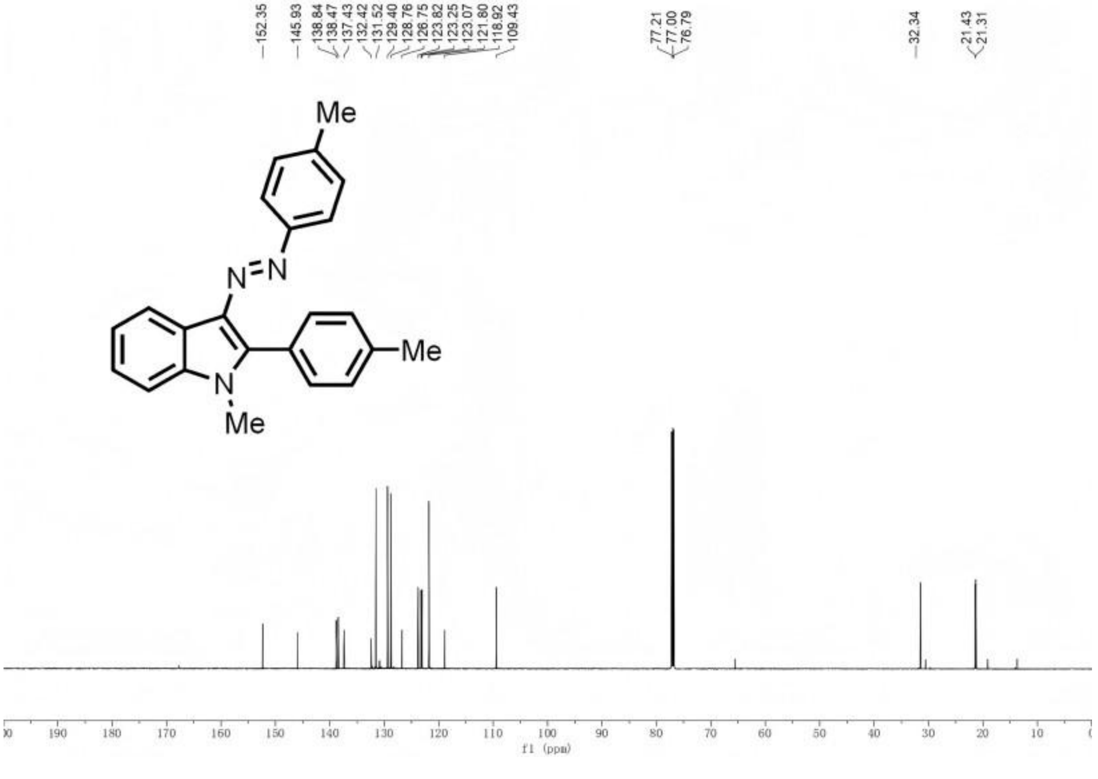

Fig. S5A: ^{1}H NMR of product **3b** in $CDCl_3$ (600 MHz)

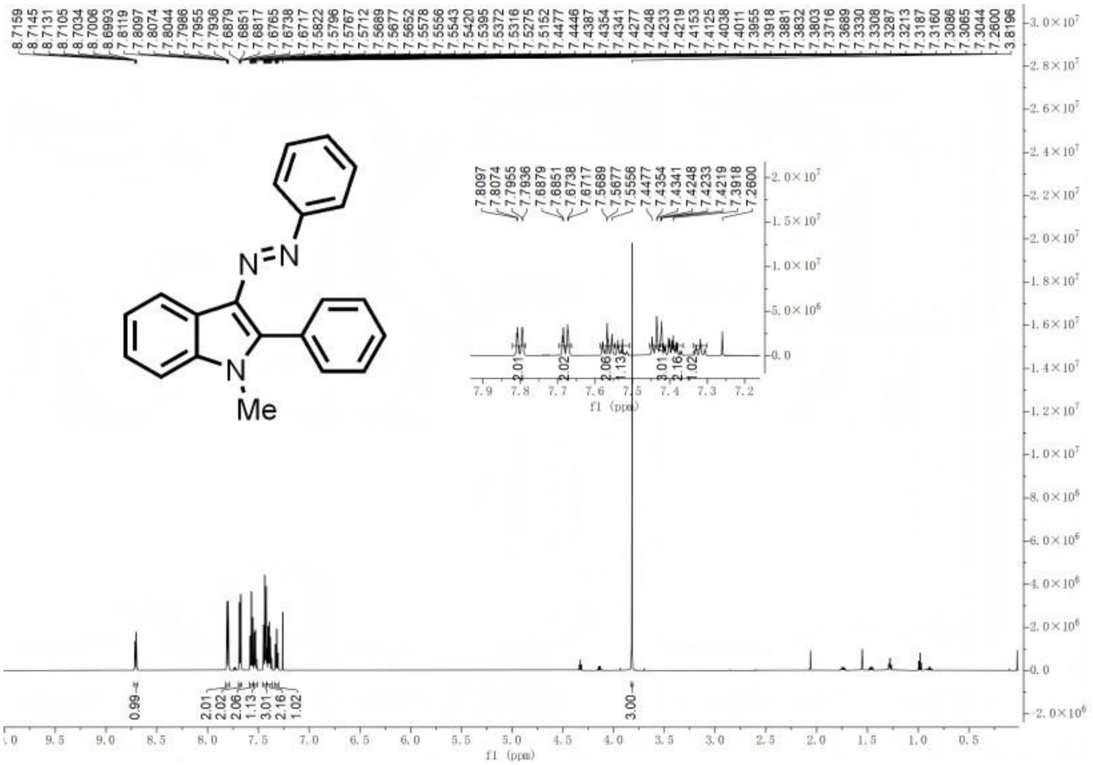

Fig. S5B: ^{13}C NMR of product **3b** in $CDCl_3$ (151 MHz)

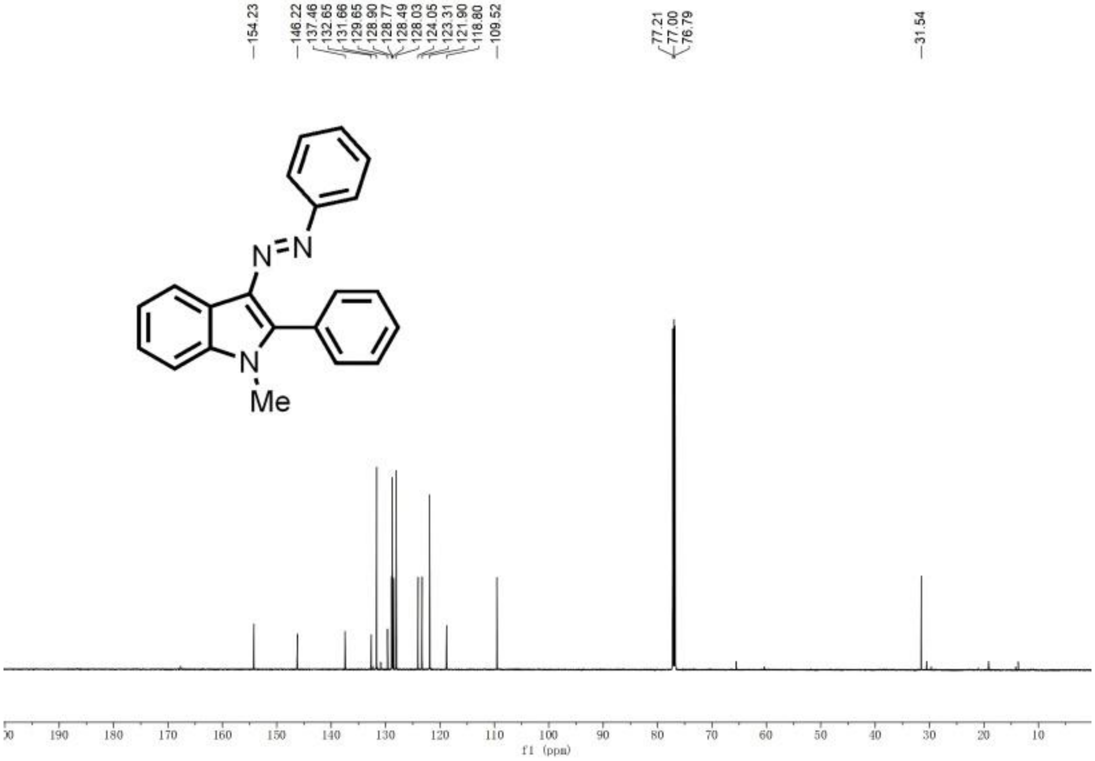

Fig. S6A: ^{1}H NMR of product **3c** in $CDCl_3$ (600 MHz)

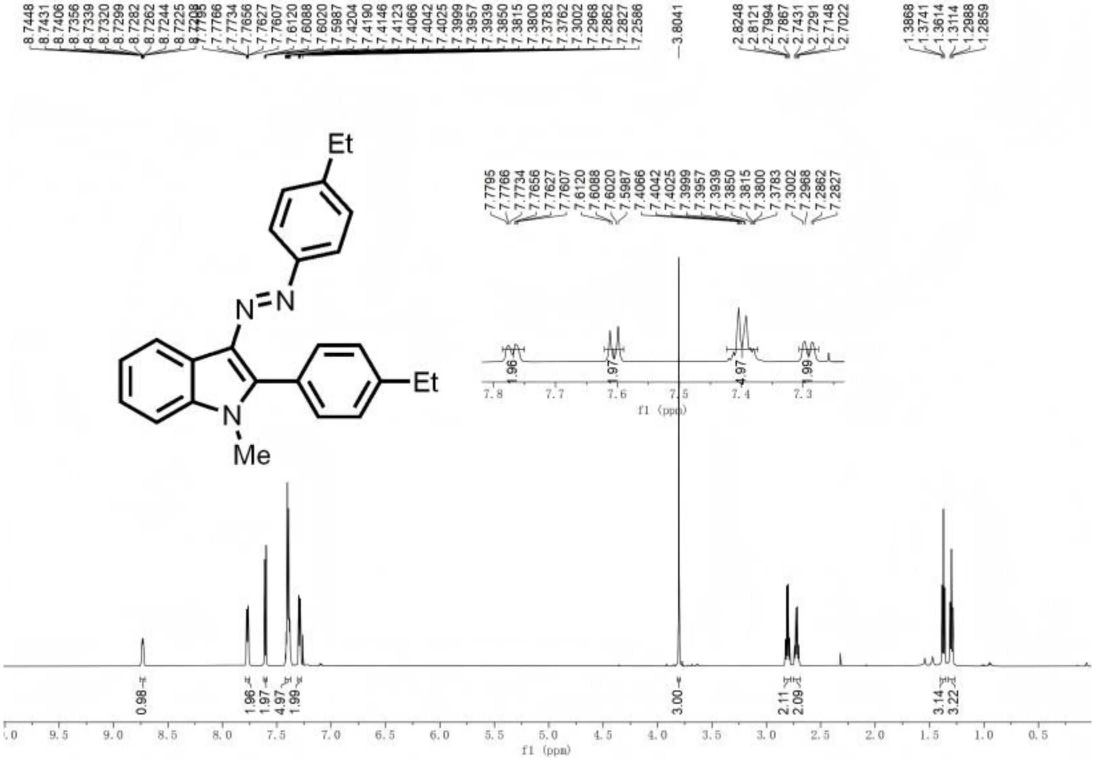

Fig. S6B: ^{13}C NMR of product **3c** in $CDCl_3$ (151 MHz)

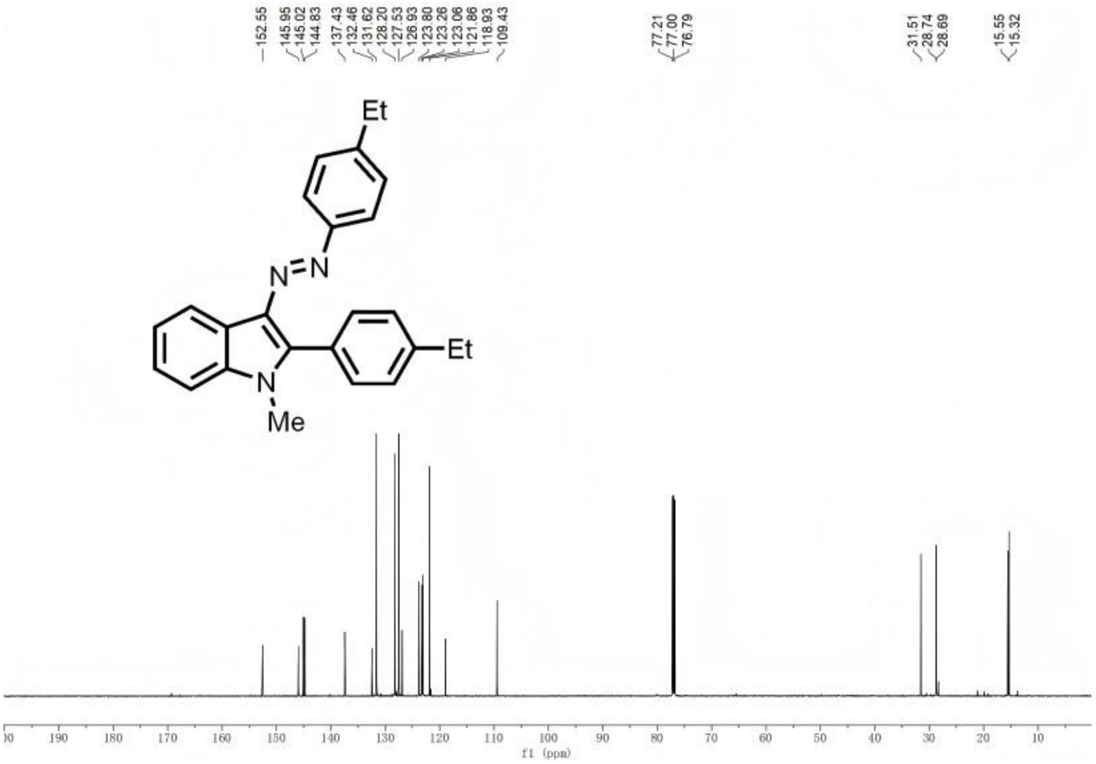

Fig. S7A: ^{1}H NMR of product **3d** in $CDCl_3$ (600 MHz)

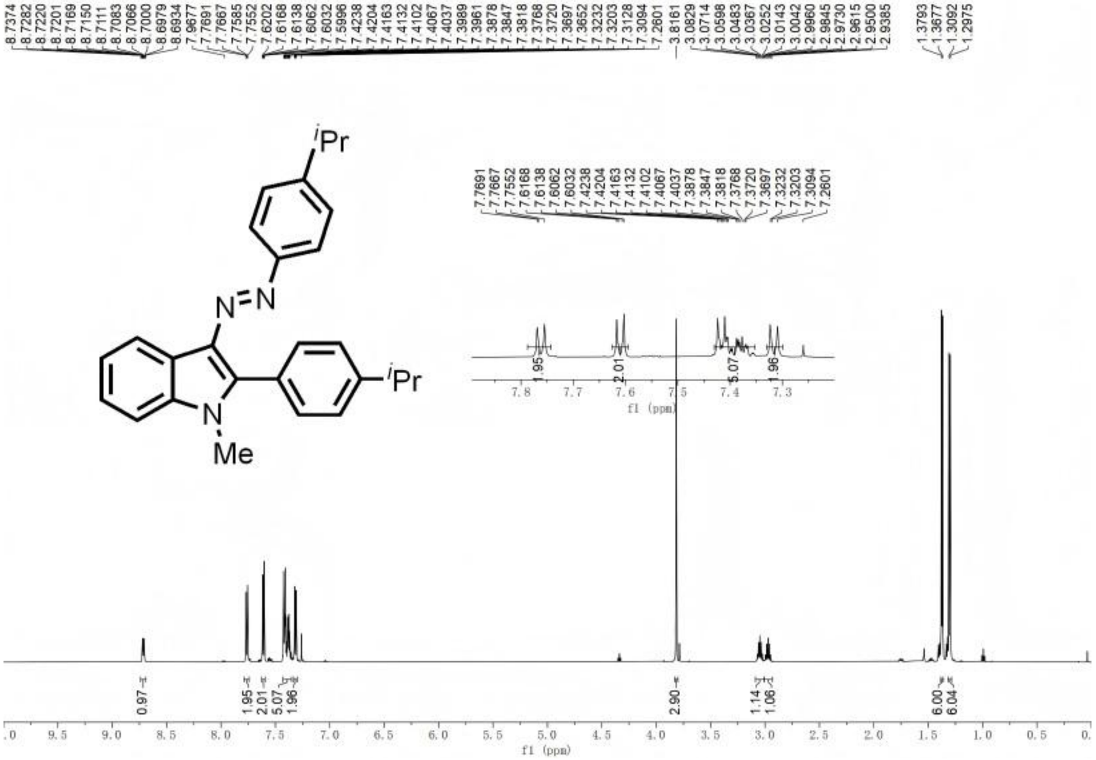

Fig. S7B: ^{13}C NMR of product **3d** in $CDCl_3$ (151 MHz)

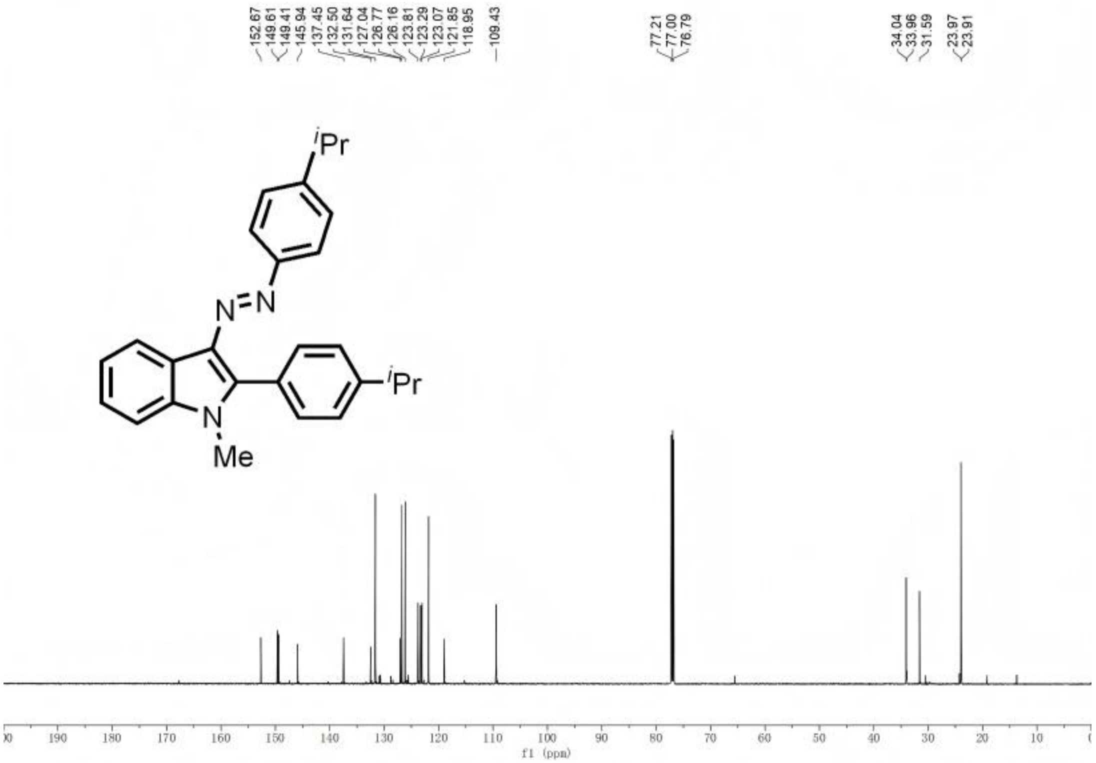

Fig. S8A: ^{1}H NMR of product **3e** in $CDCl_3$ (600 MHz)

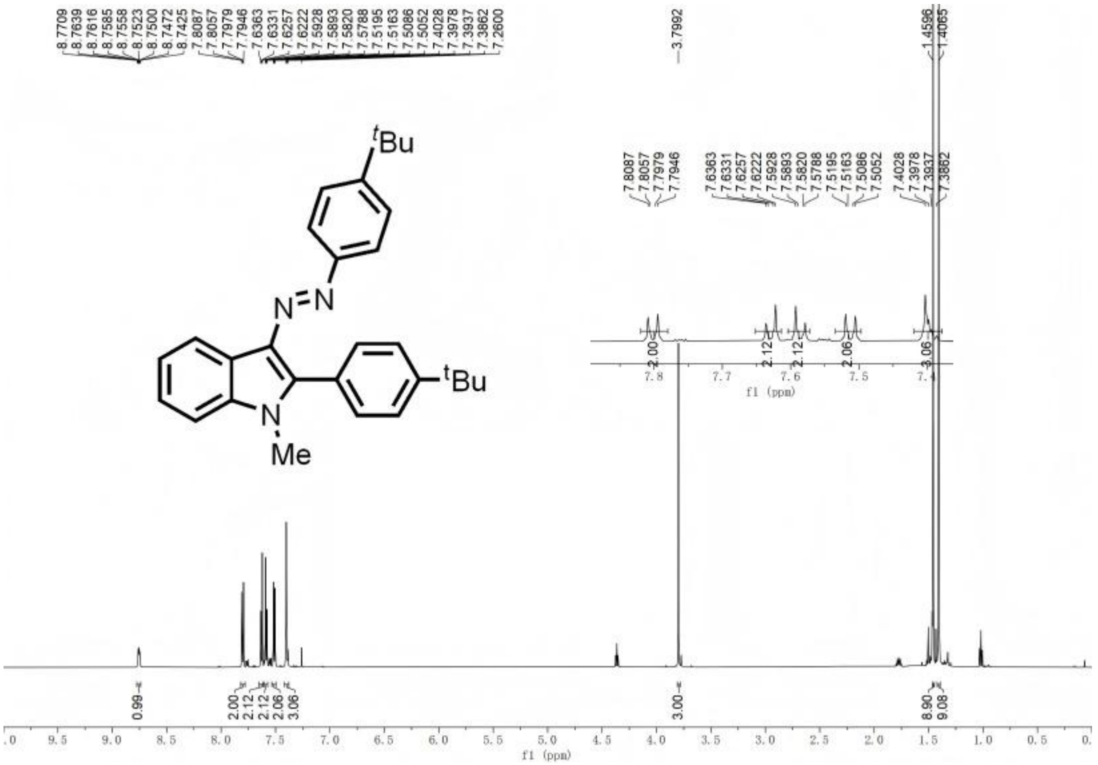

Fig. S8B: ^{13}C NMR of product **3e** in $CDCl_3$ (151 MHz)

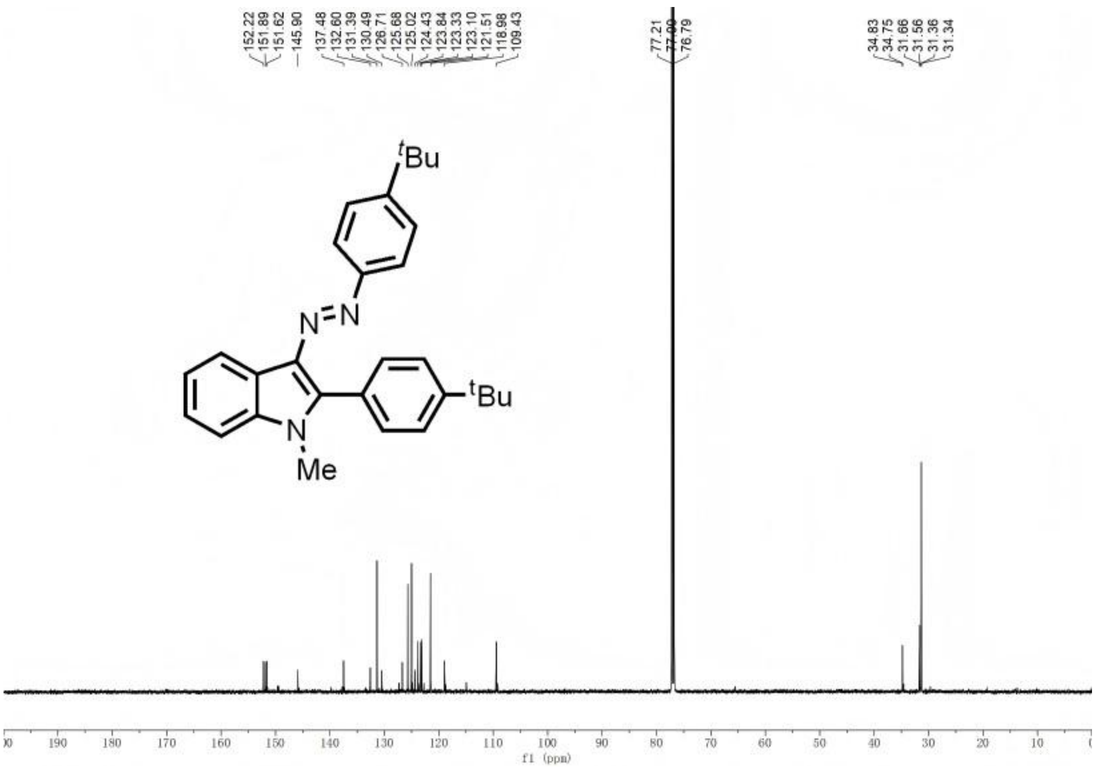

Fig. S9A: ^{1}H NMR of product **3f** in $CDCl_3$ (600 MHz)

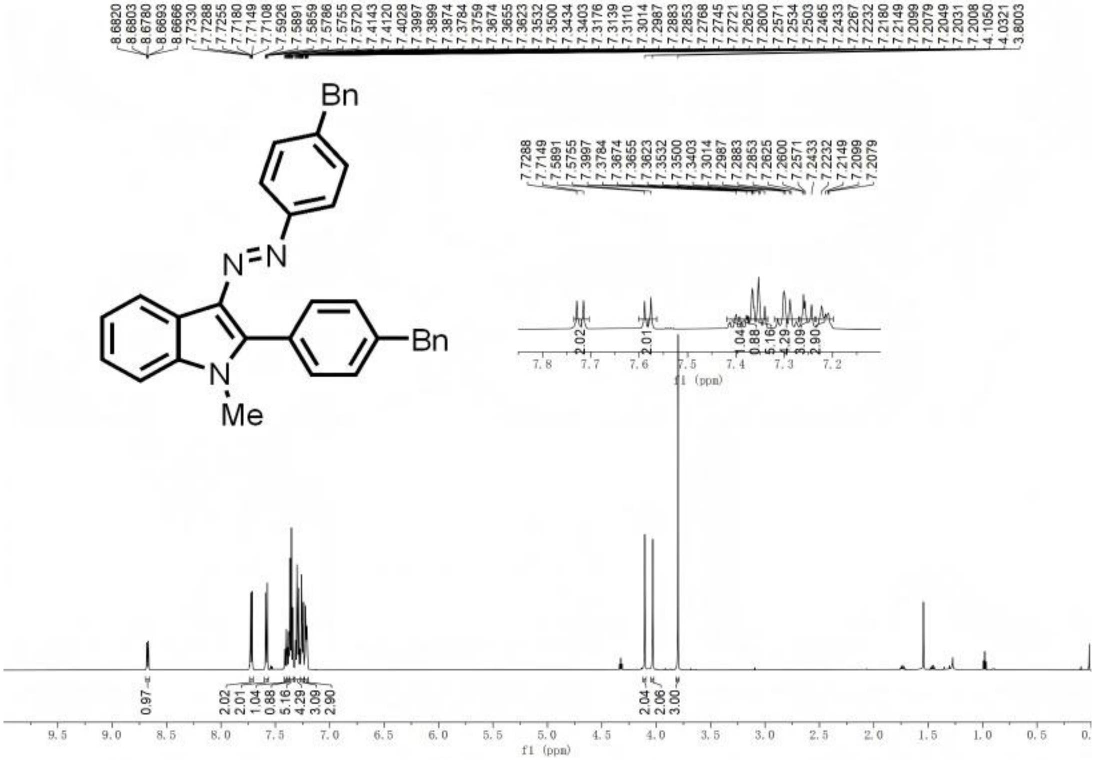

Fig. S9B: ^{13}C NMR of product **3f** in $CDCl_3$ (151 MHz)

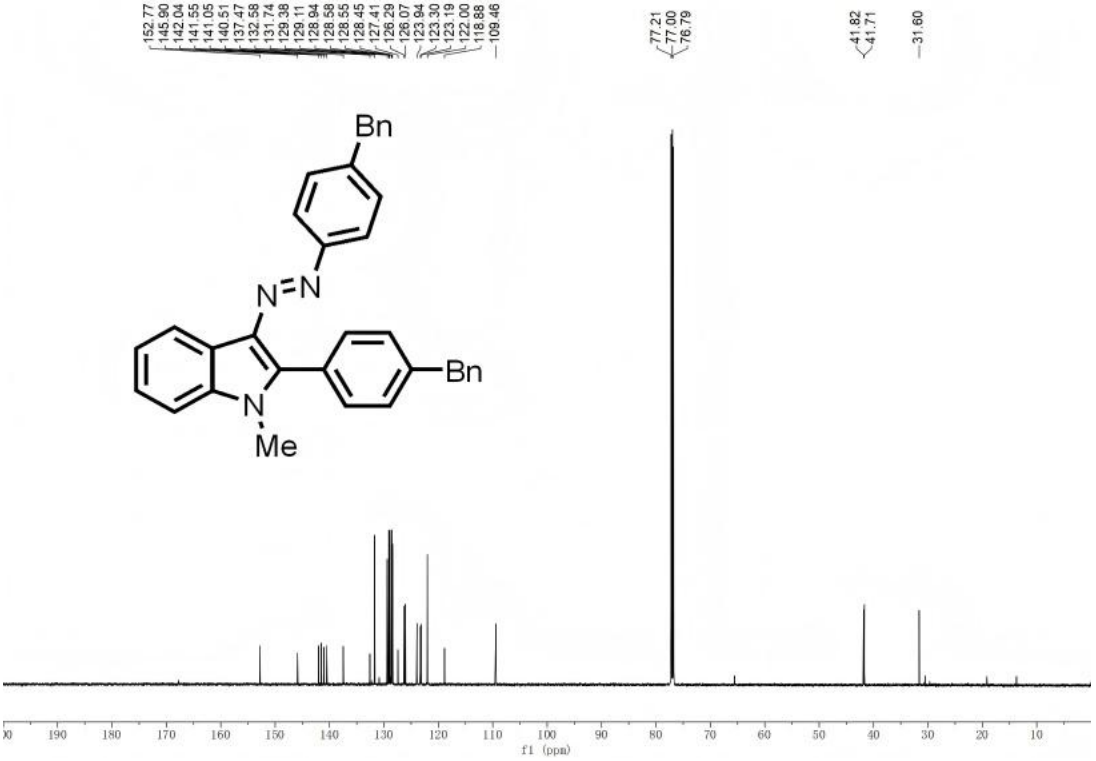

Fig. S10A: ^{1}H NMR of product **3g** in $CDCl_3$ (400 MHz)

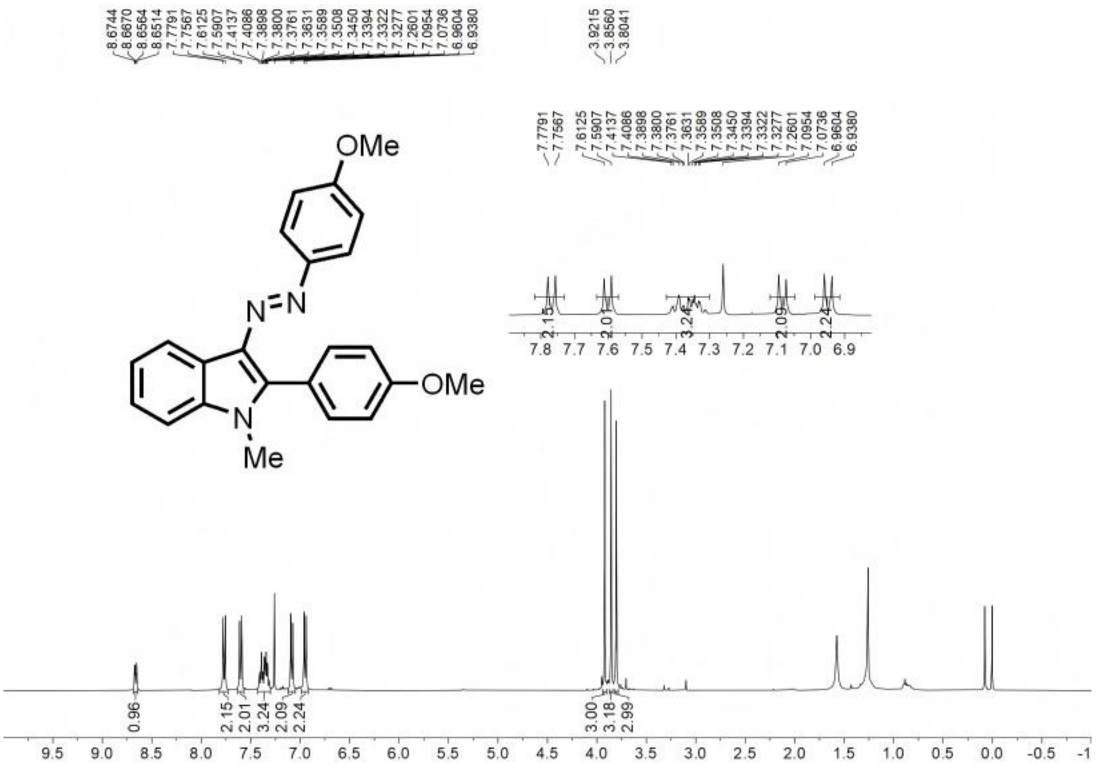

Fig. S10B: ^{13}C NMR of product **3g** in $CDCl_3$ (101 MHz)

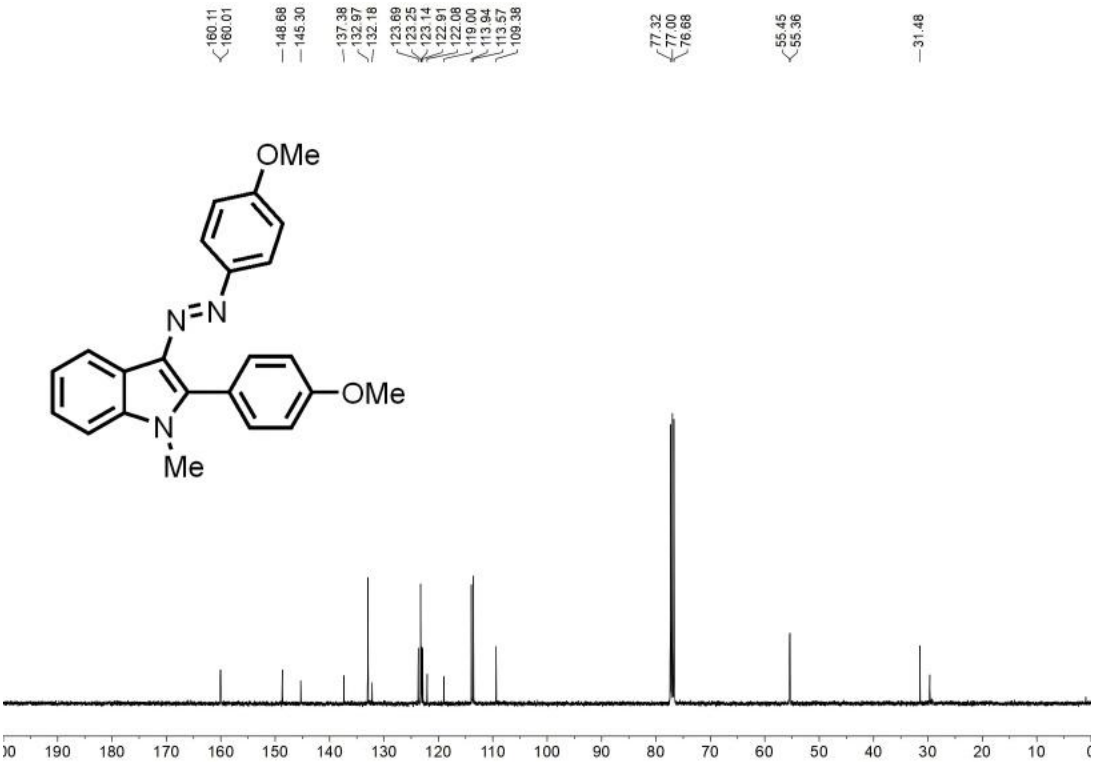

Fig. S11A: ^{1}H NMR of product **3h** in $CDCl_3$ (400 MHz)

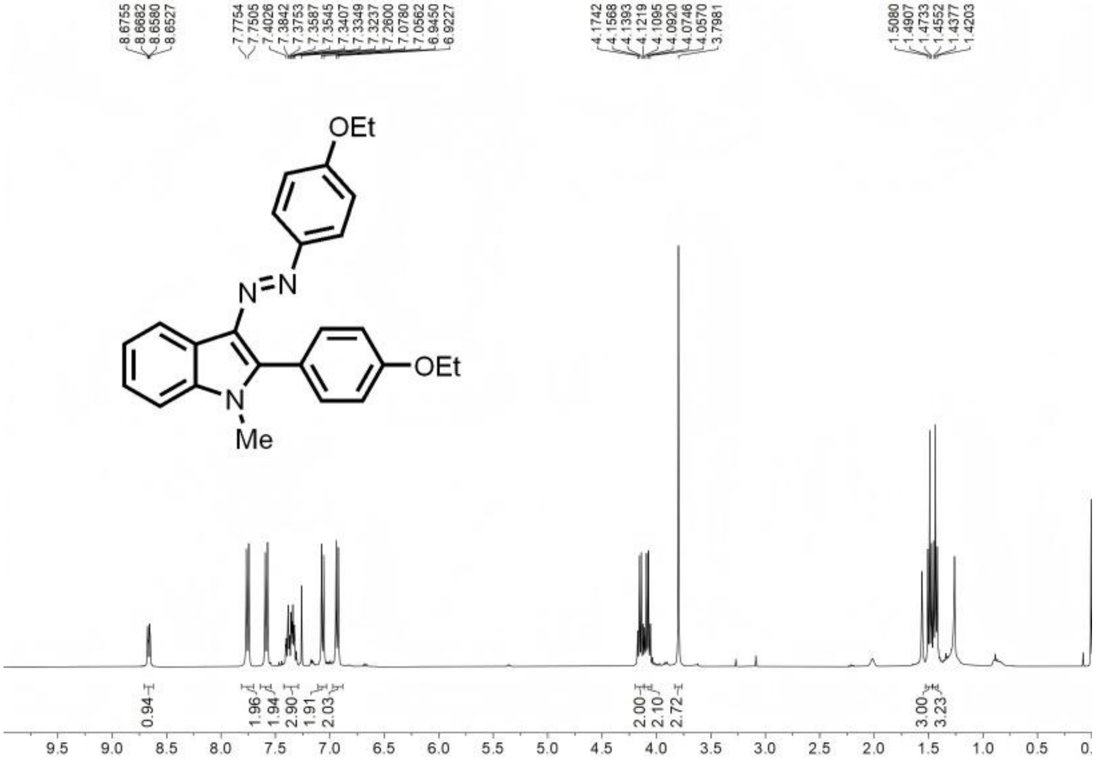

Fig. S11B: ^{13}C NMR of product **3h** in $CDCl_3$ (101 MHz)

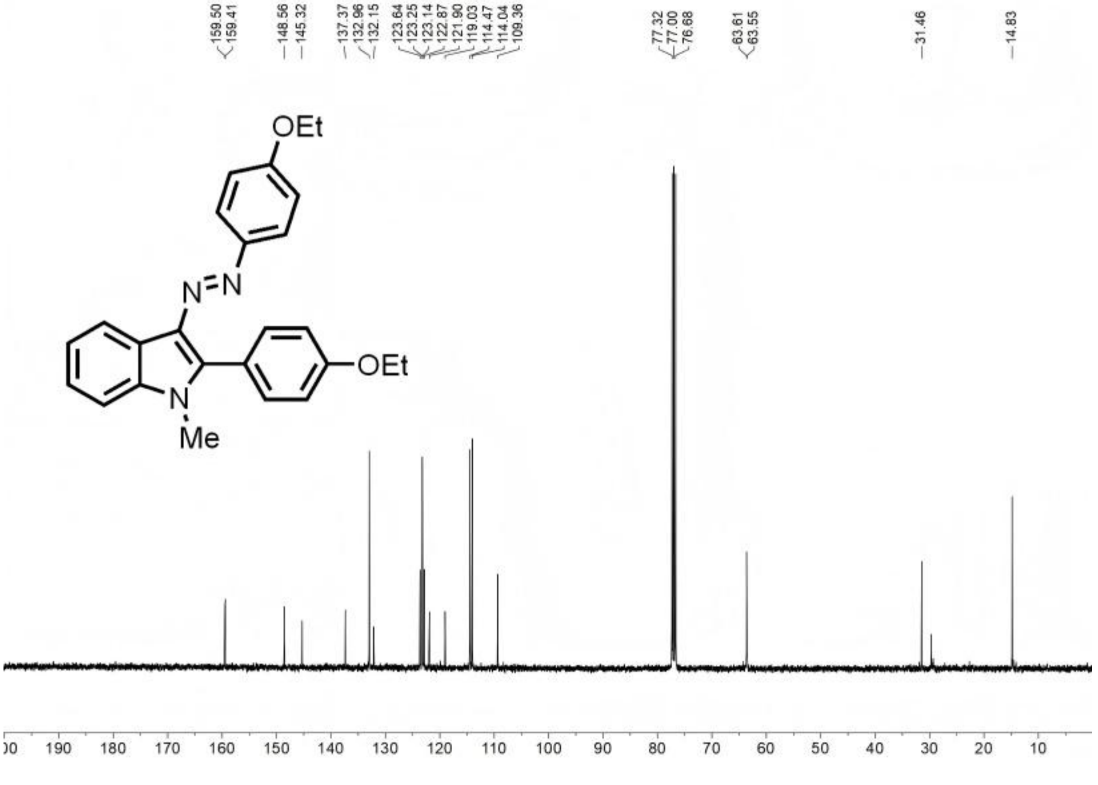

Fig. S12A: ^{1}H NMR of product **3i** in $CDCl_3$ (400 MHz)

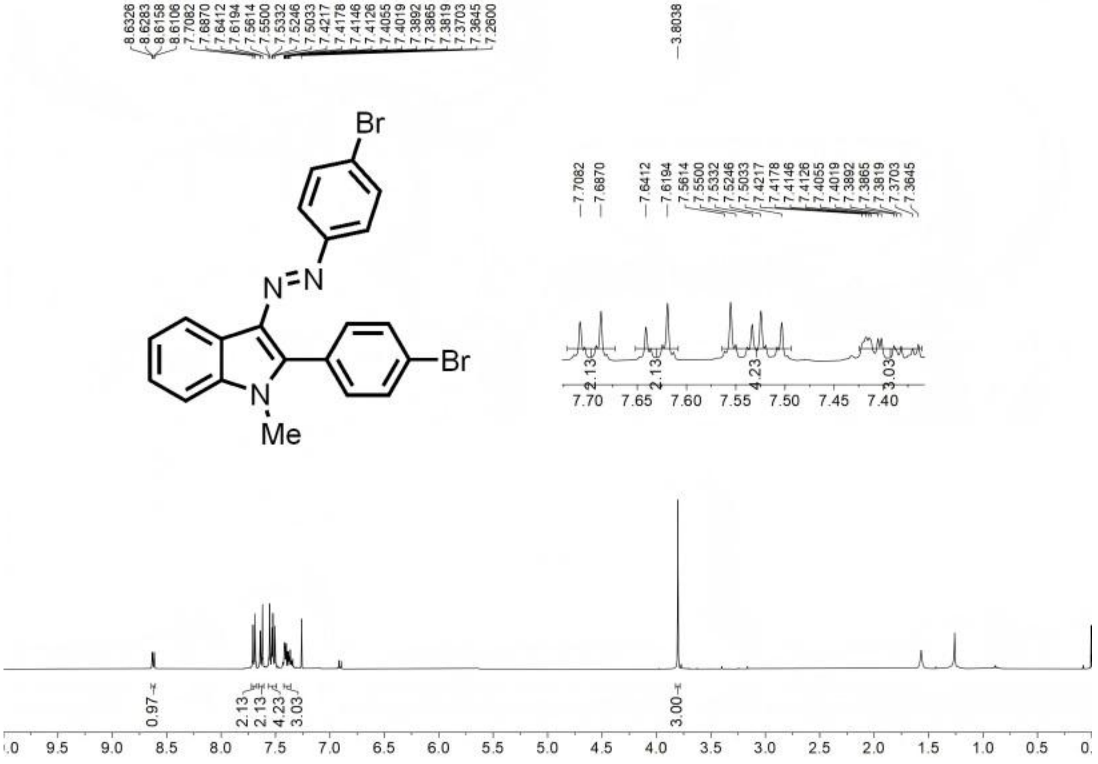

Fig. S12B: ^{13}C NMR of product **3i** in $CDCl_3$ (101 MHz)

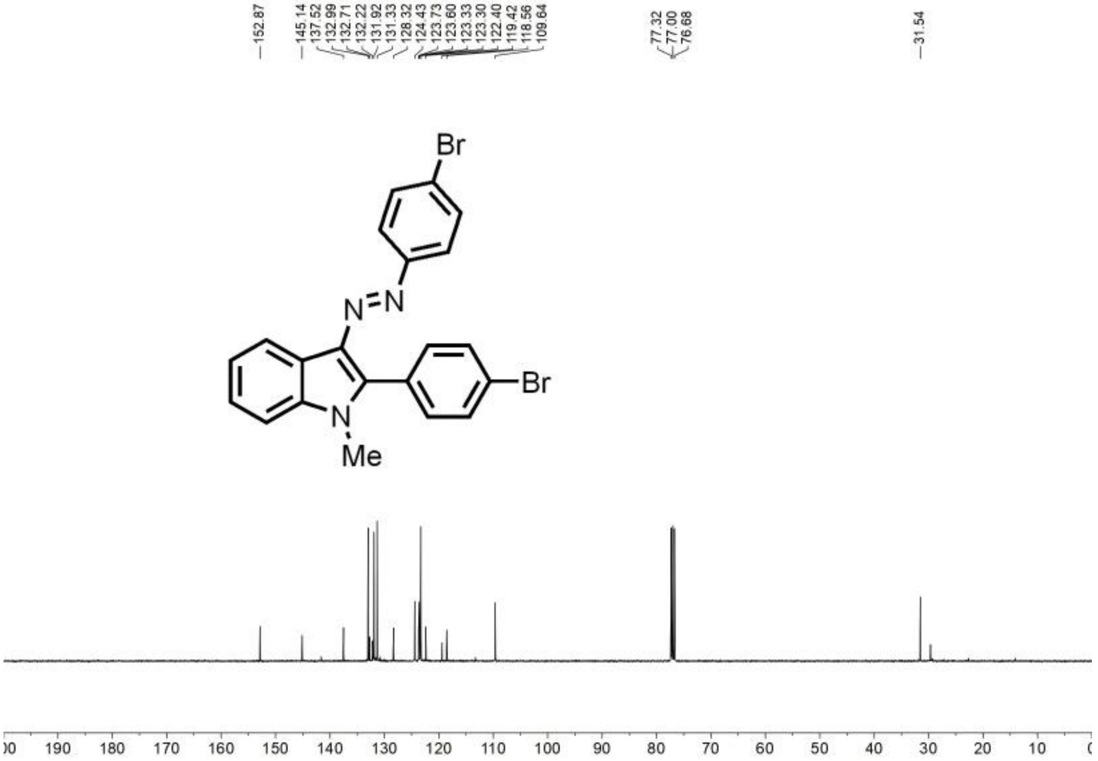

Fig. S13A: ^{1}H NMR of product **3j** in $CDCl_3$ (600 MHz)

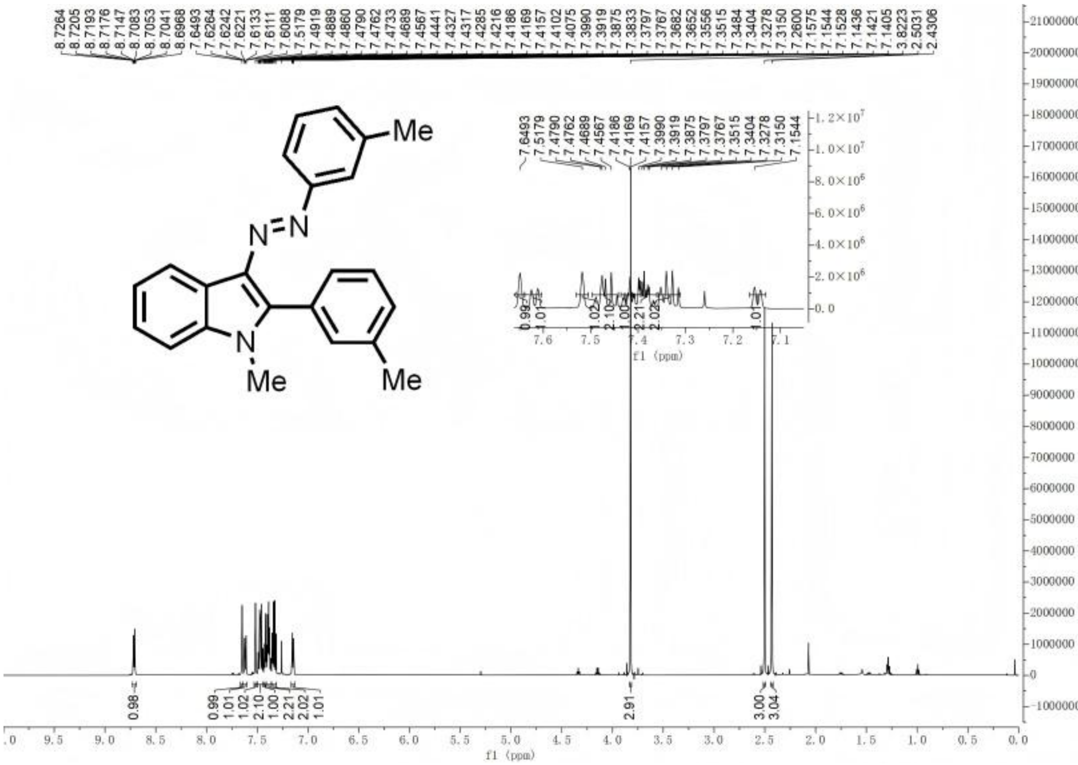

Fig. S13B: ^{13}C NMR of product **3j** in $CDCl_3$ (151 MHz)

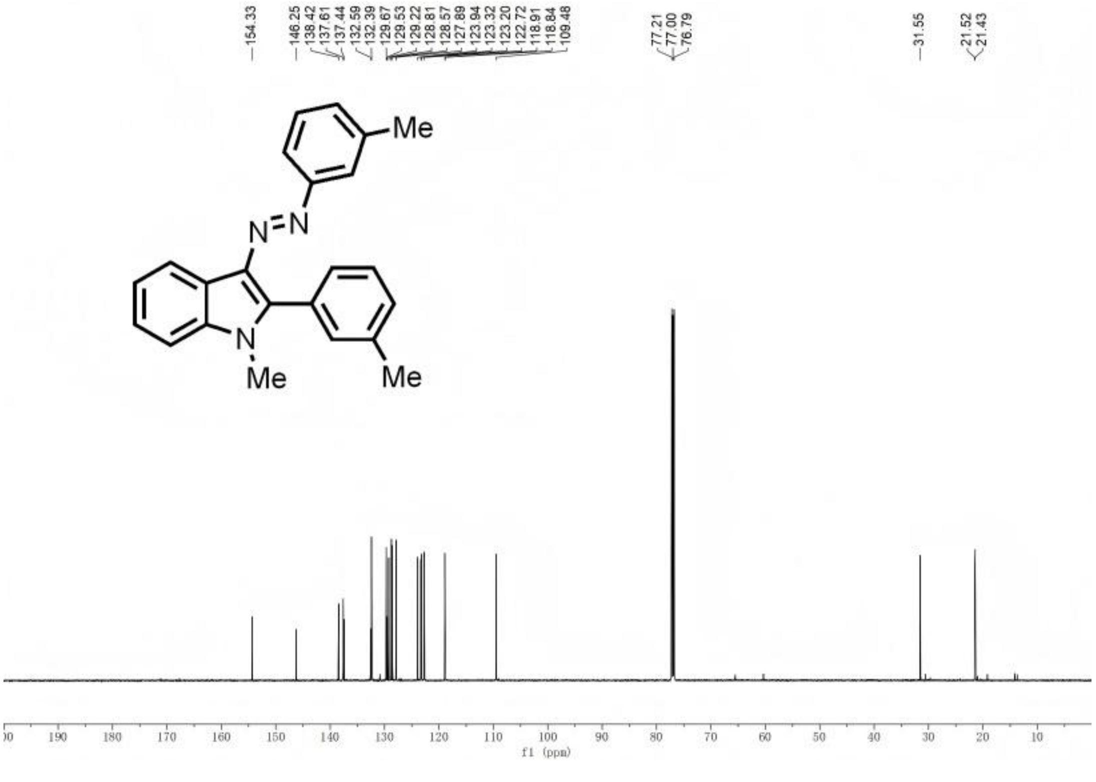

Fig. S14A: ^{1}H NMR of product **3k** in $CDCl_3$ (400 MHz)

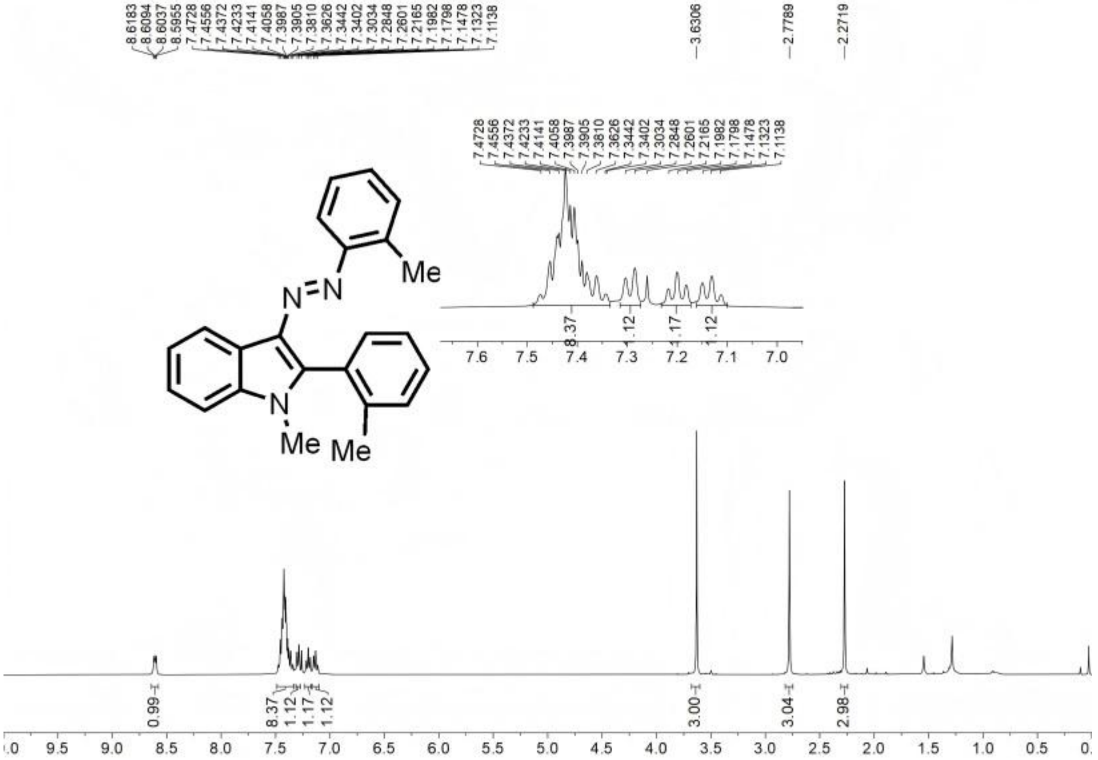

Fig. S14B: ^{13}C NMR of product **3k** in $CDCl_3$ (101 MHz)

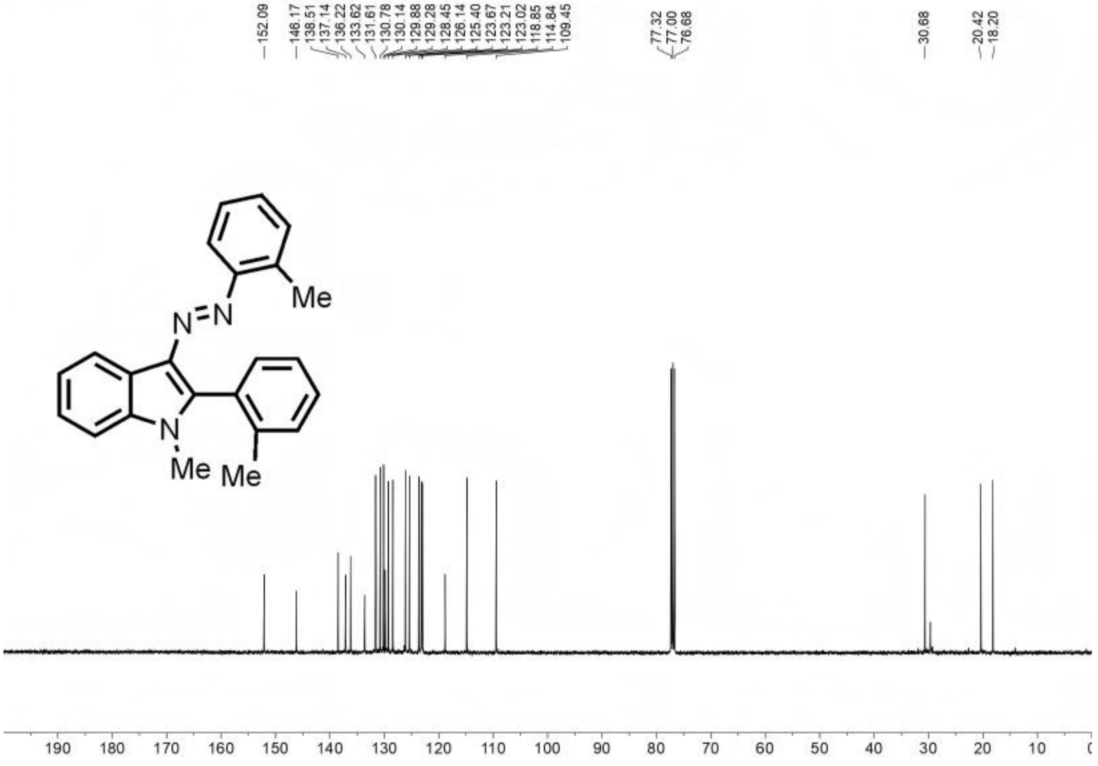

Fig. S15A: ^{1}H NMR of product **3l** in $CDCl_3$ (400 MHz)

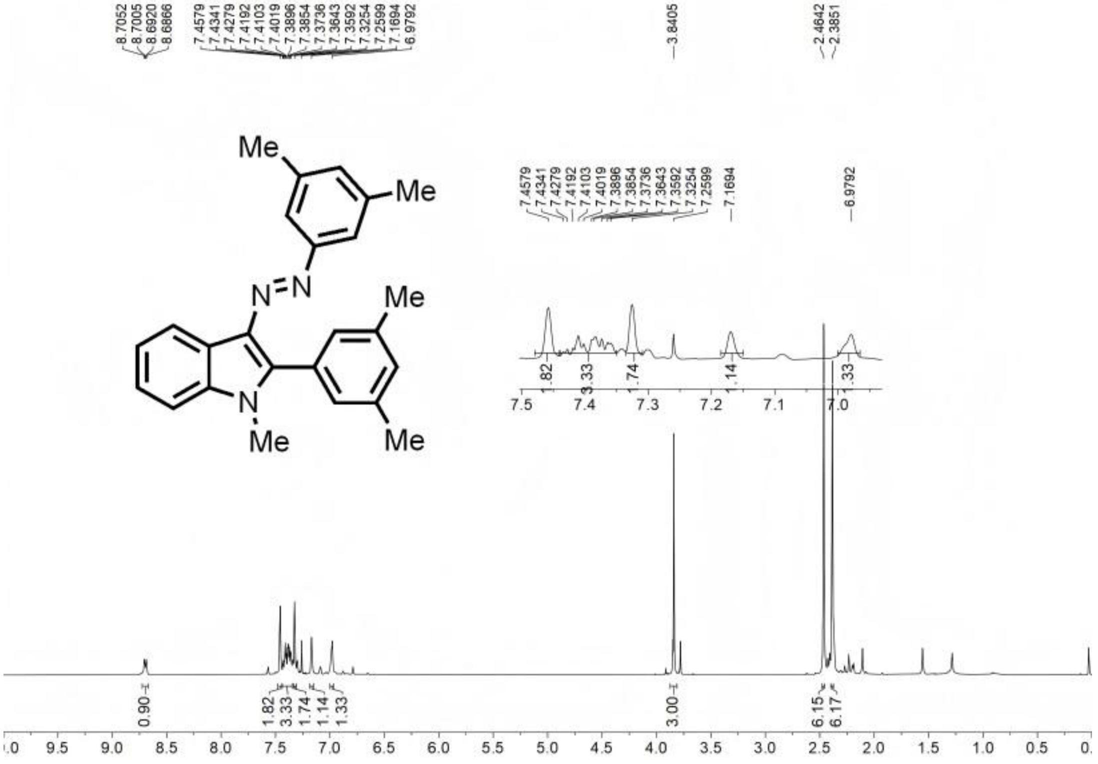

Fig. S15B: ^{13}C NMR of product **3l** in $CDCl_3$ (101 MHz)

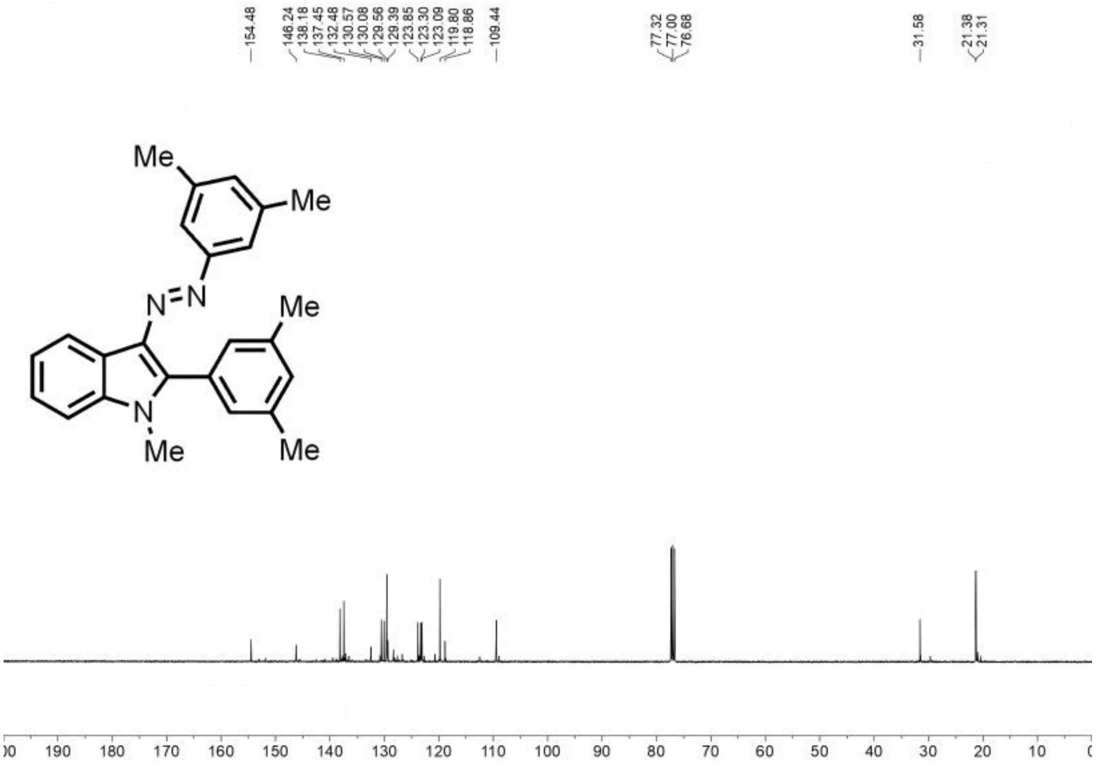

Fig. S16A: ^{1}H NMR of product **3m** in $CDCl_3$ (400 MHz)

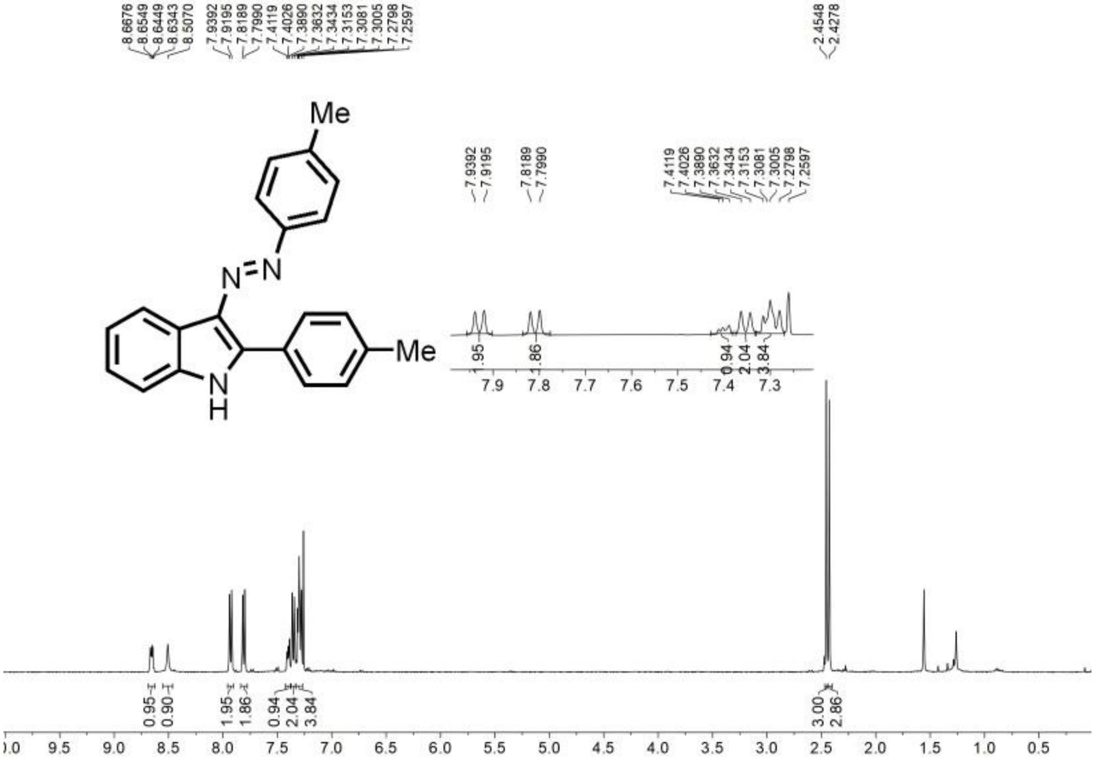

Fig. S16B: ^{13}C NMR of product **3m** in $CDCl_3$ (101 MHz)

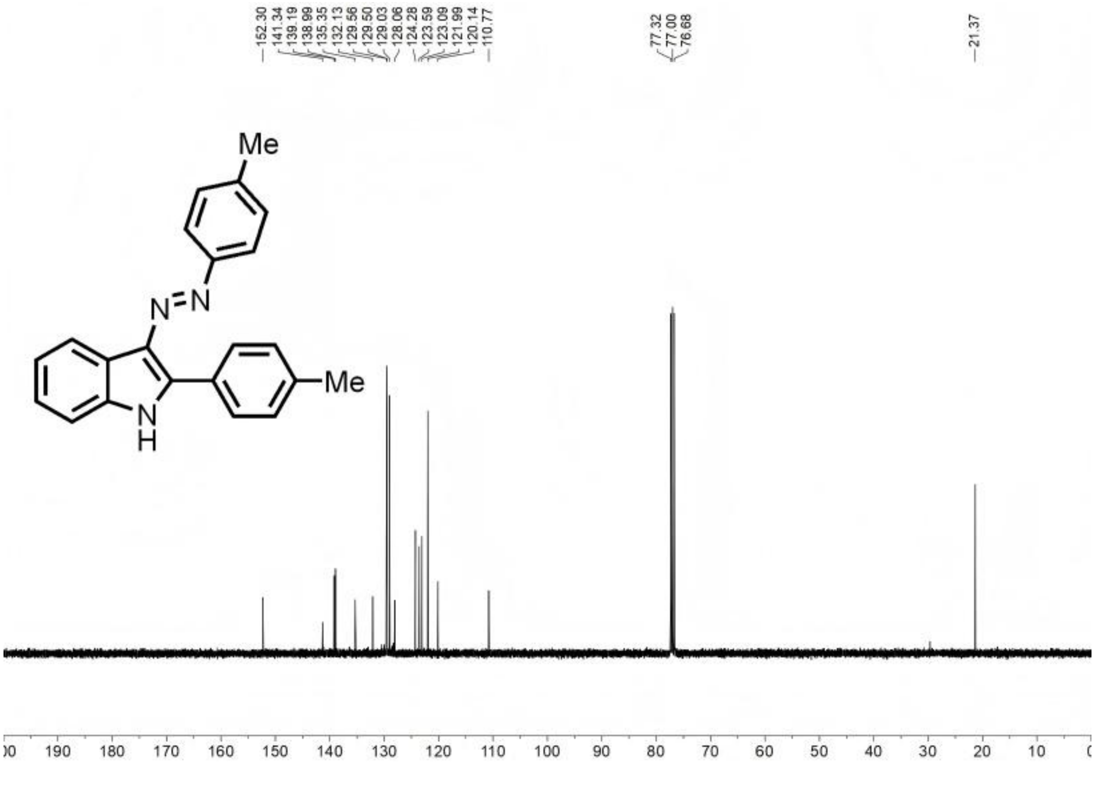

Fig. S17A: ^{1}H NMR of product **3n** in $CDCl_3$ (600 MHz)

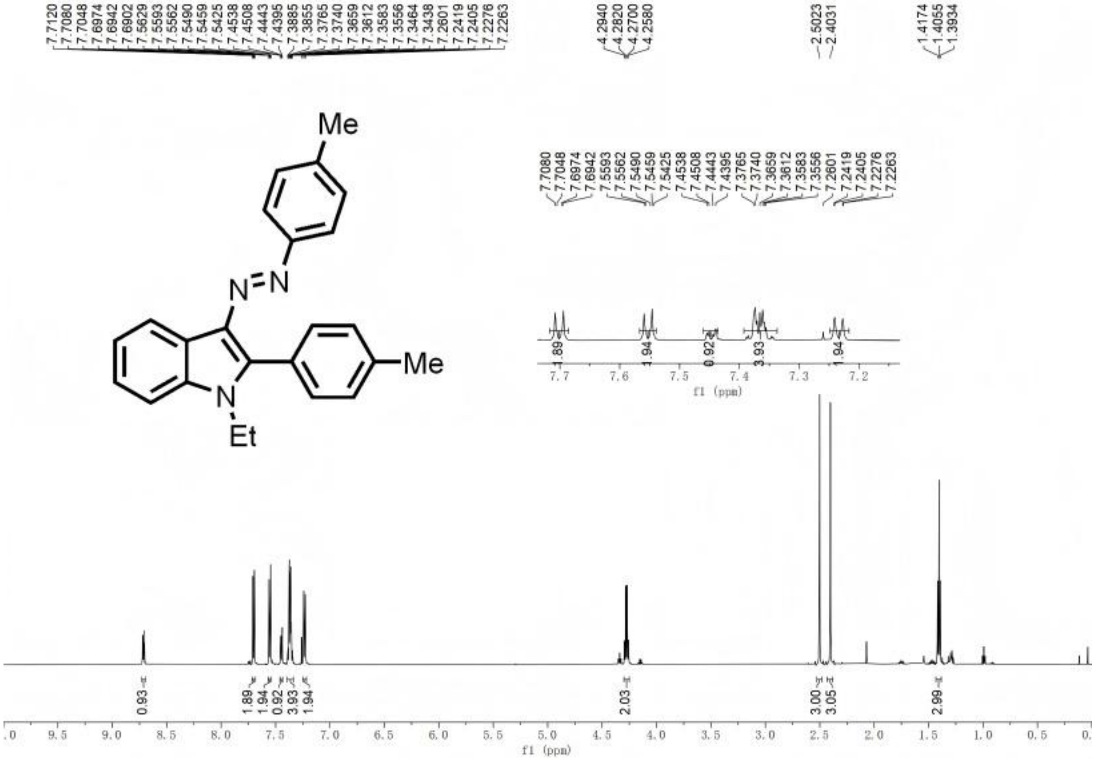

Fig. S17B: ^{13}C NMR of product **3n** in $CDCl_3$ (151 MHz)

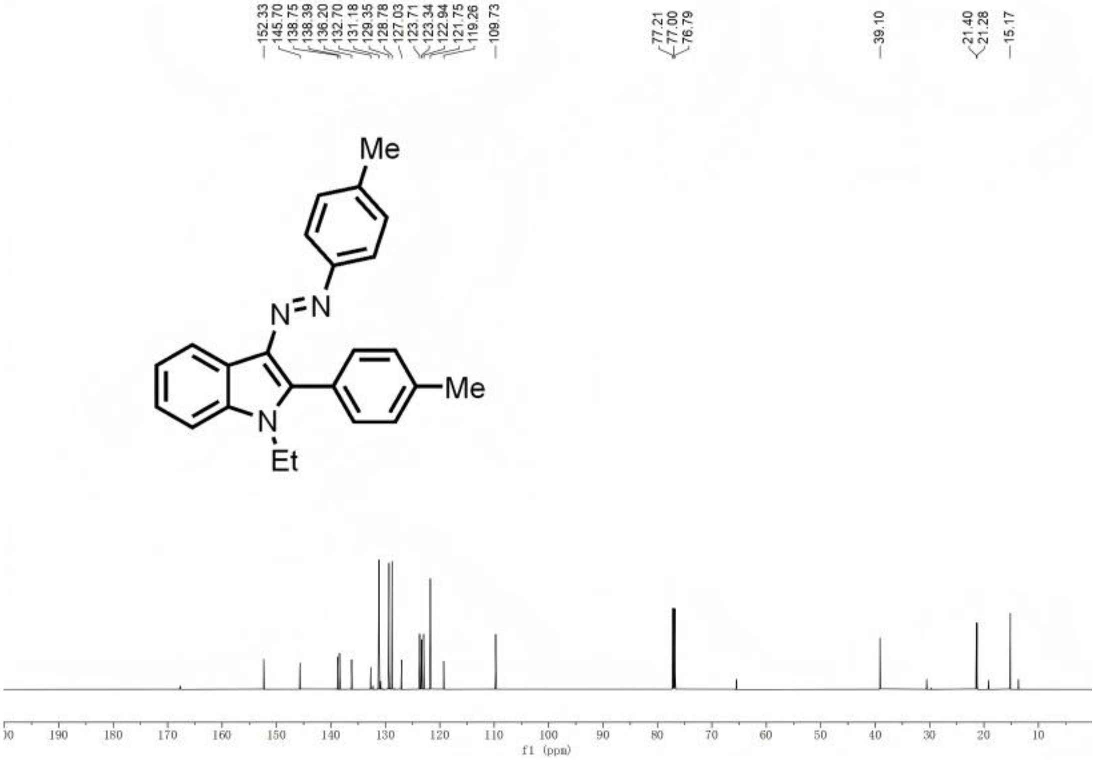

Fig. S18A: ^{1}H NMR of product **3o** in $CDCl_3$ (600 MHz)

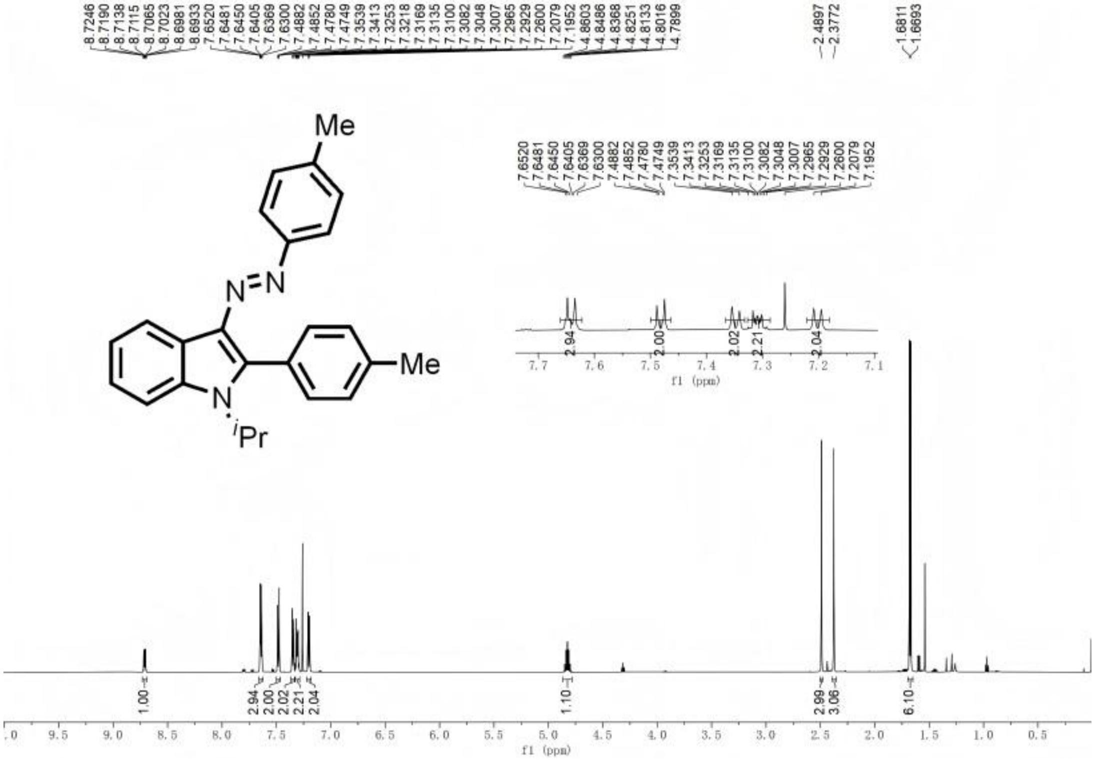

Fig. S18B: ^{13}C NMR of product **3o** in $CDCl_3$ (151 MHz)

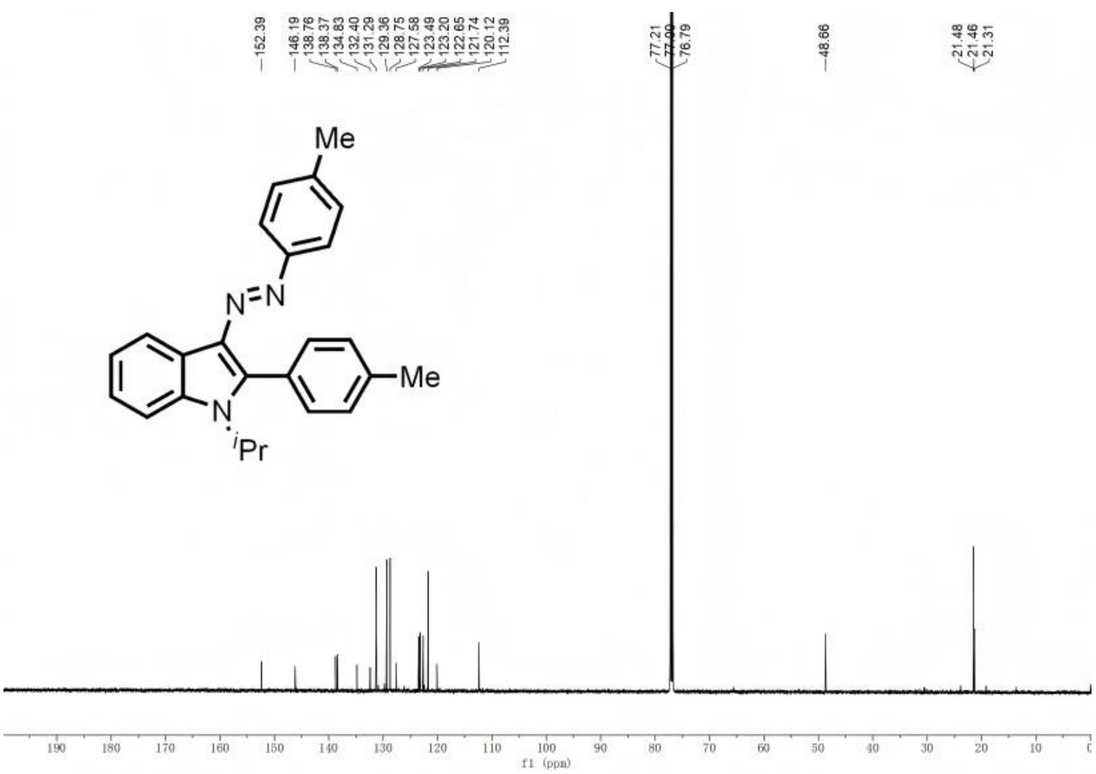

Fig. S19A: ^{1}H NMR of product **3p** in $CDCl_3$ (600 MHz)

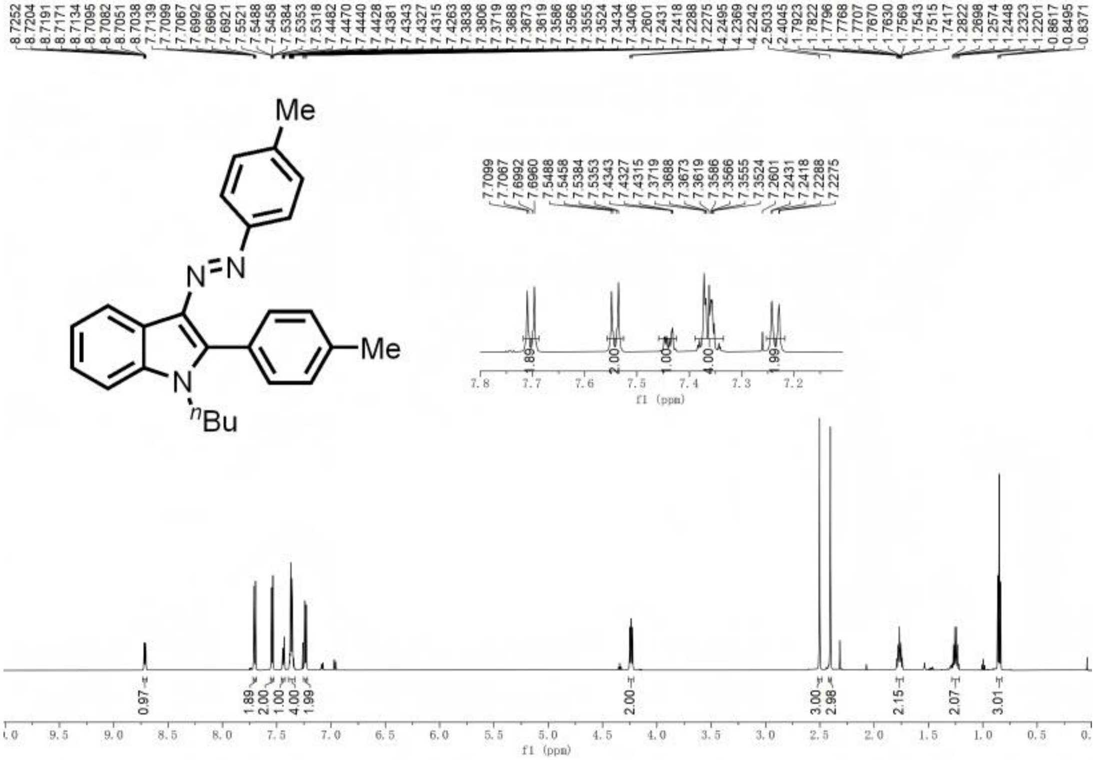

Fig. S19B: ^{13}C NMR of product **3p** in $CDCl_3$ (151 MHz)

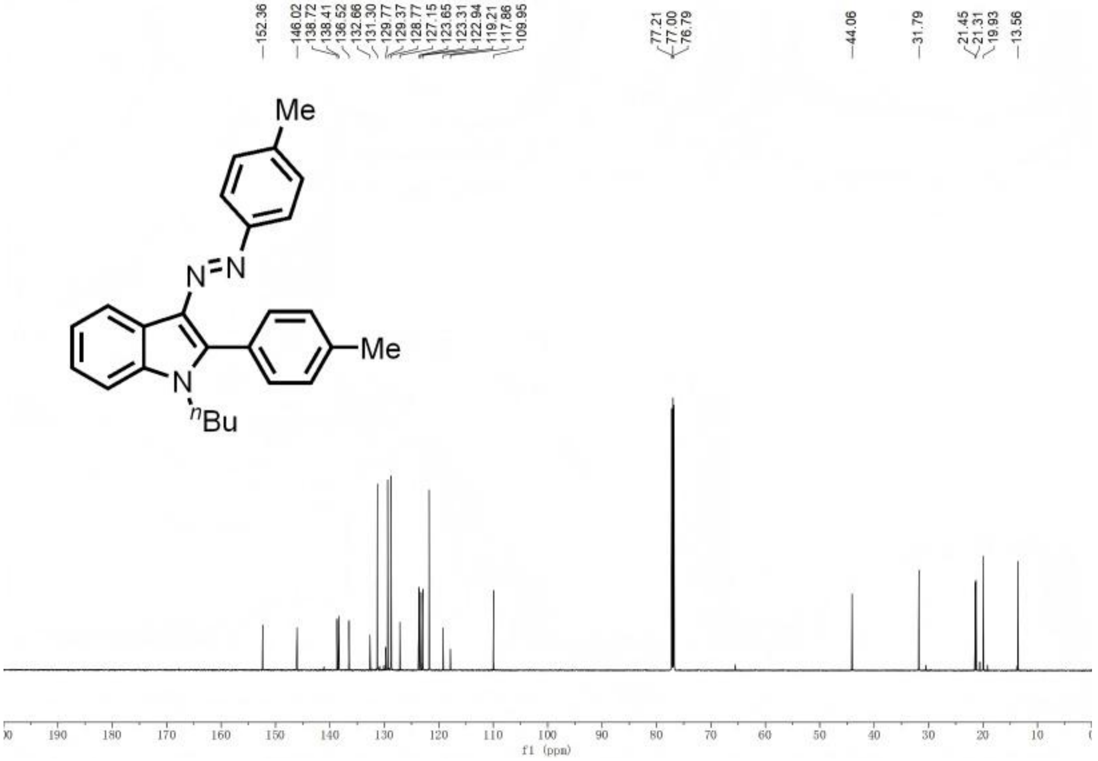

Fig. S20A: ^{1}H NMR of product **3r** in $CDCl_3$ (600 MHz)

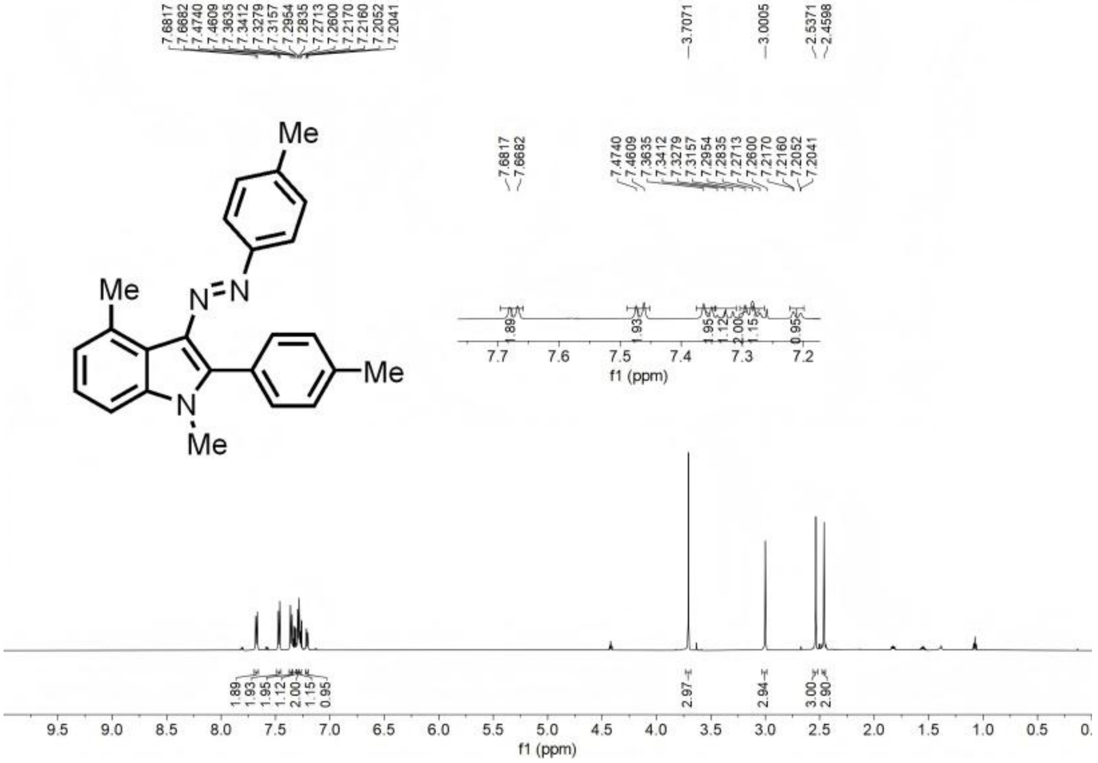

Fig. S20B: ^{13}C NMR of product **3r** in $CDCl_3$ (151 MHz)

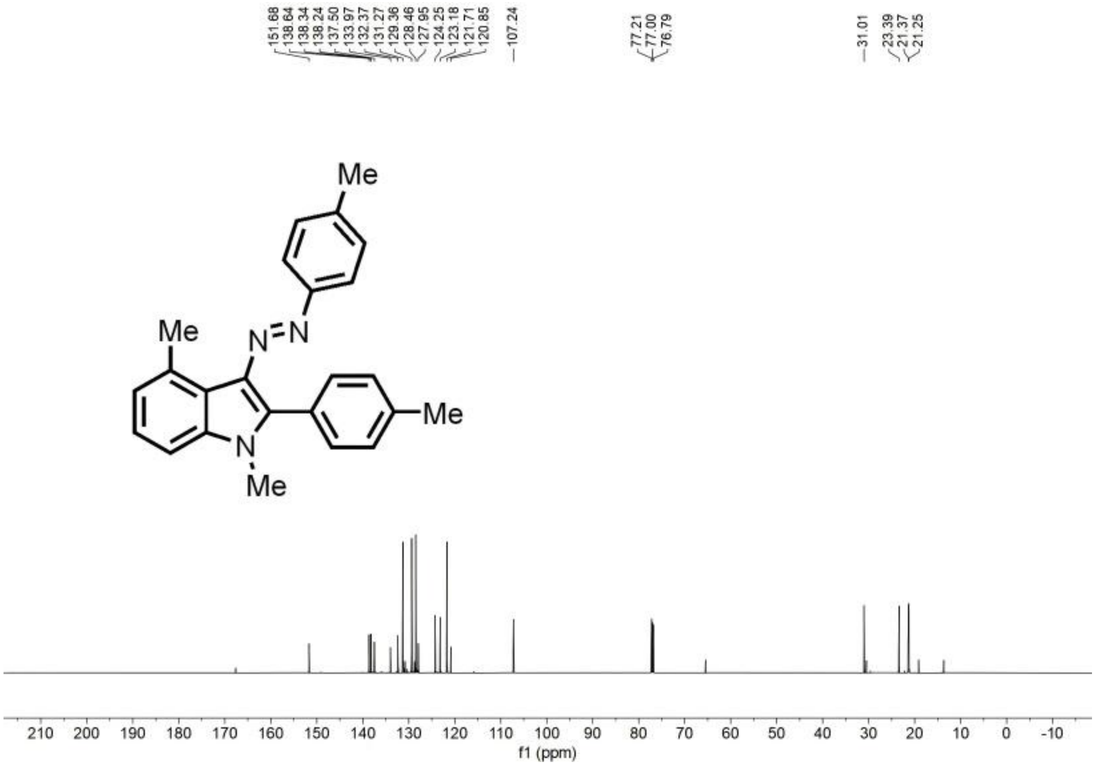

Fig. S21A: ^{1}H NMR of product **3s** in $CDCl_3$ (600 MHz)

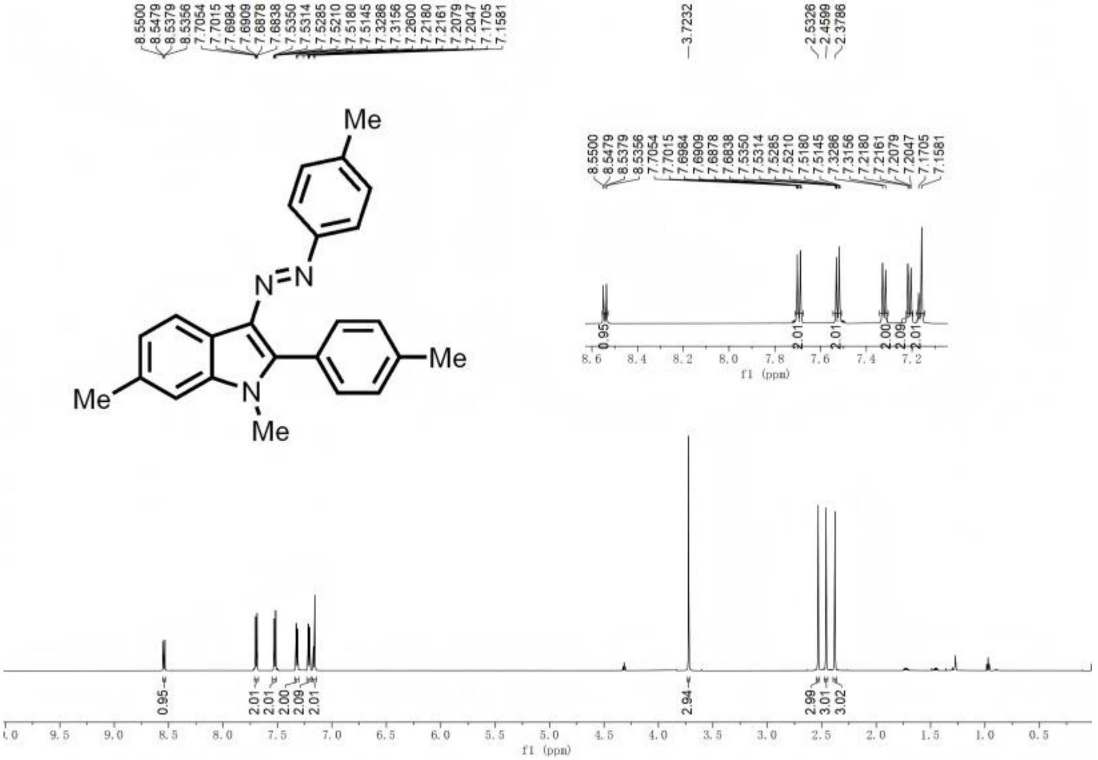

Fig. S21B: ^{13}C NMR of product **3s** in $CDCl_3$ (151 MHz)

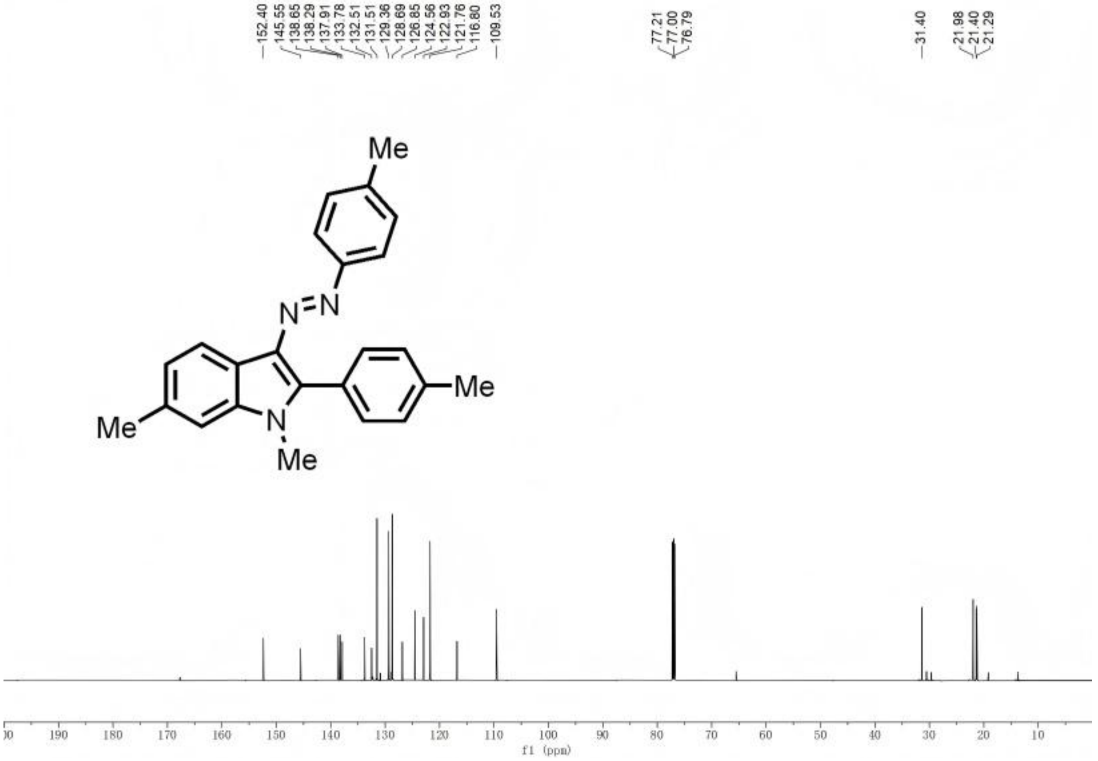

Fig. S22A: ^{1}H NMR of product **3t** in $CDCl_3$ (400 MHz)

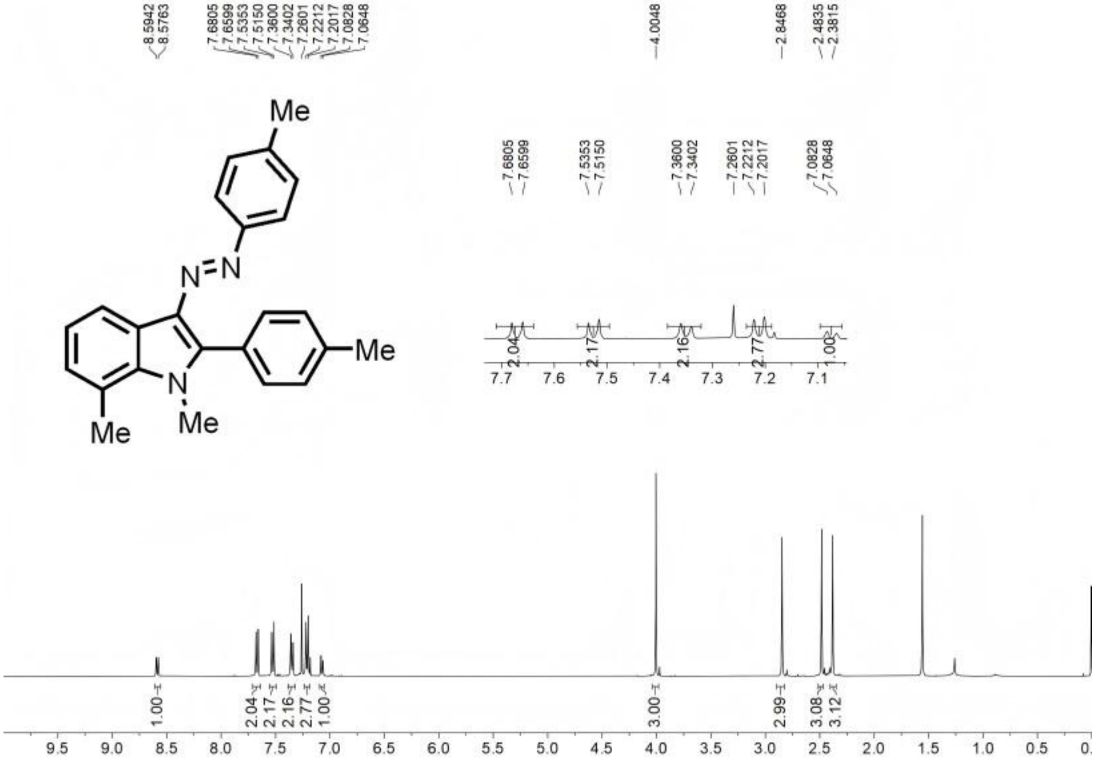

Fig. S22B: ^{13}C NMR of product **3t** in $CDCl_3$ (101 MHz)

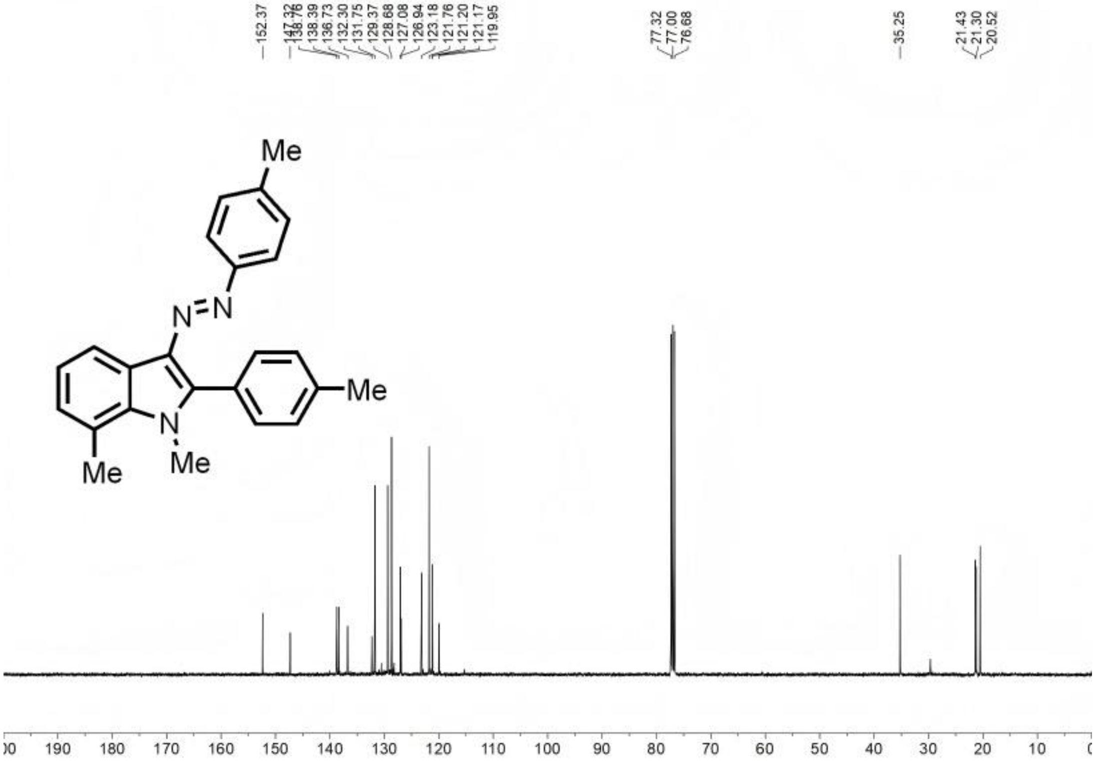

Fig. S23A: ^{1}H NMR of product **3u** in $CDCl_3$ (400 MHz)

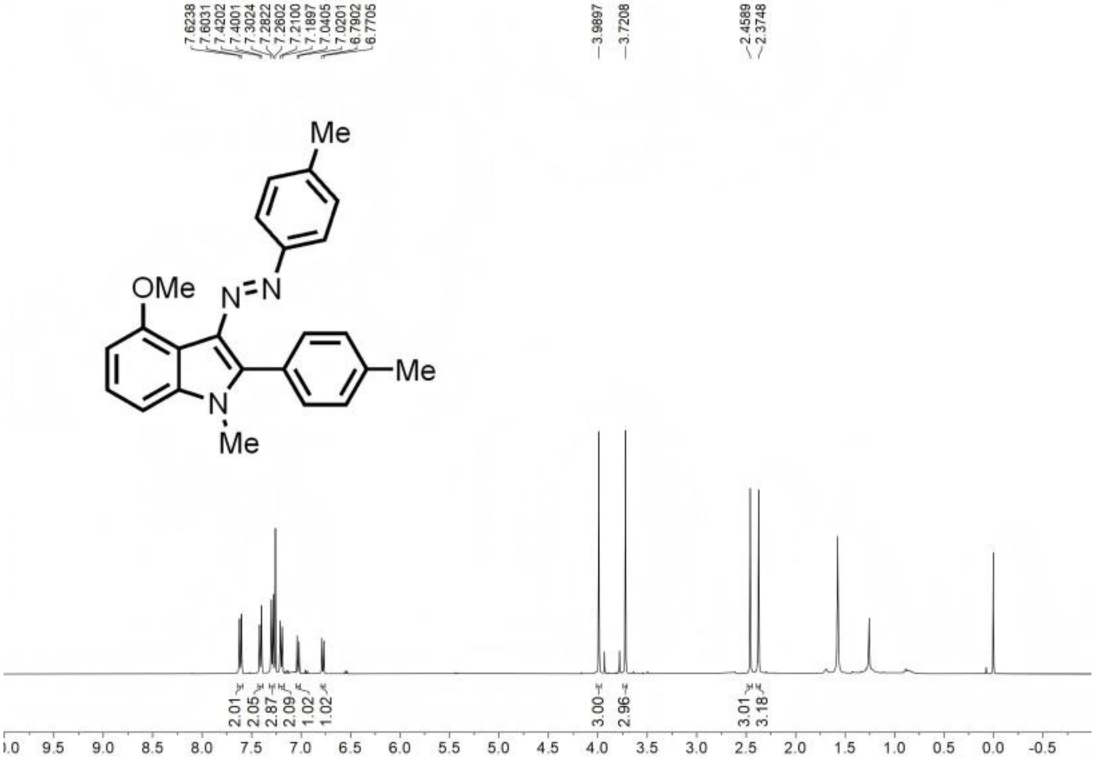

Fig. S23B: ^{13}C NMR of product **3u** in $CDCl_3$ (101 MHz)

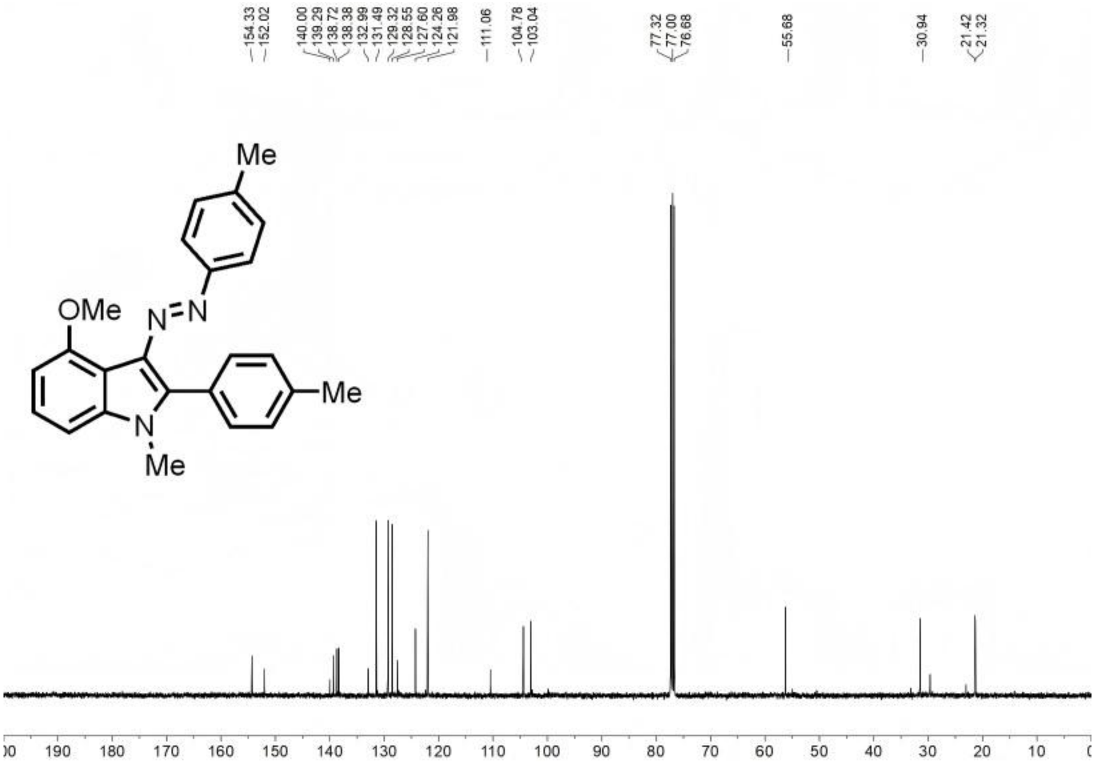

Fig. S24A: ^{1}H NMR of product **3v** in $CDCl_3$ (400 MHz)

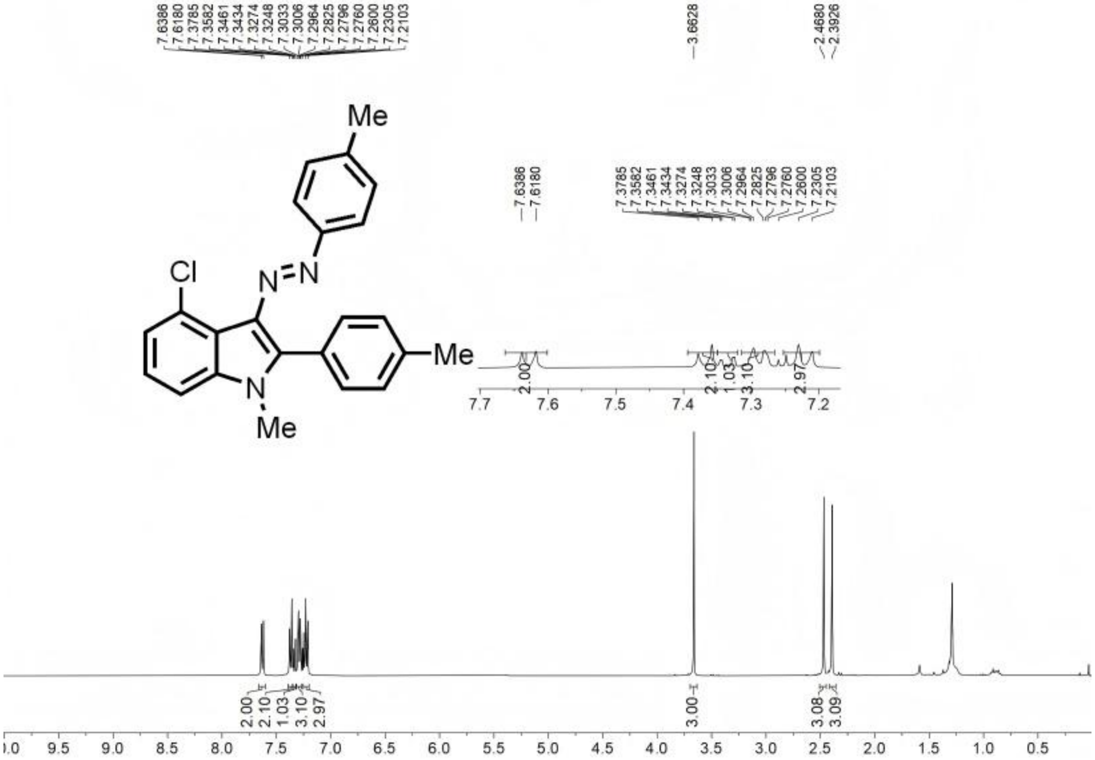

Fig. S24B: ^{13}C NMR of product **3v** in $CDCl_3$ (101 MHz)

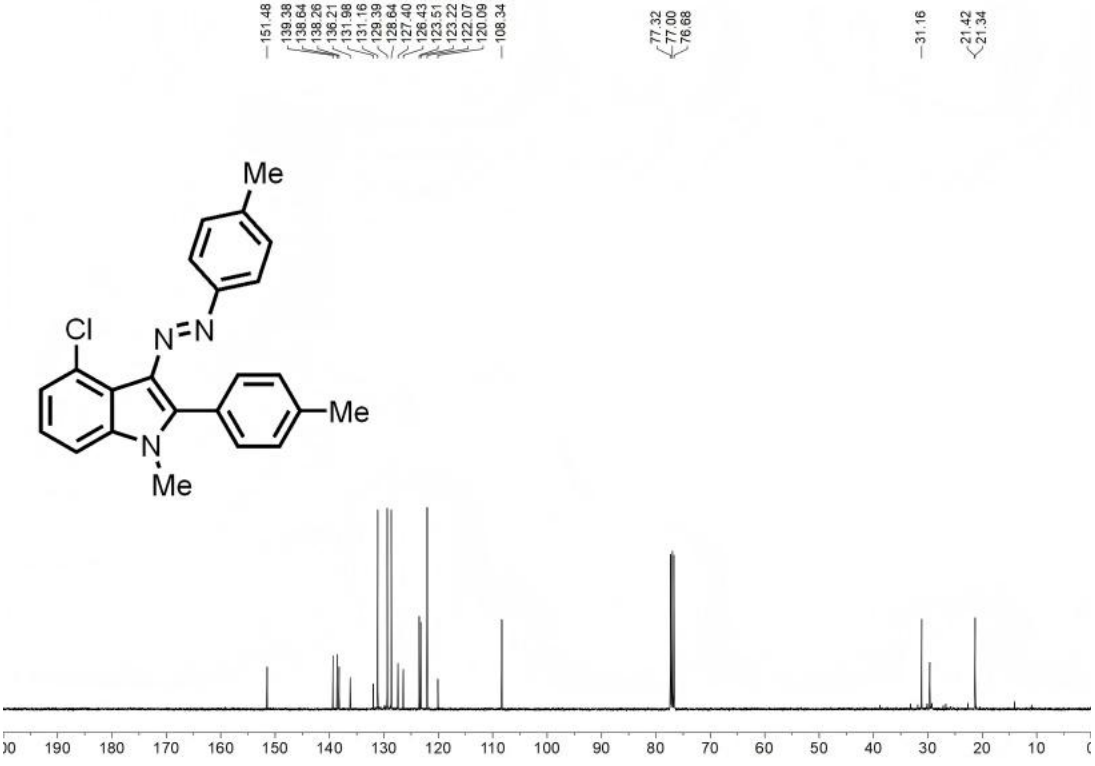

Fig. S25A: ^{1}H NMR of product **3w** in $CDCl_3$ (600 MHz)

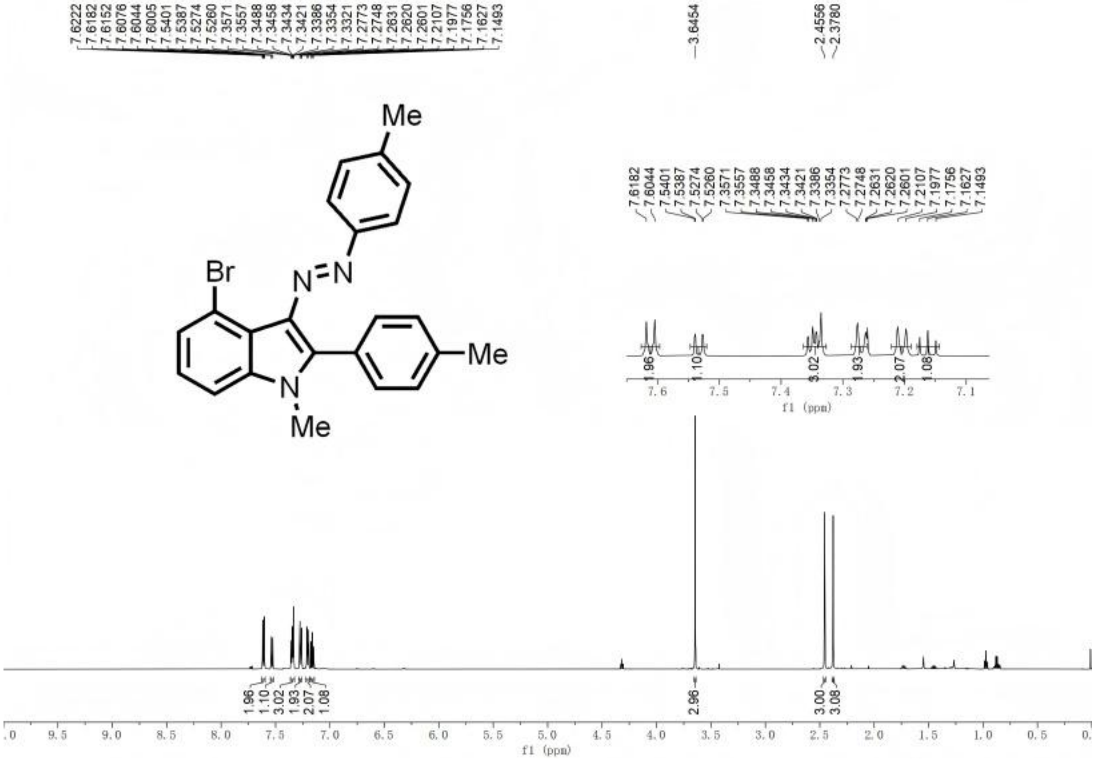

Fig. S25B: ^{13}C NMR of product **3w** in $CDCl_3$ (151 MHz)

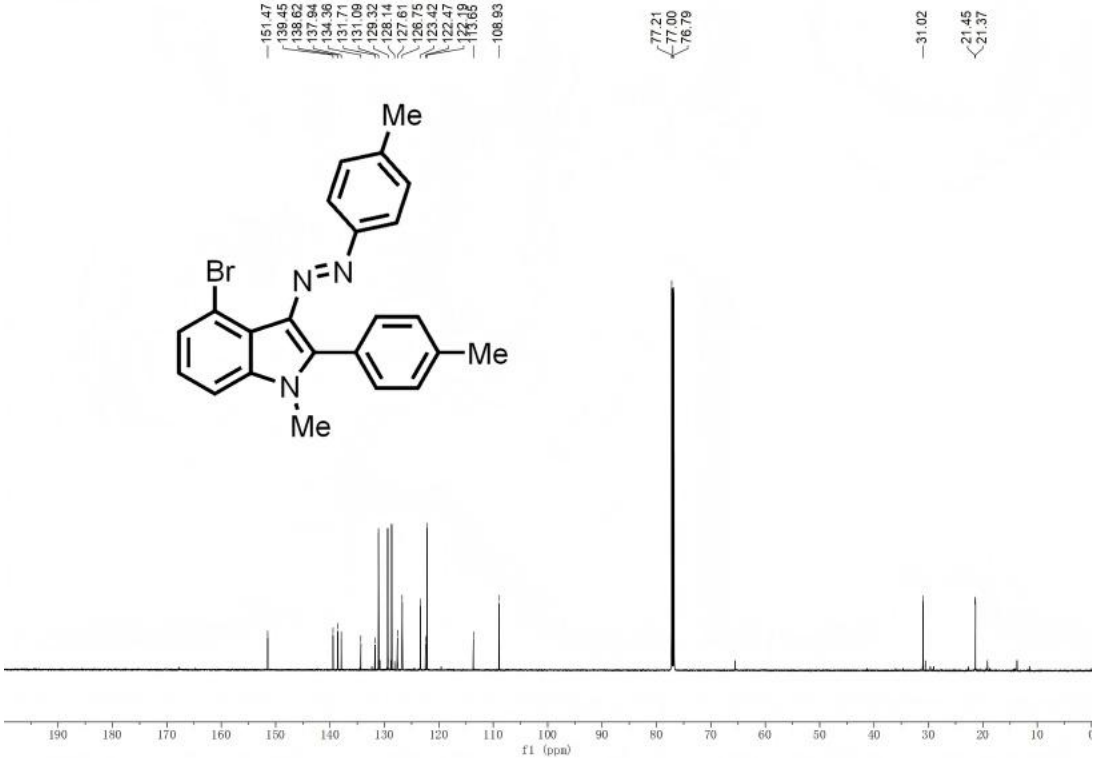

Fig. S26A: ^{1}H NMR of product **3x** in $CDCl_3$ (400 MHz)

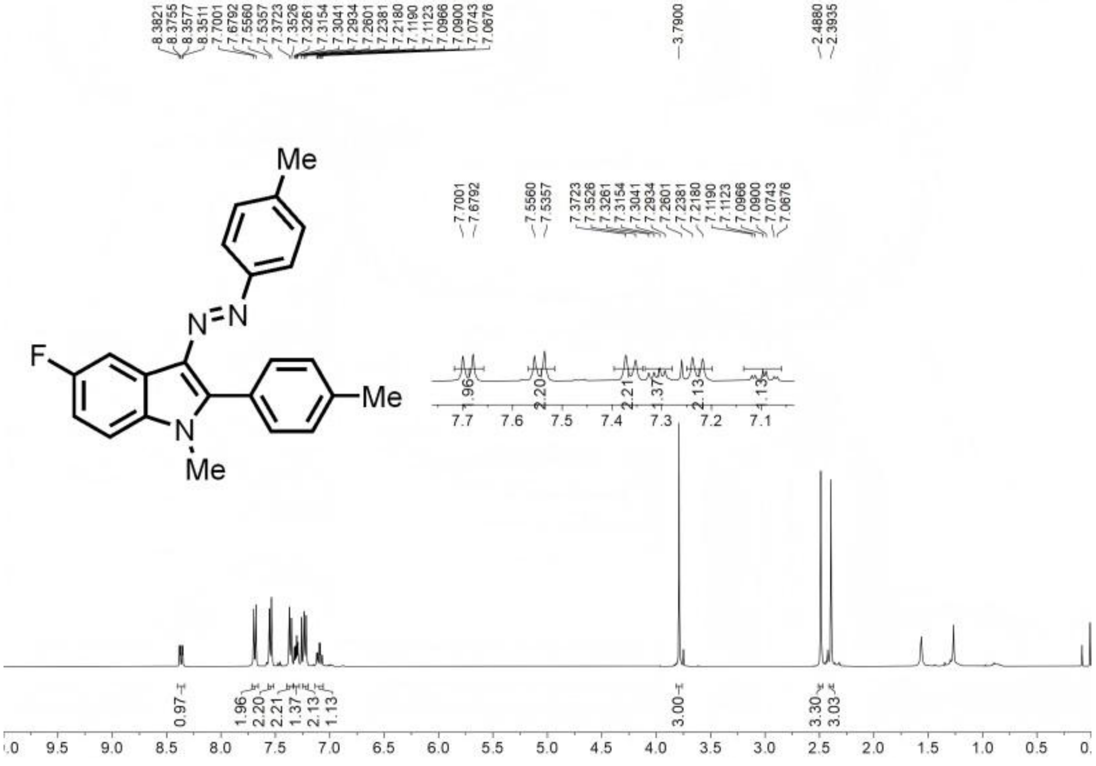

Fig. S26B: ^{13}C NMR of product **3x** in $CDCl_3$ (101 MHz)

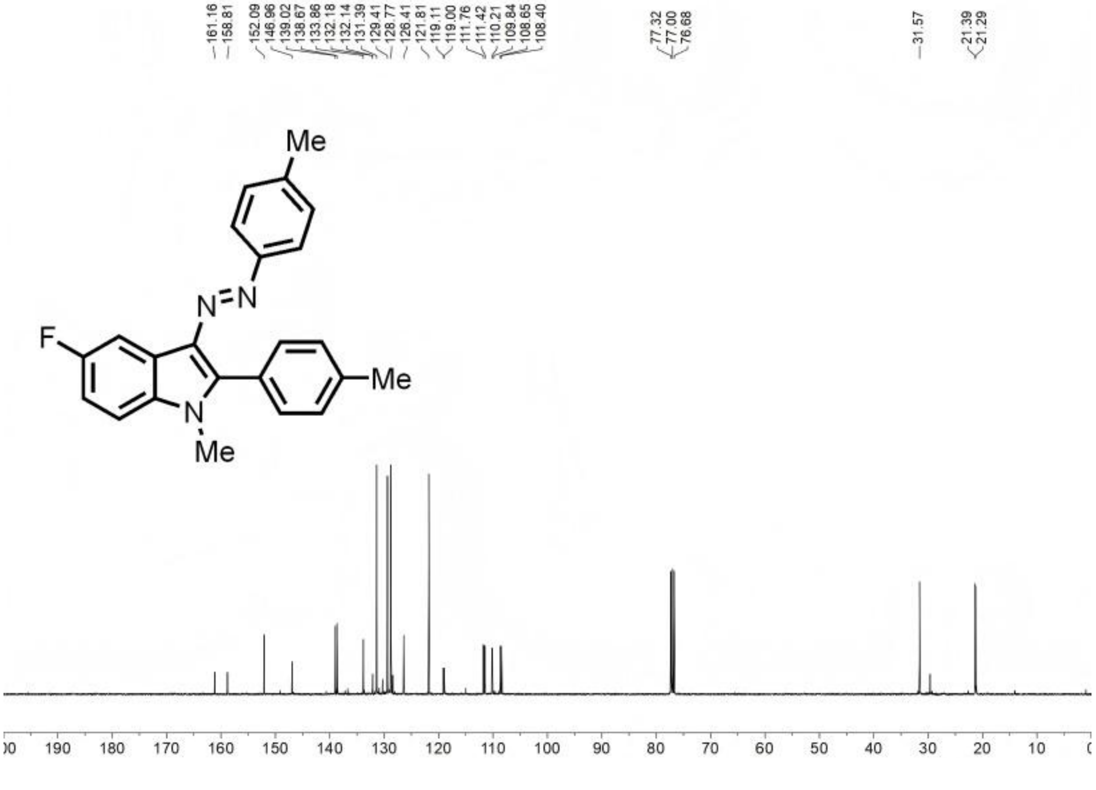

Fig. S27A: ^{1}H NMR of product **3y** in $CDCl_3$ (600 MHz)

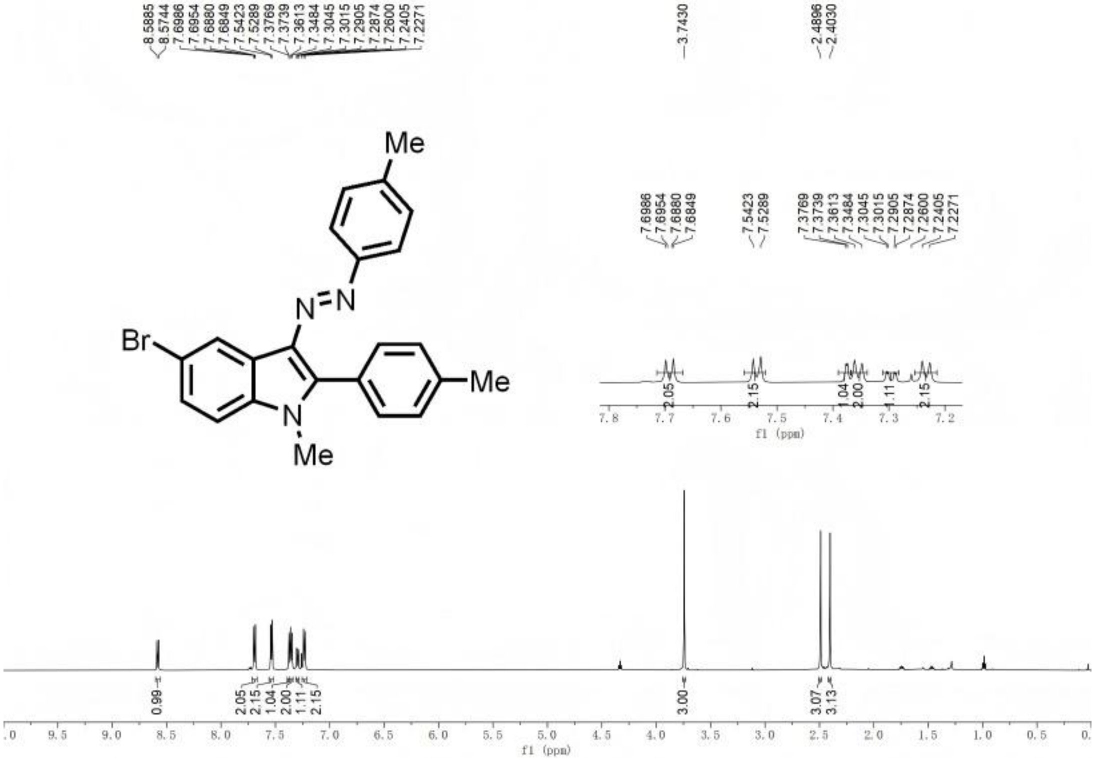

Fig. S27B: ^{13}C NMR of product **3y** in $CDCl_3$ (151 MHz)

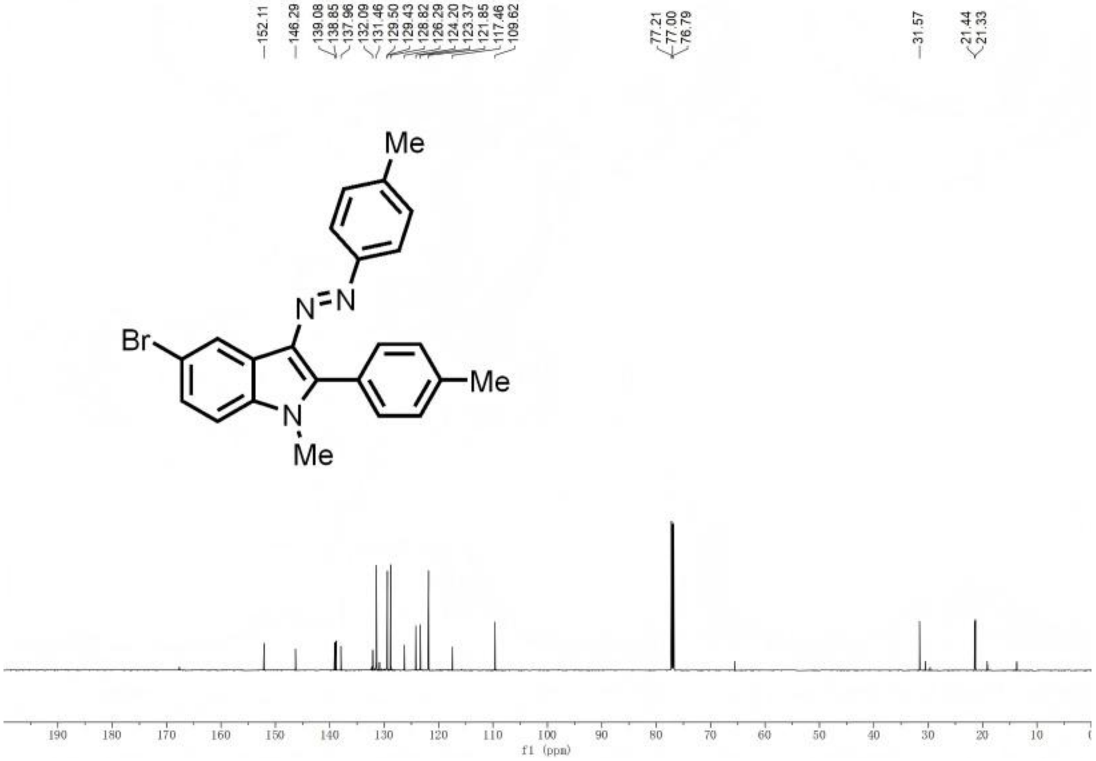

Fig. S28A: ^{1}H NMR of product **3z** in $CDCl_3$ (600 MHz)

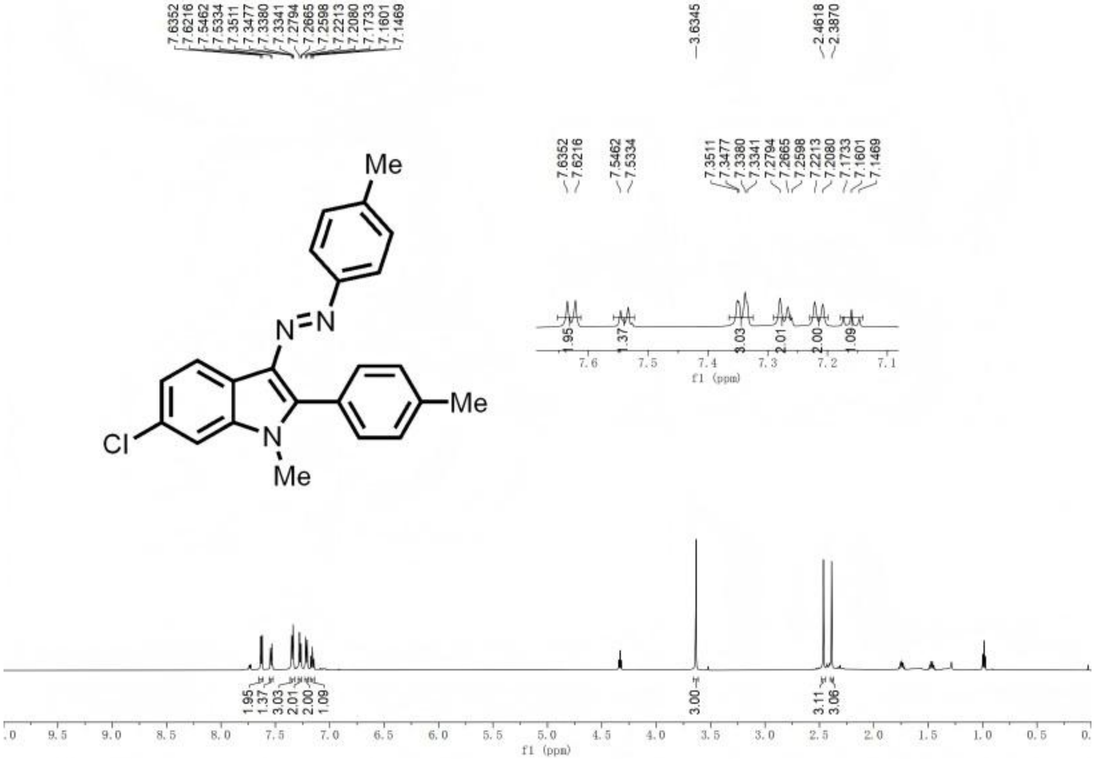

Fig. S28B: ^{13}C NMR of product **3z** in $CDCl_3$ (101 MHz)

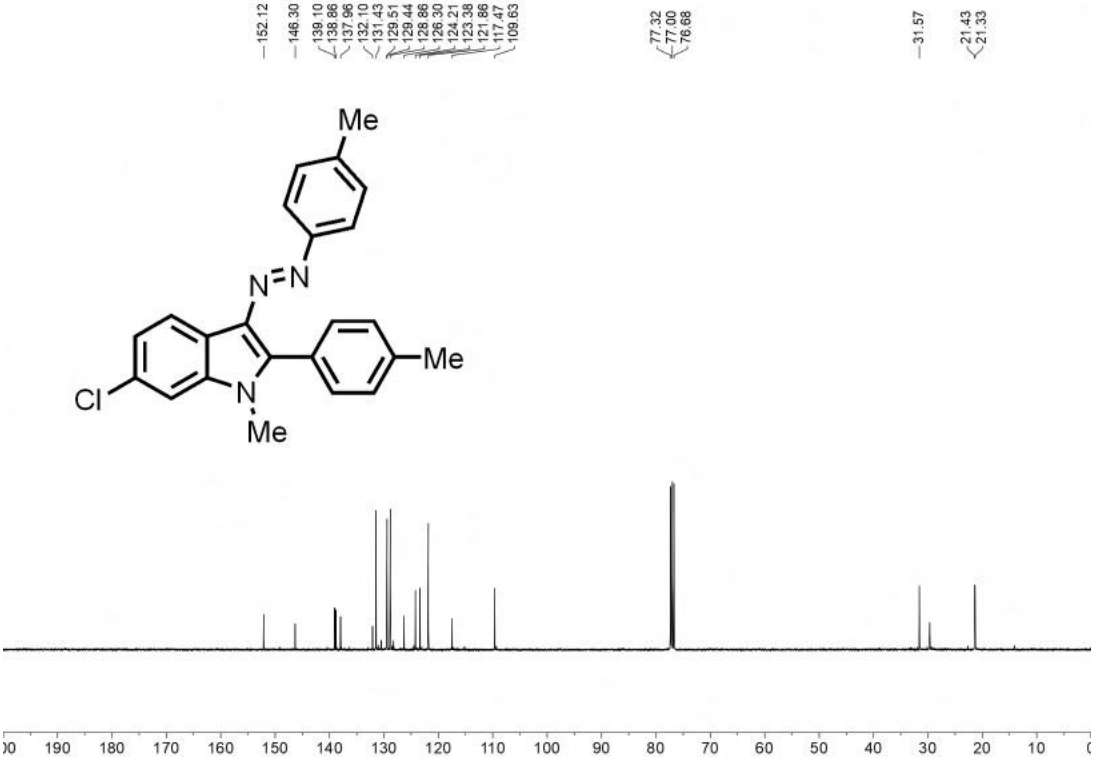

Fig. S29A: ^{1}H NMR of product **4a** in DMSO (600 MHz)

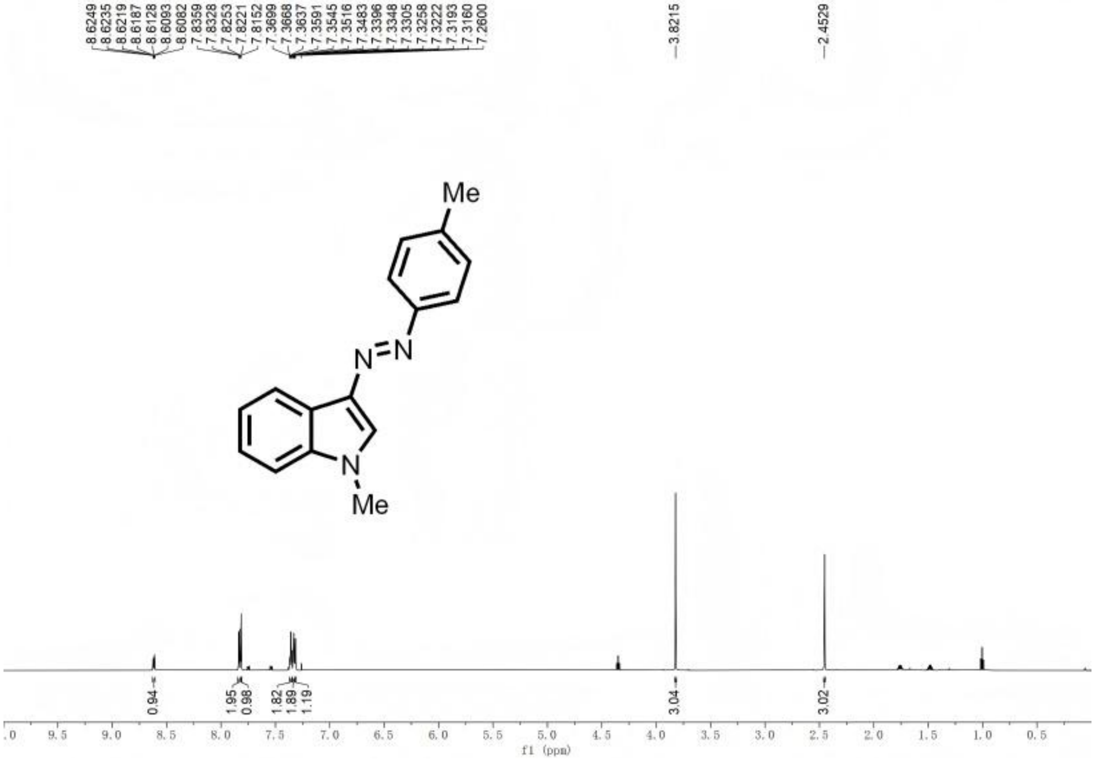

Fig. S29B: ^{13}C NMR of product **4a** in $CDCl_3$ (151 MHz)

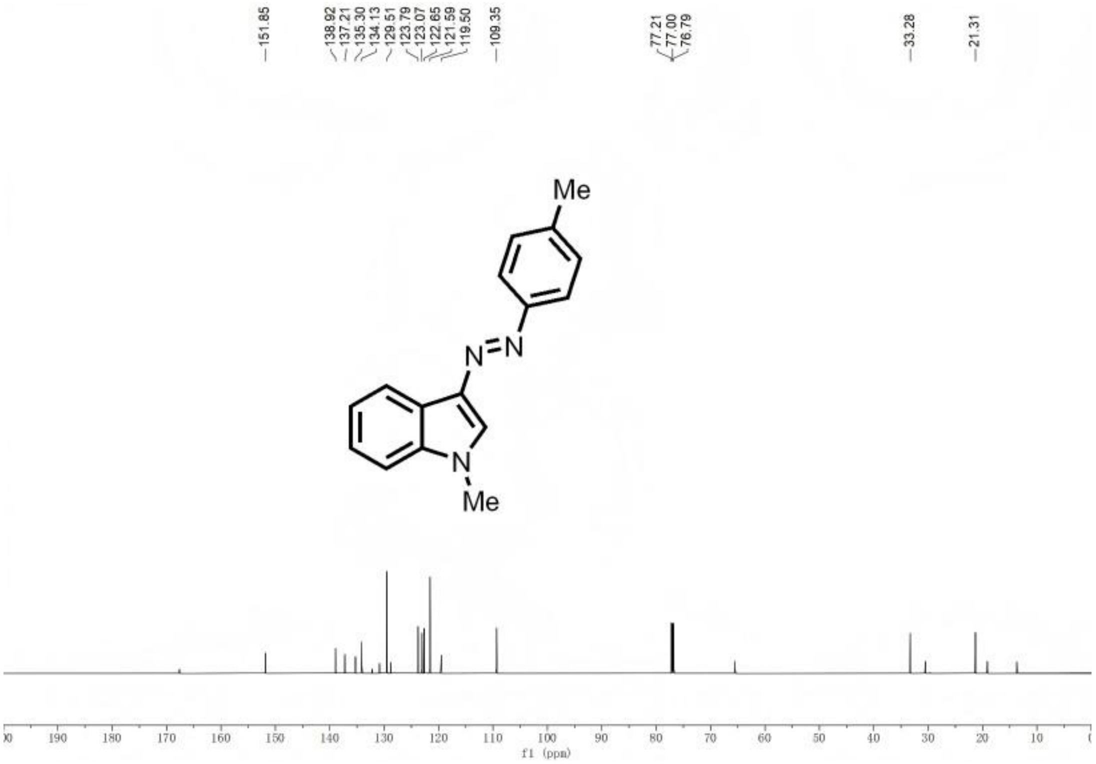

Fig. S30A: ^{1}H NMR of product **3aa** in DMSO (400 MHz)

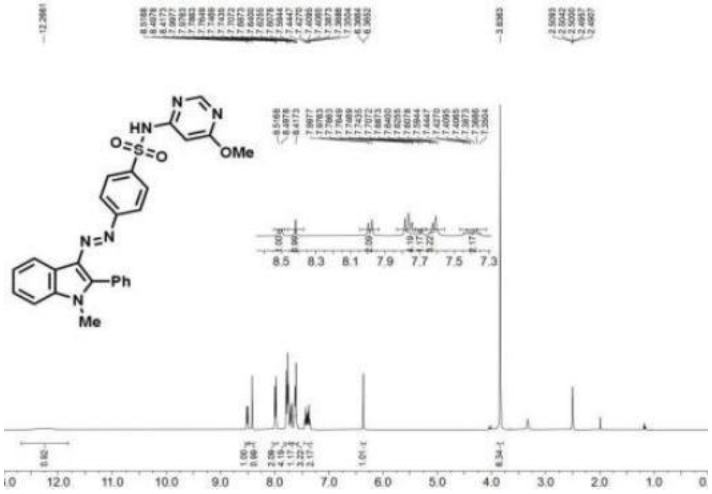

Fig. S30B: ^{13}C NMR of product **3aa** in DMSO (101 MHz)

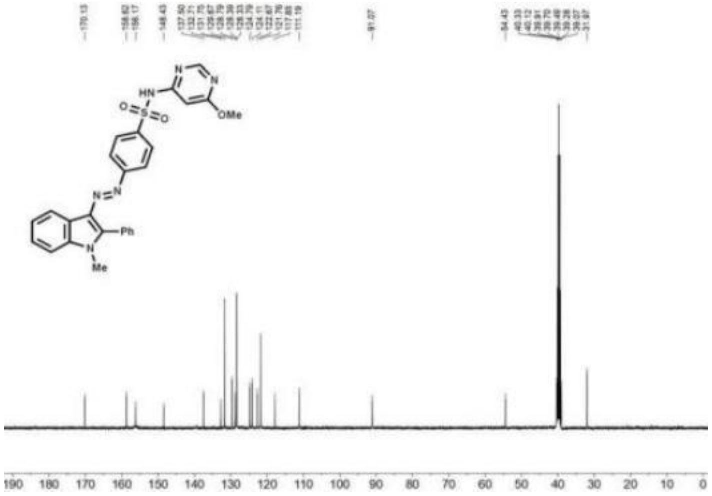

Fig. S30C: UV-Vis Absorption Spectrum of **3aa**

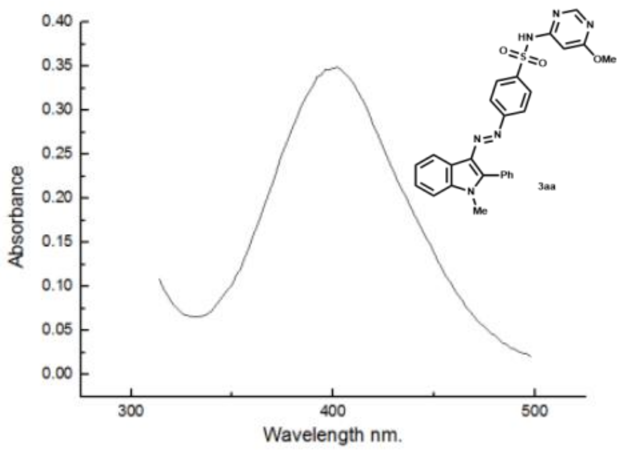

Fig. S31A: ^{1}H NMR of product **3ab** in DMSO (400 MHz)

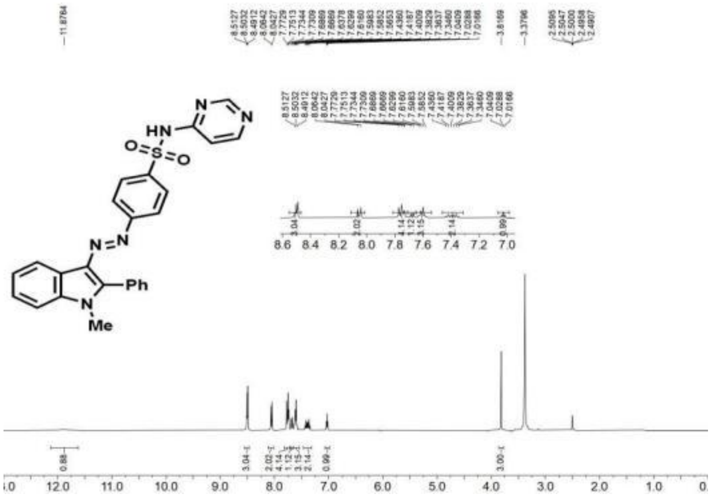

Fig. S31B: ^{13}C NMR of product **3ab** in DMSO (101 MHz)

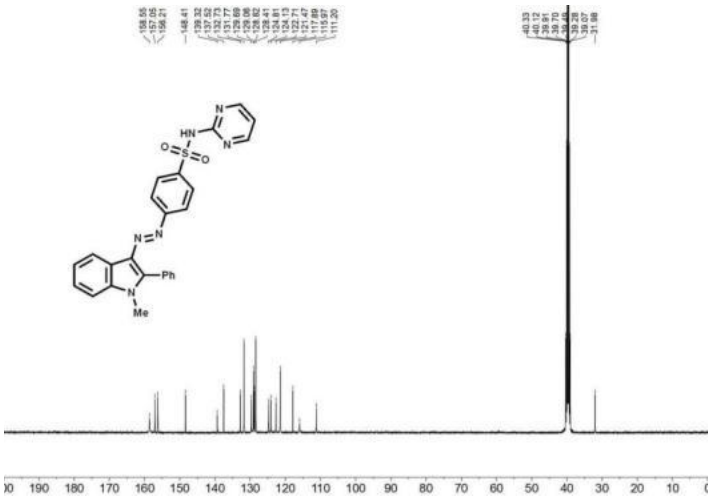

Fig. S31C: UV-Vis Absorption Spectrum of **3ab**

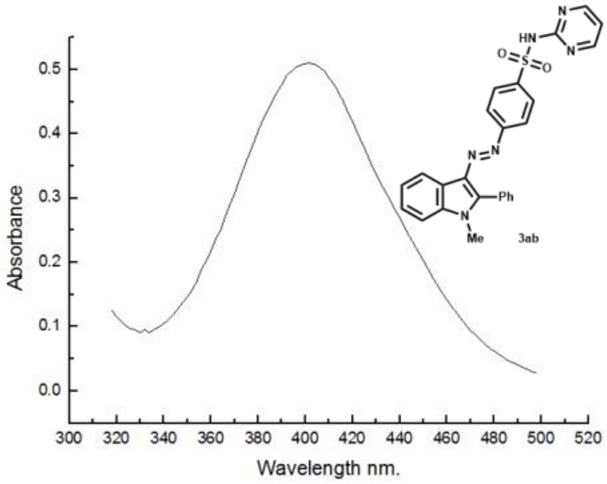

Fig. S32A: ^{1}H NMR of product **5a** in $CDCl_3$ (600 MHz)

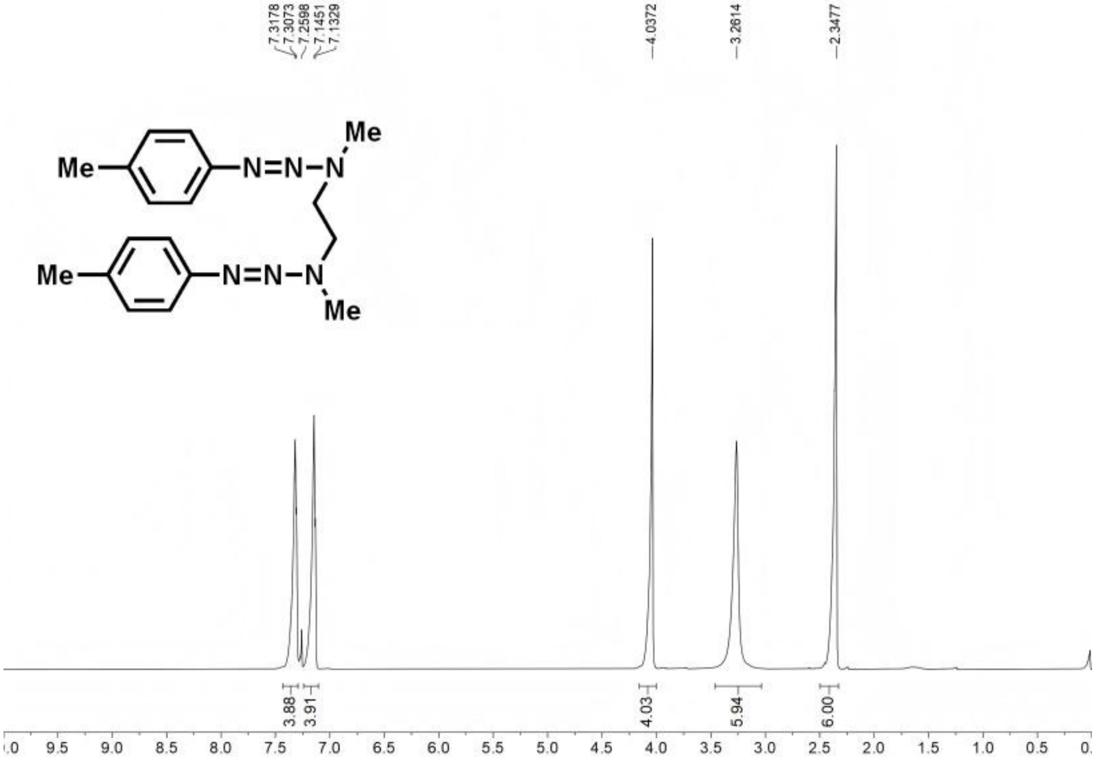

Fig. S32B: ^{13}C NMR of product **5a** in $CDCl_3$ (151 MHz)

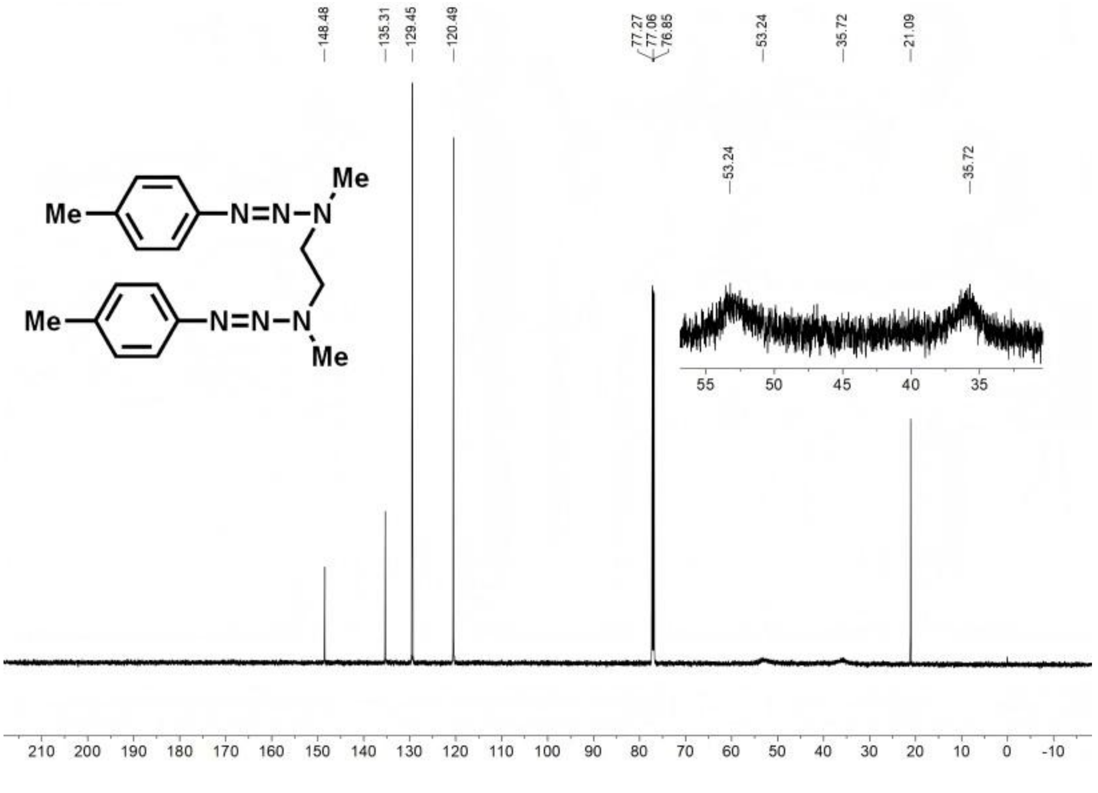

Fig. S33A: ^{1}H NMR of product **5b** in $CDCl_3$ (600 MHz)

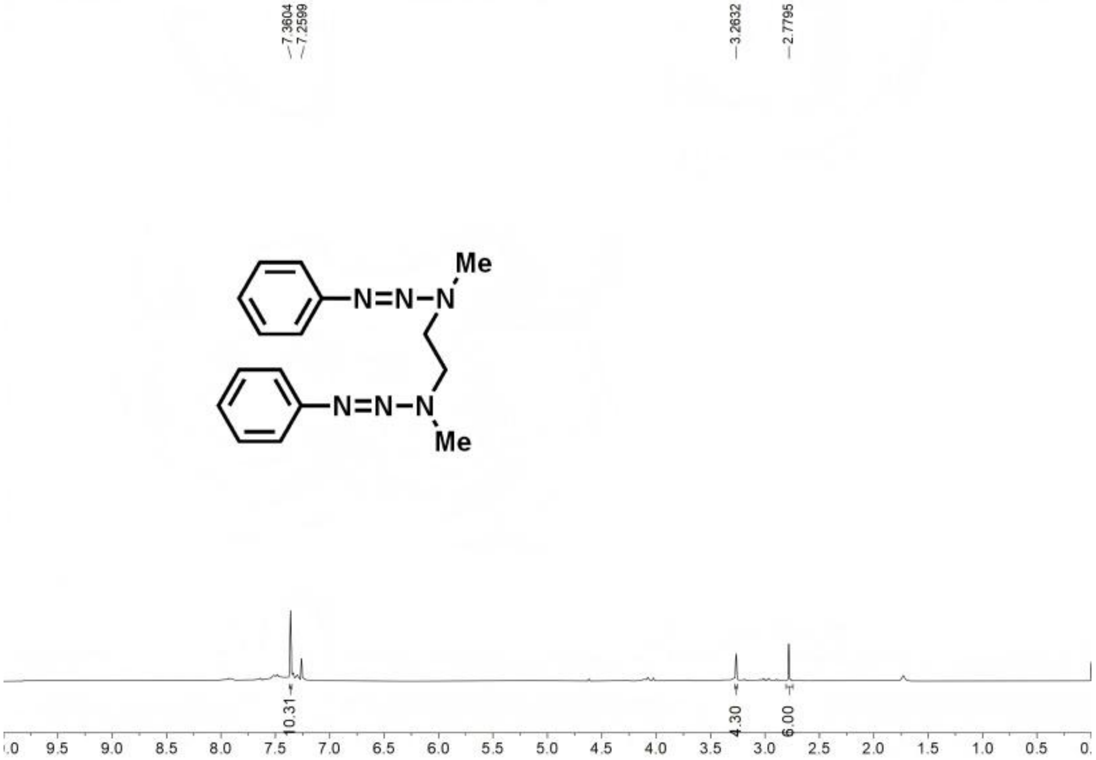

Fig. S33B: ^{13}C NMR of product **5b** in $CDCl_3$ (151 MHz)

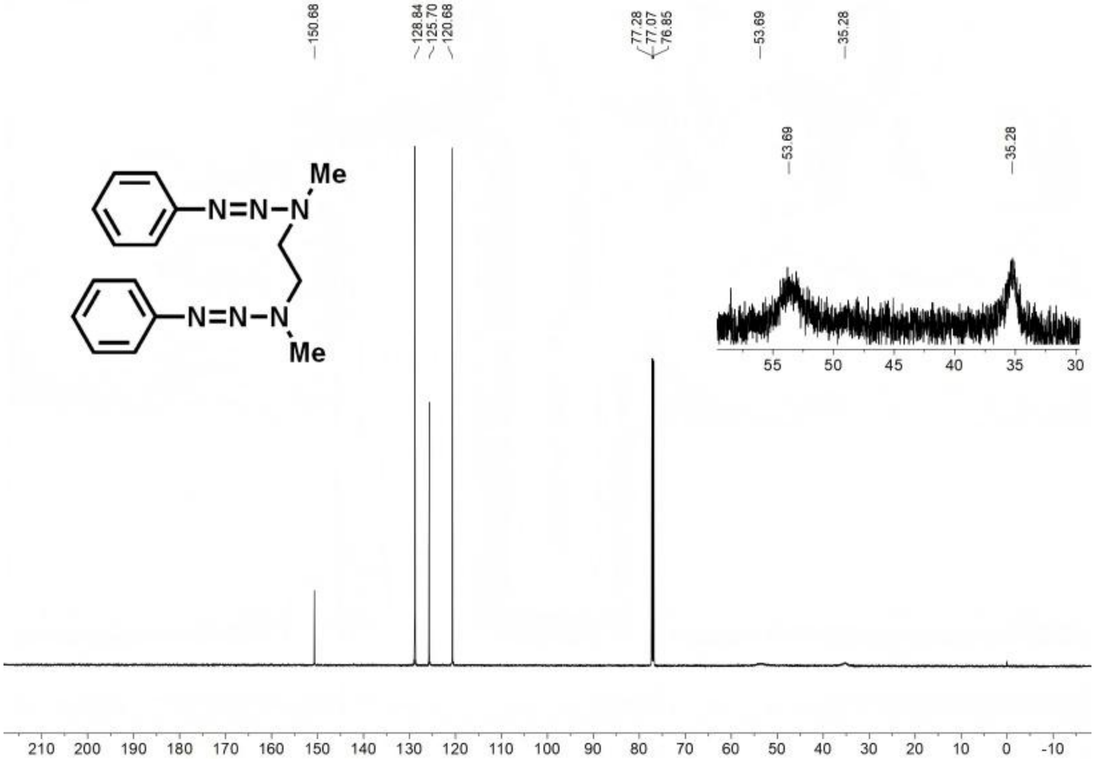

Fig. S34A: ^{1}H NMR of product **5e** in $CDCl_3$ (600 MHz)

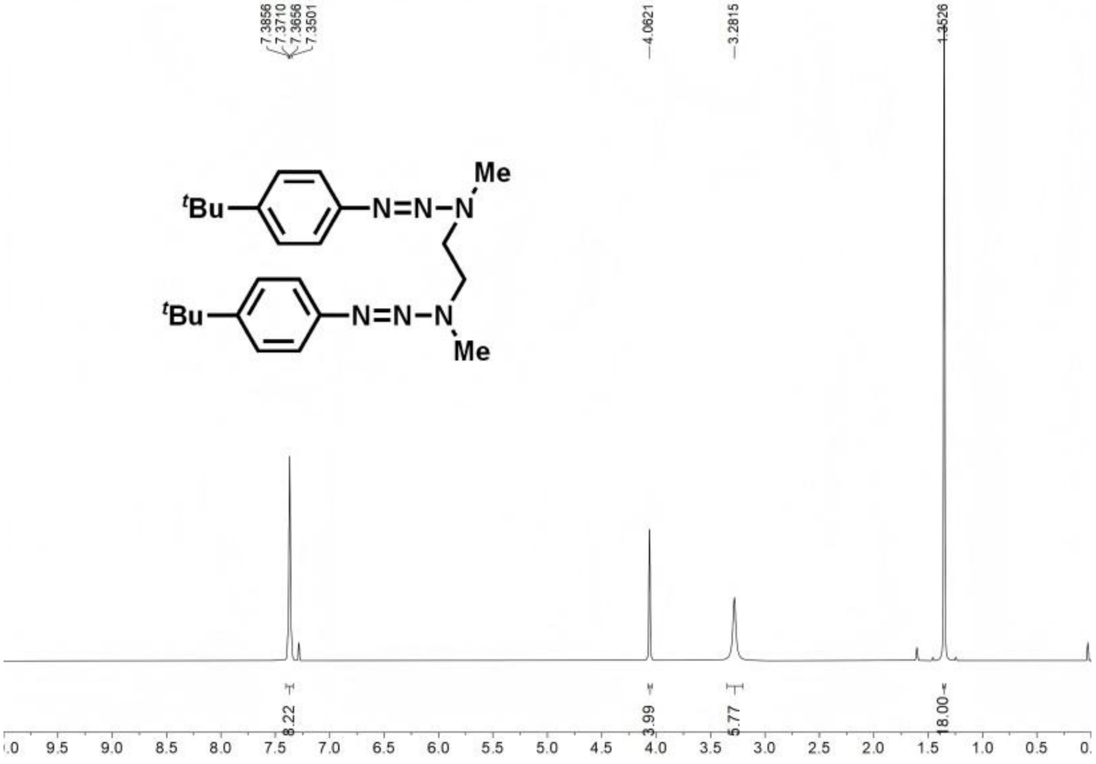

Fig. S34B: ^{13}C NMR of product **5e** in $CDCl_3$ (151 MHz)

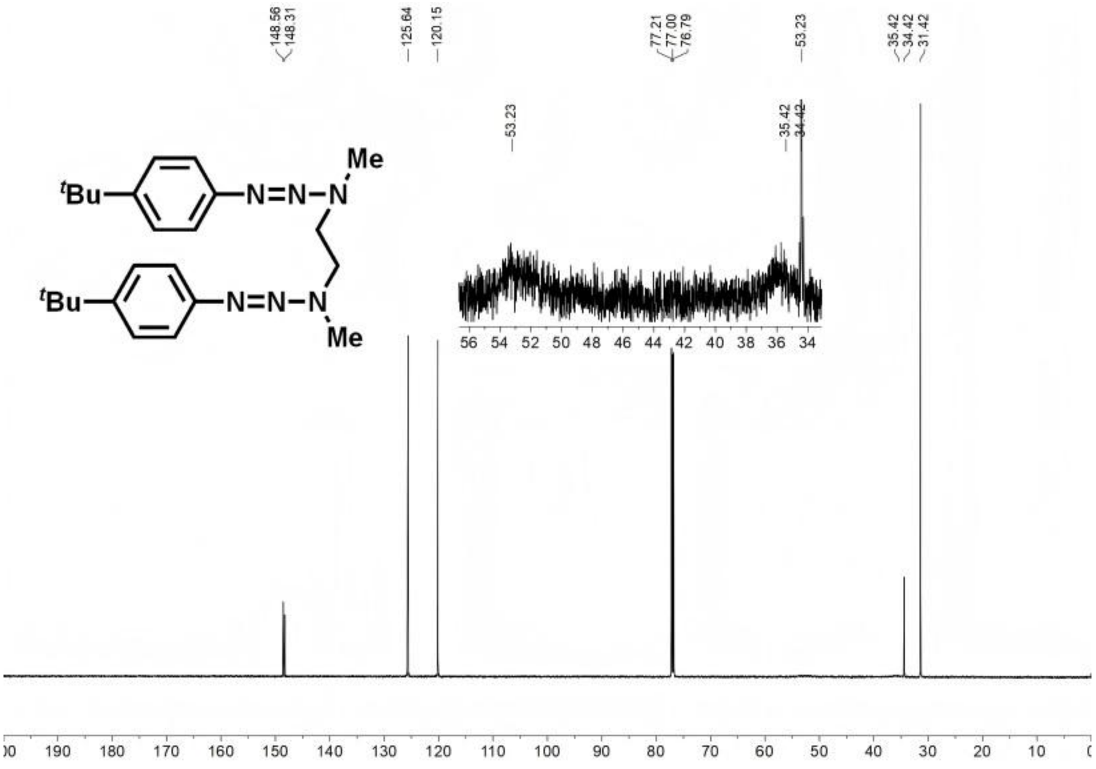

Fig. S35A: ^{1}H NMR of product **5i** in $CDCl_3$ (600 MHz)

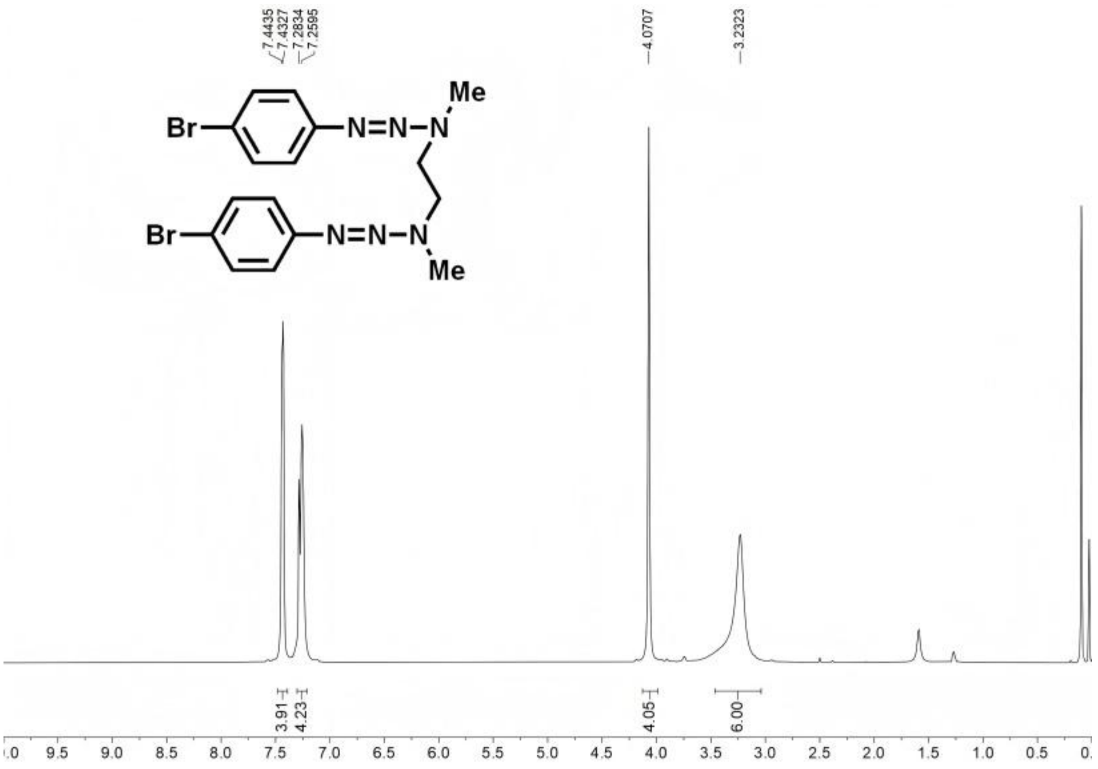

Fig. S35B: ^{13}C NMR of product **5i** in $CDCl_3$ (151 MHz)

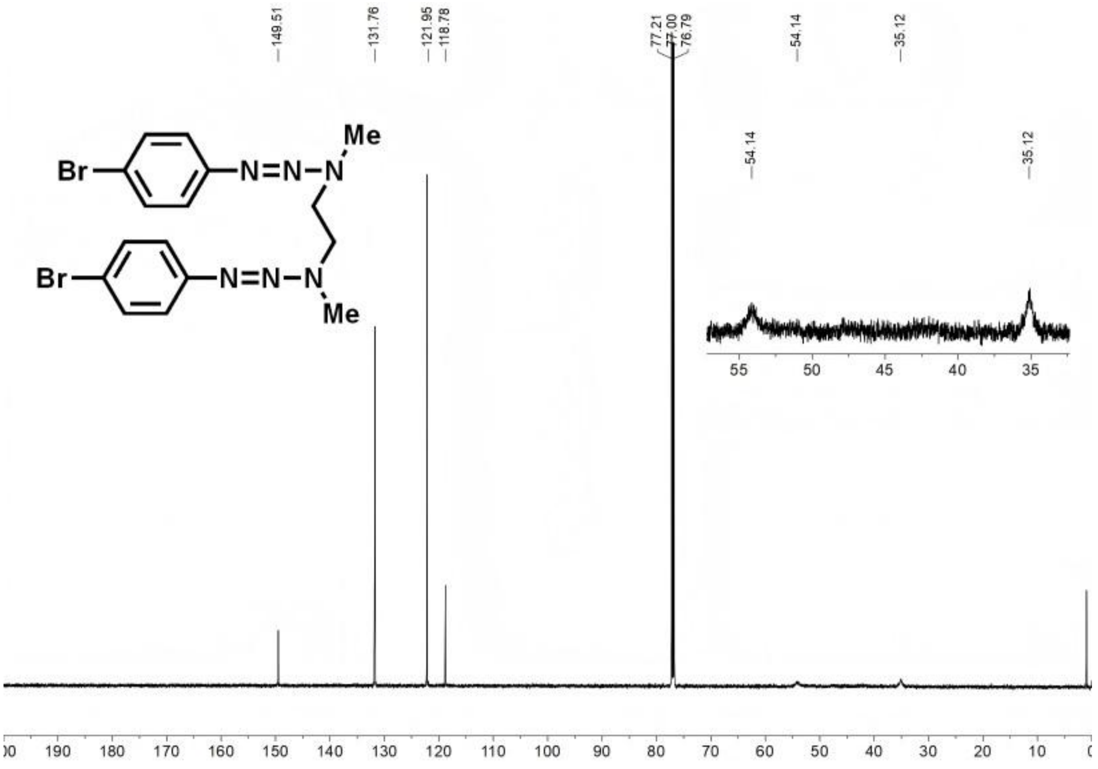

Supplement: Document S1. Figures S1–S35, Schemes S1–S3, and Table S1 [file mmc1.pdf]
