# Supplementary material for: Metal-free molecular editing of indole via tandem reaction: Access to 2-aryl-3-aryldiazenylindole for theranostic applications
Source: iScience. 2025 Aug 8;28(9):113325. doi: 10.1016/j.isci.2025.113325 (PMC12496193; doi:10.1016/j.isci.2025.113325)
Supplement: Data S1. Analytical data [file mmc3.pdf]

Date s1. Analytical Data

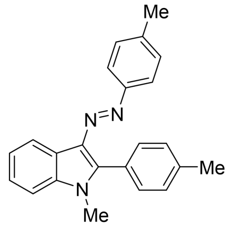

(*E*)-1-Methyl-2-(*p*-tolyl)-3-(*p*-tolyldiazenyl)-1*H*-indole (3a): Yellow semisolid (55.8 mg, yield: 82%). ^{1}H NMR (600 MHz, $CDCl_3$) δ 8.73-8.69 (m, 1H), 7.72 (dt, *J* = 8.3, 2.3 Hz, 2H), 7.57 (dt, *J* = 8.0, 2.1 Hz, 2H), 7.42-7.40 (m, 1H), 7.40-7.35 (m, 4H), 7.24 (dd, *J* = 8.5, 0.9 Hz, 2H), 3.80 (s, 3H), 2.50 (s, 3H), 2.41 (s, 3H); ^{13}C NMR (151 MHz, $CDCl_3$) δ 152.35, 145.93, 138.84, 138.47, 137.43, 132.42, 131.52, 129.40, 128.76, 126.75, 123.82, 123.25, 123.07, 121.80, 118.92, 109.43, 32.34, 21.43, 21.31.; HRMS (ESI$^+$): *m/z* calcd. for $C_{23}H_{22}N_3$ [M+H]$^+$: 340.1808, found: 340.1806.

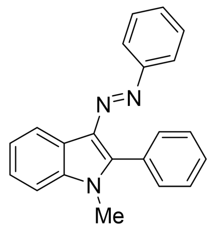

(*E*)-1-Methyl-2-phenyl-3-(phenyldiazenyl)-1*H*-indole (3b): Yellow solid (49.2 mg, yield: 79%), mp: 147.3-147.7 °C. ^{1}H NMR (600 MHz, $CDCl_3$) δ 8.73-8.69 (m, 1H), 7.82-7.79 (m, 2H), 7.69-7.66 (m, 2H), 7.59-7.55 (m, 2H), 7.55-7.51 (m, 1H), 7.45-7.42 (m, 3H), 7.42-7.36 (m, 2H), 7.32 (tt, *J* = 7.3, 1.3 Hz, 1H), 3.82 (s, 3H); ^{13}C NMR (151 MHz, $CDCl_3$) δ 154.23, 146.22, 137.46, 132.65, 131.66, 129.65, 128.90, 128.77, 128.49, 128.03, 124.05, 123.34, 123.31, 121.90, 118.80, 109.52, 31.54.; HRMS (ESI$^+$): calcd. for $C_{21}H_{18}N_3$ [M+H]$^+$: 312.1495, found: 312.1493.[1]

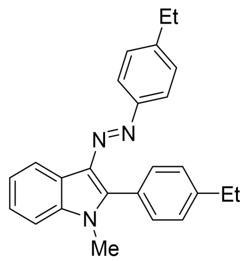

(*E*)-2-(*p*-Ethylphenyl)-3-((*p*-ethylphenyl)diazenyl)-1-methyl-1*H*-indole (3c): Yellow solid (39.6 mg, yield: 54%), mp: 95.5-97.5 °C. ^{1}H NMR (600 MHz, $CDCl_3$) δ 8.75-8.71 (m, 1H), 7.77 (dt, *J* = 8.3, 1.7 Hz, 2H), 7.61 (dd, *J* = 6.0, 1.9 Hz, 2H), 7.42-7.37 (m, 5H), 7.29 (dd, *J* = 8.4, 2.0 Hz, 2H), 3.80 (s, 3H), 2.81 (q, *J* = 7.6 Hz, 2H), 2.72 (q, *J* = 8.4 Hz, 2H), 1.37 (t, *J* = 7.6 Hz, 3H), 1.30 (t, *J* = 7.6 Hz, 3H); ^{13}C NMR

(151 MHz, $CDCl_3$) δ 152.55, 145.95, 145.02, 144.83, 137.43, 132.46, 131.62, 128.20, 127.53, 126.93, 123.80, 123.26, 123.06, 121.86, 118.93, 109.43, 31.51, 28.74, 28.69, 15.55, 15.32; HRMS (ESI$^+$): *m/z* calcd. for $C_{25}H_{26}N_3$ $[M+H]^+$: 368.2121, found: 368.2117.

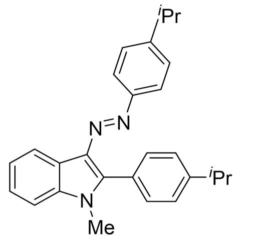

(*E*)-2-(*p*-Isopropylphenyl)-3-((*p*-isopropylphenyl)diazenyl)-1-methyl-1*H*-indole (3d): Yellow solid (51.4 mg, yield: 65%), mp: 93.5-95.5 °C. ^{1}H NMR (600 MHz, $CDCl_3$) δ 8.74-8.69 (m, 1H), 7.76 (dd, *J* = 6.4, 1.4 Hz, 2H), 7.61 (dt, *J* = 8.2, 2.0 Hz, 2H), 7.43-7.36 (m, 5H), 7.36 (dd, *J* = 6.2, 1.7 Hz, 2H), 3.82 (s, 3H), 3.05 (hept, *J* = 7.0 Hz, 1H), 2.97 (hept, *J* = 6.8 Hz, 1H), 1.37 (d, *J* =7.0 Hz, 6H), 1.30 (d, *J* =7.0 Hz, 6H); ^{13}C NMR (151 MHz, $CDCl_3$) δ 152.67, 149.61, 149.41, 145.94, 137.45, 132.50, 131.64, 127.04, 126.77, 126.16, 123.81, 123.29, 123.07, 121.85, 118.95, 109.43, 34.04, 33.96, 31.59, 23.97, 23.91; HRMS (ESI$^+$): *m/z* calcd. for $C_{27}H_{30}N_3$ $[M+H]^+$: 396.2434, found: 396.2430.

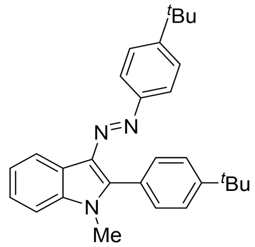

(*E*)-2-(*p*-(Tert-butyl)phenyl)-3-((*p*-(tert-butyl)phenyl)diazenyl)-1-methyl-1*H*-indole (3e):Yellow semisolid (67.8 mg, yield: 80%). ^{1}H NMR (600 MHz, $CDCl_3$) δ 8.77-8.74 (m, 1H), 7.80 (dd, *J* = 6.5, 1.9 Hz 2H), 7.63 (dd, *J* = 6.4, 1.9 Hz 2H), 7.59 (dd, *J* = 6.5, 2.1 Hz, 2H), 7.51 (dd, *J* = 6.5, 1.9 Hz, 2H), 7.42-7.38 (m, 3H), 3.80 (s, 3H), 1.46 (s, 9H), 1.41 (s, 9H); ^{13}C NMR (151 MHz, $CDCl_3$) δ 152.22, 151.90, 151.62, 145.90, 137.48, 132.60, 131.39, 130.49, 126.71, 125.68, 125.05, 124.43, 123.84, 123.33, 123.10, 121.51, 118.98, 109.43, 34.83, 34.75, 31.66, 31.56, 31.36, 31.34; HRMS (ESI+): *m/z* calcd. for $C_{29}H_{34}N_3$ $[M+H]^+$: 424.2747, found: 424.2744.

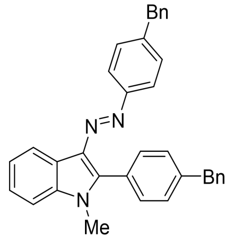

(*E*)-2-(*p*-Benzylphenyl)-3-((*p*-benzylphenyl)diazenyl)-1-methyl-1*H*-indole (3f): Yellow semisolid (29.5 mg, yield: 30%). ^1H NMR (600 MHz, $CDCl_3$) δ 8.69-8.66 (m, 1H), 7.72 (dt, *J* = 8.3, 2.5 Hz, 2H), 7.58 (dt, *J* = 8.2, 2.1 Hz, 2H), 7.41 (dd, *J* = 6.9, 1.4 Hz, 1H), 7.38 (dd, *J* = 6.9, 1.5 Hz, 1H), 7.37-7.33 (m, 5H), 7.32-7.27 (m, 4H), 7.27-7.24 (m, 3H), 7.23-7.20(m, 3H), 4.11 (s, 2H), 4.03 (s, 2H), 3.80 (s, 3H); ^{13}C NMR (151 MHz, $CDCl_3$) δ 152.77, 145.90, 142.04, 141.55, 141.05, 140.51, 137.47, 132.58, 131.74, 129.38, 129.11, 128.94, 128.58, 128.55, 128.45, 127.41, 126.29, 126.07, 123.94, 123.30, 123.19, 122.00, 118.88, 109.46, 41.82, 41.71, 31.60; HRMS (ESI^+): *m/z* calcd. for $C_{35}H_{30}N_3$ $[M+H]^+$: 492.2434, found: 492.2431.

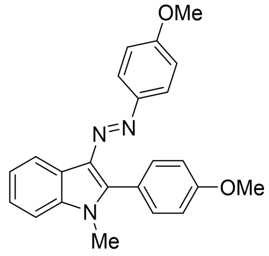

(*E*)-2-(*p*-Methoxyphenyl)-3-((*p*-methoxyphenyl)diazenyl)-1-methyl-1*H*-indole (3g): Yellow solid (36.4 mg, yield: 49%), mp: 175.2-176.7 °C. ^1H NMR (400 MHz, $CDCl_3$) δ 8.69-8.64 (m, 1H), 7.77 (d, *J* = 9.0 Hz, 2H), 7.60 (d, *J* = 8.7 Hz, 2H), 7.43-7.30 (m, 3H), 7.08 (d, *J* = 8.7, Hz, 2H), 6.95 (d, *J* = 9.0 Hz, 2H), 3.92 (s, 3H), 3.86 (s, 3H), 3.80 (s, 3H); ^{13}C NMR (101 MHz, $CDCl_3$) δ 160.11, 160.01, 148.68, 145.30, 137.38, 132.97, 132.18, 123.69, 123.25, 123.14, 122.91, 122.08, 119.00, 113.94, 113.57, 109.38, 55.45, 55.36, 31.48; HRMS (ESI^+): *m/z* calcd. for $C_{23}H_{22}N_3O_2$ $[M+H]^+$: 372.1707, found: 372.1704.

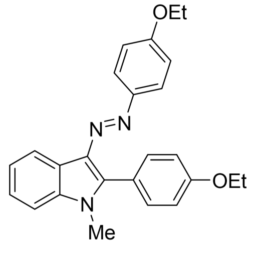

(*E*)-2-(*p*-Ethoxyphenyl)-3-((*p*-ethoxyphenyl)diazenyl)-1-methyl-1*H*-indole (3h): Yellow solid (38.4 mg, yield: 48%), mp: 127.0-128.2 °C. ^1H NMR (400 MHz, $CDCl_3$) δ 8.68-8.64 (m, 1H), 7.76 (d, *J* = 10.0 Hz, 2H), 7.39 (d, *J* = 7.4 Hz, 2H), 7.38-7.30 (m, 3H),7.07 (d, *J* = 8.7 Hz, 2H), 6.93 (d, *J* = 8.9 Hz, 2H), 4.15 (q, *J* = 7.0 Hz, 2H), 4.08 (q, *J* = 7.0 Hz, 2H), 3.80 (s, 3H), 1.49 (t, *J* = 6.9 Hz, 3H), 1.44 (t, *J* = 7.0 Hz, 3H); ^{13}C NMR (101 MHz, $CDCl_3$) δ 159.50, 159.41, 148.56, 145.32, 137.37, 132.96, 132.15, 123.64, 123.25, 123.14, 122.87, 121.90, 119.03, 114.47, 114.04, 109.36, 63.61, 63.55, 31.46, 14.83; HRMS (ESI^+): *m/z* calcd. for $C_{25}H_{26}N_3O_2$ $[M+H]^+$: 400.2020, found:

400.2015.

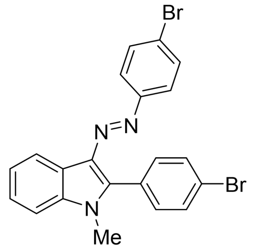

(*E*)-2-(*p*-Bromophenyl)-3-((*p*-bromophenyl)diazenyl)-1-methyl-1*H*-indole (3i): Yellow solid (44.8 mg, yield: 48%), mp: 145.5-147.0 °C. ^{1}H NMR (400 MHz, $CDCl_3$) δ 8.64-8.61 (m, 1H), 7.70 (d, J = 8.5 Hz, 2H), 7.63 (d, J = 8.7 Hz, 2H), 7.56-7.50 (m, 4H), 7.43-7.35 (m, 3H), 3.80 (s, 3H); ^{13}C NMR (101 MHz, $CDCl_3$) δ 152.87, 145.14, 137.52, 132.99, 131.92, 131.33, 128.32, 124.43, 123.73, 123.60, 123.33, 123.30, 122.40, 119.42, 118.56, 109.64, 31.54; HRMS (ESI$^+$): *m/z* calcd. for $C_{21}H_{16}Br_2N_3$ $[M+H]^+$: 467.9705, found: 467.9700.

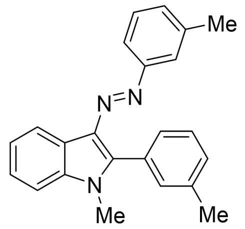

(*E*)-1-Methyl-2-(*m*-tolyl)-3-(*m*-tolyldiazenyl)-1*H*-indole (3j): Yellow semisolid (52.2 mg, yield: 77%). ^{1}H NMR (600 MHz, $CDCl_3$) δ 8.73-8.69 (m, 1H), 7.65 (s, 1H), 7.62 (dt, J = 7.9, 1.3 Hz, 1H), 7.52 (s, 1H), 7.50-7.44 (m, 2H), 7.44-7.41 (m, 1H), 7.41-7.36 (m, 2H), 7.36-7.31 (m, 2H), 7.15 (dt, J = 7.4, 1.9 Hz, 1H), 3.82 (s, 3H), 2.50 (s, 3H), 2.43 (s, 3H); ^{13}C NMR (151 MHz, $CDCl_3$) δ 154.33, 146.25, 138.42, 137.61, 137.44, 132.59, 132.39, 129.67, 129.53, 129.22, 128.81, 128.57, 127.89, 123.94, 123.32, 123.20, 122.72, 118.91, 118.84, 109.48, 31.55, 21.52, 21.43; HRMS (ESI$^+$): *m/z* calcd. for $C_{23}H_{22}N_3$ $[M+H]^+$: 340.1808, found: 340.1806.

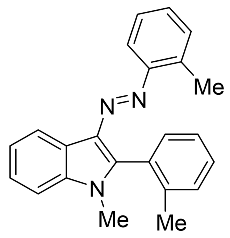

(*E*)-1-Methyl-2-(*o*-tolyl)-3-(*o*-tolyldiazenyl)-1*H*-indole (3k): Yellow semisolid (27.2 mg, yield: 40%). ^{1}H NMR (400 MHz, $CDCl_3$) δ 8.64-8.58 (m, 1H), 7.49-7.33 (m, 8H), 7.29 (d, J = 7.4 Hz, 1H), 7.20 (t, J = 7.3 Hz, 1H), 7.13 (t, J = 7.4 Hz, 1H), 3.63 (s, 3H), 2.78 (s, 3H), 2.27 (s, 3H); ^{13}C NMR (101 MHz, $CDCl_3$) δ 152.09, 146.17,

138.51, 137.14, 136.22, 133.62, 131.61, 130.78, 130.14, 129.88, 129.28, 128.45, 126.14, 125.40, 123.67, 123.21, 123.02, 118.85, 114.84, 109.45, 30.68, 20.42, 18.20; HRMS (ESI$^+$): *m/z* calcd. for $C_{23}H_{22}N_3$ [M+H]$^+$: 340.1808, found: 340.1807.

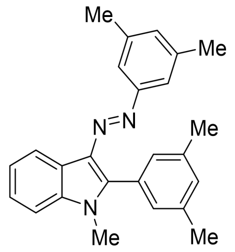

(*E*)-2-(3,5-Dimethylphenyl)-3-((3,5-dimethylphenyl)diazenyl)-1-methyl-1*H*-indole (3l): Yellowish solid (40.4 mg, yield: 55%), mp: 98.0-99.5 °C. ^{1}H NMR (400 MHz, $CDCl_3$) δ 8.72-8.66 (m, 1H), 7.46 (s, 2H), 7.43-7.35 (m, 3H), 7.33 (s, 2H), 7.17 (s, 1H), 6.98 (s, 1H), 3.84 (s, 3H), 2.46 (s, 6H), 2.39 (s, 6H); ^{13}C NMR (101 MHz, $CDCl_3$) δ 154.48, 146.24, 138.18, 137.45, 132.48, 130.57, 130.08, 129.56, 129.39, 123.85, 123.30, 123.09, 119.80, 118.86, 109.44, 31.58, 21.38, 21.31; HRMS (ESI$^+$): *m/z* calcd. for $C_{25}H_{26}N_3$ [M+H]$^+$: 368.2121, found: 368.2117.

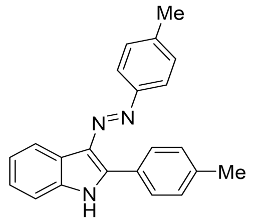

(*E*)-2-(*p*-Tolyl)-3-(*p*-tolyldiazenyl)-1*H*-indole (3m): Yellow semisolid (37.8 mg, yield: 58%). ^{1}H NMR (400 MHz, $CDCl_3$) δ 8.69-8.62 (m, 1H), 8.51 (s, 1H), 7.93 (d, *J* = 7.9 Hz, 2H), 7.81 (d, *J* = 8.0 Hz, 2H), 7.42-7.38 (m, 1H), 7.35 (d, *J* = 7.9 Hz, 2H), 7.33-7.27 (m, 4H), 2.45 (s, 3H), 2.43 (s, 3H); ^{13}C NMR (101 MHz, $CDCl_3$) δ 152.30, 141.34, 139.19, 138.99, 135.35, 132.13, 129.56, 129.50, 129.03, 128.06, 124.28, 123.59, 123.09, 121.99, 120.14, 110.77, 21.37; HRMS (ESI$^+$): *m/z* calcd. for $C_{22}H_{20}N_3$ [M+H]$^+$: 326.1652, found:326.1649.

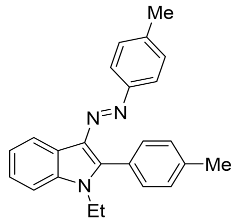

(*E*)-1-Ethyl-2-(*p*-tolyl)-3-(*p*-tolyldiazenyl)-1*H*-indole (3n): Yellow semisolid (55.8 mg, yield: 79%). ^{1}H NMR (600 MHz, $CDCl_3$) δ 8.73-8.70 (m, 1H), 7.70 (dt, *J* = 8.3, 2.4 Hz, 2H), 7.55 (dt, *J* = 8.0, 2.2 Hz, 2H), 7.45 (dd, *J* = 5.7, 1.8 Hz, 1H), 7.39-7.34

(m, 4H), 7.23 (dd, *J* = 8.6, 0.8 Hz, 2H), 4.28 (q, *J* = 7.2 Hz, 2H), 2.50 (s, 3H), 2.40 (s, 3H), 1.41 (t, *J* = 7.1 Hz, 3H); ^{13}C NMR (151 MHz, $CDCl_3$) δ 152.33, 145.70, 138.75, 138.39, 136.20, 132.70, 131.18, 129.35, 128.78, 127.03, 123.71, 123.34, 122.94, 121.75, 119.26, 109.73, 39.10, 21.40, 21.28, 15.17; HRMS (ESI$^+$): *m/z* calcd. for $C_{24}H_{24}N_3$ [M+H]$^+$: 354.1965, found:354.1961.

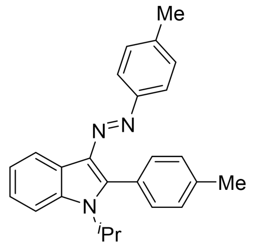

(*E*)-1-Isopropyl-2-(*p*-tolyl)-3-(*p*-tolyldiazenyl)-1*H*-indole (3o): Yellow solid (50.7 mg, yield: 69%), mp: 142.0-143.5 °C. ^{1}H NMR (600 MHz, $CDCl_3$) δ 8.73-8.69 (m, 1H), 7.66-7.62 (m, 3H), 7.48 (dd, *J* = 6.1, 1.8 Hz, 2H), 7.35 (d, *J* = 7.6 Hz, 2H), 7.33-7.28 (m, 2H), 7.20 (d, *J* = 7.6 Hz, 2H), 4.83 (hept, *J* =7.1 Hz, 1H), 2.49 (s, 3H), 2.38 (s, 3H), 1.68 (d, *J* = 7.1 Hz, 6H); ^{13}C NMR (151 MHz, $CDCl_3$) δ 152.39, 146.19, 138.76, 138.37, 134.83, 132.40, 131.29, 129.36, 128.75, 127.58, 123.49, 123.20, 122.65, 121.74, 120.12, 112.39, 48.66, 21.48, 21.46, 21.31; HRMS (ESI$^+$): *m/z* calcd. for $C_{25}H_{26}N_3$ [M+H]$^+$: 368.2121, found: 368.2119.

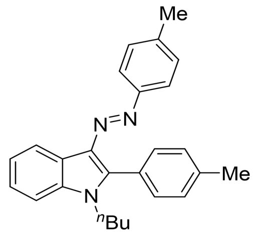

(*E*)-1-Butyl-2-(*p*-tolyl)-3-(*p*-tolyldiazenyl)-1*H*-indole (3p): Yellow semisolid (60.2 mg, yield: 79%). ^{1}H NMR (600 MHz, $CDCl_3$) δ 8.73-8.69 (m, 1H), 7.70 (dt, *J* = 8.3, 2.4 Hz, 2H), 7.54 (dt, *J* = 8.1, 2.0 Hz, 2H), 7.46-7.42 (m, 1H), 7.39-7.34 (m, 4H), 7.21 (dd, *J* = 8.6, 0.78 Hz, 2H), 4.24 (t, *J* = 7.6 Hz, 2H), 2.50 (s, 3H), 2.40 (s, 3H), 1.80-1.73 (m, 2H), 1.28-1.22 (m, 2H), 0.85 (t, *J* = 7.3 Hz, 3H); ^{13}C NMR (151 MHz, $CDCl_3$) δ 152.36, 146.02, 138.72, 138.41, 136.52, 132.66, 131.30, 129.77, 129.37, 128.77, 127.15, 123.65, 123.31, 122.94, 119.21, 117.86, 109.95, 44.06, 31.79, 21.45, 21.31, 19.93, 13.56; HRMS (ESI$^+$): *m/z* calcd. for $C_{26}H_{28}N_3$ [M+H]$^+$: 382.2278, found:382.2275.

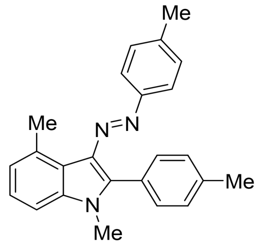

(*E*)-1,4-Dimethyl-2-(*p*-tolyl)-3-(*p*-tolyldiazenyl)-1*H*-indole (3r): Yellow semisolid (49.6 mg, yield: 70%). ^{1}H NMR (600 MHz, $CDCl_3$) δ 7.67 (dd, *J* = 8.1 Hz, 2H), 7.47 (d, *J* = 7.9 Hz, 2H), 7.36 (d, *J* = 8.0 Hz, 2H), 7.33 (t, *J* = 8.0 Hz, 1H), 7.29 (d, *J* = 7.1 Hz, 2H), 7.28 (d, *J* = 7.3 Hz, 1H), 7.21 (dd, *J* = 7.1, 0.6 Hz, 1H), 3.71 (s, 3H), 3.00 (s, 3H), 2.54 (s, 3H), 2.46 (s, 3H); ^{13}C NMR (151 MHz, $CDCl_3$) δ 151.68, 138.64, 138.34, 138.24, 137.50, 133.97, 132.37, 131.27, 129.36, 128.46, 127.95, 124.25, 123.18, 121.71, 120.85, 107.24, 31.01, 23.39, 21.37, 21.25; HRMS (ESI$^+$): *m/z* calcd. for $C_{24}H_{24}N_3$ $[M+H]^+$: 354.1965, found: 354.1962.

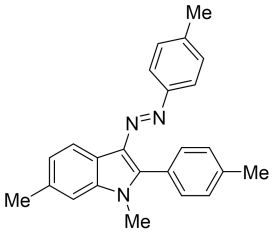

(*E*)-1,6-Dimethyl-2-(*p*-tolyl)-3-(*p*-tolyldiazenyl)-1*H*-indole (3s): Yellow solid (55.7 mg, yield: 79%), mp: 116.7-118.2 °C. ^{1}H NMR (600 MHz, $CDCl_3$) δ 8.54 (dd, *J* = 7.3, 1.3 Hz, 1H), 7.69 (dt, *J* = 8.2, 2.3 Hz, 2H), 7.52 (dt, *J* = 8.0, 2.2 Hz, 2H), 7.32 (d, *J* = 7.8 Hz, 2H), 7.21 (dd, *J* = 6.1, 1.1 Hz, 2H), 7.16 (d, *J* = 7.4 Hz, 2H), 3.72 (s, 3H), 2.53 (s, 3H), 2.46 (s, 3H), 2.38 (s, 3H); ^{13}C NMR (151 MHz, $CDCl_3$) δ 152.39, 145.54, 138.64, 138.29, 137.90, 133.77, 132.51, 131.50, 129.36, 128.69, 126.84, 124.55, 122.92, 121.76, 116.80, 109.52, 31.40, 21.98, 21.41, 21.30; HRMS (ESI$^+$): *m/z* calcd. for $C_{24}H_{24}N_3$ $[M+H]^+$: 354.1965, found: 354.1962.

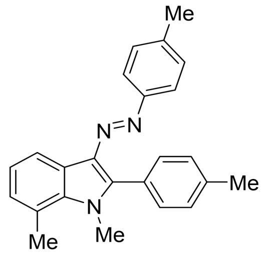

(*E*)-1,7-Dimethyl-2-(*p*-tolyl)-3-(*p*-tolyldiazenyl)-1*H*-indole (3t): Yellow solid (44.5 mg, yield: 63%), mp: 175.5-176.8 °C. ^{1}H NMR (400 MHz, $CDCl_3$) δ 8.59 (d, *J* = 7.2 Hz, 1H), 7.67 (d, *J* = 8.2 Hz, 2H), 7.53 (d, *J* = 8.1 Hz, 2H), 7.35 (d, *J* = 7.9 Hz, 2H), 7.21 (d, *J* = 7.8 Hz, 3H), 7.07 (d, *J* = 7.2 Hz, 1H), 4.00 (s, 3H), 2.85 (s, 3H), 2.48 (s,

3H), 2.38 (s, 3H); ^{13}C NMR (101 MHz, CDCl$_3$) δ 152.37, 147.32, 138.76, 138.39, 136.73, 132.30, 131.75, 129.37, 128.68, 127.08, 126.94, 123.18, 121.76, 121.20, 121.17, 119.95, 35.25, 21.43, 21.30, 20.52; HRMS (ESI$^+$): *m/z* calcd. for $C_{24}H_{24}N_3$ $[M+H]^+$: 354.1965, found: 354.1962.

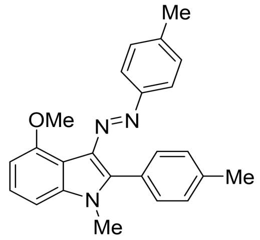

(*E*)-4-Methoxy-1-methyl-2-(*p*-tolyl)-3-(*p*-tolyldiazenyl)-1*H*-indole (3u): Yellow semisolid (33.5 mg, yield: 45%). ^{1}H NMR (400 MHz, CDCl$_3$) δ 7.61 (d, *J* = 8.3 Hz, 2H), 7.41 (d, *J* = 8.0 Hz, 2H), 7.29 (d, *J* = 8.1 Hz, 3H), 7.20 (d, *J* = 8.1 Hz, 2H), 7.03 (d, *J* = 8.2 Hz, 1H), 6.78 (d, *J* = 7.9 Hz, 1H), 3.99 (s, 3H), 3.72 (s, 3H), 2.46 (s, 3H), 2.37 (s, 3H); ^{13}C NMR (101 MHz, CDCl$_3$) δ 154.33, 152.02, 140.00, 139.29, 138.72, 138.38, 132.99, 131.49, 129.32, 128.55, 127.60, 124.26, 121.98, 111.06, 104.78, 103.04, 55.68, 30.94, 21.42, 21.32; HRMS (ESI$^+$): *m/z* calcd. for $C_{24}H_{23}N_3ONa$ $[M+Na]^+$: 392.1733, found:392.1733.

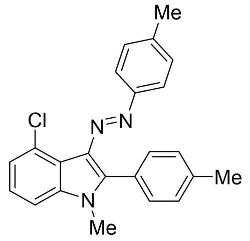

(*E*)-4-Chloro-1-methyl-2-(*p*-tolyl)-3-(*p*-tolyldiazenyl)-1*H*-indole (3v): Yellow semisolid (21.8 mg, yield: 29%). ^{1}H NMR (400 MHz, CDCl$_3$) δ 7.61 (d, *J* = 8.2 Hz, 2H), 7.37 (d, *J* = 8.1 Hz, 2H), 7.34 (dd, *J* = 7.5, 1.1 Hz, 1H), 7.29 (dt, *J* = 8.4, 1.1 Hz, 3H), 7.22 (d, *J* = 8.1 Hz, 3H), 3.66 (s, 3H), 2.47 (s, 3H), 2.39 (s, 3H); ^{13}C NMR (101 MHz, CDCl$_3$) δ 151.48, 139.38, 138.64, 138.26, 136.21, 131.98, 131.16, 129.39, 128.64, 127.40, 126.43, 123.51, 123.22, 122.07, 120.09, 108.34, 31.16, 21.42, 21.34; HRMS (ESI$^+$): *m/z* calcd. for $C_{23}H_{20}ClN_3Na$ $[M+Na]^+$: 396.1238, found: 396.1233.

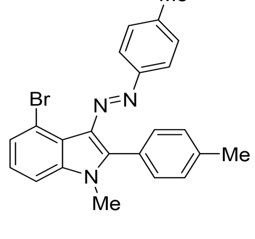

(*E*)-4-Bromo-1-methyl-2-(*p*-tolyl)-3-(*p*-tolyldiazenyl)-1*H*-indole (3w): Yellow

semisolid (45.8 mg, yield: 55%). ^{1}H NMR (600 MHz, $CDCl_3$) δ 7.61 (dt, *J* = 8.3, 2.4 Hz, 2H), 7.53 (dd, *J* = 7.6, 0.8 Hz, 1H), 7.36-7.33 (m, 3H), 7.27 (dd, *J* = 8.5, 1.5 Hz, 2H), 7.20 (d, *J* = 7.8 Hz, 2H), 7.16 (t, *J* = 7.7 Hz, 1H), 3.65 (s, 3H), 2.46 (s, 3H), 2.38 (s, 3H); ^{13}C NMR (151 MHz, $CDCl_3$) δ 151.47, 139.45, 138.62, 137.94, 134.36, 131.71, 131.09, 129.32, 128.14, 127.61, 126.75, 123.42, 122.47, 122.19, 113.65, 108.93, 31.02, 21.45, 21.37; HRMS (ESI$^+$): *m/z* calcd. for $C_{23}H_{21}BrN_3$ $[M+H]^+$: 418.0913, found: 418.0909.

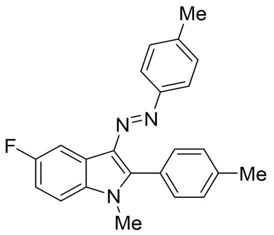

(*E*)-5-Fluoro-1-methyl-2-(*p*-tolyl)-3-(*p*-tolyldiazenyl)-1*H*-indole (3x): Yellow semisolid (39.5 mg, yield: 55%). ^{1}H NMR (400 MHz, $CDCl_3$) δ 8.37 (dd, *J* = 9.8, 2.6 Hz, 1H), 7.69 (d, *J* = 8.4 Hz, 2H), 7.54 (d, *J* = 8.1 Hz, 2H), 7.36 (d, *J* = 7.9 Hz, 2H), 7.31 (dd, *J* = 8.8, 4.3 Hz, 1H), 7.23 (d, *J* = 8.0 Hz, 2H), 7.09 (td, *J* = 8.9, 2.6 Hz, 1H), 3.79 (s, 3H), 2.49 (s, 3H), 2.39 (s, 3H); ^{13}C NMR (101 MHz, $CDCl_3$) δ 161.16, 158.81, 152.09, 146.96, 139.02, 138.67, 133.86, 132.16 (d, *J* = 4.0 Hz), 131.39, 129.41, 128.77, 126.41, 121.81, 119.06 (d, *J* = 11.1 Hz), 111.59 (d, *J* = 34.3 Hz), 110.03 (d, *J* = 37.4 Hz), 108.53 (d, *J* = 25.3 Hz), 31.57, 21.39, 21.29; HRMS (ESI$^+$): *m/z* calcd. for $C_{23}H_{21}FN_3$ $[M+H]^+$: 358.1714, found: 358.1709.

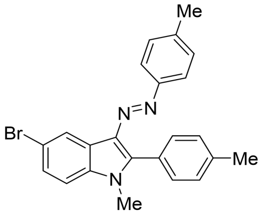

(*E*)-5-Bromo-1-methyl-2-(*p*-tolyl)-3-(*p*-tolyldiazenyl)-1*H*-indole (3y): Yellow solid (35.1 mg, yield: 42%), mp: 173.6-174.9 °C. ^{1}H NMR (600 MHz, $CDCl_3$) δ 8.58 (d, *J* = 8.5Hz, 1H), 7.69 (dd, *J* = 6.4, 1.9 Hz, 2H), 7.54 (d, *J* = 8.0 Hz, 2H), 7.38 (d, *J* = 1.8 Hz, 1H), 7.35 (d, *J* = 7.7 Hz, 2H), 7.30 (dd, *J* = 8.4, 1.8 Hz, 1H), 7.23 (d, *J* = 8.0 Hz, 2H) 3.74 (s, 3H), 2.49 (s, 3H), 2.40 (s, 3H); ^{13}C NMR (151 MHz, $CDCl_3$) δ 152.11, 146.29, 139.08, 138.85, 137.96, 132.09, 131.46, 129.50, 129.43, 128.82, 126.29, 124.20, 123.37, 121.85, 117.46, 109.62, 31.57, 21.44, 21.33; HRMS (ESI$^+$): *m/z* calcd. for $C_{23}H_{21}BrN_3$ $[M+H]^+$: 418.0913, found: 418.0909.

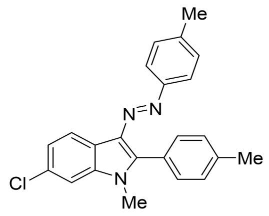

(*E*)-6-Chloro-1-methyl-2-(*p*-tolyl)-3-(*p*-tolyldiazenyl)-1*H*-indole (3z): Yellow solid (38.0 mg, yield: 51%), mp: 149.4-151.2 °C. ^{1}H NMR (600 MHz, $CDCl_3$) δ 7.63 (d, *J* = 8.2 Hz, 2H), 7.54 (d, *J* = 7.7 Hz, 1H), 7.34 (dd, *J* = 7.9, 2.0 Hz, 3H), 7.27 (d, *J* = 7.7 Hz, 2H), 7.21 (d, *J* = 8.0 Hz, 2H), 7.16 (t, *J* = 7.9 Hz, 1H), 3.63 (s, 3H), 2.46 (s, 3H), 2.39 (s, 3H); ^{13}C NMR (101 MHz, $CDCl_3$) δ 152.12, 146.30, 139.10, 138.86, 137.96, 132.10, 131.43, 129.51, 129.44, 128.86, 126.30, 124.21, 123.38, 121.86, 117.47, 109.63, 31.57, 21.43, 21.33; HRMS (ESI^{+}): *m/z* calcd. for $C_{23}H_{21}ClN_3$ $[M+H]^{+}$: 375.1497, found: 375.1459.

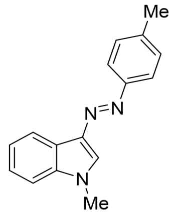

(*E*)-1-Methyl-2-(*p*-tolyl)-3-(*p*-tolyldiazenyl)-1*H*-indole (4a): Yellow solid (4.5 mg, yield: 9%), mp: 110.0-110.9 °C. ^{1}H NMR (400 MHz, $CDCl_3$) δ 8.63-8.60 (m, 1H), 7.83 (dd, *J* = 6.4, 1.9 Hz, 2H), 7.82 (s, 1H), 7.37-7.35 (m, 2H), 7.34-7.32 (m, 2H), 7.32-7.31 (m, 1H), 3.82 (s, 3H), 2.45 (s, 3H); ^{13}C NMR (151 MHz, $CDCl_3$) δ 151.85, 138.92, 137.21, 135.30, 134.13, 129.51, 123.79, 123.07, 122.65, 121.59, 119.50, 109.35, 33.28, 21.31; HRMS (ESI^{+}): *m/z* calcd. for $C_{16}H_{16}N_3$ $[M+H]^{+}$: 250.13387, found: 250.13377.

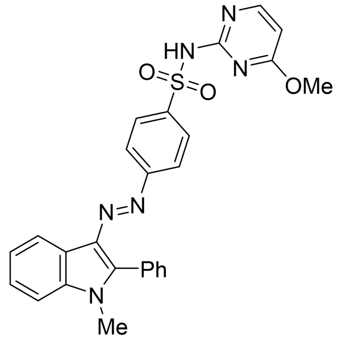

(*E*)-*N*-(6-Methoxypyrimidin-4-yl)-4-((1-methyl-2-phenyl-1*H*-indol-3-yl)diazenyl)-benzeneesulfonamide (3aa): Red solid, (95.0 mg, yield: 95%), mp: 248.6-250.0 °C. ^{1}H NMR (400 MHz, $CDCl_3$) δ 12.27 (s, 1H), 8.51 (d, *J* = 7.6 Hz, 1H), 8.42 (s, 1H),

7.99 (d, *J* = 8.6 Hz, 2H), 7.79-7.74 (m, 4H), 7.70 (d, *J* = 8.0 Hz, 1H), 7.64-7.59 (m, 3H), 7.44-7.35 (m, 2H), 6.36 (d, *J* = 0.5 Hz, 1H), 3.84 (s, 6H). ^{13}C NMR (101 MHz, $CDCl_3$) δ 170.13, 158.62, 156.17, 148.43, 137.50, 132.71, 131.75, 129.67, 128.79, 128.39, 128.33, 124.79, 124.11, 122.67, 121.76, 117.85, 111.19, 91.07, 54.43, 31.97; HRMS (ESI) calcd. for $C_{26}H_{23}N_6O_3S$ ($[M+H]^+$): 499.1547, found: 499.1543. UV– absorption (EtOH, c = 2 × 10^{-5} mol·L^{-1}): λ_{max} = 402 (17450) nm (L·mol^{-1}·cm^{-1}).

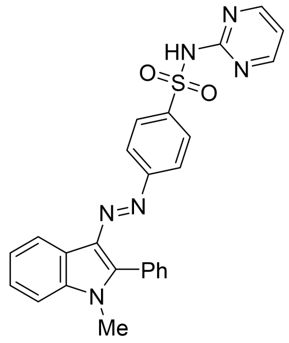

(*E*)-4-((1-Methyl-2-phenyl-1*H*-indol-3-yl)diazenyl)-*N*-(pyrimidin-2-yl)benzene-sulfonamide (3ab): Red solid, (88.4 mg, yield: 94%) mp: 254.9-256.3 °C. ^{1}H NMR (400 MHz, $CDCl_3$) δ 11.88 (s, 1H), 8.50 (t, *J* = 8.8 Hz, 3H), 8.05 (d, *J* = 8.6 Hz, 2H), 7.79-7.72 (m, 4H), 7.68 (d, *J* = 8 Hz, 1H), 7.64-7.56 (m, 3H), 7.44-7.34 (m, 2H), 7.03 (t, *J* = 4.9 Hz, 1H), 3.82 (s, 3H). ^{13}C NMR (101 MHz, $CDCl_3$) δ 158.55, 157.05, 156.21, 148.41, 139.32, 137.52, 132.73, 131.77, 129.69, 129.06, 128.82, 128.41, 124.81, 124.13, 122.71, 121.47, 117.89, 115.97, 111.20, 31.98. HRMS (ESI) calcd. for $C_{25}H_{21}N_6O_2S$ ($[M+H]^+$): 469.1441, found: 469.1438. UV– absorption (EtOH, c = 2 × 10^{-5} mol·L^{-1}): λ_{max} = 402 (25500) nm (L·mol^{-1}·cm^{-1}).

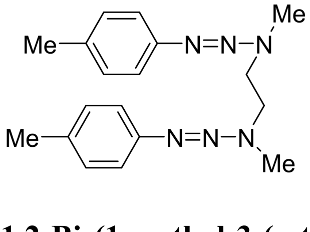

1,2-Bis(1-methyl-3-(*p*-tolyl)triaz-2-en-1-yl)ethane (5a): Yellow solid (2.91 g, 90%), mp: 117.5-118.4 °C. ^{1}H NMR (600 MHz, $CDCl_3$) δ 7.31 (d, *J* = 6.3 Hz, 4H), 7.14 (d, *J* = 7.3 Hz, 4H), 4.04 (s, 4H), 3.26 (s, 6H), 2.35 (s, 6H); ^{13}C NMR (151 MHz, $CDCl_3$) δ 148.48, 135.31, 129.45, 120.49, 53.24, 35.72, 21.09; HRMS (ESI$^+$): *m/z* calcd. for $C_{18}H_{24}N_6Na$ $[M+Na]^+$: 347.1955, found:347.1950.

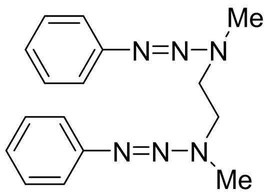

1,2-Bis(1-methyl-3-phenyltriaz-2-en-1-yl)ethane (5b): Yellow solid (2.52 g, 85%), mp: 97.4-99.0 °C. ^{1}H NMR (600 MHz, $CDCl_3$) δ 7.38 (s, 10H), 3.29 (s, 4H), 2.80 (s, 6H); ^{13}C NMR (151 MHz, $CDCl_3$) δ 150.68, 128.84, 125.70, 120.68, 53.69, 35.28; HRMS (ESI^+): *m/z* calcd. for $C_{16}H_{20}N_6Na$ $[M+Na]^+$: 319.1642, found:319.1637.

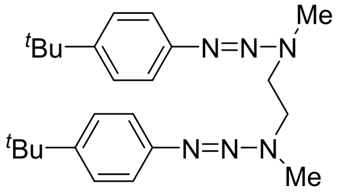

1,2-Bis(3-(4-(*tert*-butyl)phenyl)-1-methyltriaz-2-en-1-yl)ethane (5e): Yellow solid (3.55 g, 87%), mp: 143.5-145.0 °C. ^{1}H NMR (600 MHz, $CDCl_3$) δ 7.40-7.33 (m, 8H), 4.06 (s, 4H), 3.28 (s, 6H), 1.35 (s, 18H); ^{13}C NMR (151 MHz, $CDCl_3$) δ 148.56, 148.31, 125.64, 120.15, 53.23, 35.42, 34.42, 31.42; HRMS (ESI^+): *m/z* calcd. for $C_{24}H_{36}N_6K$ $[M+K]^+$: 447.2633, found:447.2628.

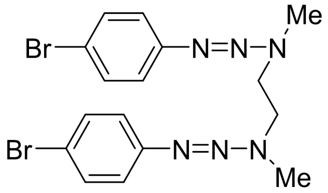

1,2-Bis(3-(4-bromophenyl)-1-methyltriaz-2-en-1-yl)ethane (5i): Yellow solid (4.21 g, 93%), mp: 190.5-191.4 °C. ^{1}H NMR (600 MHz, $CDCl_3$) δ 7.44 (d, J = 6.5 Hz, 4H), 7.27 (d, J = 14.3 Hz, 4H), 4.07 (s, 4H), 3.23 (s, 6H); ^{13}C NMR (151 MHz, $CDCl_3$) δ 149.51, 131.76, 121.95, 118.78, 54.14, 35.12; HRMS (ESI^+): *m/z* calcd. for $C_{16}H_{18}Br_2N_6K$ $[M+K]^+$: 494.9550, found:494.9552.

References

1. Liu YH, Ma X, Wu GX, et, al. The controllable C2 arylation and C3 diazenylation of indoles with aryltriazenes under ambient conditions. New J. Chem. 2019; 43:9255-9259.
