# Supplementary material for: Metal-free molecular editing of indole via tandem reaction: Access to 2-aryl-3-aryldiazenylindole for theranostic applications
Source: iScience. 2025 Aug 8;28(9):113325. doi: 10.1016/j.isci.2025.113325 (PMC12496193; doi:10.1016/j.isci.2025.113325)

Date s2. Copies of NMR Spectra

Fig. S4A: ^{1}H NMR of product **3a** in $CDCl_3$ (600 MHz)

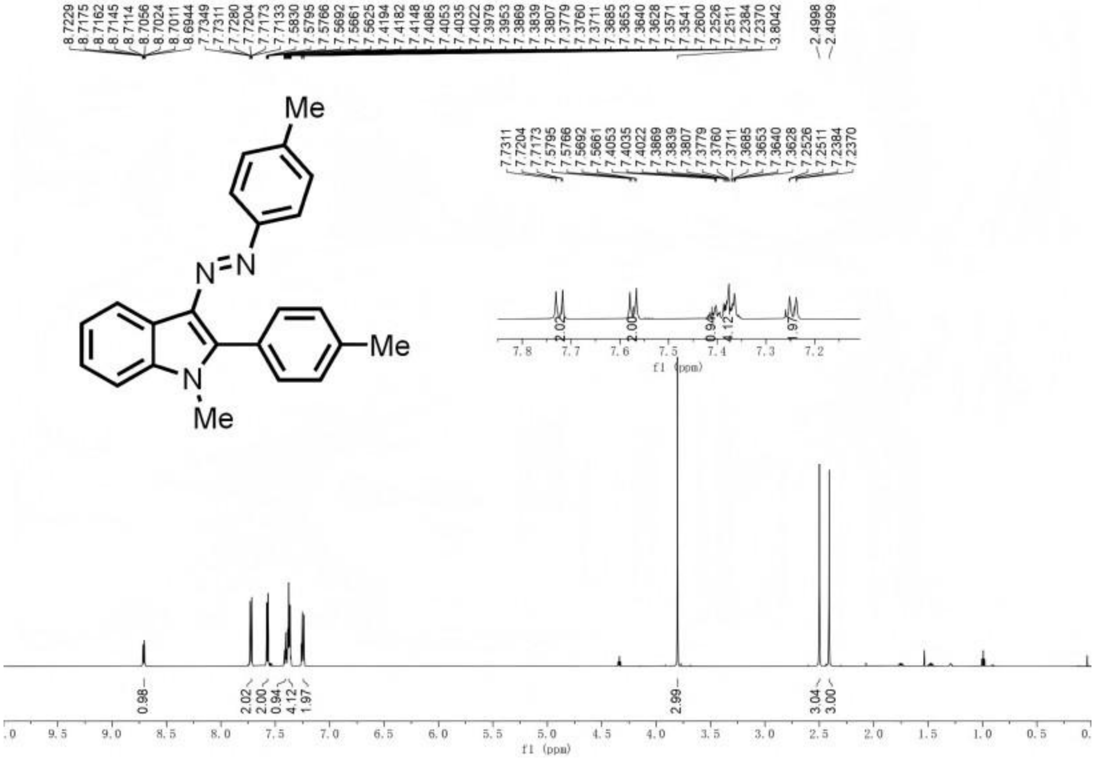

Fig. S4B: ^{13}C NMR of product **3a** in $CDCl_3$ (151 MHz)

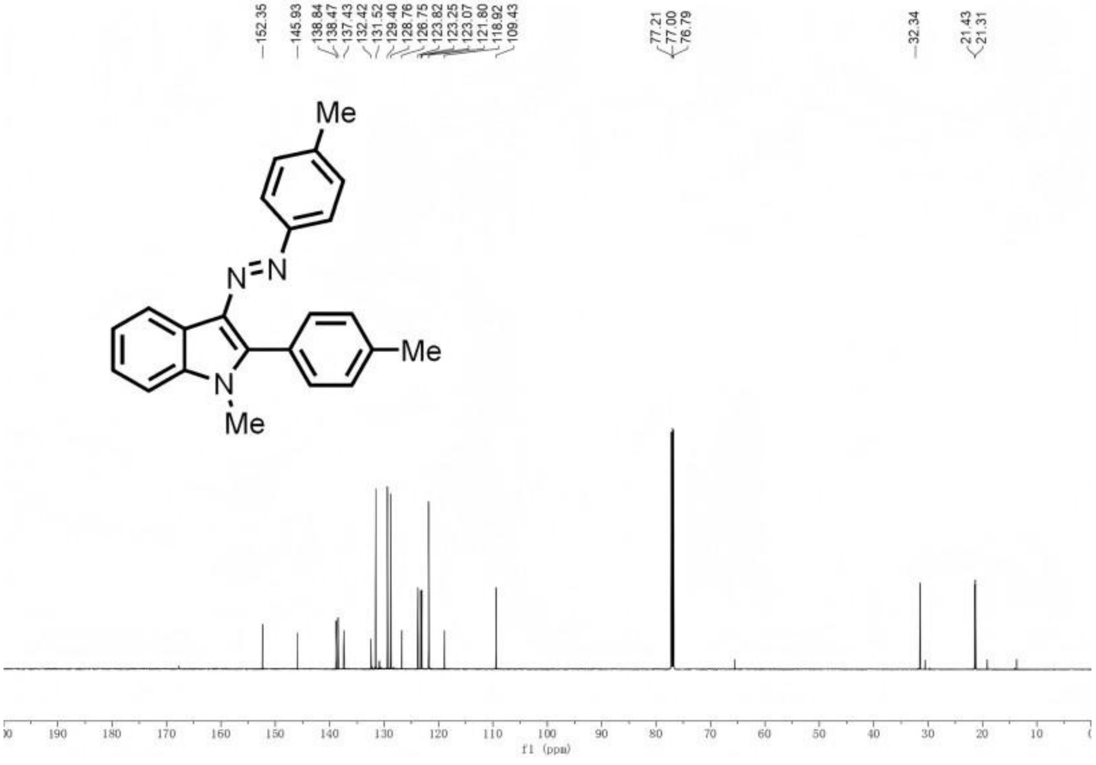

Fig. S5A: ^{1}H NMR of product **3b** in $CDCl_3$ (600 MHz)

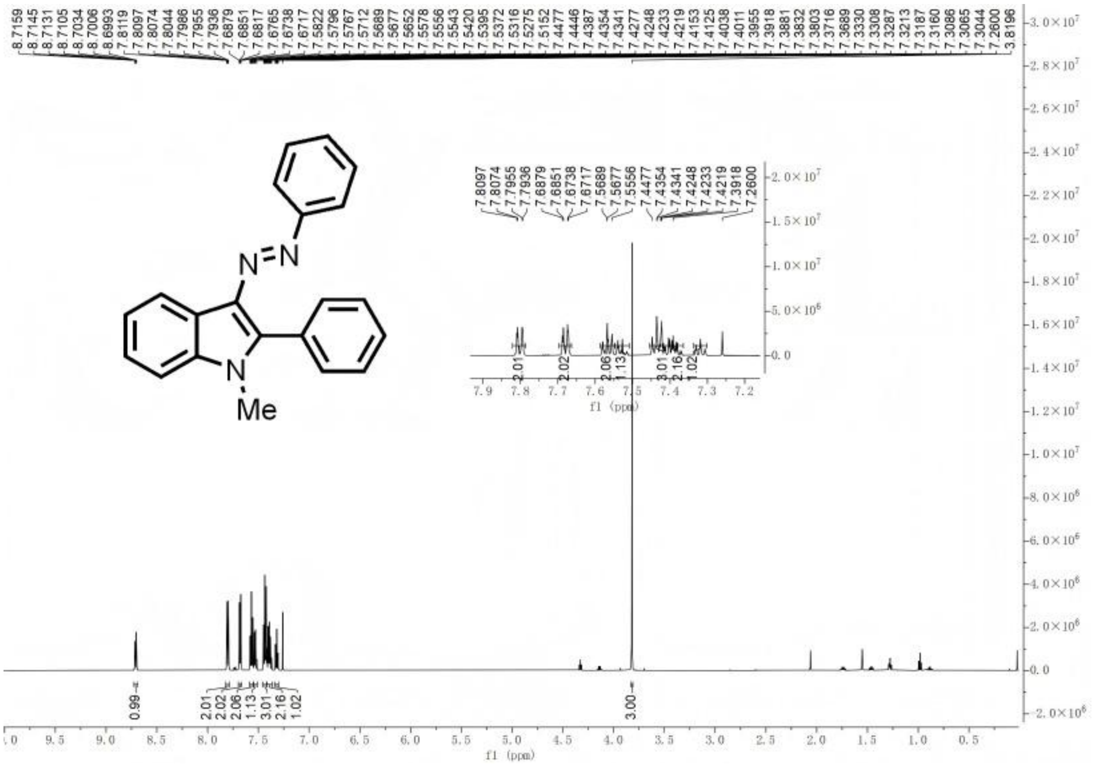

Fig. S5B: ^{13}C NMR of product **3b** in $CDCl_3$ (151 MHz)

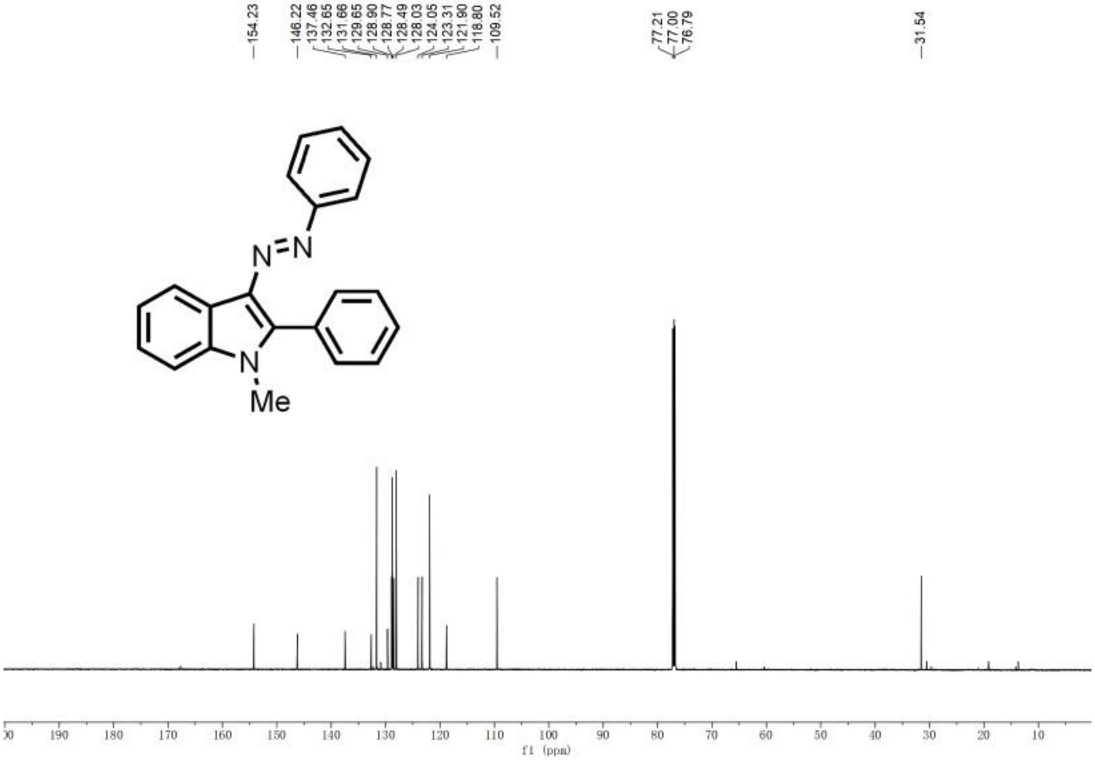

Fig. S6A: ^{1}H NMR of product **3c** in $CDCl_3$ (600 MHz)

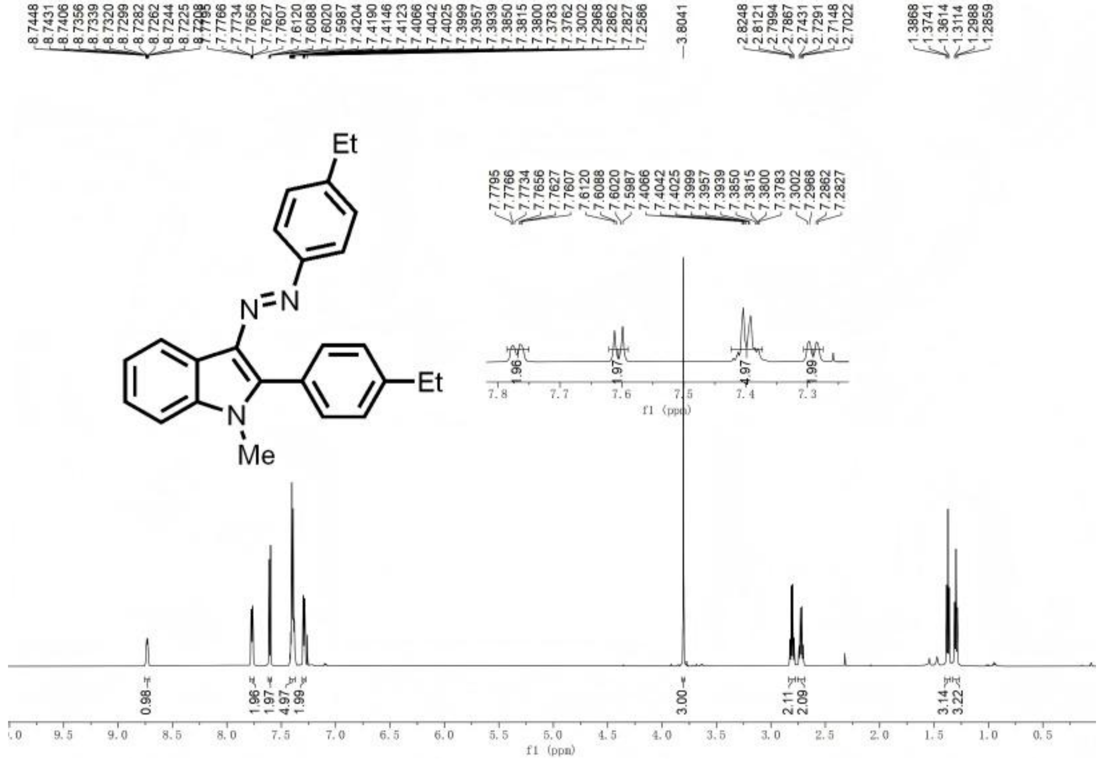

Fig. S6B: ^{13}C NMR of product **3c** in $CDCl_3$ (151 MHz)

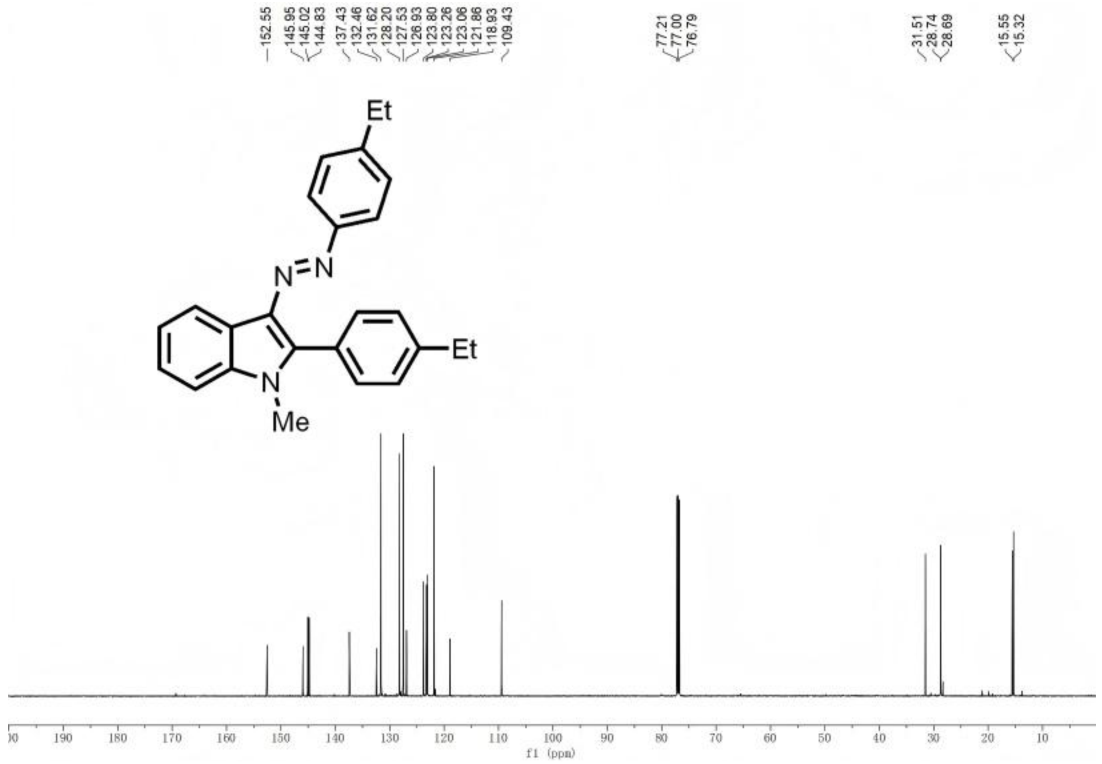

Fig. S7A: ^{1}H NMR of product **3d** in $CDCl_3$ (600 MHz)

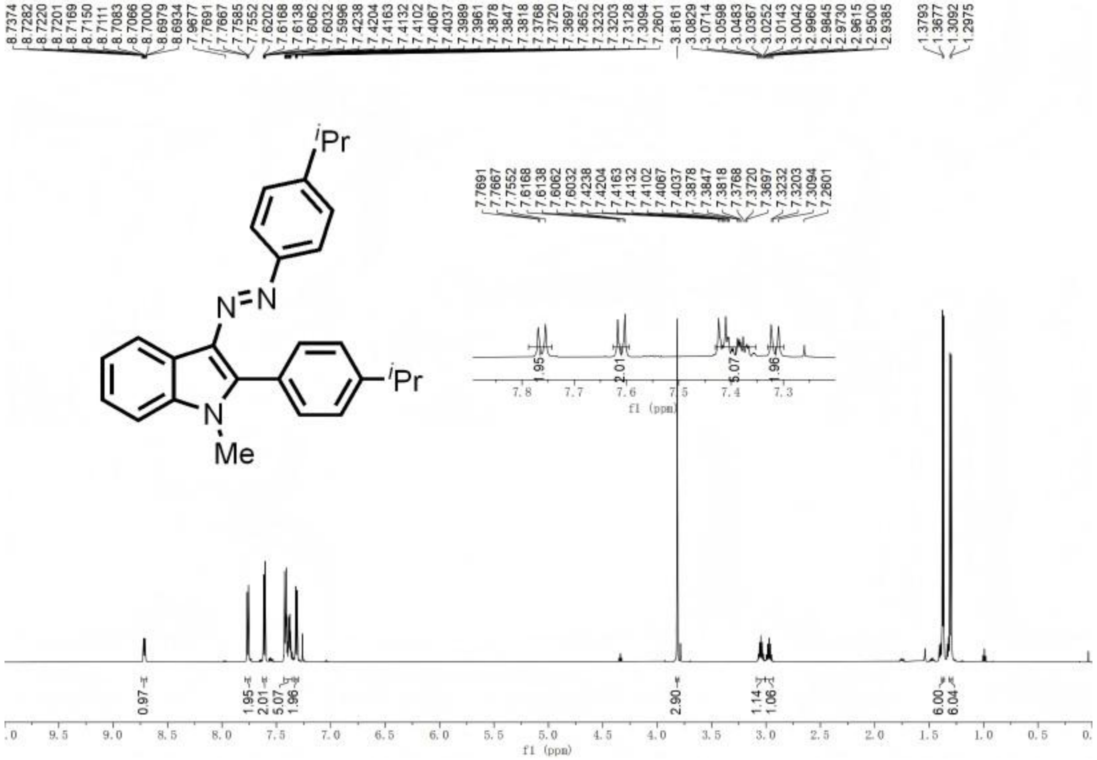

Fig. S7B: ^{13}C NMR of product **3d** in $CDCl_3$ (151 MHz)

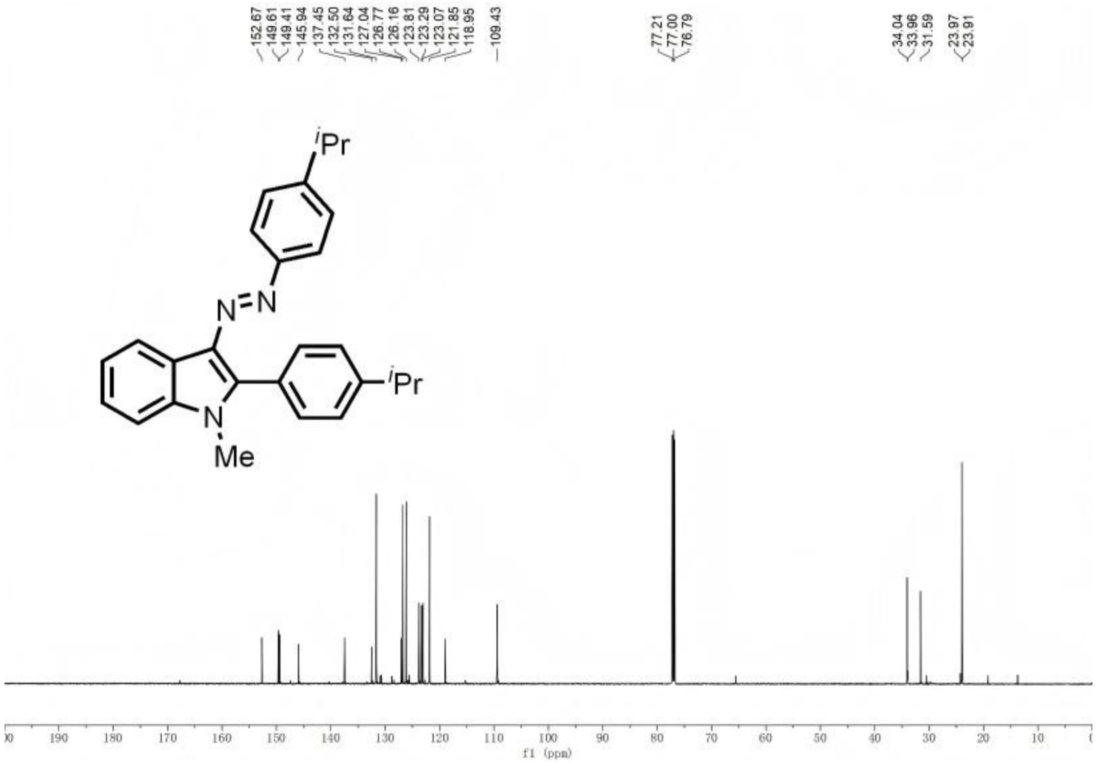

Fig. S8A: ^{1}H NMR of product **3e** in $CDCl_3$ (600 MHz)

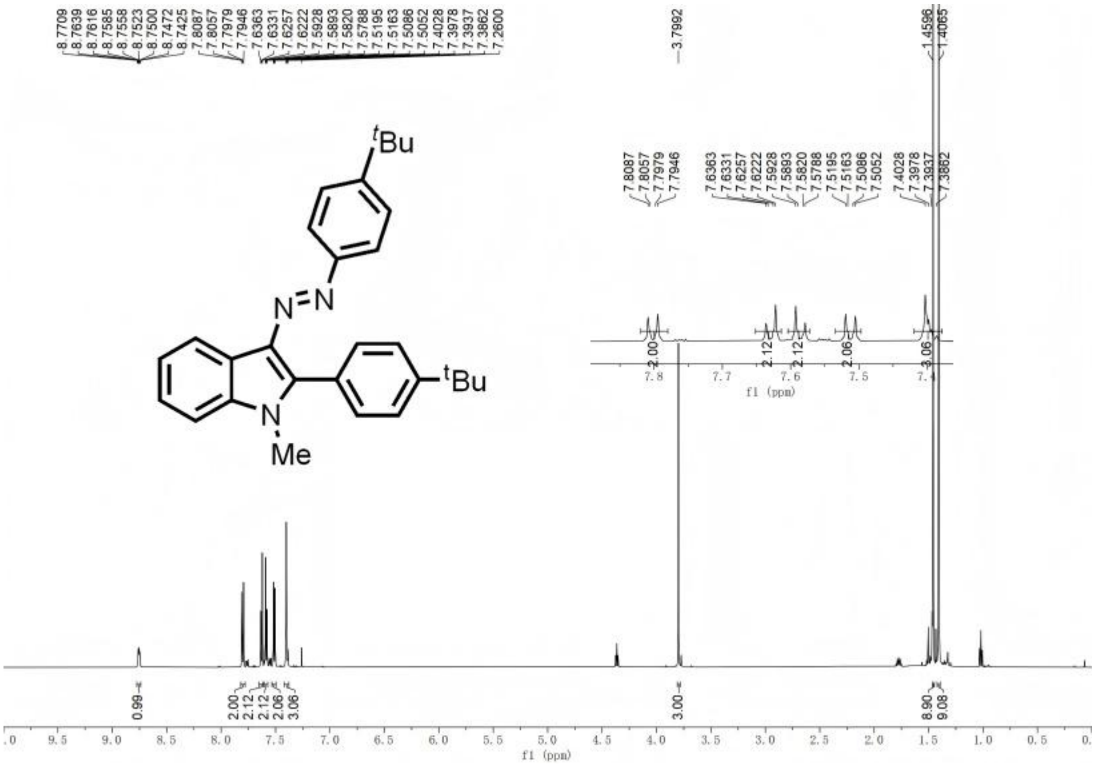

Fig. S8B: ^{13}C NMR of product **3e** in $CDCl_3$ (151 MHz)

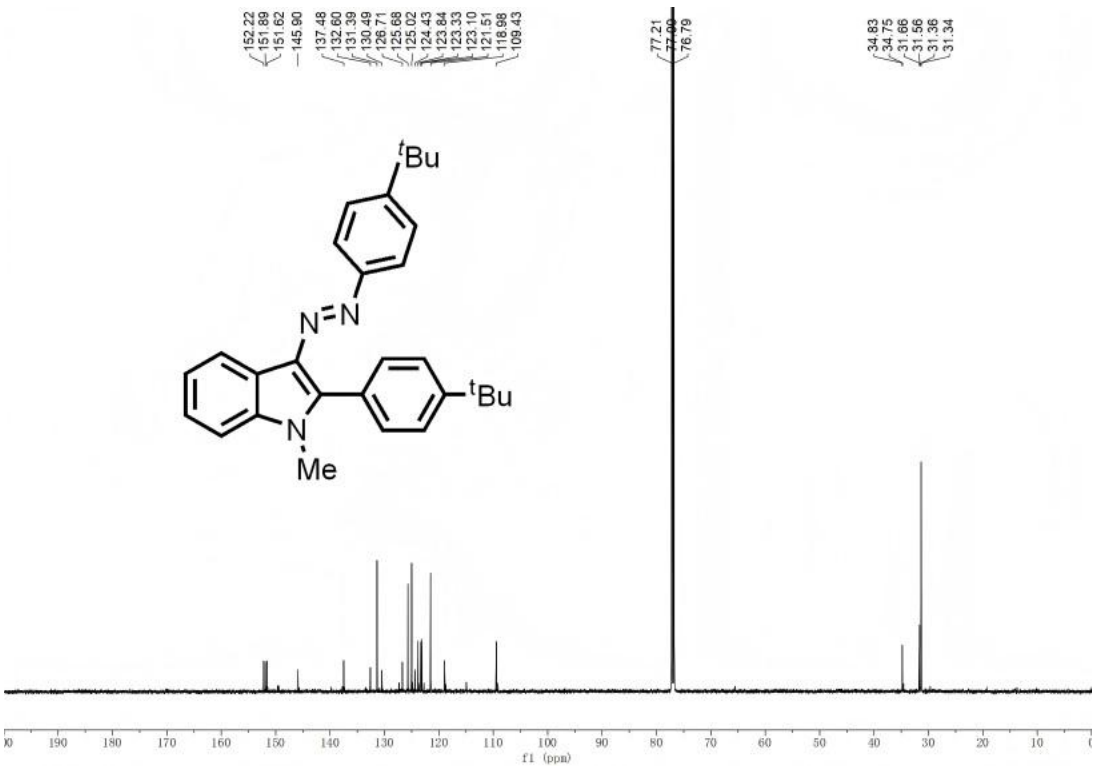

Fig. S9A: ^{1}H NMR of product **3f** in $CDCl_3$ (600 MHz)

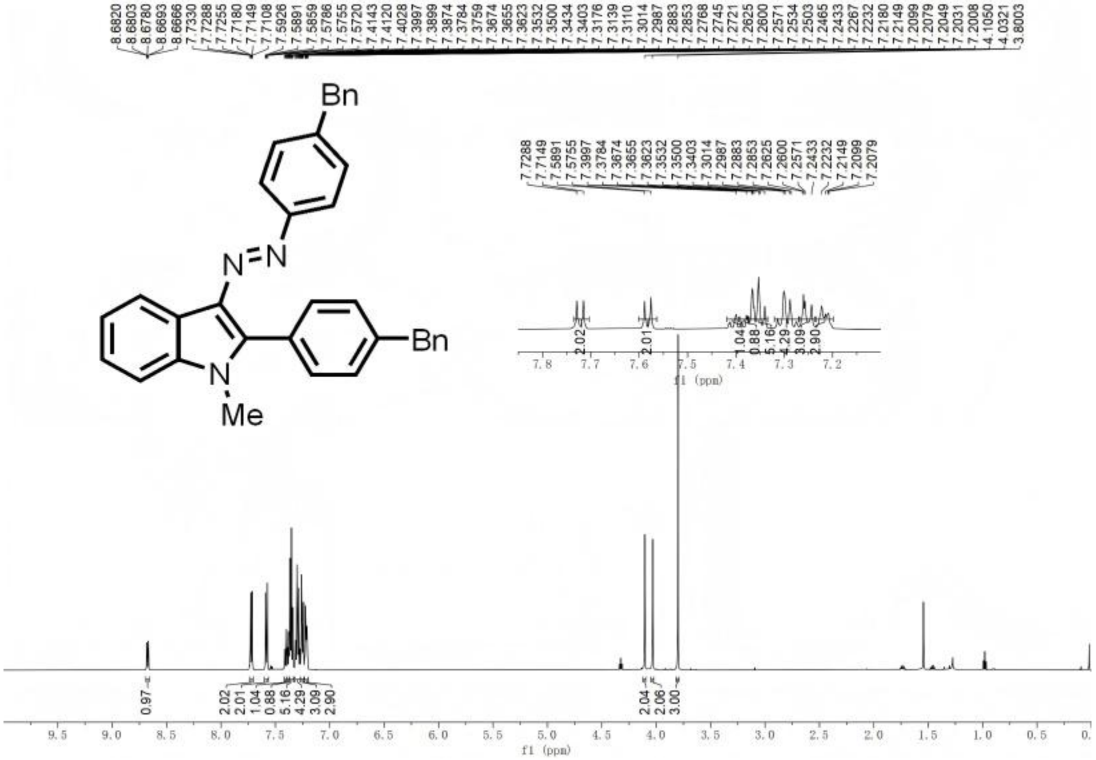

Fig. S9B: ^{13}C NMR of product **3f** in $CDCl_3$ (151 MHz)

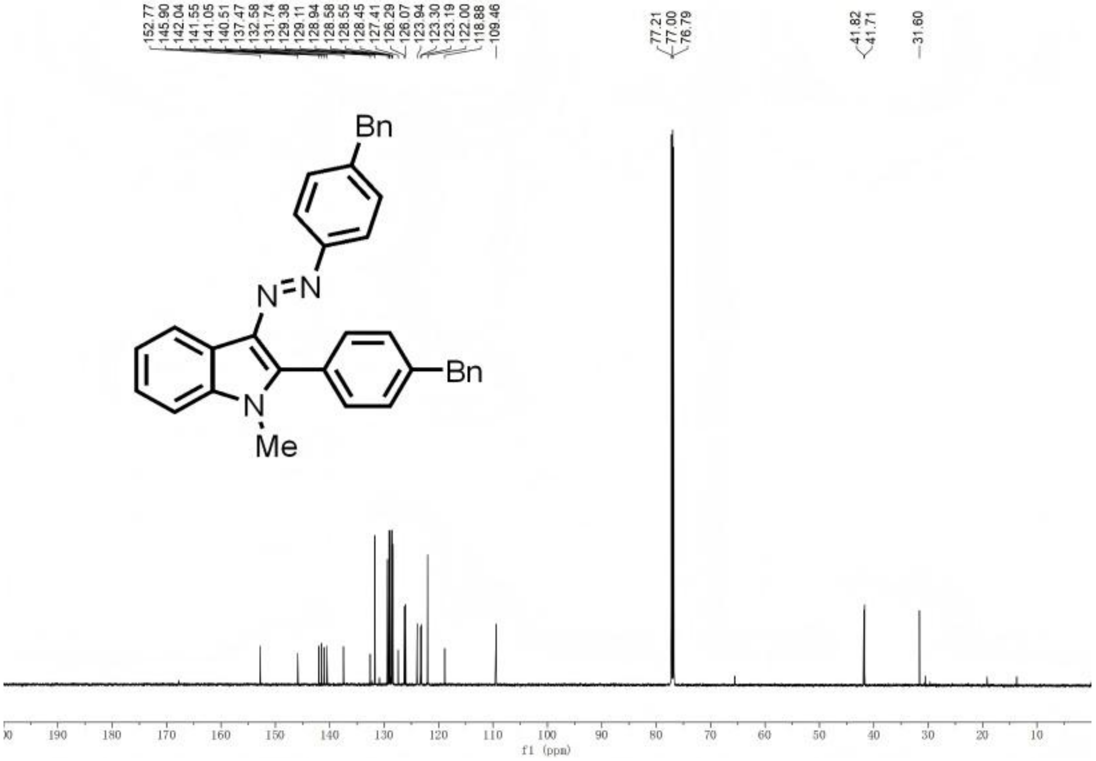

Fig. S10A: ^{1}H NMR of product **3g** in $CDCl_3$ (400 MHz)

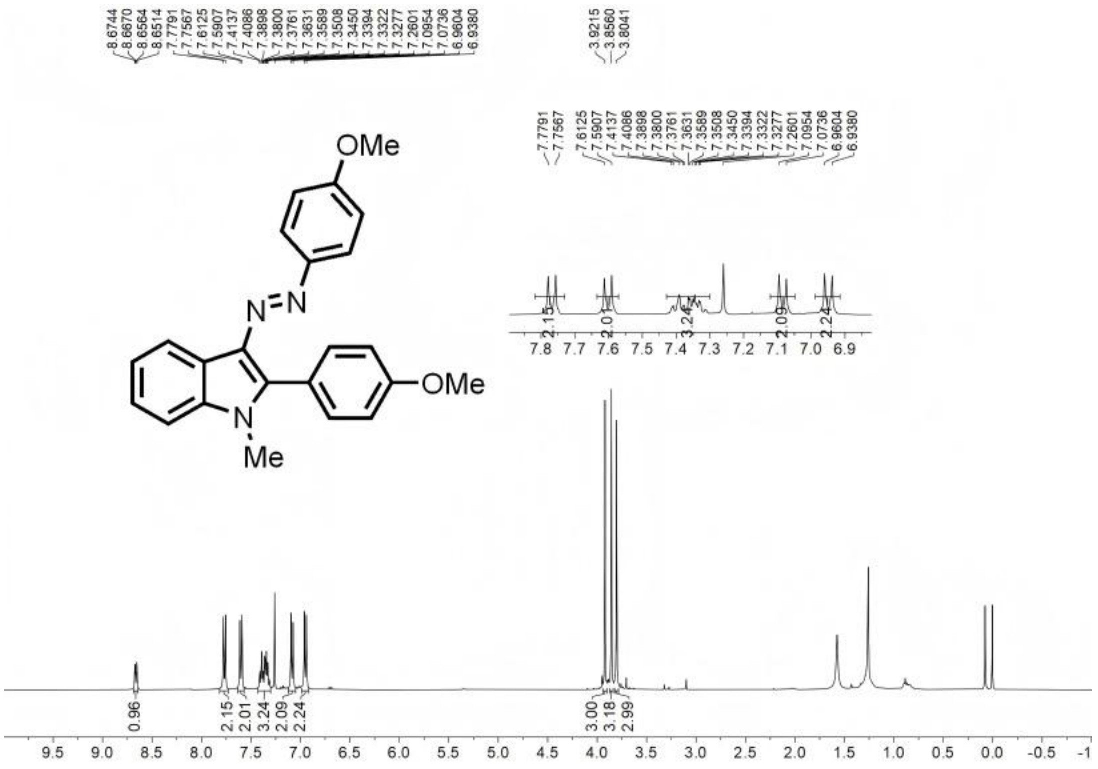

Fig. S10B: ^{13}C NMR of product **3g** in $CDCl_3$ (101 MHz)

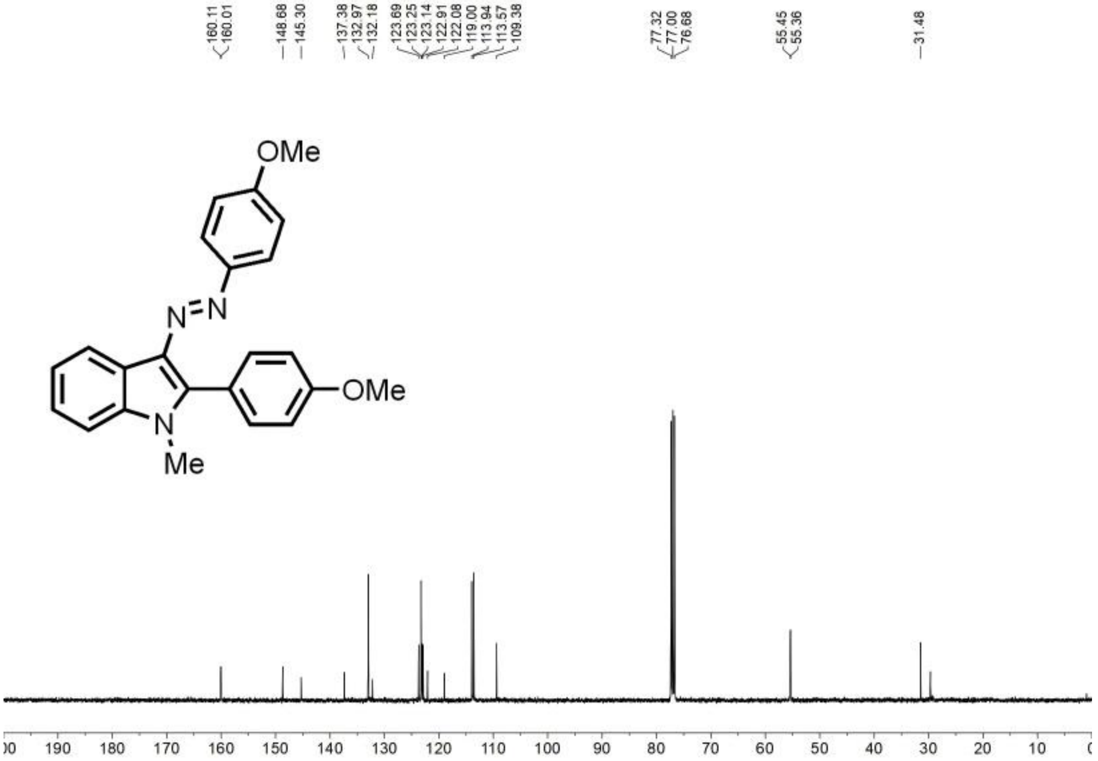

Fig. S11A: ^{1}H NMR of product **3h** in $CDCl_3$ (400 MHz)

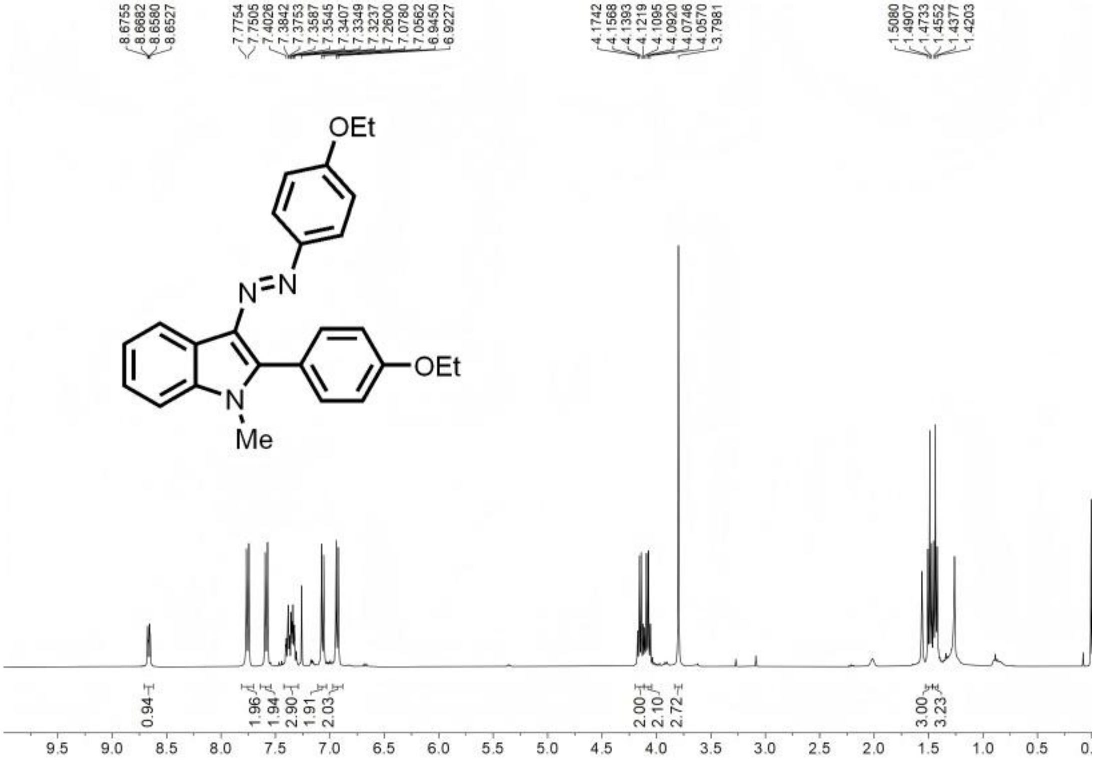

Fig. S11B: ^{13}C NMR of product **3h** in $CDCl_3$ (101 MHz)

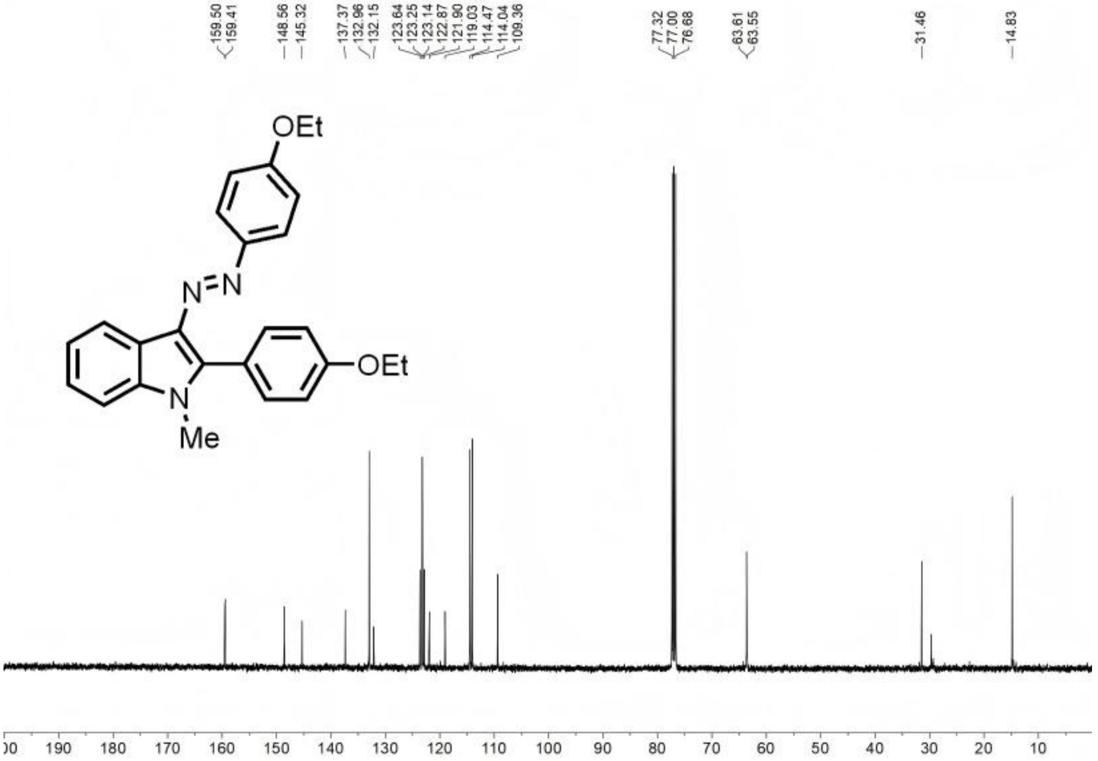

Fig. S12A: ^{1}H NMR of product **3i** in $CDCl_3$ (400 MHz)

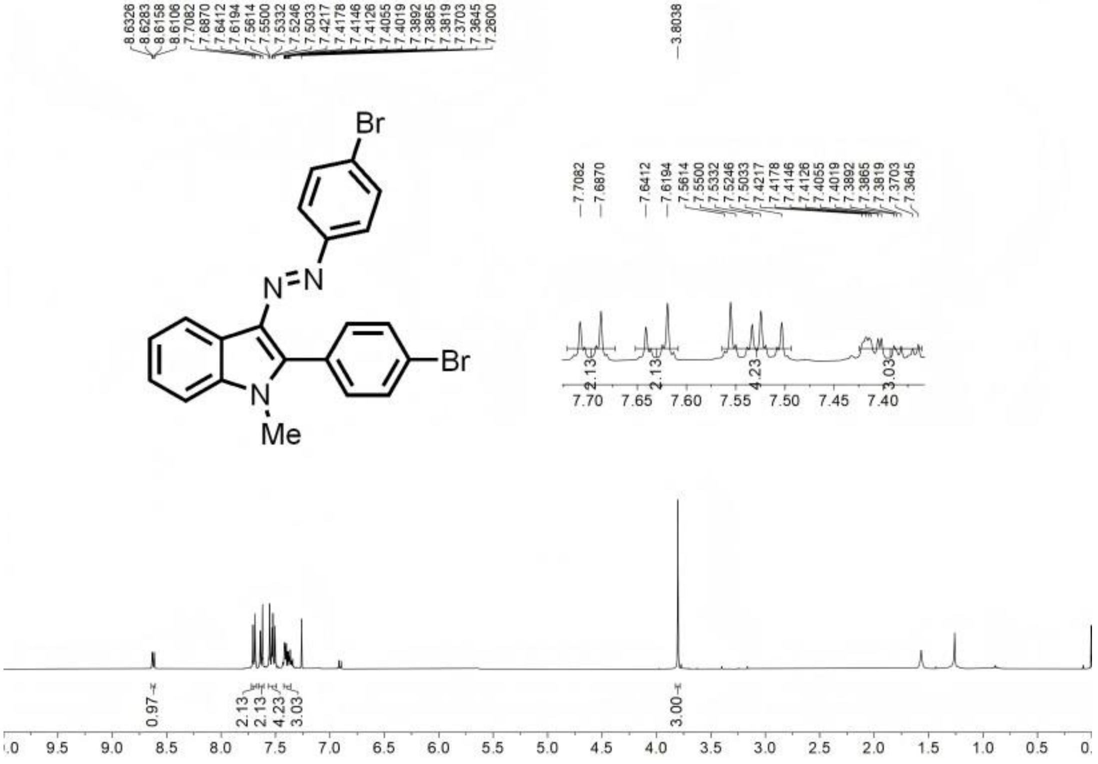

Fig. S12B: ^{13}C NMR of product **3i** in $CDCl_3$ (101 MHz)

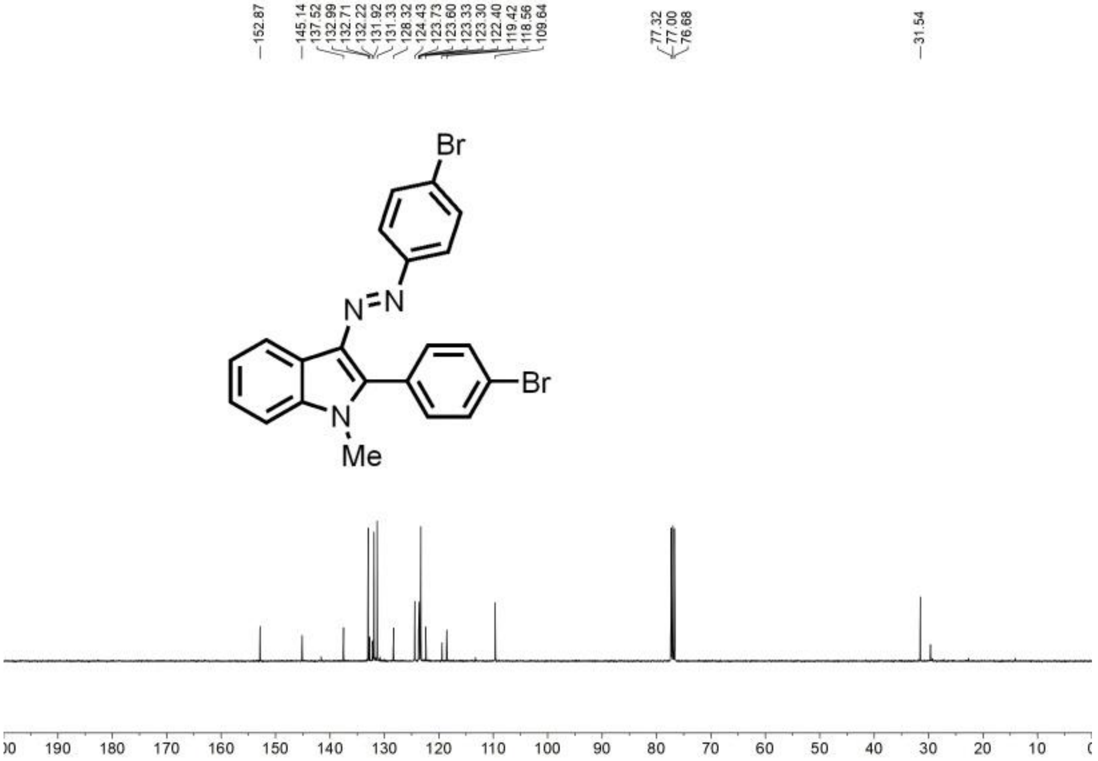

Fig. S13A: ^{1}H NMR of product **3j** in $CDCl_3$ (600 MHz)

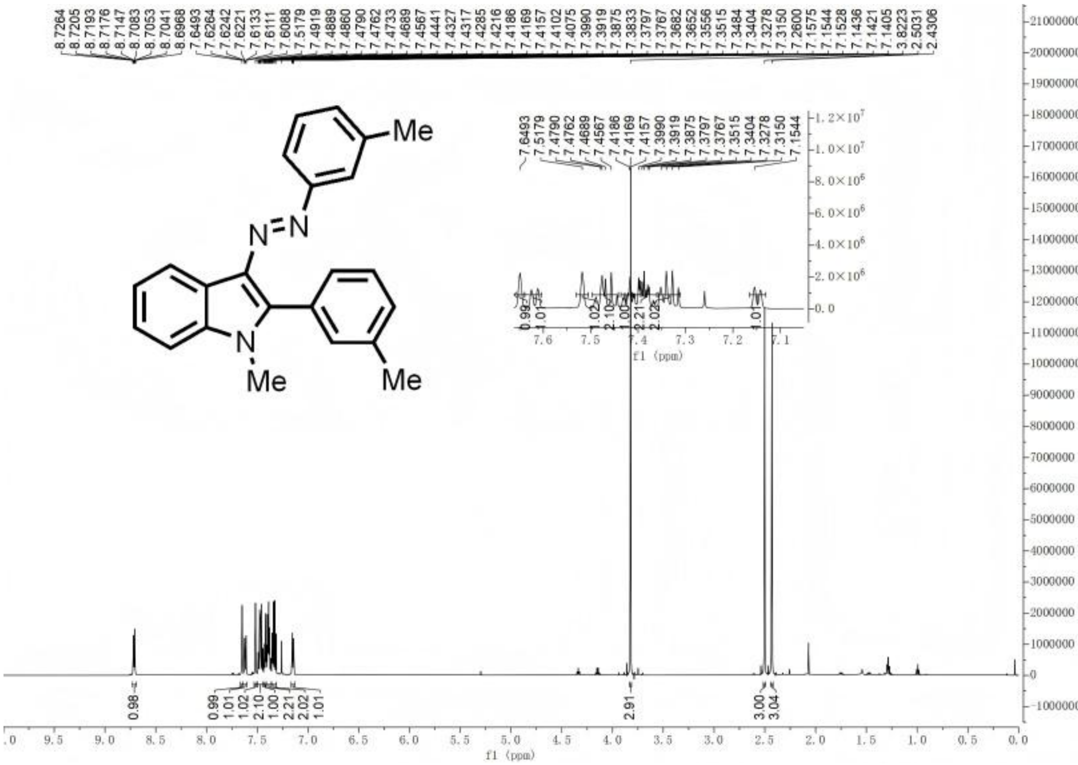

Fig. S13B: ^{13}C NMR of product **3j** in $CDCl_3$ (151 MHz)

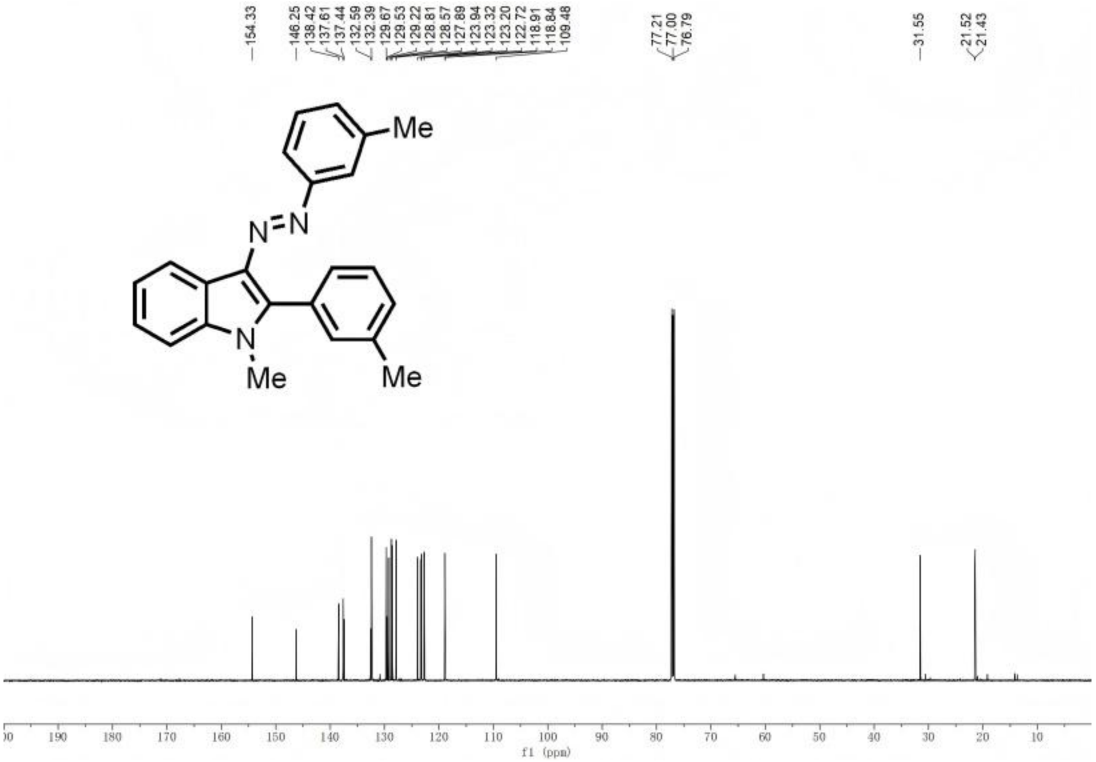

Fig. S14A: ^{1}H NMR of product **3k** in $CDCl_3$ (400 MHz)

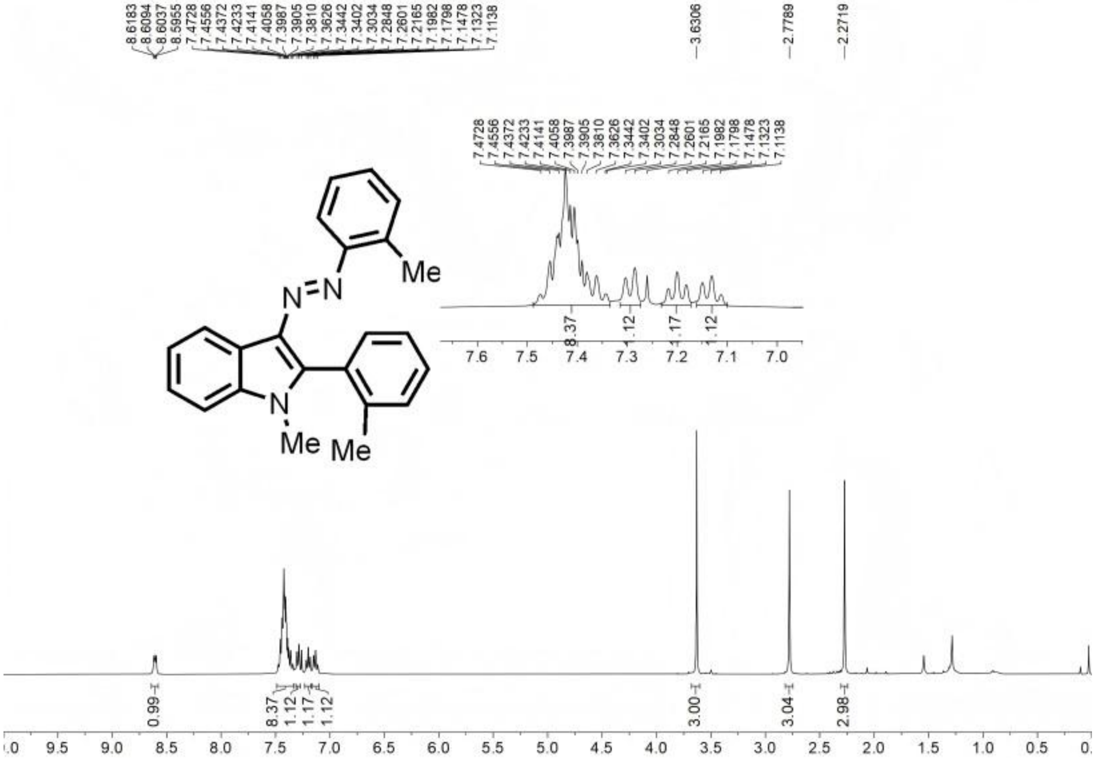

Fig. S14B: ^{13}C NMR of product **3k** in $CDCl_3$ (101 MHz)

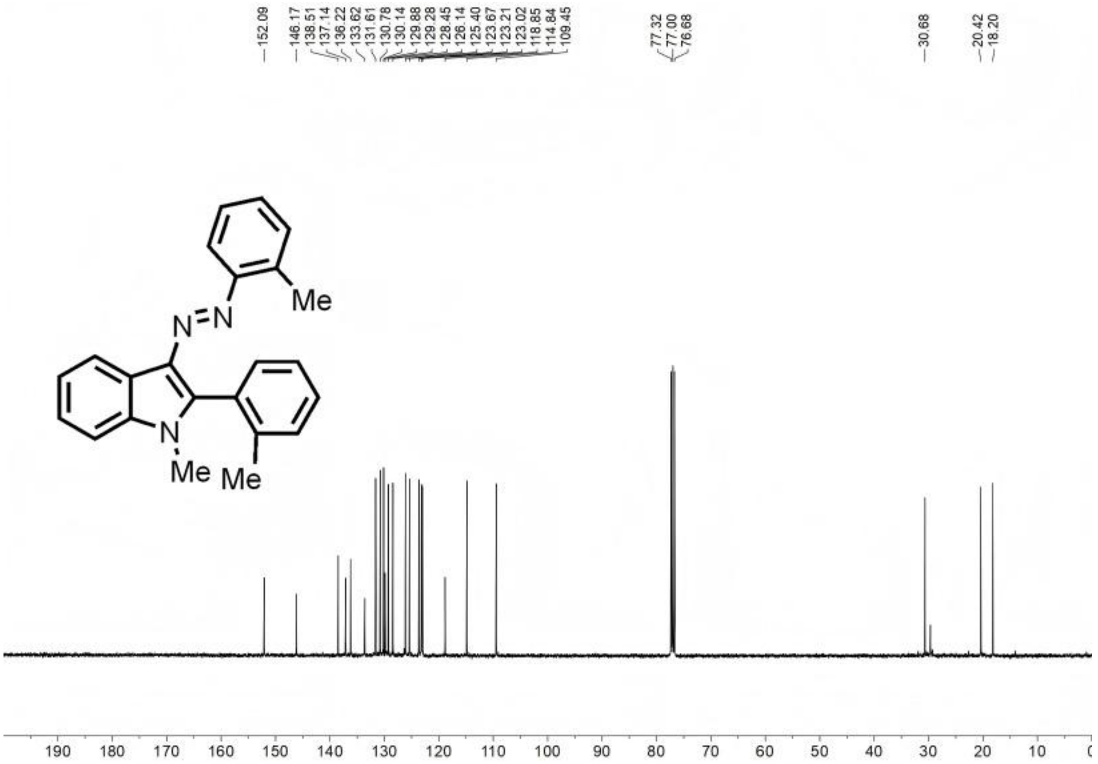

Fig. S15A: ^{1}H NMR of product **3l** in $CDCl_3$ (400 MHz)

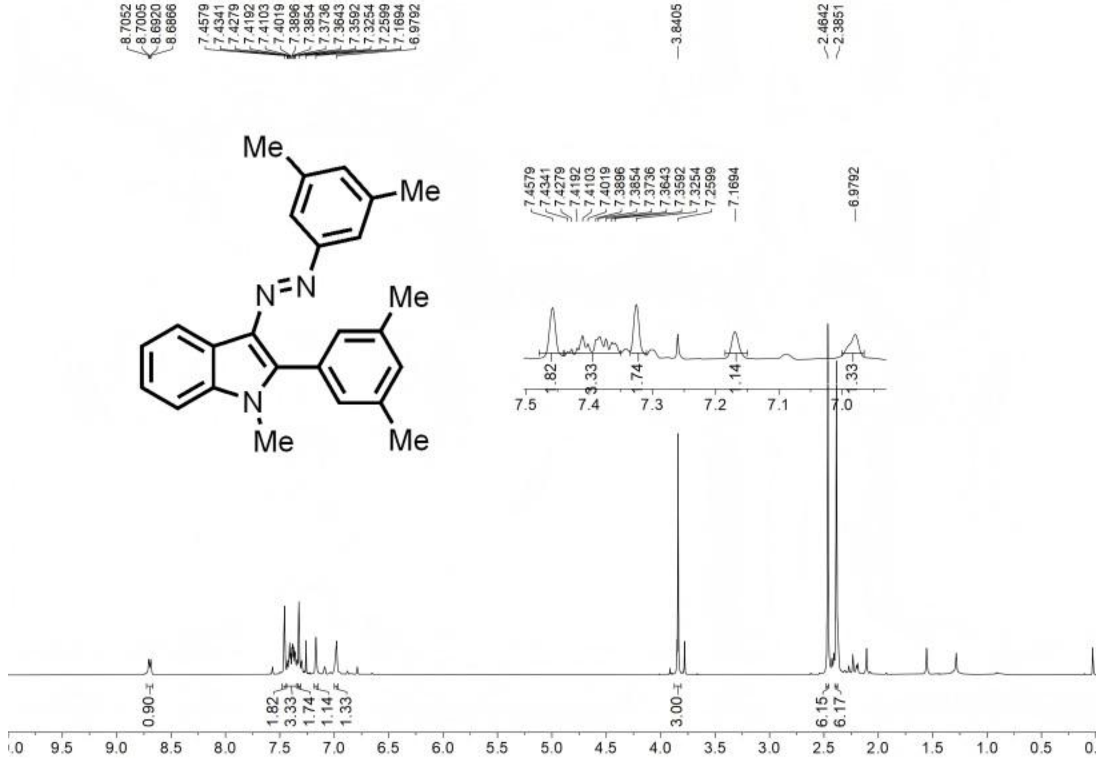

Fig. S15B: ^{13}C NMR of product **3l** in $CDCl_3$ (101 MHz)

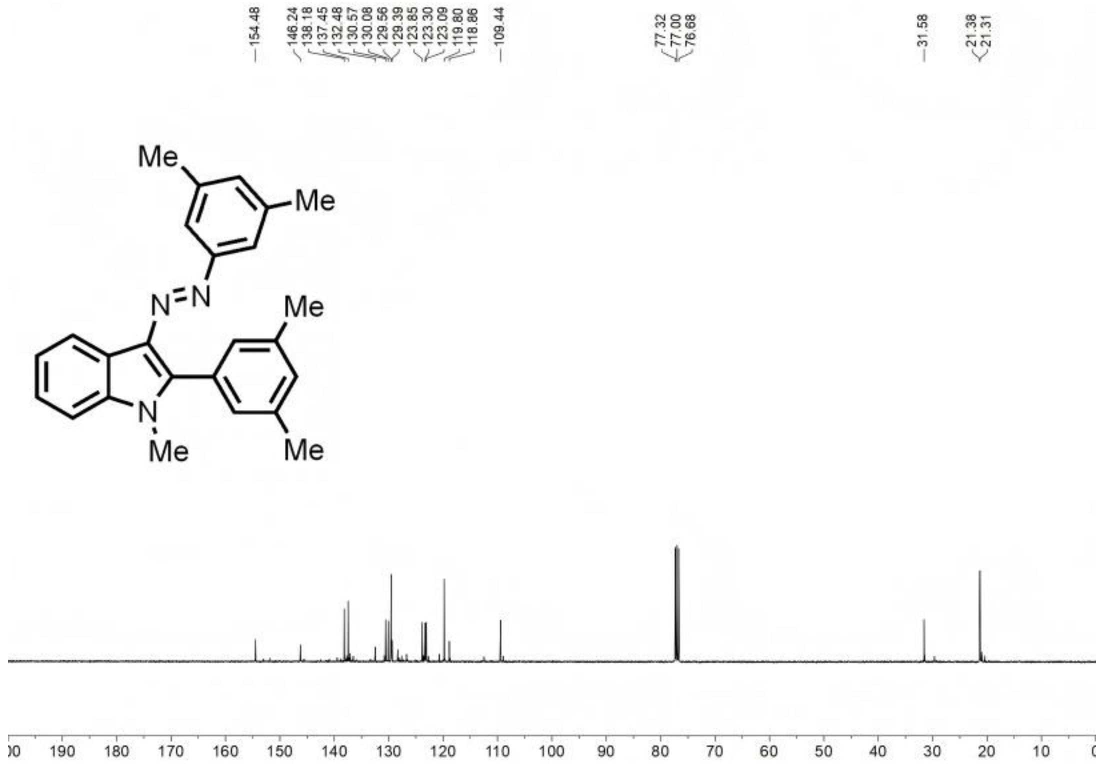

Fig. S16A: ^{1}H NMR of product **3m** in $CDCl_3$ (400 MHz)

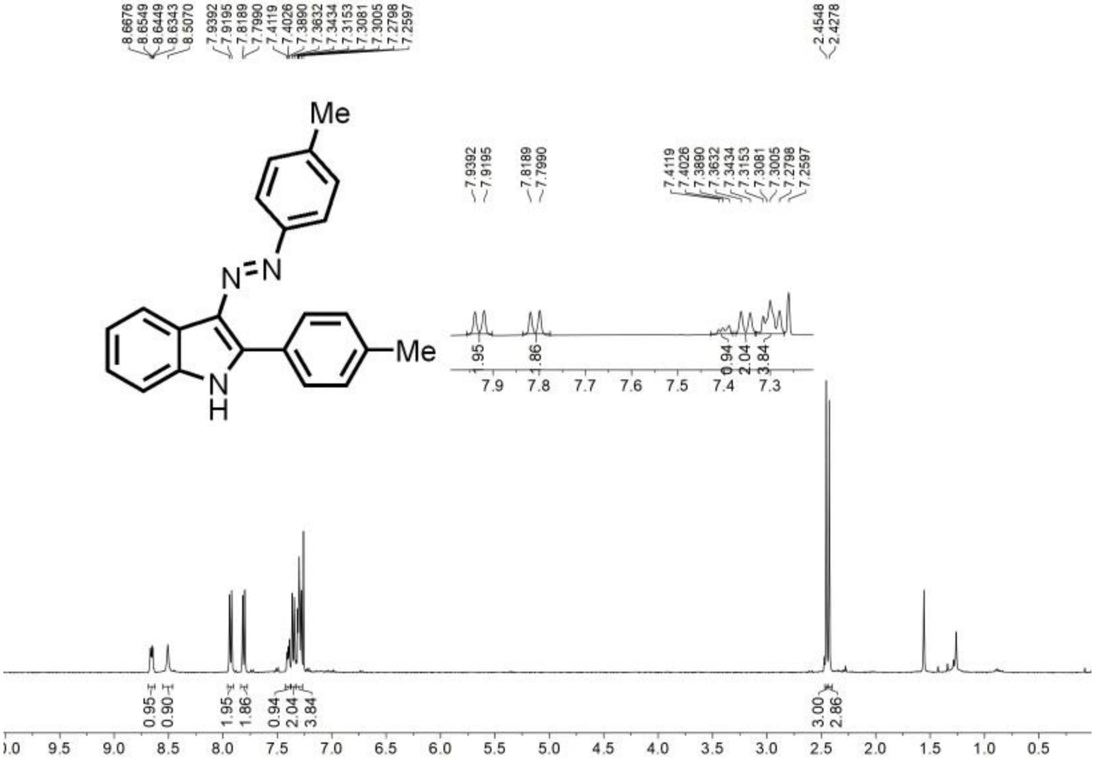

Fig. S16B: ^{13}C NMR of product **3m** in $CDCl_3$ (101 MHz)

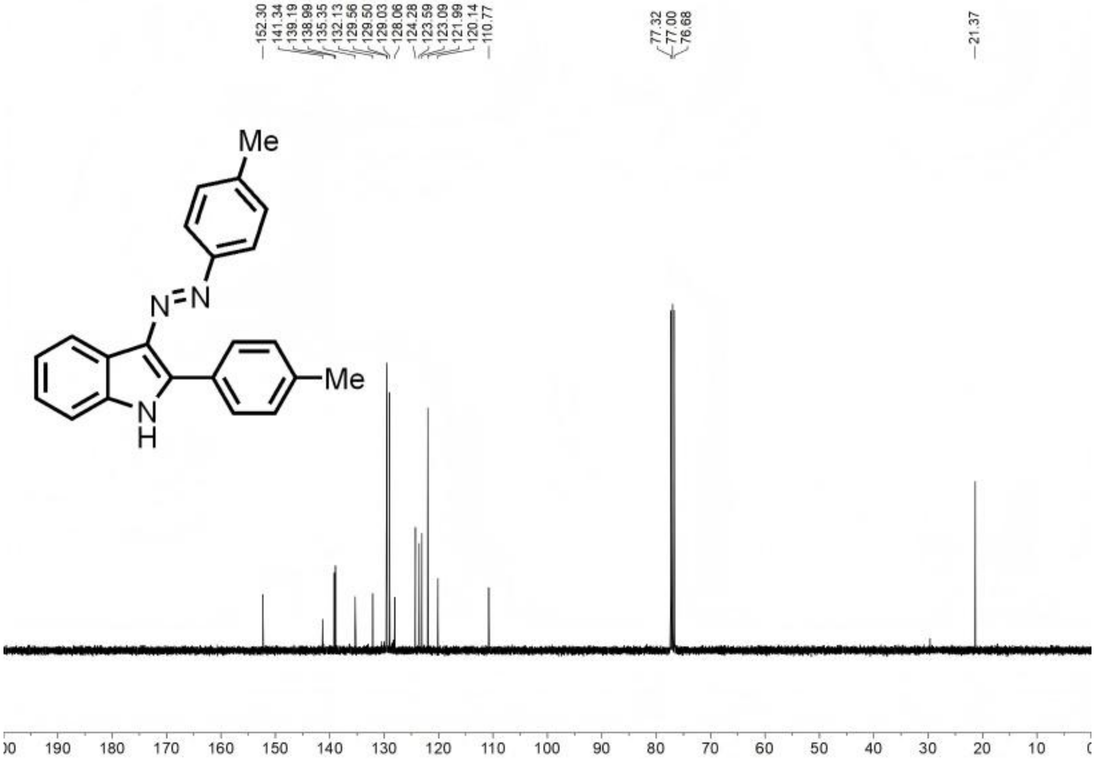

Fig. S17A: ^{1}H NMR of product **3n** in $CDCl_3$ (600 MHz)

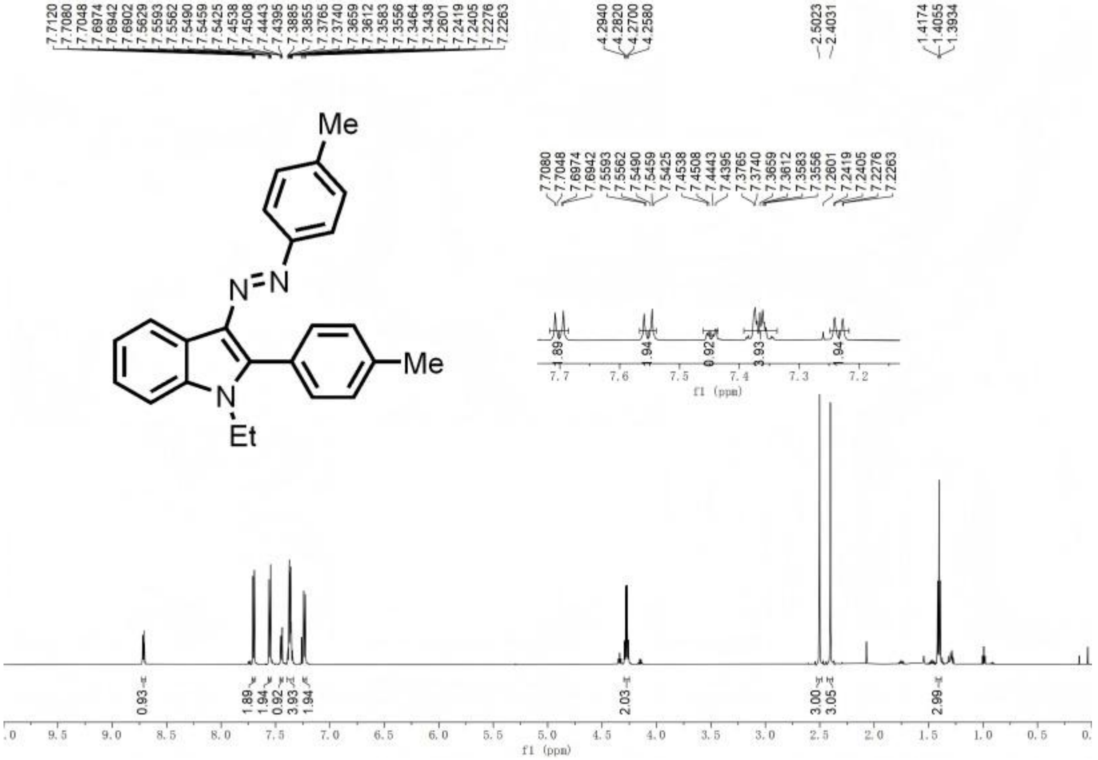

Fig. S17B: ^{13}C NMR of product **3n** in $CDCl_3$ (151 MHz)

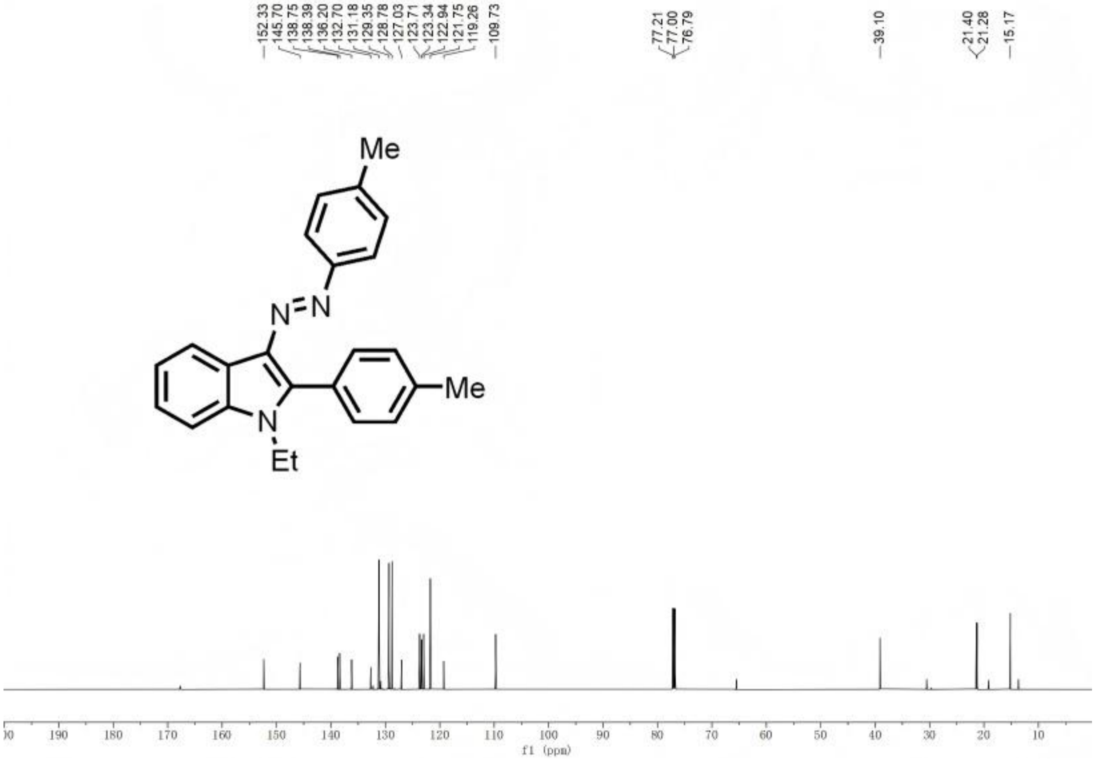

Fig. S18A: ^{1}H NMR of product **3o** in $CDCl_3$ (600 MHz)

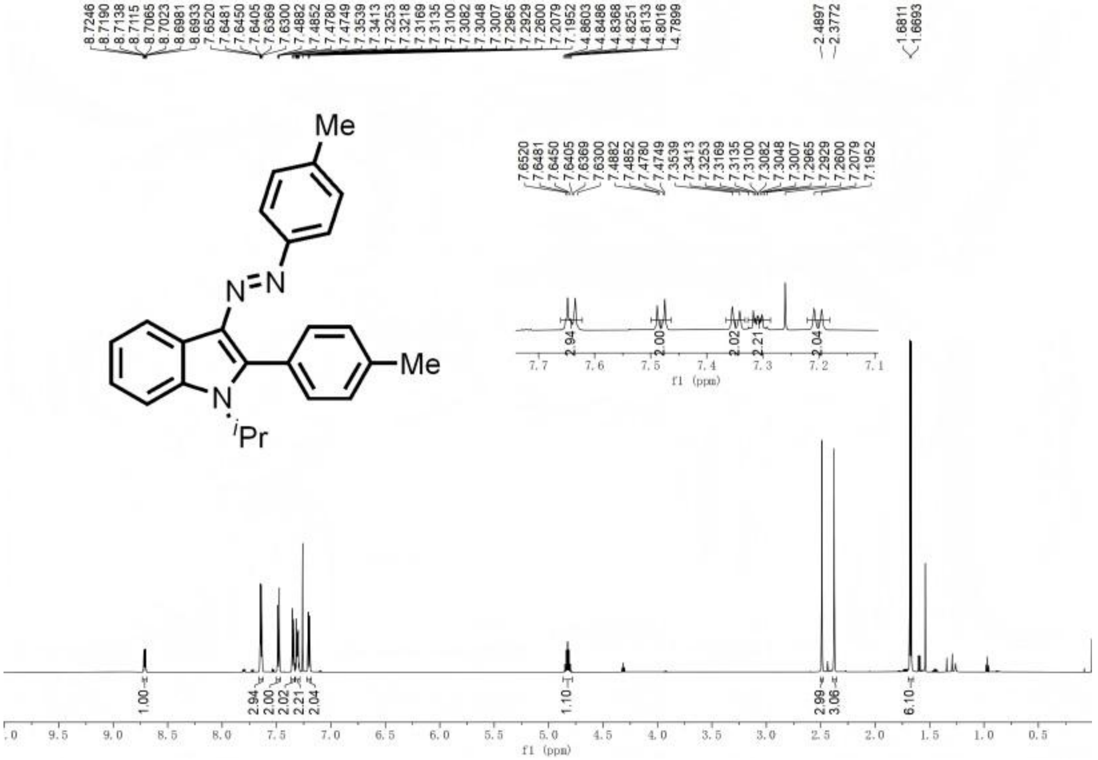

Fig. S18B: ^{13}C NMR of product **3o** in $CDCl_3$ (151 MHz)

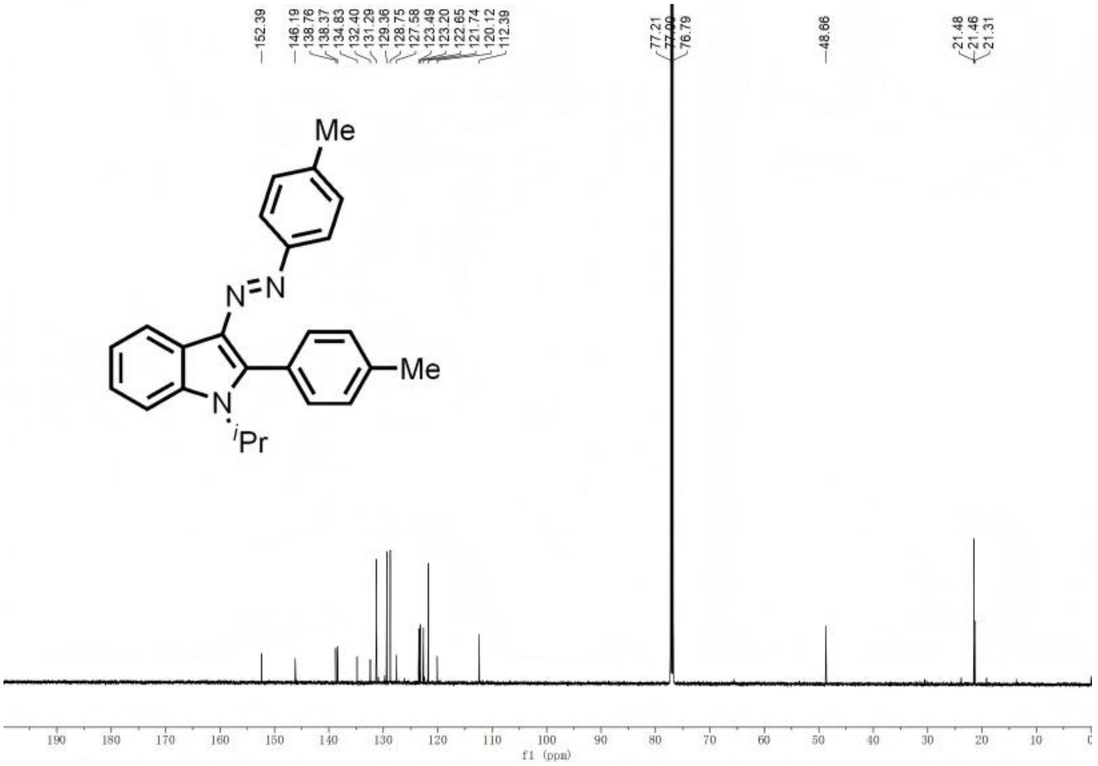

Fig. S19A: ^{1}H NMR of product **3p** in $CDCl_3$ (600 MHz)

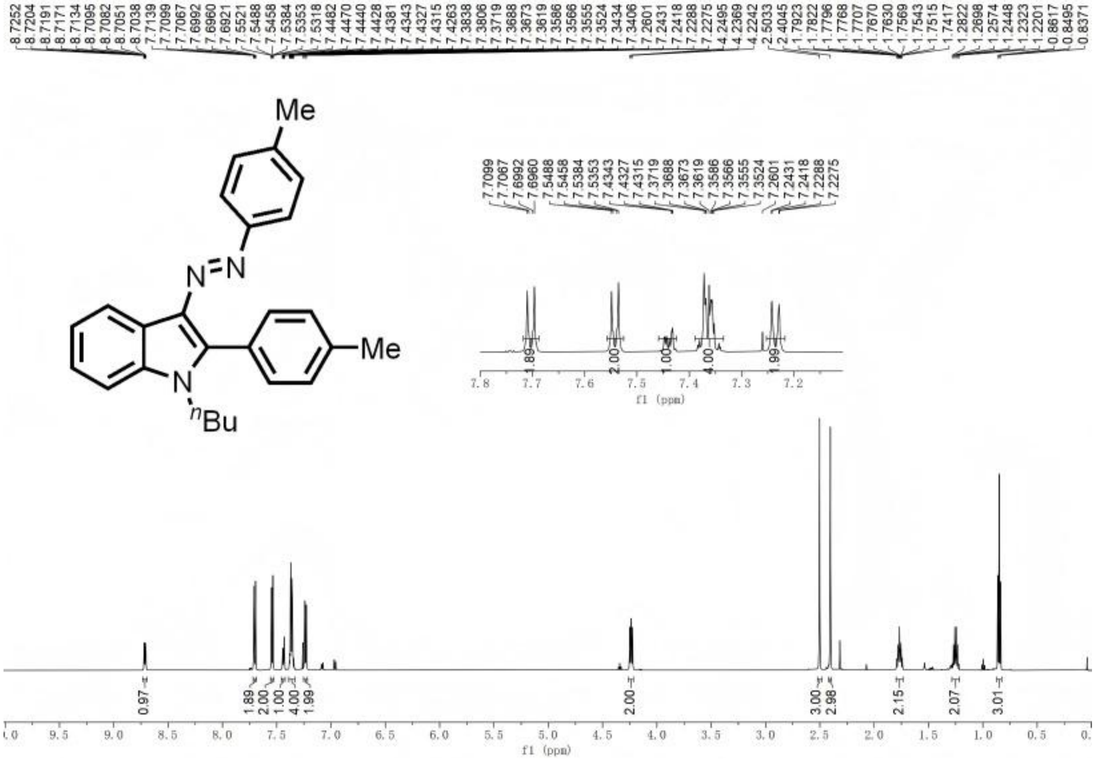

Fig. S19B: ^{13}C NMR of product **3p** in $CDCl_3$ (151 MHz)

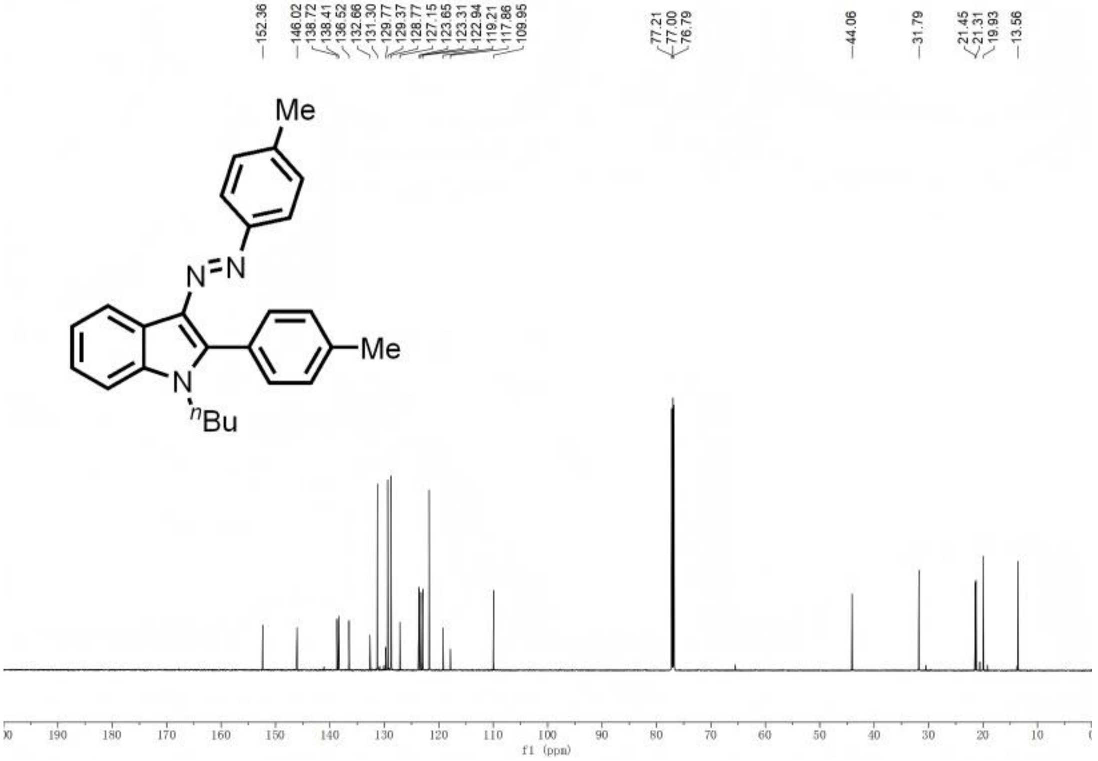

Fig. S20A: ^{1}H NMR of product **3r** in $CDCl_3$ (600 MHz)

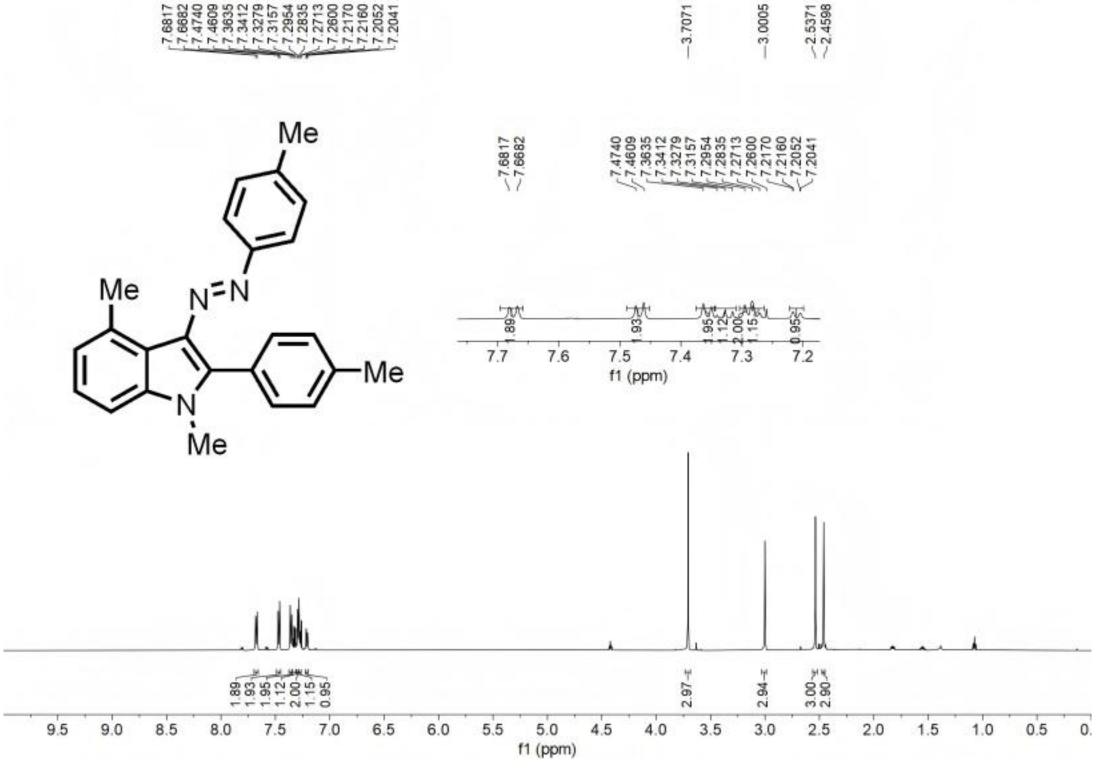

Fig. S20B: ^{13}C NMR of product **3r** in $CDCl_3$ (151 MHz)

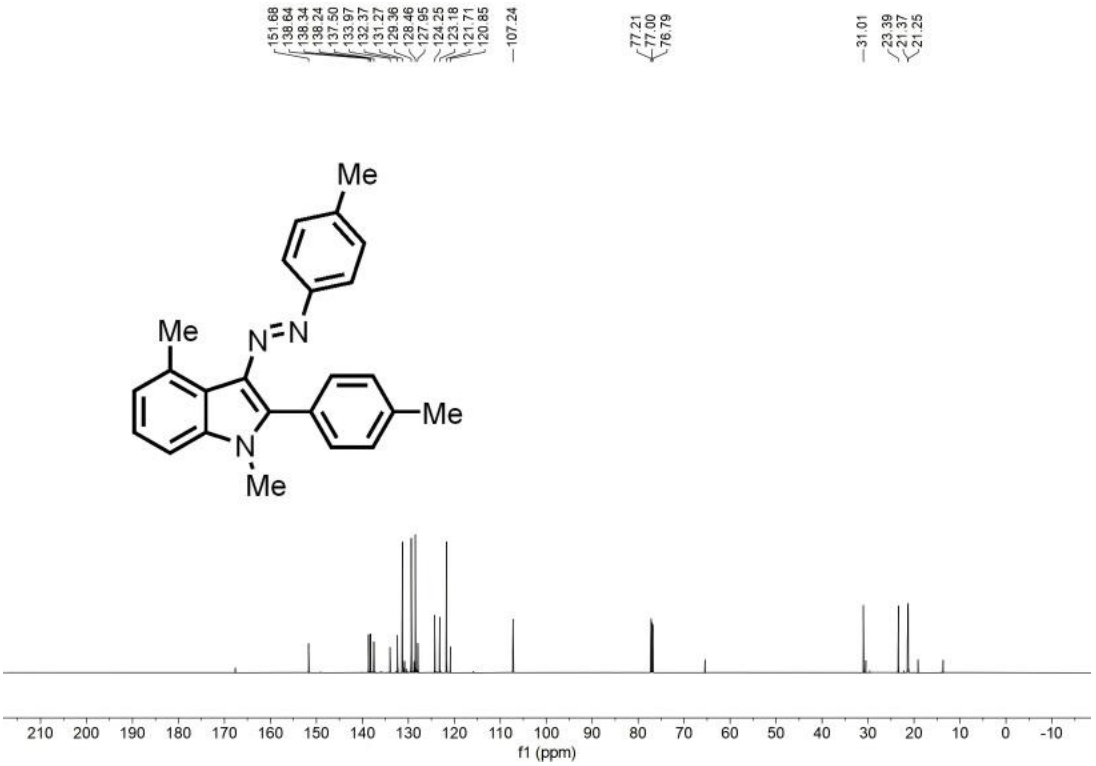

Fig. S21A: ^{1}H NMR of product **3s** in $CDCl_3$ (600 MHz)

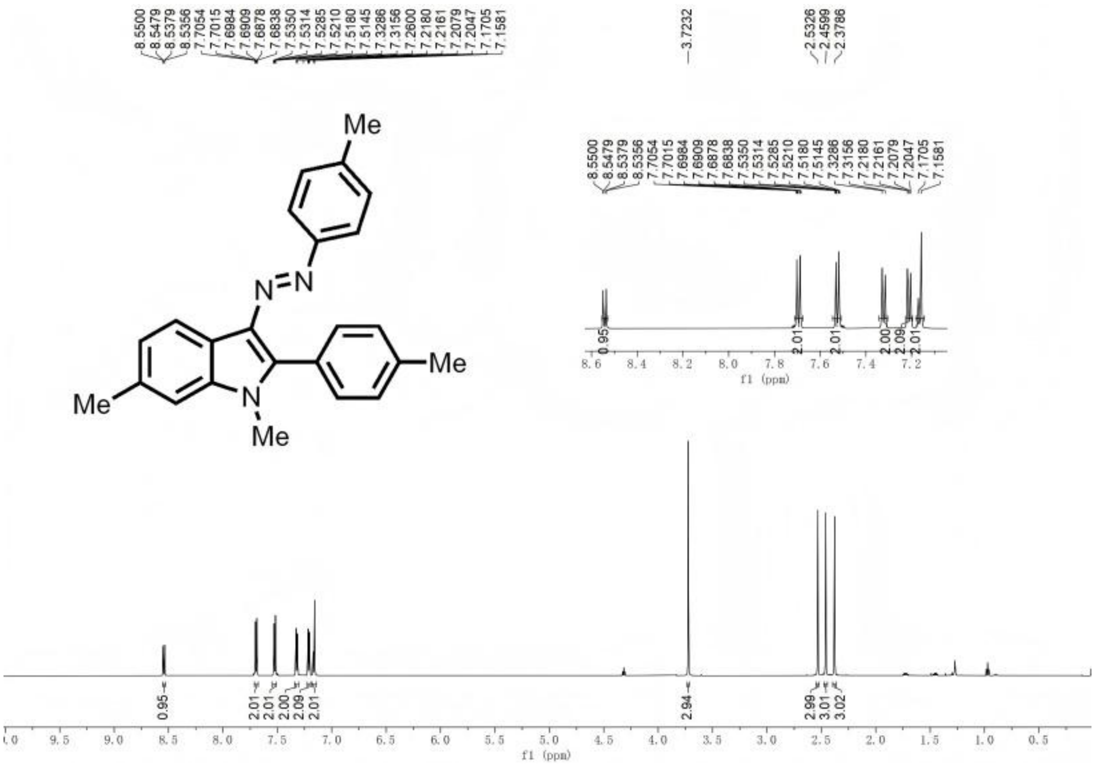

Fig. S21B: ^{13}C NMR of product **3s** in $CDCl_3$ (151 MHz)

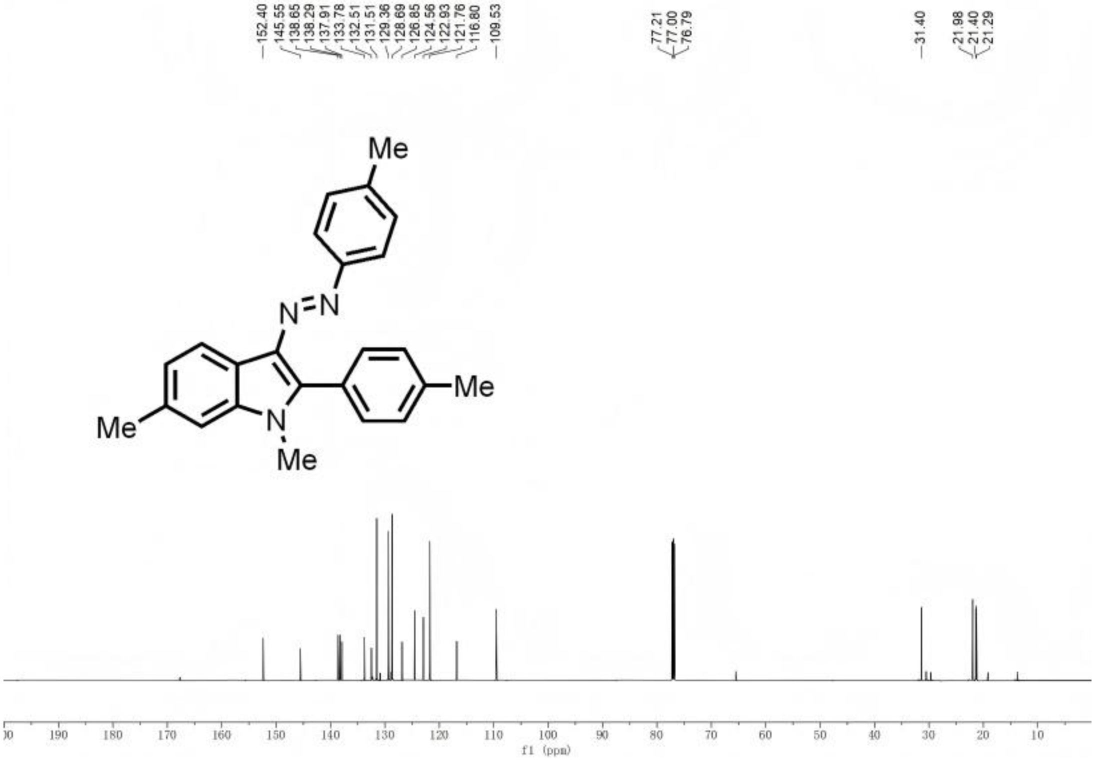

Fig. S22A: ^{1}H NMR of product **3t** in $CDCl_3$ (400 MHz)

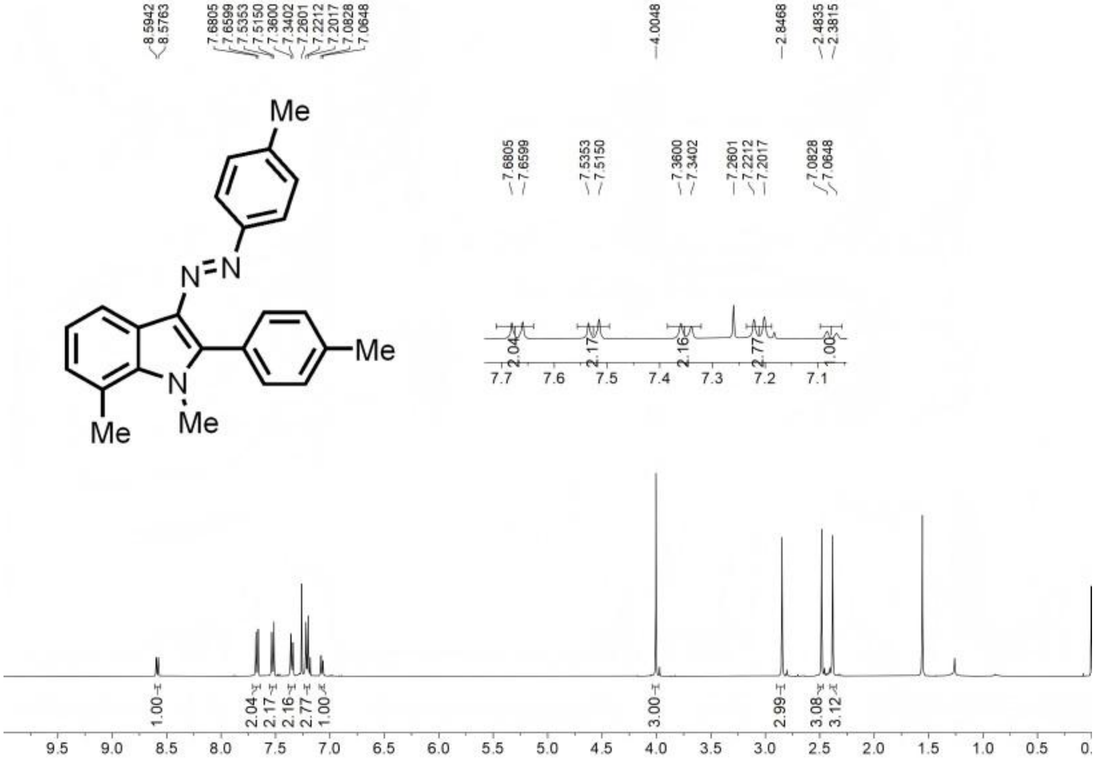

Fig. S22B: ^{13}C NMR of product **3t** in $CDCl_3$ (101 MHz)

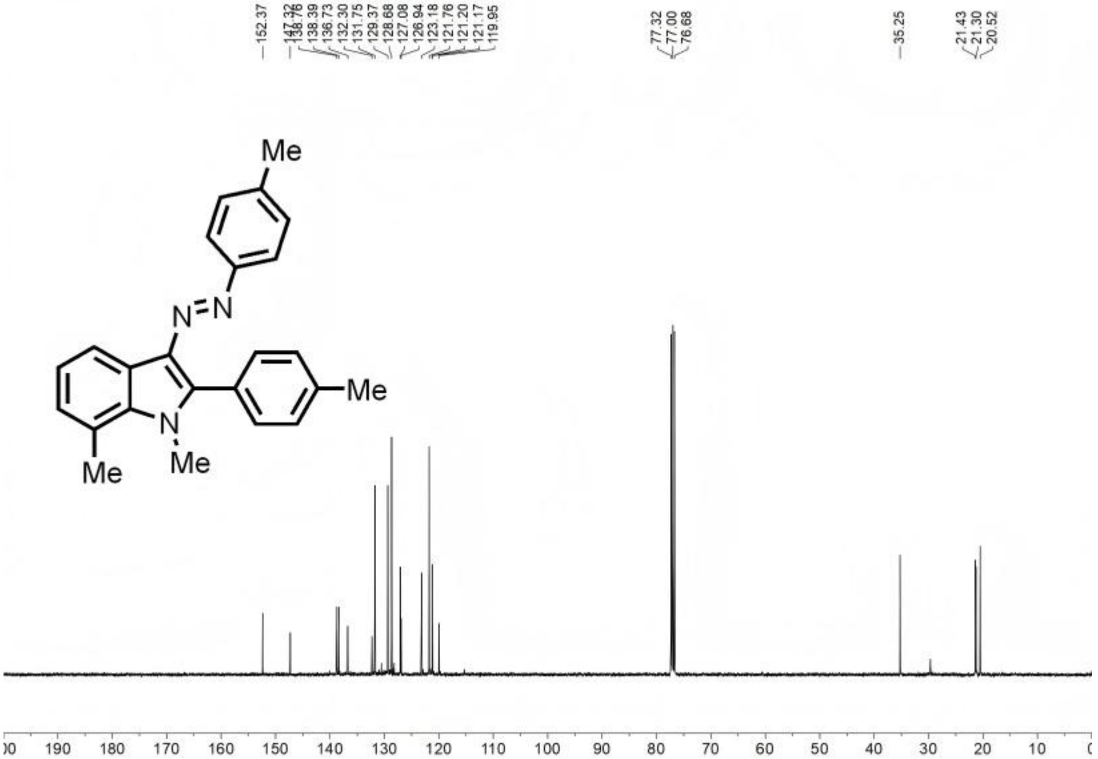

Fig. S23A: ^{1}H NMR of product **3u** in $CDCl_3$ (400 MHz)

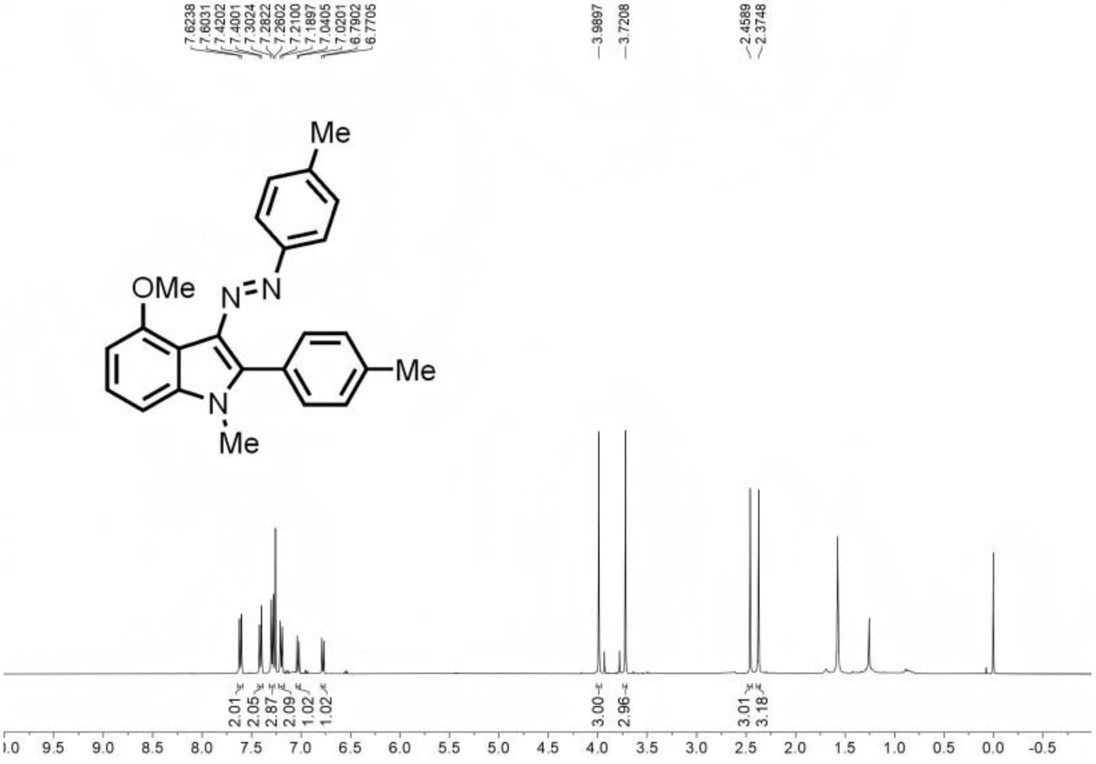

Fig. S23B: ^{13}C NMR of product **3u** in $CDCl_3$ (101 MHz)

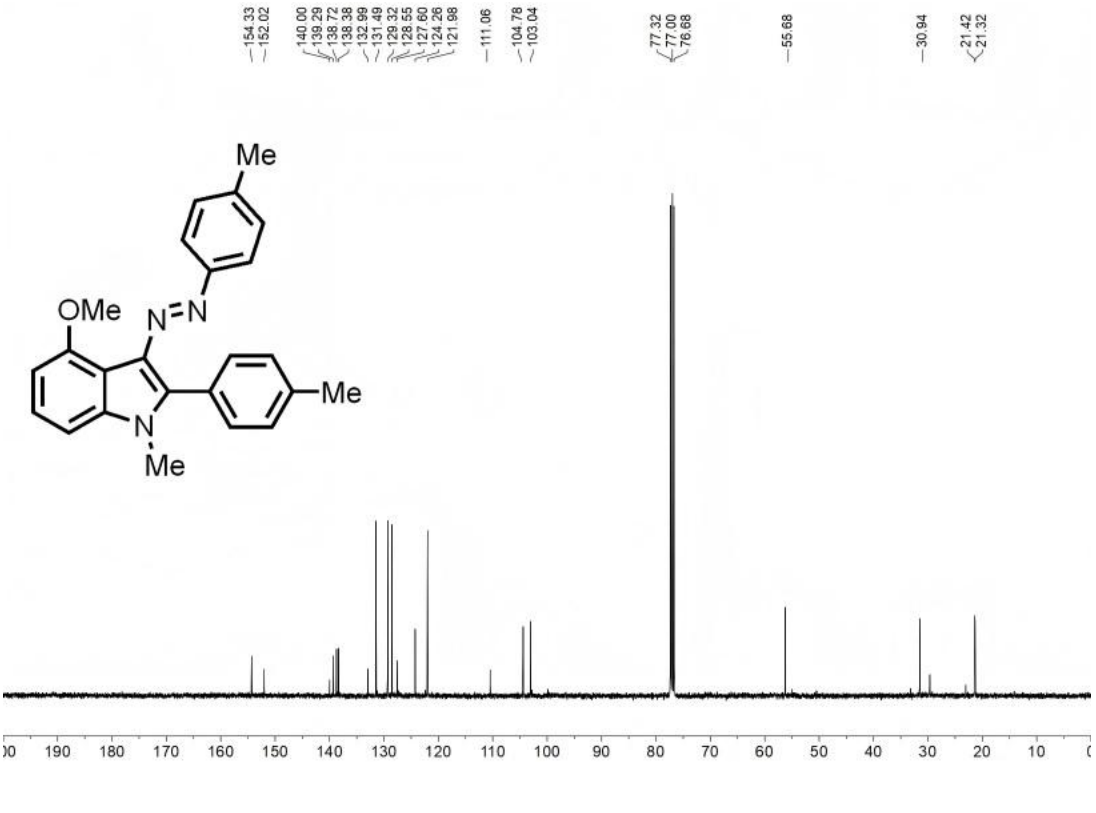

Fig. S24A: ^{1}H NMR of product **3v** in $CDCl_3$ (400 MHz)

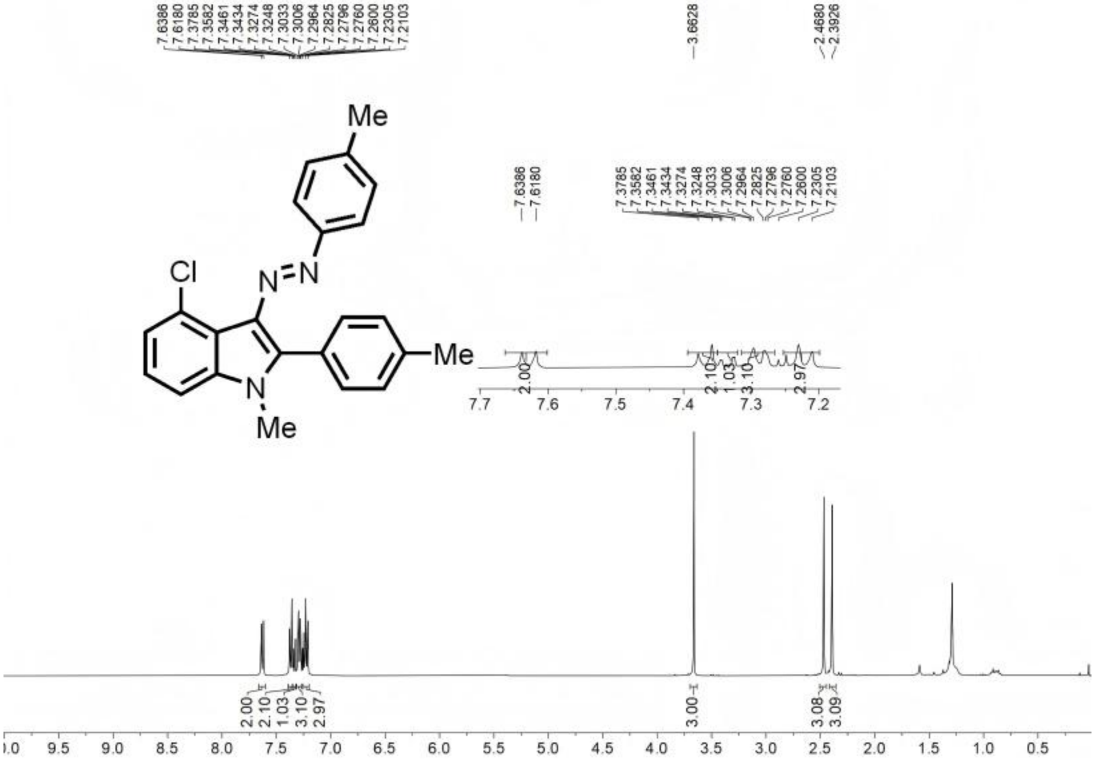

Fig. S24B: ^{13}C NMR of product **3v** in $CDCl_3$ (101 MHz)

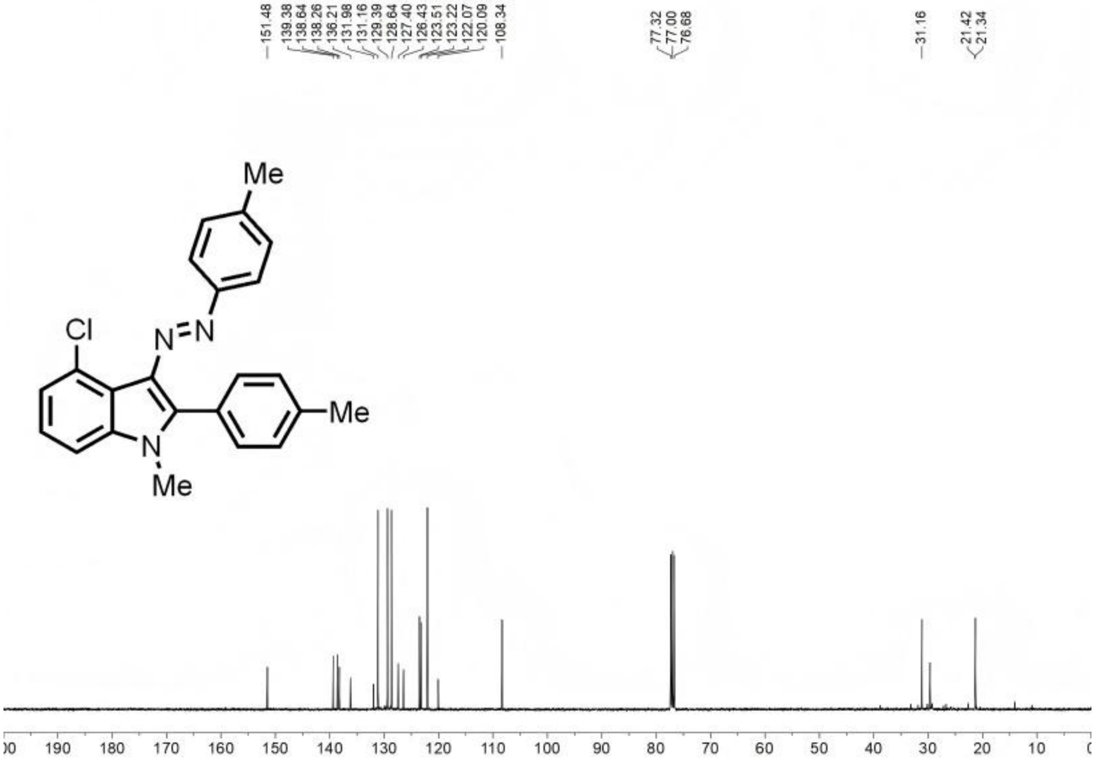

Fig. S25A: ^{1}H NMR of product **3w** in $CDCl_3$ (600 MHz)

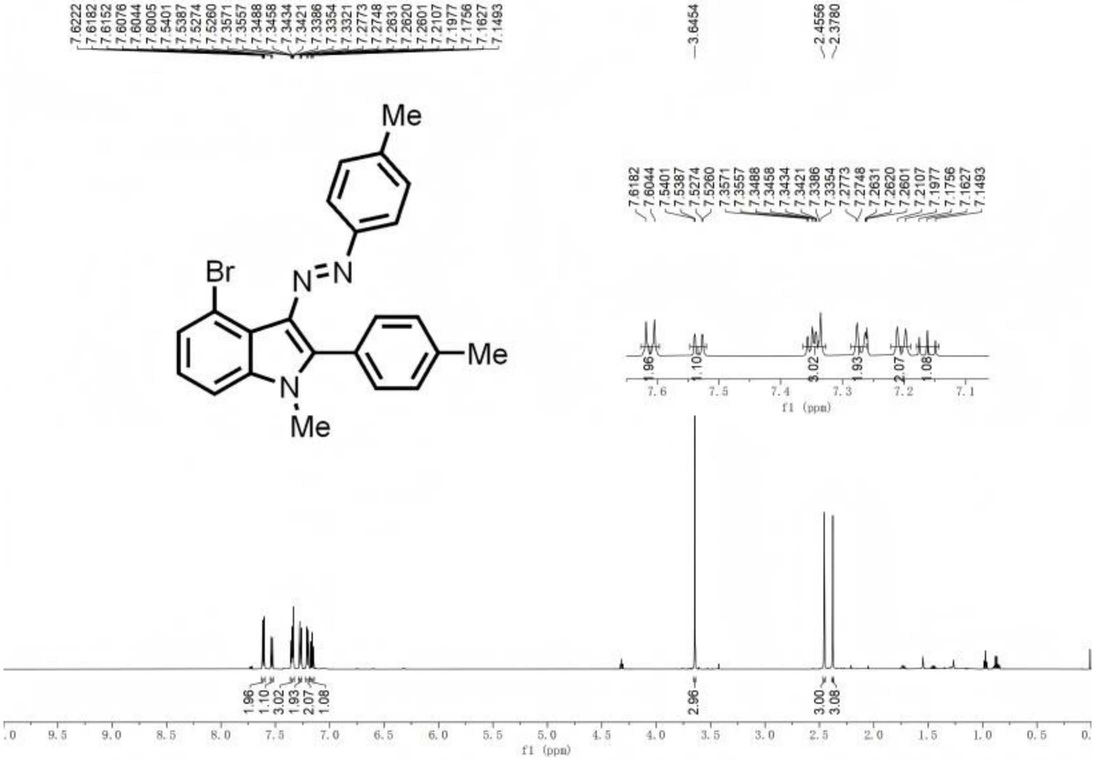

Fig. S25B: ^{13}C NMR of product **3w** in $CDCl_3$ (151 MHz)

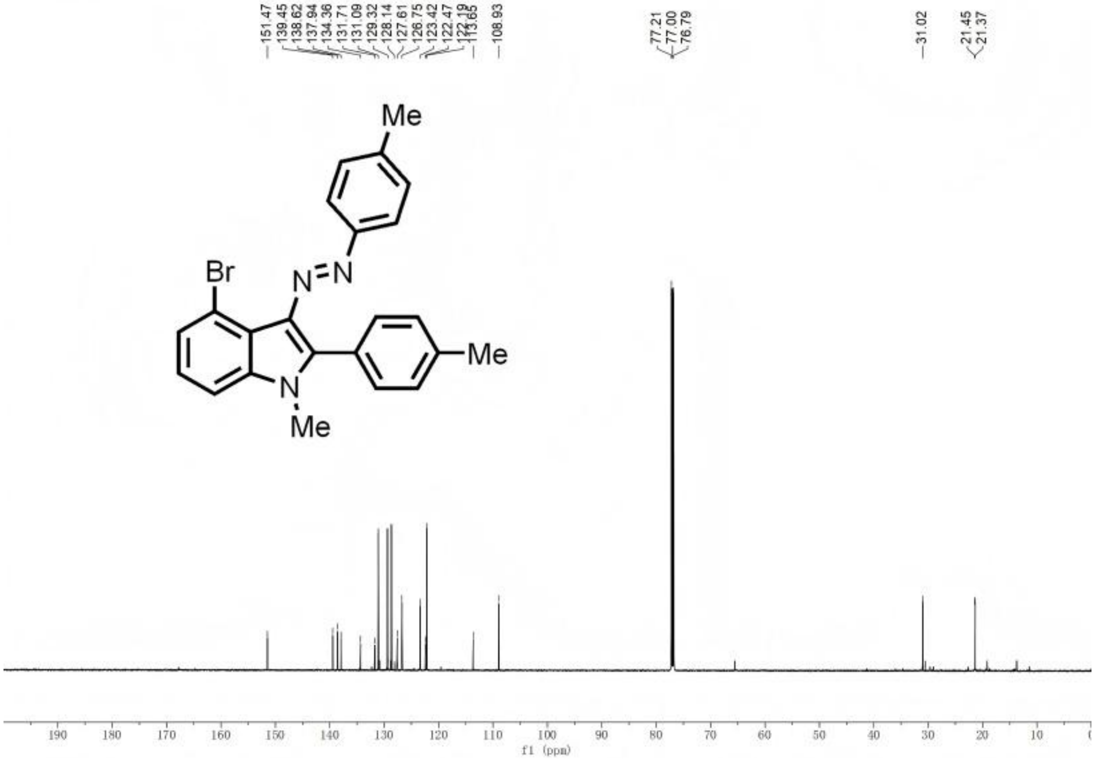

Fig. S26A: ^{1}H NMR of product **3x** in $CDCl_3$ (400 MHz)

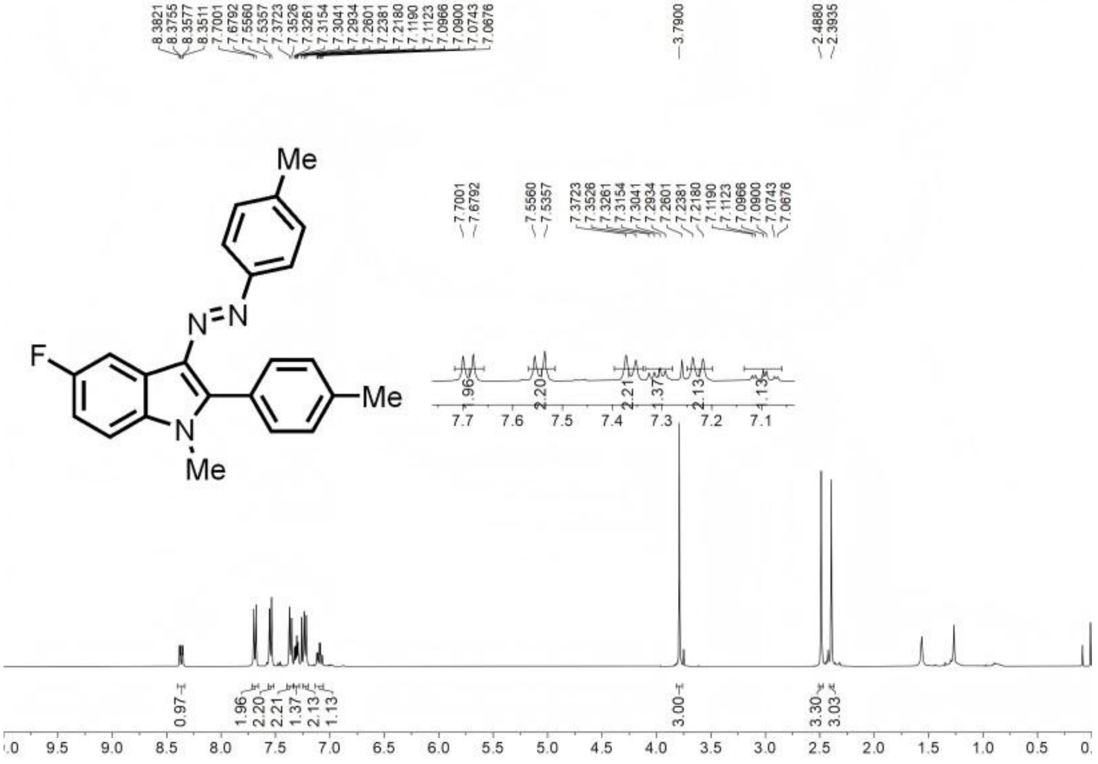

Fig. S26B: ^{13}C NMR of product **3x** in $CDCl_3$ (101 MHz)

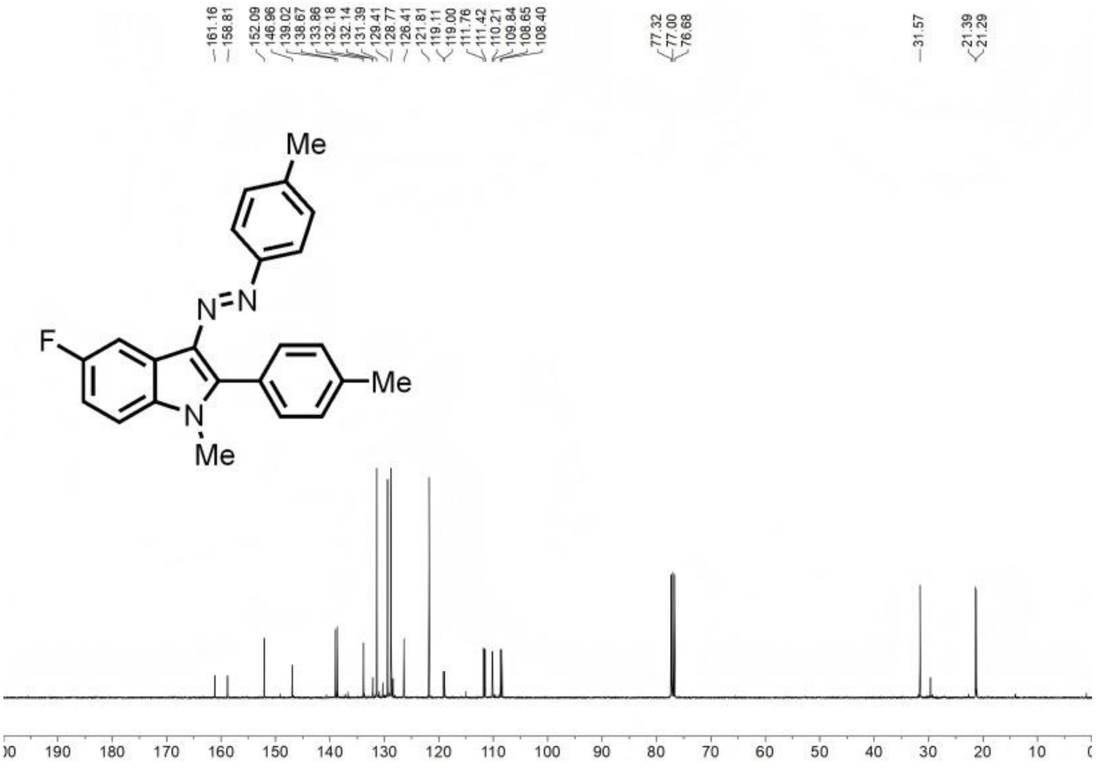

Fig. S27A: ^{1}H NMR of product **3y** in $CDCl_3$ (600 MHz)

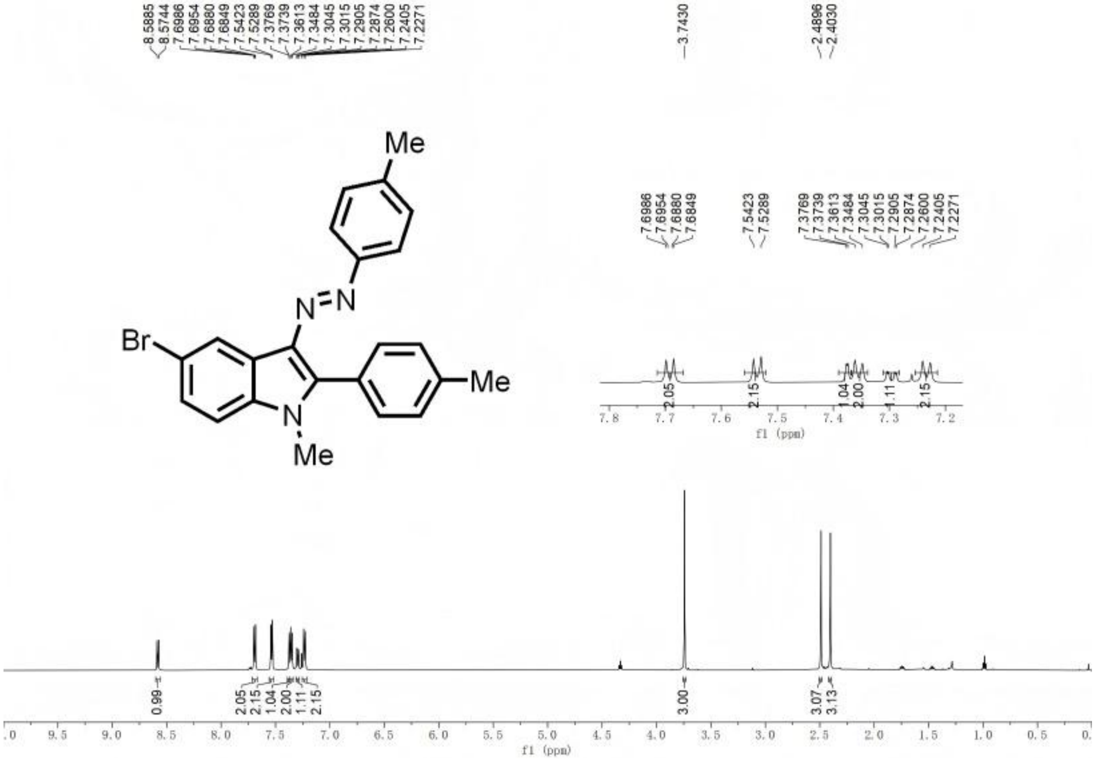

Fig. S27B: ^{13}C NMR of product **3y** in $CDCl_3$ (151 MHz)

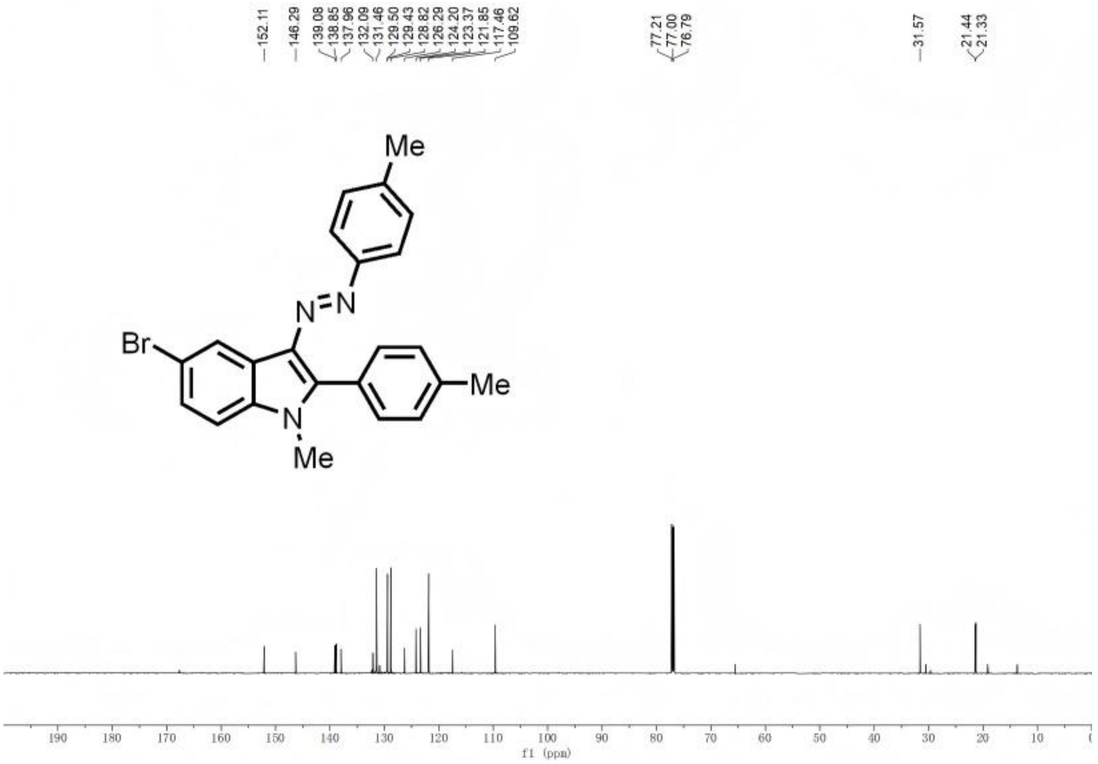

Fig. S28A: ^{1}H NMR of product **3z** in $CDCl_3$ (600 MHz)

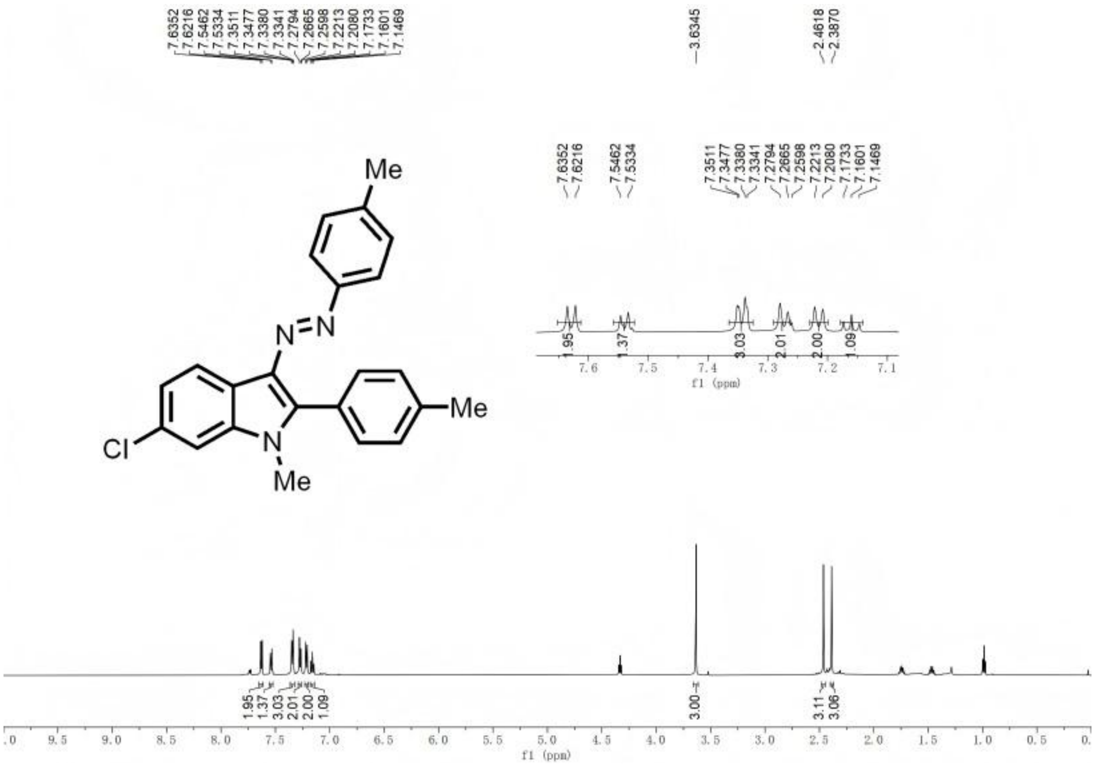

Fig. S28B: ^{13}C NMR of product **3z** in $CDCl_3$ (101 MHz)

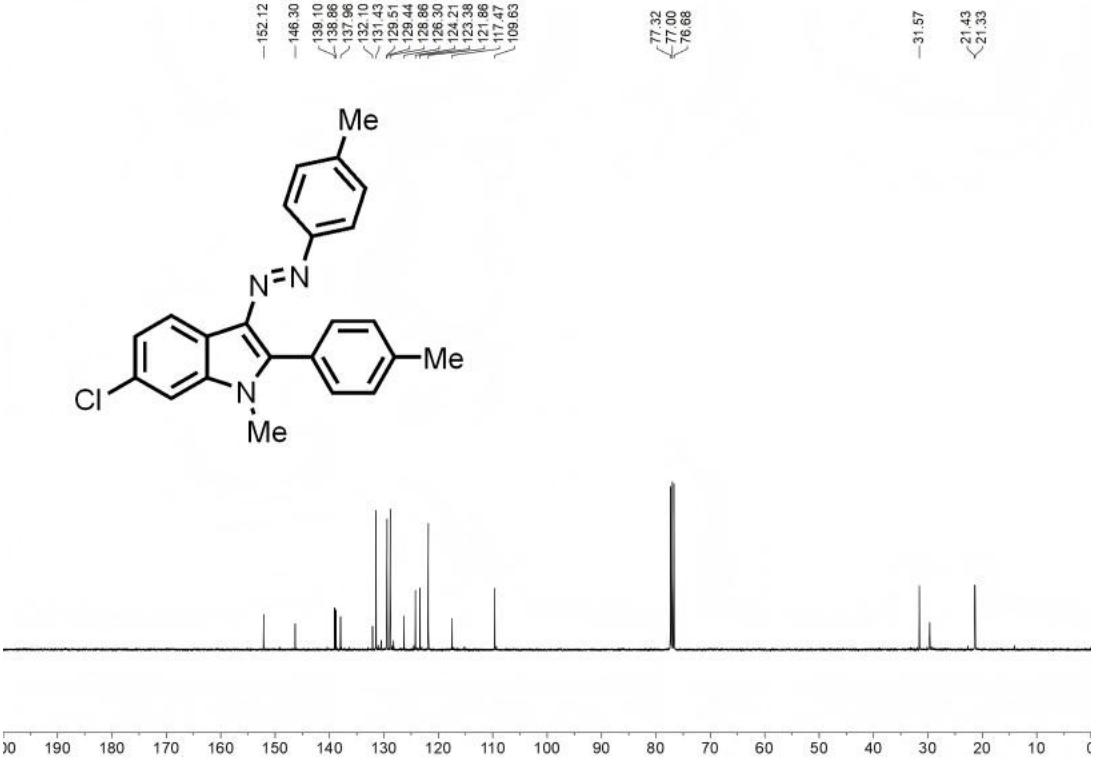

Fig. S29A: ^{1}H NMR of product **4a** in DMSO (600 MHz)

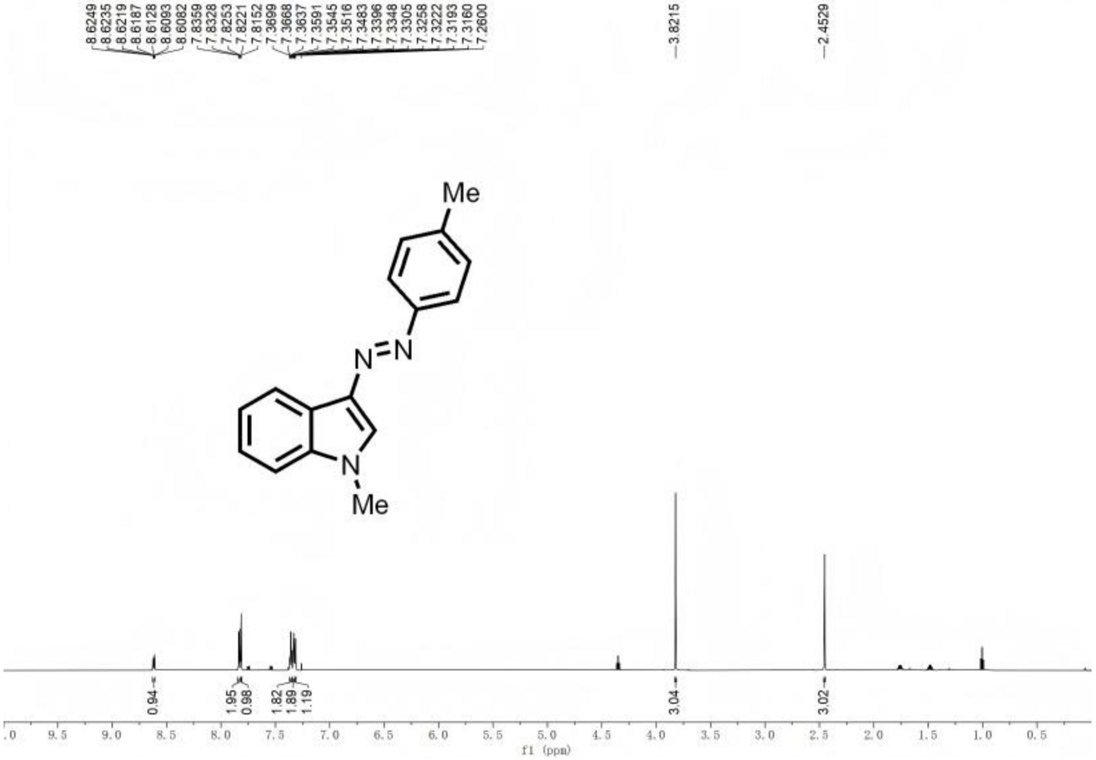

Fig. S29B: ^{13}C NMR of product **4a** in $CDCl_3$ (151 MHz)

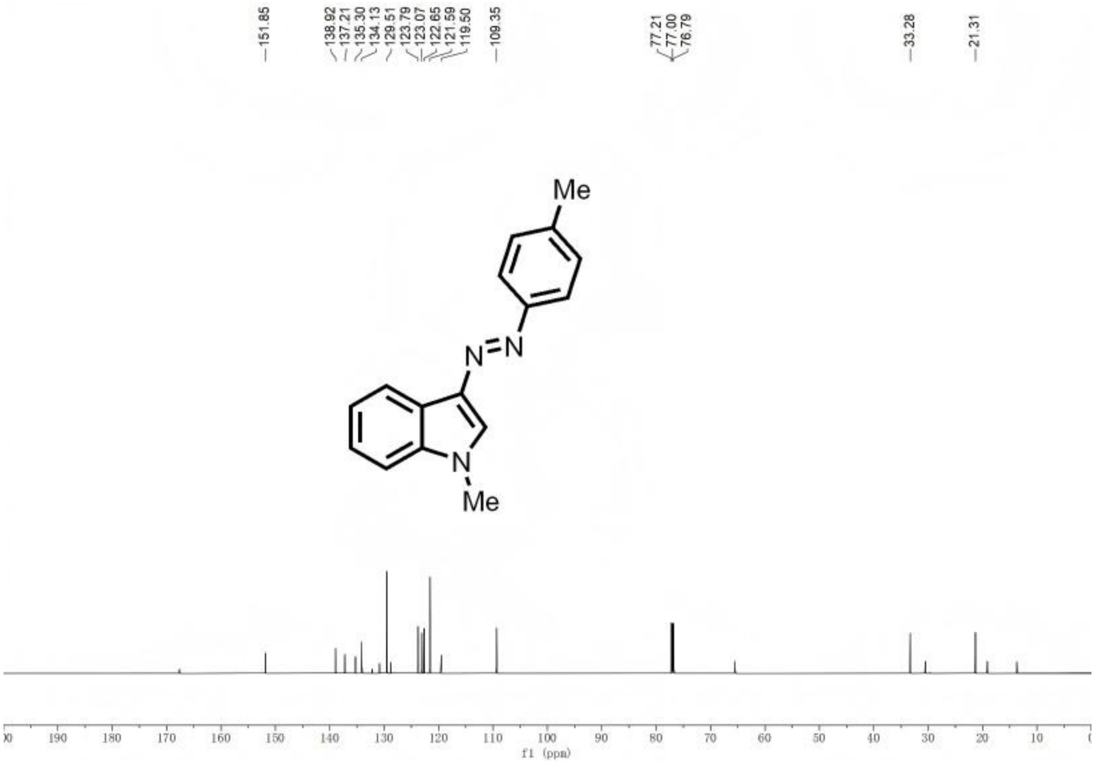

Fig. S30A: ^{1}H NMR of product **3aa** in DMSO (400 MHz)

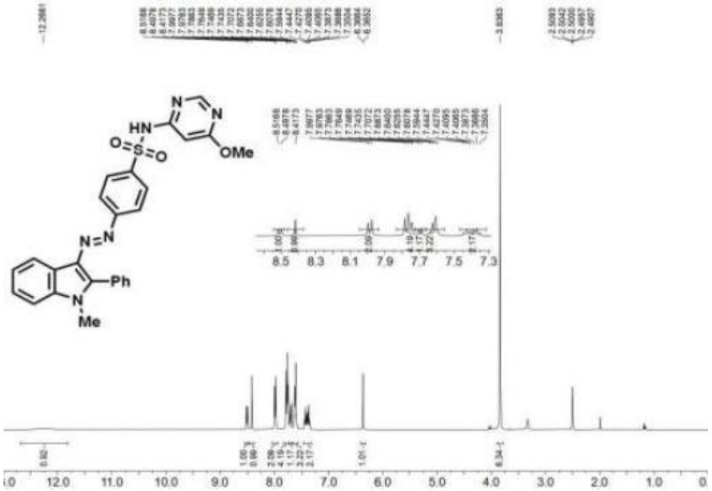

Fig. S30B: ^{13}C NMR of product **3aa** in DMSO (101 MHz)

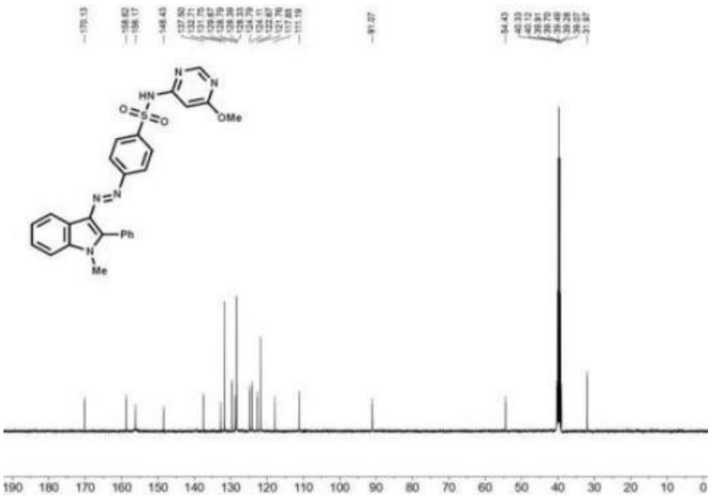

Fig. S30C: UV-Vis Absorption Spectrum of **3aa**

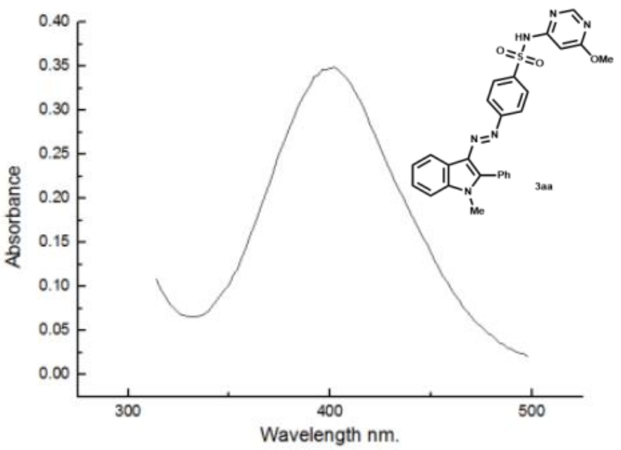

Fig. S31A: ^{1}H NMR of product **3ab** in DMSO (400 MHz)

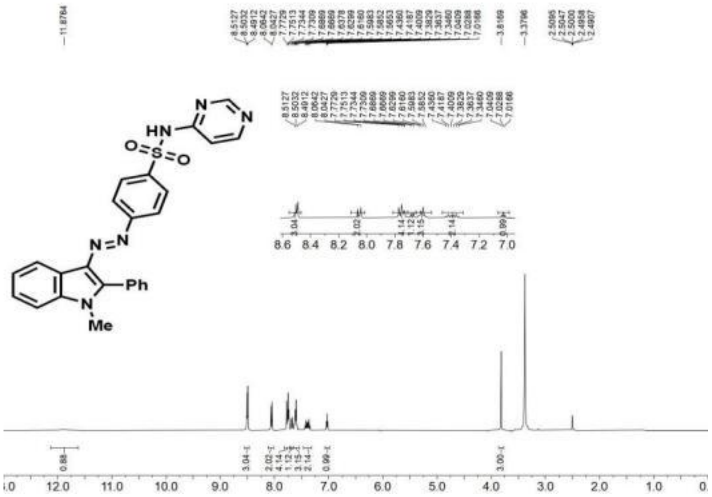

Fig. S31B: ^{13}C NMR of product **3ab** in DMSO (101 MHz)

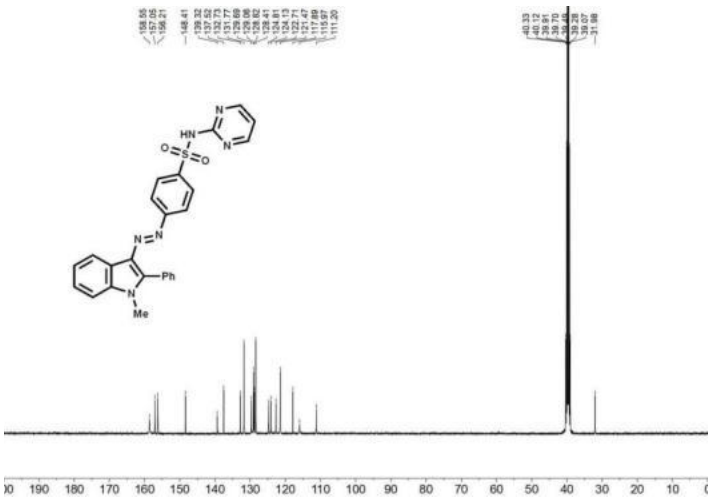

Fig. S31C: UV-Vis Absorption Spectrum of **3ab**

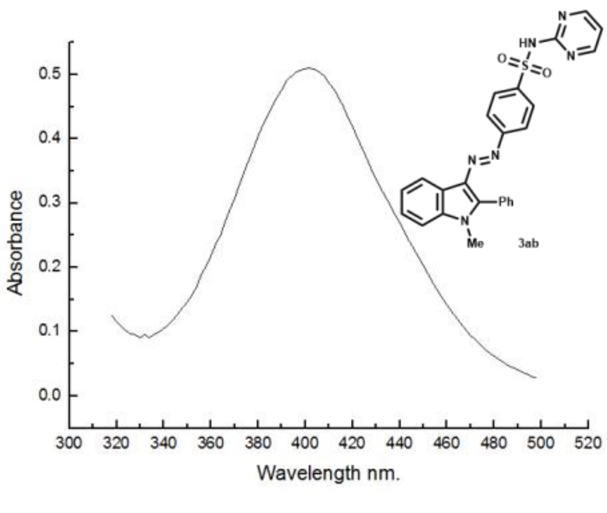

Fig. S32A: ^{1}H NMR of product **5a** in $CDCl_3$ (600 MHz)

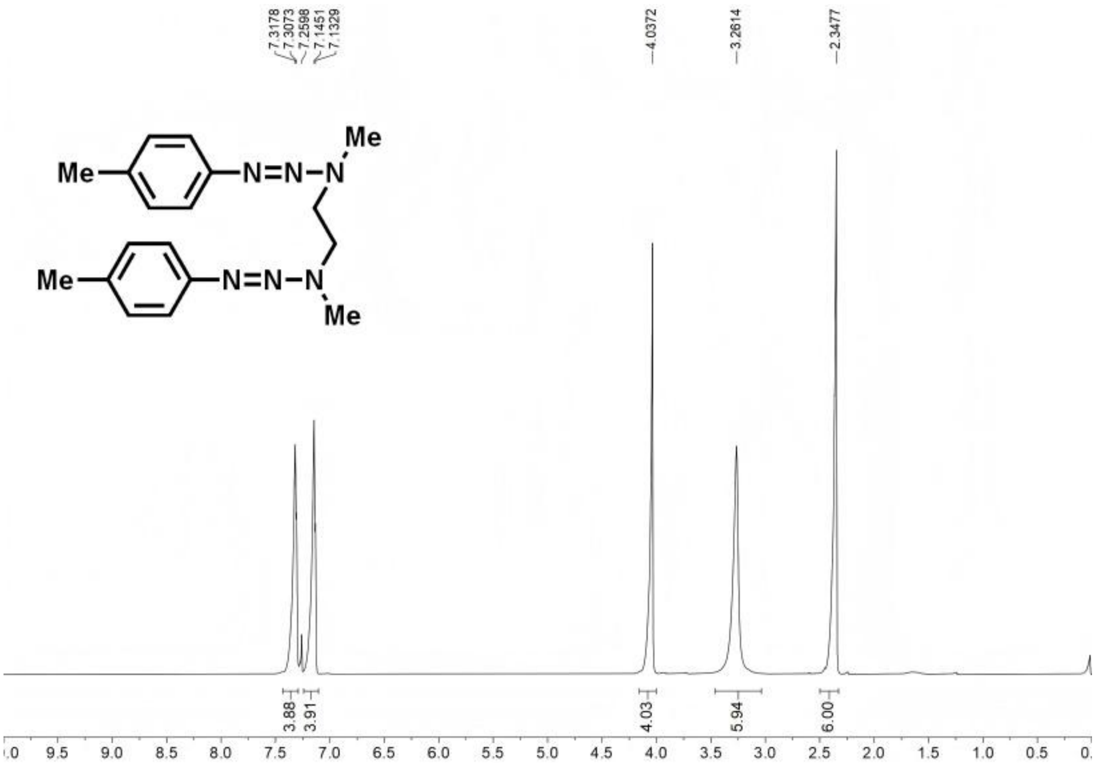

Fig. S32B: ^{13}C NMR of product **5a** in $CDCl_3$ (151 MHz)

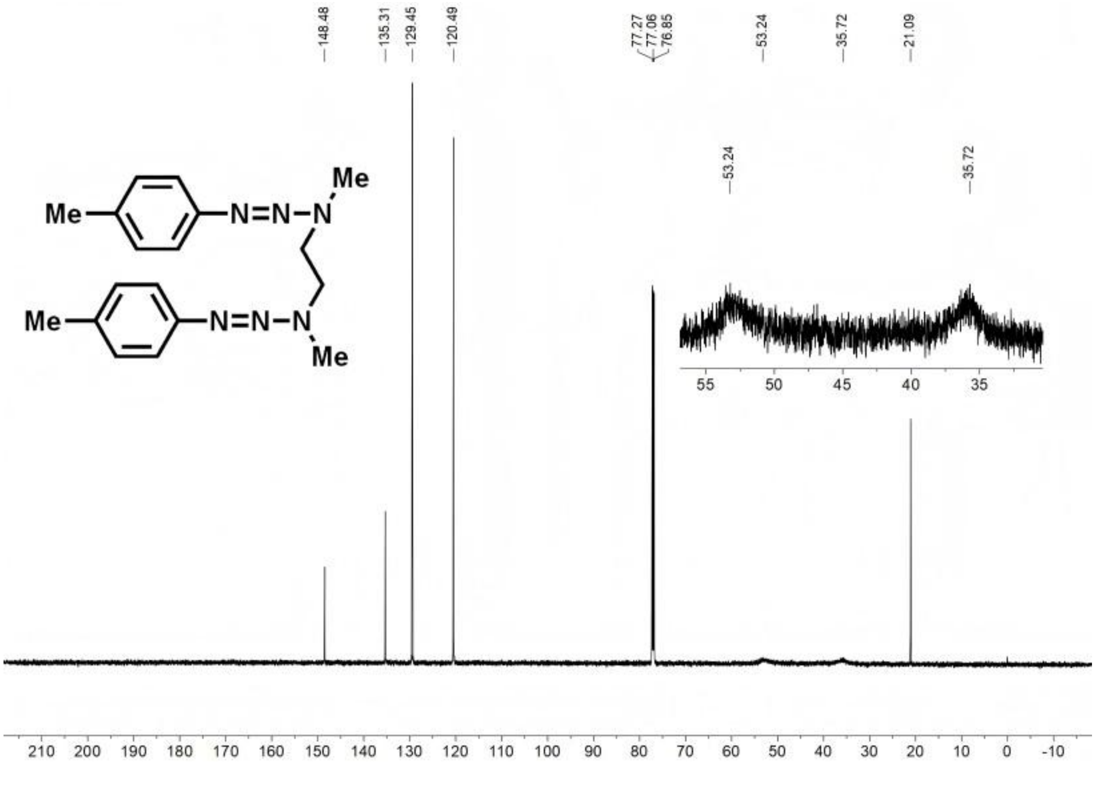

Fig. S33A: ^{1}H NMR of product **5b** in $CDCl_3$ (600 MHz)

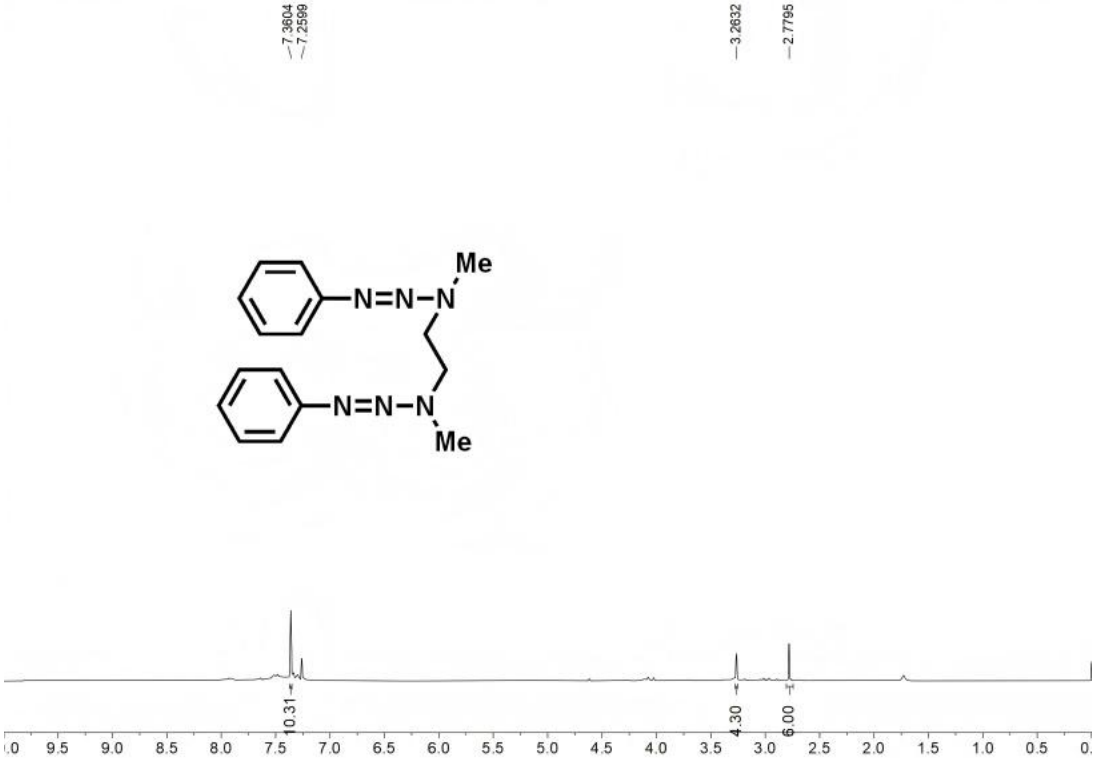

Fig. S33B: ^{13}C NMR of product **5b** in $CDCl_3$ (151 MHz)

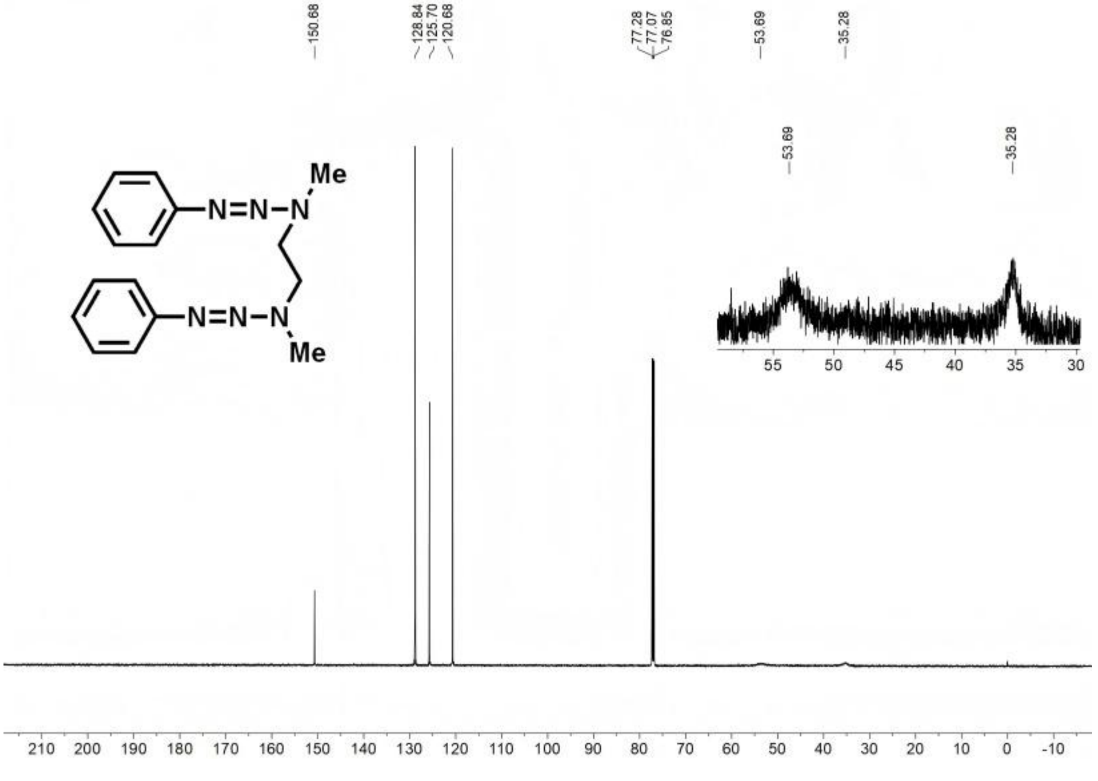

Fig. S34A: ^{1}H NMR of product **5e** in $CDCl_3$ (600 MHz)

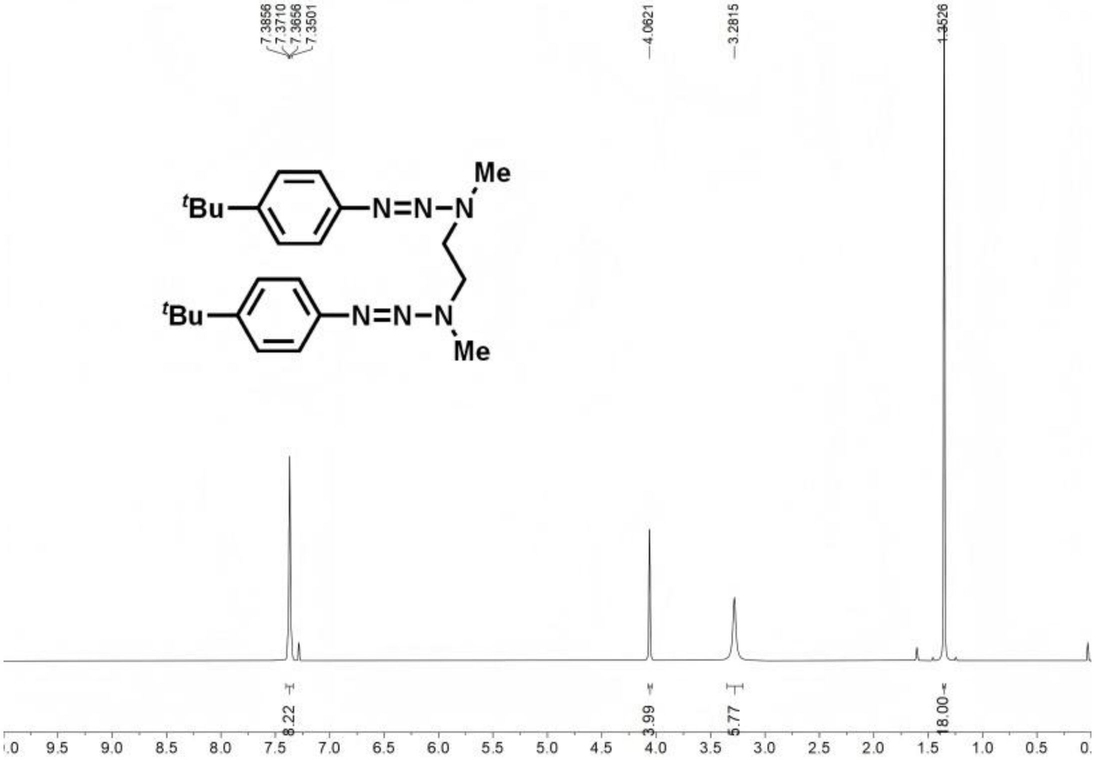

Fig. S34B: ^{13}C NMR of product **5e** in $CDCl_3$ (151 MHz)

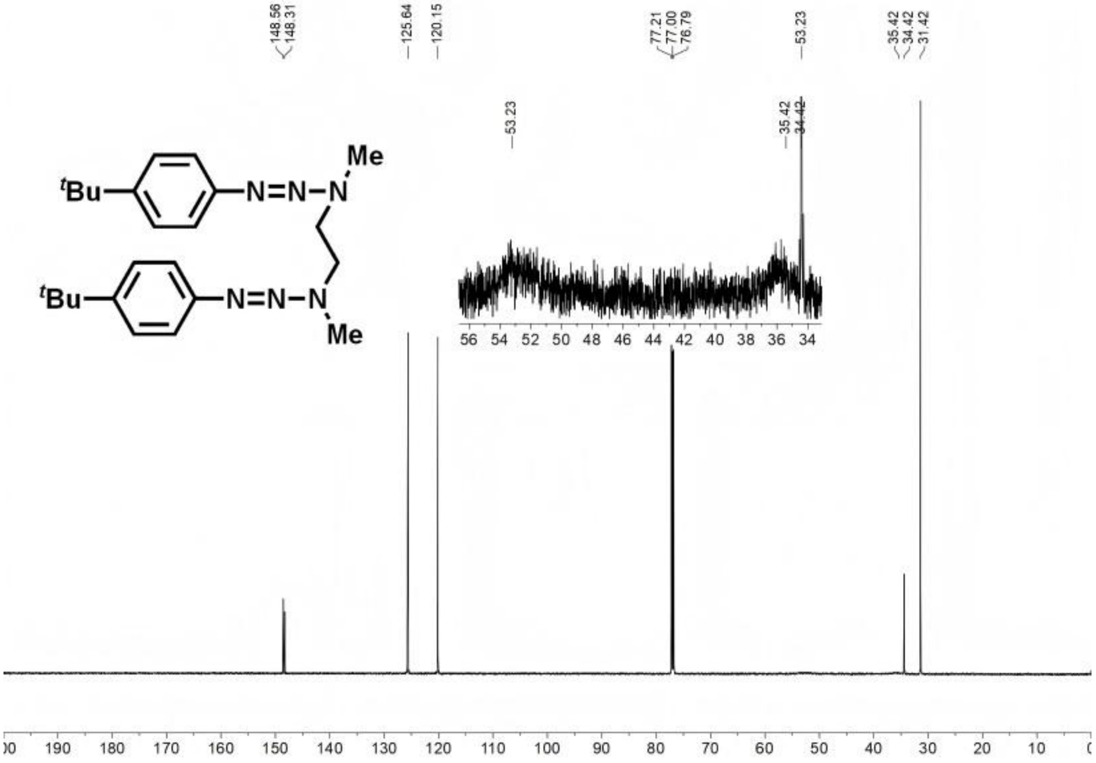

Fig. S35A: ^{1}H NMR of product **5i** in $CDCl_3$ (600 MHz)

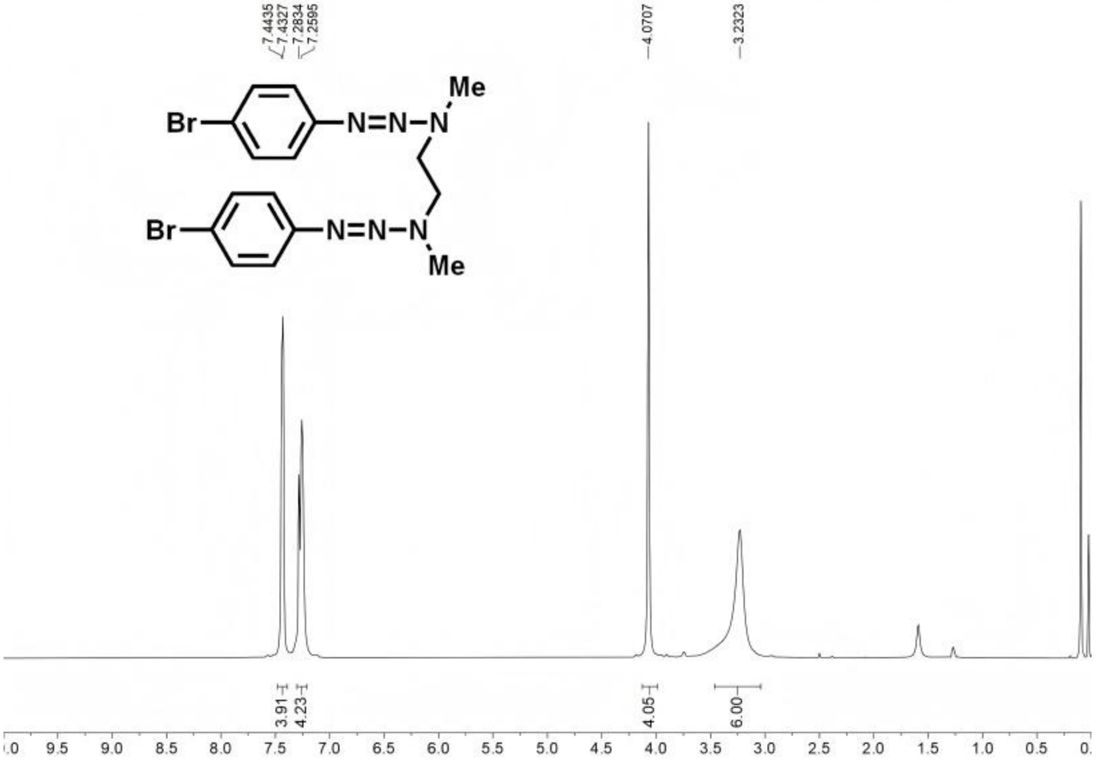

Fig. S35B: ^{13}C NMR of product **5i** in $CDCl_3$ (151 MHz)

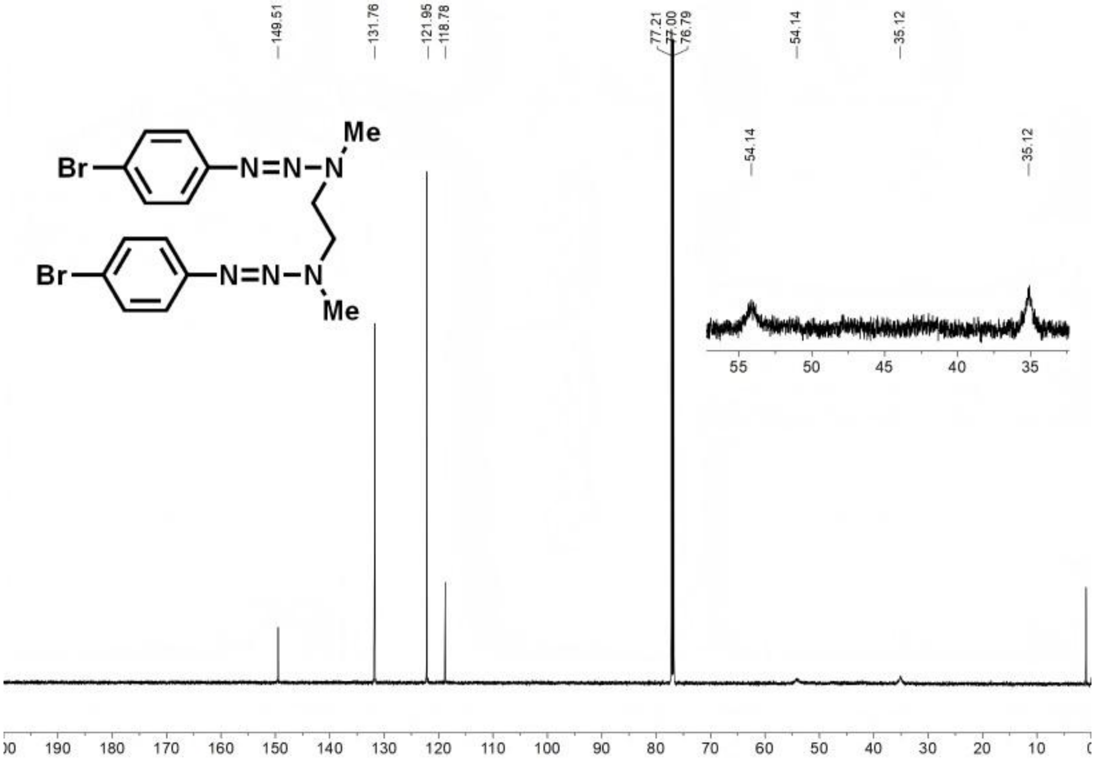

Supplement: Data S2. Copies of NMR spectra [file mmc4.pdf]
